# Radiolabeled Silicon-Rhodamines as Bimodal PET/SPECT-NIR Imaging Agents

**DOI:** 10.3390/ph14111155

**Published:** 2021-11-12

**Authors:** Thines Kanagasundaram, Markus Laube, Johanna Wodtke, Carsten Sven Kramer, Sven Stadlbauer, Jens Pietzsch, Klaus Kopka

**Affiliations:** 1 Helmholtz-Zentrum Dresden-Rossendorf, Institute of Radiopharmaceutical Cancer Research, Department of Radiopharmaceutical and Chemical Biology, Bautzner Landstrasse 400, 01328 Dresden, Germany; t.kanagasundaram@hzdr.de (T.K.); m.laube@hzdr.de (M.L.); j.wodtke@hzdr.de (J.W.); s.stadlbauer@hzdr.de (S.S.); j.pietzsch@hzdr.de (J.P.); 2 Institute of Inorganic Chemistry, Heidelberg University, Im Neuenheimer Feld 270, 69120 Heidelberg, Germany; 3 Radiopharmaceutical Chemistry, German Cancer Research Center (DKFZ), Im Neuenheimer Feld 223, 69120 Heidelberg, Germany; carsten_kramer@gmx.de; 4 Faculty of Chemistry and Food Chemistry, Technische Universität Dresden, Mommsenstrasse 4, 01062 Dresden, Germany

**Keywords:** multimodal imaging, PET imaging, SPECT imaging, optical imaging, organic chemistry, near-infrared fluorophores, silicon-rhodamines, radiochemistry, radiofluorination, radioiodination

## Abstract

Radiolabeled fluorescent dyes are decisive for bimodal imaging as well as highly in demand for nuclear- and optical imaging. Silicon-rhodamines (SiRs) show unique near-infrared (NIR) optical properties, large quantum yields and extinction coefficients as well as high photostability. Here, we describe the synthesis, characterization and radiolabeling of novel NIR absorbing and emitting fluorophores from the silicon-rhodamine family for use in optical imaging (OI) combined with positron emission tomography (PET) or single photon emission computed tomography (SPECT), respectively. The presented photostable SiRs were characterized using NMR-, UV-Vis-NIR-spectroscopy and mass spectrometry. Moreover, the radiolabeling conditions using fluorine-18 or iodine-123 were extensively explored. After optimization, the radiofluorinated NIR imaging agents were obtained with radiochemical conversions (RCC) up to 70% and isolated radiochemical yields (RCY) up to 54% at molar activities of g.t. 70 GBq/µmol. Radioiodination delivered RCCs over 92% and allowed to isolate the ^123^I-labeled product in RCY of 54% at a molar activity of g.t. 7.6 TBq/µmol. The radiofluorinated SiRs exhibit in vitro stabilities g.t. 70% after two hours in human serum. The first described radiolabeled SiRs are a promising step toward their further development as multimodal PET/SPECT-NIR imaging agents for planning and subsequent imaging-guided oncological surgery.

## 1. Introduction

Fluorescent materials such as quantum dots, inorganic and organic fluorophores are highly sought for biomedical (imaging) applications [1,2,3,4,5]. In general, the fluorophores are often utilized as agents in high resolution live-cell and organism imaging [6,7,8]. Especially the real-time visualization of molecular and physiological processes in living cells, staining of tissue and cell compartments (e.g., nuclei, mitochondria or lysosomes) or the characterization of proteins are in emerging demand [9,10,11]. Moreover, various approved organic dyes such as fluorescein or methylene blue have successfully found their way into clinical application for sentinel lymph node biopsy and image-guided surgery [12,13,14,15,16,17]. The fluorescent materials show unique optical properties and the capability in molecular finetuning through chemical modifications, tailored to the required applications [11,18]. On the other hand, common organic dyes suffer from low water solubility, short fluorescence lifetimes, enhanced photobleaching, autofluorescence and limited photochemical brightness [11,19]. However, the chemical adjustment of the electronic and optical properties and the possibility to actively target biomolecules or cellular compartments make organic dyes powerful for biological (imaging) applications [20]. The most biomedically relevant and traditionally used organic fluorophores are fluorescein, BODIPY- and squaraine-dyes [11,15,19]. These organic dyes possess unique properties such as high molar extinction coefficients, moderate quantum yields in aqueous solution and distinct biocompatibility, making them highly suitable for biological applications [11,15]. However, these organic dyes exhibit enhanced light scattering and suffer from optical properties in the visible light area of the electromagnetic spectrum (typical wavelength range between 400 nm and 650 nm) [11,19,21]. Most of the endogenous chromophores (i.a. DNA, melanin, lipids or proteins such as hemoglobin) in tissue, blood and water have large absorption coefficients in the visible wavelength spectrum including emission bands overlapping with the emission maxima of fluorescent dyes. Consequently, these dyes are not suitable for in vivo imaging applications [22,23,24]. A way to circumvent this issue is the utilization of near-infrared (NIR) absorbing and emitting organic dyes due to the minimized overlap of optical properties with endogenous chromophores and water [22,25,26,27,28]. Cli- nical NIR light with a wavelength in the optical window between 700 nm and 1000 nm provides the possibility for deep tissue penetration with significant lower tendency for tissue scattering and photodamaging effects to cells and tissue due to the lower radiation energy [5,22,24,29,30,31,32].

These features promise NIR dyes as important tools for high resolution in vivo (tumor) imaging [19]. So far, some relevant NIR dyes are represented by the family of indocyanine (Cy) dyes as the FDA approved indocyanine green (ICG) and methylene blue (MB), AZA-BODIPYs, phthalocyanines or porphyrin dyes [11,19,33,34,35,36,37,38]. These dyes illustrate alternative representatives to bright, shortwave imaging probes. However, some of these dyes exhibit limited water solubility with undesired aggregation in aqueous solution and low aqueous quantum yields and photostability which restrict the usage of these fluorophores for bioimaging purposes [11,19,39,40]. For all these reasons, there is an emerging demand for optical probes with low molecular weight, required hydrophilicity for biological applications and strongly red-shifted absorbing and emitting characteristics. This necessity is clarified in a rising development phase of novel NIR fluorophores in recent years. From 2011 on Nagano and coworkers introduced novel fluorophores with NIR properties belonging to the rhodamine family [41,42,43]. The new class of fluorophores are named silicon-rhodamines (SiRs) with absorption and emission properties at nearly 650 nanometers. So far, several synthesis methods are exhaustively reported for SiRs [42,44,45,46,47,48,49,50]. Among other fluorescent dyes, SiRs exhibit biocompatible characteristics: enhanced photostability and high quantum yields, water solubility, reduced autofluorescence and the ability to be simply coupled to biological targeting vectors [41,42,43,44,46].

The combination of molecular imaging techniques, e.g., positron-emission tomography (PET) or single photon emission computed tomography (SPECT) with optical ima- ging (OI) is a powerful tool for non-invasive preoperative visualization of tumors followed by image-guided intraoperative R0 resection and postoperative treatment monitoring [51,52]. The radiolabeled fluorophores as dual imaging probes combine the advantages of both modalities. Whereas nuclear imaging methods comprise (semi)quantitative non-invasive three-dimensional whole body imaging with high sensitivity and high tissue penetration, the low spatial resolution in the millimeter range can be balanced out by optical imaging methods [51,53,54]. Vice versa, the low tissue penetration of optical imaging methods can be compensated by using nuclear imaging techniques through high penetration properties. This ideal cutting-edge concept leads to synergistic effects with high tissue penetration and spatial resolution overviewing the whole body and the subcellular compartments. In the literature, multiple PET and SPECT radionuclides were attached to small molecular fluorophores to perform multimodal imaging [51,53,55].

**Figure 1:**
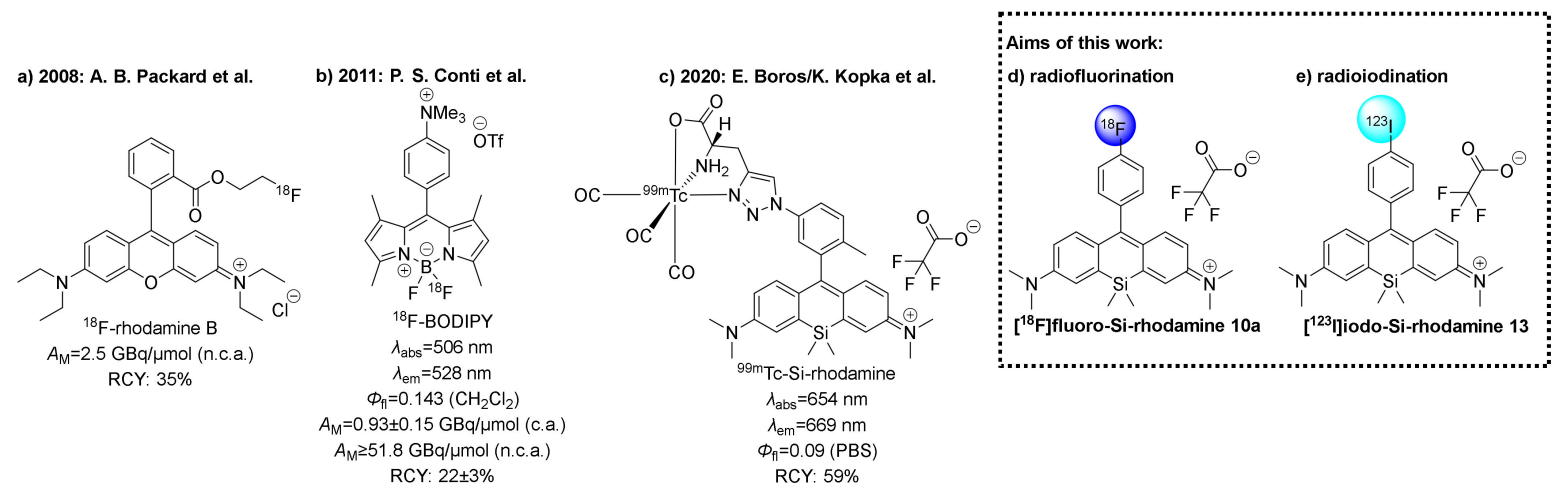
Overview of bimodal PET/SPECT-OI imaging agents and their properties: (**a**) radiofluorination of rhodamine B and (**b**) BODIPY, (**c**) a technetium-99m labeled SiR. The aims of this work: (**d**) a radiofluorinated SiR **^18^F-10a** and (**e**) a radioiodinated SiR **^123^I-13** for multimodal PET/SPECT-NIR imaging [60,61,62,63].

Initially, we focused our work on fluorine-18 radiolabeled dyes due to the excellent properties and extensive utilization of radiofluorine in PET imaging. Especially the short half-life (*t*_1/2_ = 109.8 min), high sensitivity in the nano- to picomolar range and the fast access from compact cyclotrons make radiofluorine attractive for imaging purposes. Further the suitable positron energy (*E*_max_ = 0.634 MeV) with a short positron range of <2.4 mm leads to the superior high-resolution PET images compared to other conventional PET radionuclides [56,57]. In Figure 1 a selection of radiolabeled dyes are shown. Whereas a radiofluorinated rhodamine B as the first radiofluorinated rhodamine representative was applied only for myocardial perfusion imaging from 2008, the first radiofluorinated pure PET/OI imaging probe belonging to the BODIPY family have been presented and characterized from 2011 onwards by Conti et al. and later by Weissleder et al. (Figure 1a,b) [58,59,60,61,62,63]. Since that time several classes of dyes have been radiofluorinated and characterized for PET and OI purposes [53,55]. With exception of our recently reported work of the first technetium-99m labeled SiR, no further radiolabeling on SiRs has been published yet (Figure 1c) [60].

In the present work, we describe the synthesis and optical characterization of novel SiR derivatives and their efficient copper mediated radiolabeling with ^18^F- and ^123^I (Figure 1d,e). Further, stability studies using two ^18^F-labeled SiRs as well as cell uptake studies with selected SiR derivatives were performed as a basis for the development of this class as bimodal imaging agents. The convenient (radio) synthetic access and the promising properties warrants the future development of these compound class.

## 2. Results and Discussion

### 2.1. Chemical Synthesis

Radiolabeling of SiR derivatives with fluorine-18 was envisaged by the modern copper-mediated radiofluorination (CMRF) approach which principally allows radiolabeling of electron-rich precursor molecules that are unreactive under classical nucleophilic aromatic substitution conditions [64,65,66]. Based on considerations regarding the synthetic access, reactivity, and future modifications with targeting vectors, we chose boronic acid as leaving group for the design of the precursor molecules and located them on the phenyl ring in the SiR backbone. Furthermore, we aimed for the design of *para*-(**6a/10a**), *meta*-(**6b/10b**) and *ortho*-(**6c/10c**) substituted precursor and reference compounds to carefully investigate the influence of the position on optical properties and the reactivity of these modifications under radiofluorination reactions.

**Scheme 1:**
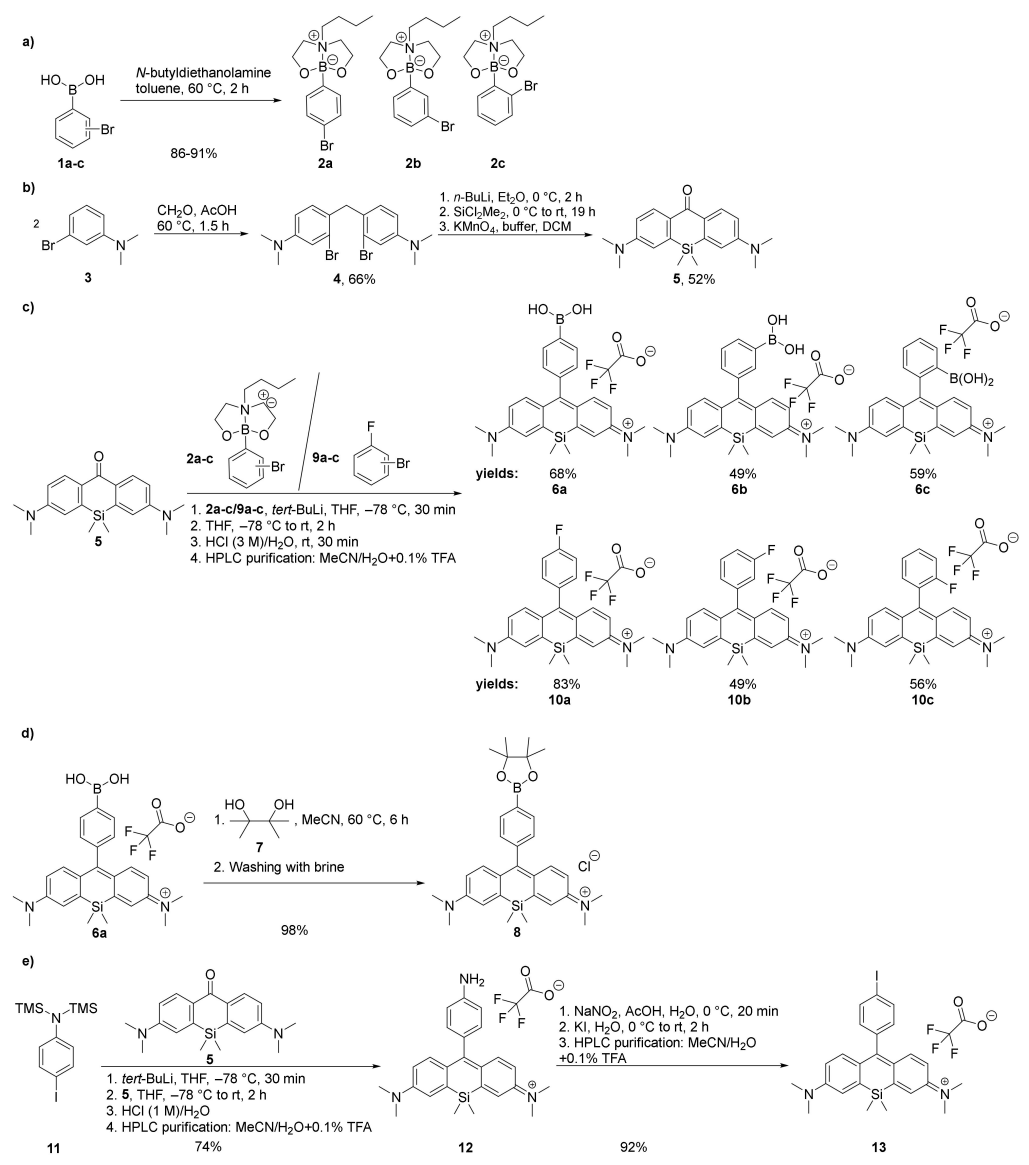
Development of precursors and non-radioactive reference compounds for the copper-mediated radiofluorination (CMRF) and radioiodination (CMRI). (**a**) Synthesis of the protected boronates **2a**–**c** from commercially available brominated phenyl boronic acids **1a**–**c** [69]. (**b**) Two-step synthesis of Si-xanthone **5** [54]. (**c**) The nucleophilic addition of the lithiated intermediates on the Si-xanthone **5** scaffold, followed by elimination to obtain the precursors **6a**–**c** and the non-radioactive reference compounds **10a**–**c**. (**d**) The synthesis of a phenylboronic acid pinacol ester functionalized SiR for copper-mediated radiofluorination. (**e**) The synthesis sequence to yield the iodinated SiR **13** as non-radioactive reference compound via an amine-functionalized SiR **12**.

For comparison, the *para*-substituted boronic acid pinacol ester SiR **8** was developed as another potential precursor for radiolabeling under CMRF conditions. Furthermore, boronic acids represent potential starting materials for copper-mediated radioiodination (CMRI) so that for this radiolabeling approach no additional precursor molecules were designed, only the *para*-substituted SiR **13** was envisaged [67,68]. The synthesis plan of boronic acid functionalized SiRs is shown in Scheme 1.

Initially, the commercially available regioisomers of the brominated phenyl boronic acids **1a**–**c** were converted in yields of 86% to 91% into the *N*-butyldiethanolamine functionalized phenyl boronates **2a**–**c** to protect the boronic acid groups against the harsh conditions of the subsequent lithiation reaction [69].

The Si-xanthone **5** was obtained starting from 3-dimethylamino-bromobenzene in a two-step synthesis and a yield of 52% [49]. The boronic acid functionalized SiRs and the corresponding fluorine-functionalized non-radioactive SiRs were obtained in a nucleophilic addition reaction with the in situ lithiated intermediates with Si-xanthone **5** followed by an elimination reaction in moderate yields (39–83%). It is worth to note, that the usage of *tert*-BuLi instead of the more nucleophilic *n*-BuLi for lithiation leads to higher yields of the boronic acid-functionalized SiRs compared to previously published results [70]. The presented SiRs were purified in a maximum of 120 mg scale on a normal phase silica column with a solvent mixture of dichloromethane/methanol (addition of acetic acid for boronic acid functionalized SiR) followed by reversed-phase HPLC purification to verify the right counter ion (trifluoroacetate ion) and to increase the purity as well. As an alternative to boronic acid functionalized SiRs, **6a** was converted in a facile double condensation reaction with pinacol **7** to the corresponding boronic acid pinacol ester functionalized SiR **8**. From literature it is known, that pinacol esters are used for copper-mediated radiofluorination as well [64,66]. Moreover, the synthesis of the non-radioactive iodinated SiR **13** was performed in a two-step reaction starting from the in situ formed trimethylsilyl-(TMS) protected aniline to obtain the amine-functionalized SiR **12** in 74% yield followed by a Sandmeyer-type reaction to form SiR **13**. SiR **13** was afforded in a yield of 92% after HPLC purification. All the synthesized compounds were unambiguously characterized with ^1^H-, ^11^B-, ^13^C- and ^19^F-NMR, IR-spectroscopy as well as mass spectrometry. Additionally, the optical data of the SiRs were measured.

### 2.2. Optical Properties

In Table 1 the optical properties of all presented SiRs in phosphate-buffered saline (PBS) as well as in dimethyl sulfoxide (DMSO) are shown. The plots of normalized absorbance and emission intensity for the appropriate SiRs are illustrated in the Supporting Information (see Supplementary Materials, Figures S59–S78). In general, the absorption and emission maxima of the SiRs are in the far-red to NIR region, between 640 nm and 680 nm. In contrast to non-NIR dyes (e.g., Rhodamine B or fluorescein) the redshift of the absorption and emission properties is beneficial for appliance in NIR-imaging due to the tissue-conserving optical properties and makes them useful for fluorescence-guided surgery.

The optical properties show a strong bathochromic shift in DMSO compared to the polar PBS solution, possibly due to negative solvatochromic effects. The absorption and emission spectra show the typically shaped mirror pattern that is distinctive for SiRs [42]. Further the Stokes shifts remain small (14 nm to 23 nm) which is a hint for a small and negligible difference in dipole moments between the energetical ground state and the excited state of the SiRs. The molar absorption coefficients of the SiRs show higher values in polar aprotic DMSO compared to the more relevant aqueous PBS for biological applications.

Especially **6c** shows a significant decrease in absorption and consequently in brightness due to an equilibrium between an open fluorescent zwitterionic and a closed colorless spiroboronate form [70]. This equilibrium effect is not given in **10c** resulting in significantly higher molar absorption coefficients either in DMSO or in PBS. The remarkable effect between an open fluorescent and a closed non-fluorescent equilibrium followed by a reduction in absorption in SiR analogues has been extensively reported in live-cell imaging applications in recent work and for this reason no further efforts were made to clarify this phenomenon [41,44,71].

Similar trends are observed for the relative quantum yields (*Φ*_fl_) of all SiRs determined in DMSO and PBS (*pH* = 7.4) with the commercially available Nile Blue A as reference fluorophore. The quantum yields for the relevant bimodal imaging purposes and non-radioactive SiRs **10a**–**c** are lying between 0.08 and 0.22 in PBS (vs. 0.12–0.15 in DMSO). The highest quantum yields were measured for the *ortho*-substituted SiRs **6c** (*Φ*_fl_ = 0.20 in 0.1 M HCl) and **10c** (*Φ*_fl_ = 0.22 in PBS) possibly due to the well-known and in previous work intensively discussed inhibition of the photoinduced electron transfer through steric hindrance in *ortho*-substituted SiRs [42]. Compared to the fluorinated SiRs **10a**–**c** the optical properties of the corresponding non-radioactive SiR **13** shows nearly the same optical properties with an absorption maximum at 648 nm and an emission maximum at 667 nm in PBS, indicating negligible influence of the iodine on optical properties compared to the fluorinated counterpart **10a**. The aqueous quantum yield is slightly higher than that of the corresponding fluorinated SiR **10a**. Further a significantly lower molar absorption coefficient possibly indicates a higher tendency to aggregation formation in aqueous solution due to the lipophilic nature of **13** (41.100 M^−1^cm^−1^). The determined aqueous quantum yields of the non-radioactive reference compounds are comparable to the FDA approved dyes, i.e., the 5-aminolevulinic acid (5-ALA) induced protoporphyrin IX (PPIX; *Φ*_fl_ = 8%) or the NIR-fluorophore indocyanine green (ICG; *Φ*_fl_ = 9%) [15]. Moreover, the non-radioactive dyes show higher aqueous quantum yields in relation to other small molecular and bimodal radiofluorinated PET/OI imaging agents [53].

Compared to nanoparticles, most of the organic fluorescent dyes show limited photostability after (electromagnetic) irradiation [72].

**Figure 2:**
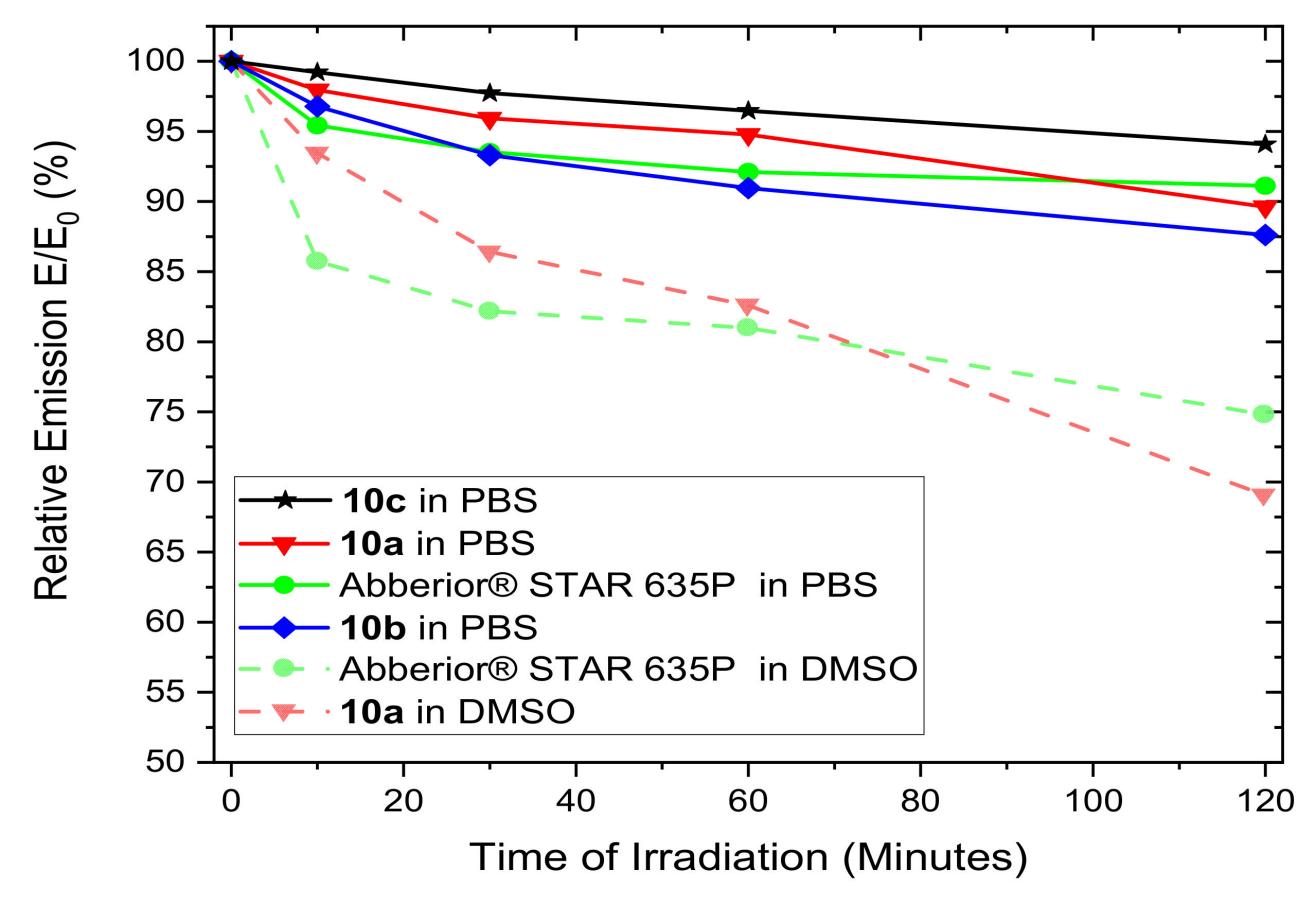
Summary of the photostability experiments of the non-radioactive fluorinated SiRs **10a**–**c** and Abberior^®^ STAR 635P after irradiation with a wavelength of 640 nm of the samples with a pulsed laser (20 kW, pulse width at half peak height < 10 μs) up to two hours in PBS (+1% DMSO) or in pure DMSO. The concentration of the samples was kept between 5 µM and 10 µM.

Since for clinical applications it is mandatory to use highly photostable fluorophores we investigated the photobleaching properties of the non-radioactive dyes **10a**–**c** in PBS or DMSO (Figure 2).

The commercially available and photostable Abberior^®^ STAR 635P was used as reference. The fluorescence experiments were performed with a pulsed laser and an irradiation wavelength of 640 nm for a period of two hours at room temperature. Distinctive for SiRs the dyes show high photostability in PBS [43]. In this series, a maximum degradation of only 12% for **10b** was observed after two hours irradiation. The dyes **10a**,**c** showed even less than 10% degradation under similar conditions. It is worth to note, that in DMSO as solvent SiR **10a** (degradation: 31%) and the reference dye Abberior^®^ STAR 635P (degradation: 25%) showed higher photobleaching properties under identical irradiation conditions. Compared to some traditional dyes (e.g., fluorescein or indocyanine) the synthesized SiRs show a superior photostability which can be helpful in fluorescence-guided surgery [15,73,74].

### 2.3. Radiofluorination

**Scheme 2:**
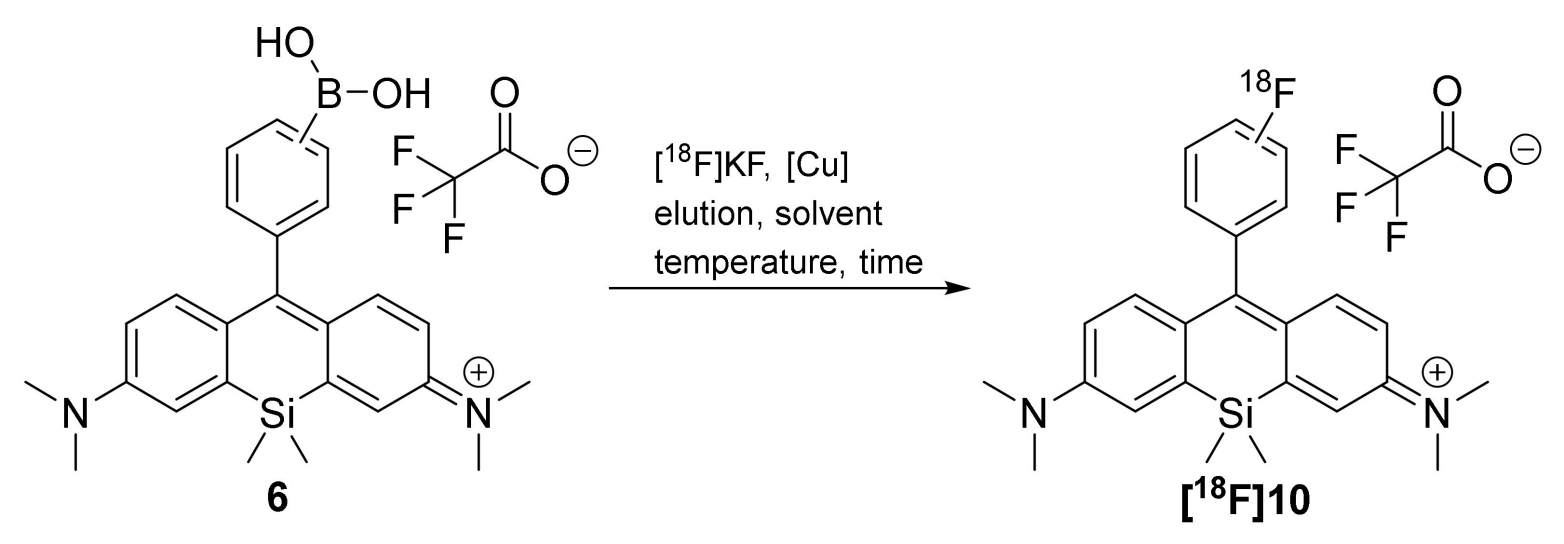
Optimization of the copper-mediated radiofluorination of the boronic-acid functionalized SiRs **6** to **[^18^F]10** under different conditions.

The radiolabeling of the boronic acids **6a**–**c** and boronic acid pinacol ester **8** functionalized SiRs with [^18^F]fluoride was extensively evaluated utilizing the copper-mediated radiofluorination reactions (Scheme 2). The symmetric SiR **10a** was used to optimize the radiolabeling conditions for this compound class. In Table 2 different conditions of the copper-mediated radiofluorination of **6a**–**c** and **8** are summarized.

The screening of the copper-mediated radiofluorination experiments was carried out utilizing a recently presented microliter scale radiofluorination approach in HPLC vials instead of conventional reaction volumes in the 0.5–1 mL scale due to limited amounts of precursor [75].

This strategy allows to perform reactions in parallel and volumes of only 25–50 µL and helps to increase the number of reactions in a limited time by working with fluorine-18 and supports to screen a reaction quickly.

Moreover, the decreased need of reactants and solvents through microliter scale reactions saves precious precursor and can ease purification procedures. Possibly, higher molar activities can result from the lower probability of [^19^F]fluoride contamination from reactants and solvents [76]. At first, several radiofluorination methods of aryl boron sources known from the literature were chosen and were adapted for the radiolabeling strategy for the SiRs **6a**–**c** and **8** [64,65,66,77,78].

The screening methods included reactions with and without azeotropic drying steps, different reaction temperatures and reaction times, various solvents and different amounts of precursor in relation to the copper agents (Table 2). To evaluate the radiolabeling, radiochemical conversions (RCC) were calculated via radio-HPLC or radio-TLC. Isolated radiochemical yields (RCY) were determined for selected optimized conditions.

In the first set of experiments (Table 2, entries 1–12), [^18^F]fluoride was eluted from a weak anion exchange (WAX) cartridge with 4-(dimethylamino)-pyridinium triflate (DMAPH^+^OTf^−^) according to a procedure recently described for CMRF by Antuganov et al. [78]. Radiolabeling of the symmetric SiR **6a** (1 equiv.) was then performed without an azeotropic drying step utilizing the copper agent tetrakis(pyridine)copper(II) triflate [Cu(OTf)_2_(py)_4_].(4 equiv.) in dimethylacetamide (DMA) at 100 °C for 20 min. As determined by radio-HPLC a radiochemical conversion of only 3% was observed (entry 1). This radiofluorination reaction was optimized by using various temperatures and reaction times (entries 1–3; 6–10). Further analysis showed that the optimum conditions for successful **[^18^F]10a** formation are at 120 °C combined with 20 min reaction time and a four-fold molar excess of copper agent in relation to the precursor (entry 3). Under these conditions the RCC was 25% and the isolated average RCY after HPLC purification was 14.0 ± 0.3%. Radiochemical purity of the isolated radiolabeled products in these and for later described experiments was found to be higher than 99% according to HPLC analyses. Higher temperatures and longer reaction times led to decreased RCC for **[^18^F]10a** (see Supplementary Figure S4 and Table S3). Surprisingly, the replacement of the solvent DMA with dimethylformamide (DMF) resulted in a complete loss of reactivity and hence no conversion to the desired product (entry 4) indicating the necessity of DMA for successful radiofluorination of SiRs. Further changing the molar ratio of copper agent and precursor to a 1:4 excess of precursor also resulted in absence of product formation (entry 5). The optimized conditions (entry 3) were utilized for the radiolabeling of the regioisomers **6b** and **6c** as well as the boronic acid pinacol ester **8** (entries 11, 12 and 25). No radiochemical conversions to the ^18^F-labeled products were observed indicating the marked influence of electronical effects or sterical hindrance that directly influences the radiofluorination of SiRs. Notably, in case of **6c** the loss of reactivity might additionally be caused by the formation of a closed spiroboronate form of the *ortho*-substituted SiR under basic reaction conditions.

The elution of [^18^F]fluoride with tetraethylammonium bicarbonate in n-butanol from a quaternary methyl ammonium (QMA) cartridge (entries 13–17) and radiofluorination under the previous optimized reaction conditions showed a maximum RCC of 16% and an isolated RCY of 15% after HPLC purification for the radiofluorination of **6a** (entry 13) [65]. Compared to previous reactions varying both the amount of copper agent and precursor **6a** (1:4 and 1:1) led to RCCs between 4% and 12% which indicates the beneficial effect of alcohols for copper-mediated radiofluorination reactions (entries 14 and 15) as recently reported [65]. However, under these conditions radiofluorination was not observed for either **6b** or **6c** (entries 16 and 17).

As an alternative approach the elution step was carried out with an aqueous mixture containing acetonitrile, potassium triflate and potassium carbonate over a QMA cartridge (entries 18–26) [64]. The removal of water to obtain highly reactive nucleophilic [^18^F]fluoride was achieved by a single dry-down step using a gentle flow of helium at 130 °C. Under these conditions the highest RCCs were achieved for SiR radiofluorination. This elution method combined with the reaction under previously optimized conditions provided RCCs up to 35% and isolated RCY of 25 ± 4% (entry 18). A simple substitution of DMA with the urea-based solvent 1,3-dimethyl-2-imidazolidinone (DMI) led to an increased RCC of 70% [79,80]. Under these conditions and a five-fold higher reaction volume of 200 µL an isolated RCY of 54 ± 1% for **[^18^F]10a** was observed (entry 19). Notably, the optimized conditions successfully yielded the radiolabeled regioisomer **[^18^F]10b** in 33% with high radiochemical purity (>99%; entry 20). However, no radiochemical conversion was observed for either **6c** or the pinacol ester functionalized SiR **8** (entries 21 and 26). Additionally the combination of copper agent [Cu(OTf)_2_(impy)_4_] and DMA showed no conversions of **6c** and **8** whereas **[^18^F]10a** was obtained with a RCC of 18% (entries 22–24) [79,80]. Further data for elution and other reaction conditions are listed in the Supporting Information.

**Figure 3:**
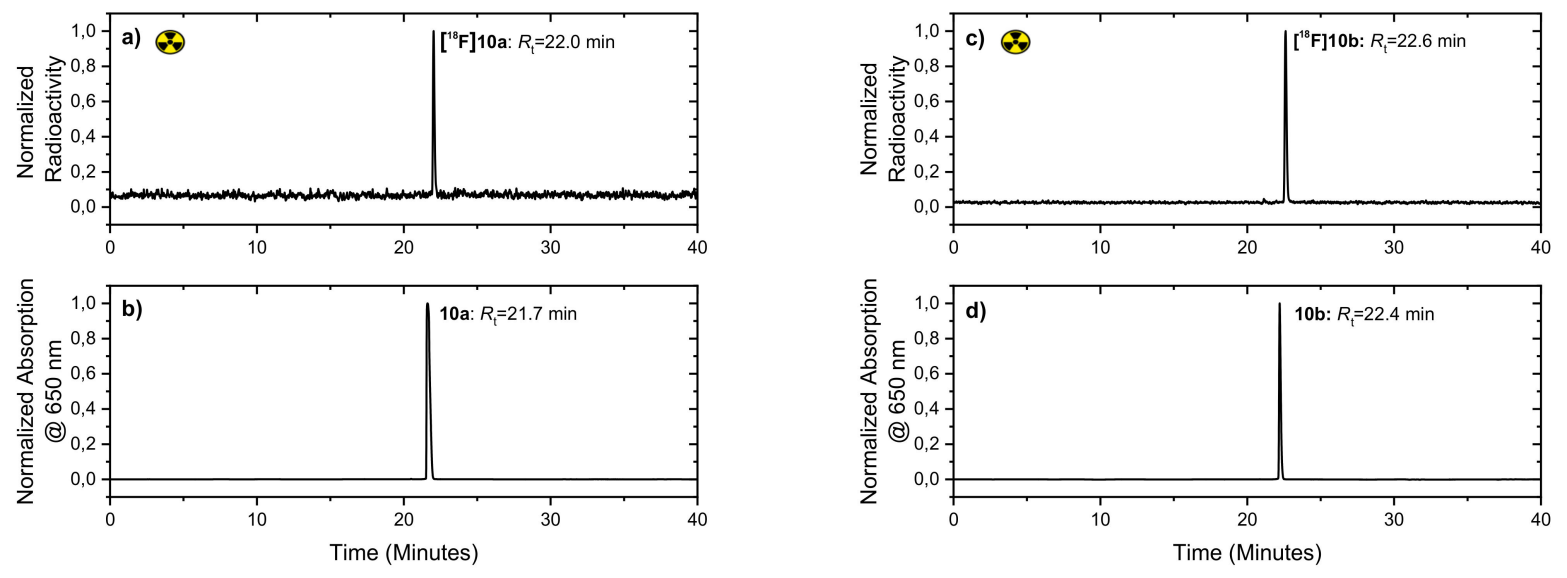
Confirmation of identity of the radiochemical pure SiRs **[^18^F]10a** and **[^18^F]10b**. (**a**) Normalized HPLC chromatogram of the purified radiofluorinated SiR **[^18^F]10a** ([*γ*-Detection], *R*_t_ = 22.0 min) and (**b**) Normalized HPLC chromatogram of the purified non-radioactive reference SiR **10a** ([NIR absorption, 650 nm], *R*_t_ = 21.7 min). (**c**) Normalized HPLC chromatogram of the purified radiofluorinated SiR **[^18^F]10b** ([*γ*-Detection], *R*_t_ = 22.6 min) and (**d**) Normalized HPLC chromatogram of the purified non-radioactive reference SiR **10b** ([NIR absorption, 650 nm], *R*_t_ = 22.2 min). Method: 75% water (containing 0.1% TFA) and 25% acetonitrile to 25% water (containing 0.1% TFA) and 75% acetonitrile (system 5, HPLC 25–75).

In Figure 3 the HPLC chromatograms of the purified radioactive SiRs **[^18^F]10a**, **[^18^F]10b** and the respective non-radioactive reference SiRs **10a** and **10b** are shown. Similar retention times of the radioactive SiRs **[^18^F]10a** or **[^18^F]10b** compared to their respective non-radioactive references (**10a** or **10b**) in HPLC prove the identity as well as the high radiochemical purity. Appropriate NIR absorption traces at 650 nm are shown in the Supporting Information (see Supplementary Material Figure S5).

After HPLC purification, solid phase extraction for solvent exchange and formulation in ethanol, **[^18^F]10a** and **[^18^F]10b** were further characterized by determination of the partition coefficient using the conventional shake-flask method [81,82]. The experimentally determined value for **[^18^F]10a** was $\log D_{\mathrm{pH}=7.4}$ = 2.92 ± 0.32 whereas **[^18^F]10b** shows a slightly enhanced lipophilic character with $\log D_{\mathrm{pH}=7.4}$ = 3.22 ± 0.18.

The partition coefficients of both SiRs show a highly lipophilic nature of the cationic SiRs which should be taken in account for further derivatization to bioconjugateable SiRs.

The evaluation of the molar activities of **[^18^F]10a** and **[^18^F]10b** showed comparable high values for both radiofluorinated compounds with 70.1 ± 3.2 GBq/µmol (*n* = 7) for **[^18^F]10a** and 42.8 ± 9.5 GBq/µmol (*n* = 2) for **[^18^F]10b**, both after the end of synthesis (EOS).

The results show that significantly higher radiochemical yields and molar activities of the SiRs **[^18^F]10a** (RCY: 54 ± 1%; *A*_m_ ≥ 70.1 ± 3.2 GBq/µmol (n.c.a.)) and **[^18^F]10b** (RCY: 33%; *A*_m_ ≥ 42.8 ± 9.5 GBq/µmol (n.c.a.)) can be achieved in comparison to the dyes ^18^F-rhodamine B (RCY: 35%; *A*_m_ = 2.5 GBq/µmol (n.c.a.)) and ^18^F-BODIPY (RCY: 22 ± 3%; *A*_m_ ≥ 51.8 GBq/µmol (n.c.a.)) [51,53]. These features make the novel radiofluorinated SiRs as far-red to NIR dyes attractive for future active targeting purposes for selective enrichment in tumor tissues.

For investigation of stability of the novel radiotracers, both compounds were dissolved in a solution of 0.9% saline and were kept at room temperature. Based on HPLC, the chromatograms did not show any degradation after two hours, indicating high stability in aqueous solution. However, in vitro human serum stability tests of the radiolabeled dyes showed a certain instability of **[^18^F]10a** (degradation: 20%) and **[^18^F]10b** (degradation: 26%) after two hours incubation in human plasma at 37 °C (Supplementary: Tables S11 and S12).

### 2.4. Radioiodination

Recently, radioiodination of a variety of aromatic compounds was found to proceed in high yield under mild conditions starting from aryl boronic acids or pinacol boronic esters using [Cu(OTf)_2_(py)_4_] as well [67,68,83,84].

Starting from boronic acids **6a**–**c** as well as boronic acid pinacol ester **8**, this radiolabeling strategy was hence identified as a quick entrance to radioiodinated SiRs for PET or SPECT imaging with their corresponding radioactive isotopologues: iodine-123 (*t*_1/2_ = 13.2 h), iodine-124 (*t*_1/2_ = 4.2 d) or iodine-125 (*t*_1/2_ = 59.4 d) and radionuclide therapy with iodine-131 (*t*_1/2_ = 8.0 d) [85]. As a model compound we tested precursor **6a** for its capability to be radioiodinated under previously optimized radioiodination conditions employed for the development of a 2-[^123^I]iodophenyl acetyl based transglutaminase-2 inhibitor [86].

**Scheme 3:**
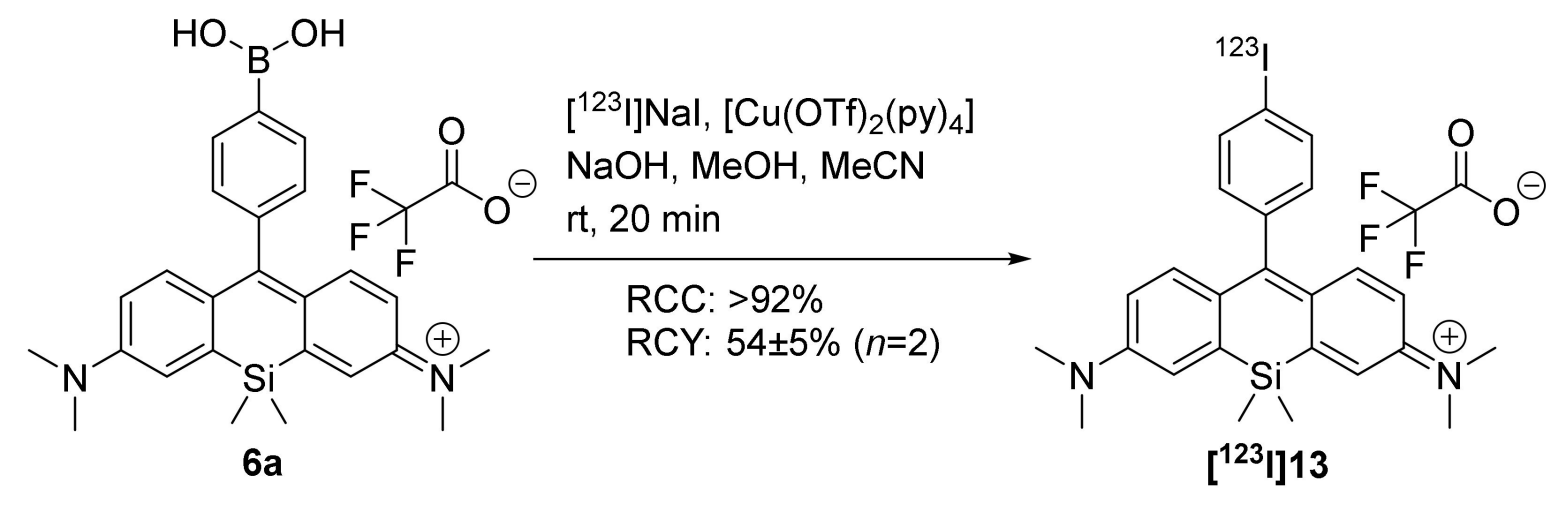
The copper-mediated synthesis of a radioiodinated SiR [**^123^I**]**13** starting from precursor **6a** in a solution mixture of [^123^I]NaI in sodium hydroxide (0.02 M), methanol and acetonitrile with tetrakis(pyridine)copper(II) triflate. The reaction was performed for 20 min at room temperature and radiochemical conversions higher than 92% combined with a radiochemical yield of 54 ± 5% (*n* = 2) after semi-preparative HPLC purification were obtained [86].

The copper-mediated radioiodination of SiR [**^123^I**]**13** was performed in a solution of radioactive [^123^I]NaI in sodium hydroxide, methanol and acetonitrile (Scheme 3).

The reaction was performed at room temperature for 20 min. The radiochemical conversion of the semi-preparative HPLC purified **[^123^I]13** was g.t. 92% with a corresponding isolated radiochemical yield of 54 ± 5% (*n* = 2) after HPLC and SPE purification as well as reformulation in EtOH. The radiochemical purity was found to be 81% and 96% after a similar workup procedure indicating an instability during final evaporation (see Supplementary Figures S15–S18). At this stage, no further optimization of the formulation method was performed because the principal applicability of the labeling procedure could be successfully demonstrated.

**Figure 4:**
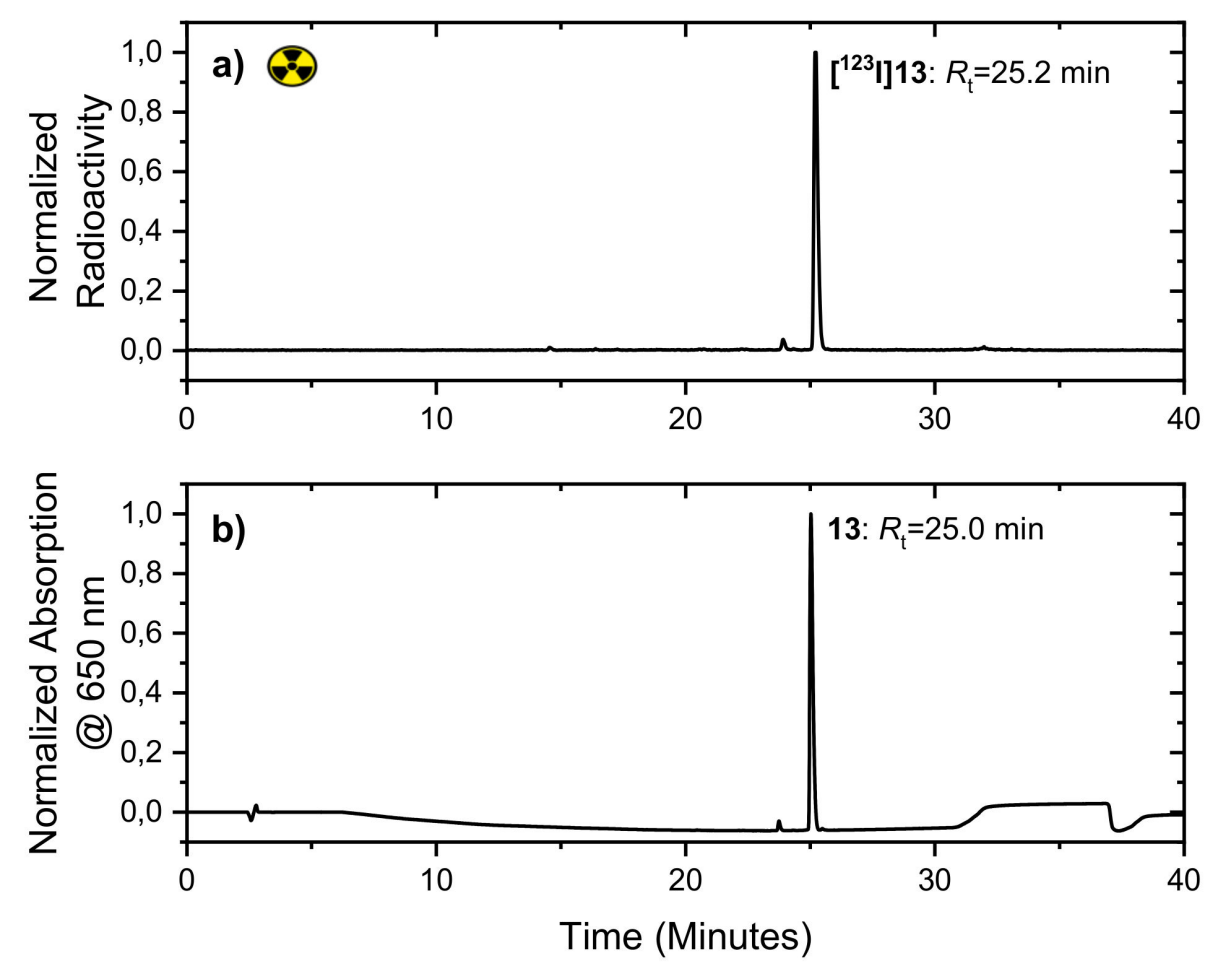
Confirmation of the successful radioiodination of **6a** to **[^123^I]13**. (**a**) Normalized HPLC chromatograms of the radioiodinated SiR **[^123^I]13** ([*γ*-Detection], *R*_t_ = 25.2 min) (**b**) spiked with the non-radioactive reference SiR **13** ([NIR absorption, 650 nm], *R*_t_ = 25.0 min). Method: 75% water (containing 0.1% TFA) and 25% acetonitrile to 25% water (containing 0.1% TFA) and 75% acetonitrile (system 5, HPLC 25–75).

Both, HPLC-traces of the radioactive and NIR absorption at 650 nm of [**^123^I**]**13** spiked with the non-radioactive SiR **13** are shown in Figure 4. Compared to the previous radiofluorination experiments the conditions are milder with enhanced radiochemical conversions and radiochemical yields due to the more reactive iodide-123.

As expected, **[^123^I]13** shows a higher lipophilicity than **[^18^F]10a** and **[^18^F]10b** which can be seen in the difference of the partition coefficients (**[^123^I]13**: ${\log D}_{\mathrm{pH}=7.4}$ = 3.48 ± 0.29). A significantly higher molar activity of **[^123^I]13** was experimentally determined with *A*_M_ = 7.64 ± 0.27 TBq/µmol (*n* = 4) based on a starting activity of 180 MBq. Compared to the radiofluorinated **[^18^F]10a** the radioiodinated SiR demonstrates a 100-fold higher value. To the best of our knowledge, this is the highest value of molar activities observed for radiolabeled NIR fluorophores (EOS). Besides radiofluorination the successful radioiodination shows a new entrance to radiolabeled SiRs for multimodal imaging with unique properties as well.

### 2.5. In Vitro Colocalization Studies in Mitochondria

**Figure 5:**
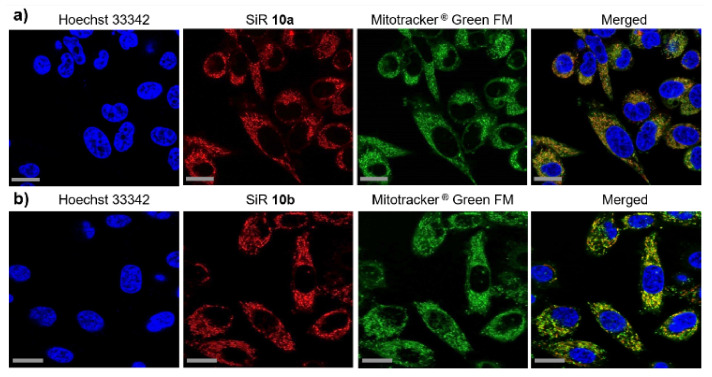
Confocal laser scanning microscopy images of living PC-3 cells through colocalization experiments. The mitochondria were stained with (**a**) SiR **10a** or (**b**) SiR **10b** (red) and the commercially available Mitotracker^®^ Green FM (green) and the cell nuclei were stained with Hoechst 33342 (blue). Afterwards an overlay of the images was acquired. Scale bar: 30 µm.

Colocalization experiments using live-cell imaging with the selected non-radioactive SiRs **10a** and **10b** by confocal laser scanning microscopy were performed to investigate general uptake behavior of SiRs without targeting unit (Figure 5).

The enhanced lipophilic nature of the small molecular SiRs **10a** and **10b**, the delocalized positive charge over the whole rhodamine backbone combined with a negative environment in mitochondrial matrix principally promote mitochondrial internalization as previously reported in detail for (silicon-)rhodamines [53,87,88]. Based on these consideration we used human prostate cancer cells (PC3 cell line) for colocalization experiments with commercially available MitoTracker^®^ Green FM to image and verify selective enrichment in mitochondria. The cell nuclei were stained with Hoechst 33342 (blue). The colocalization studies show a high grade of accumulation of SiRs **10a** and **10b** (red) as well as the MitoTracker^®^ Green FM (green) in mitochondria within few minutes (Figure 5).

The calculation of the Pearson correlation of **10a** (0.79 ± 0.03; *n* = 16) and **10b** (0.75 ± 0.04; *n* = 12) confirm high overlap with MitoTracker^®^ Green FM.

The selective enrichment of the cationic and lipophilic SiRs show similar properties as conventionally used mitochondria imaging agents (e.g., triphenylphosphonium ions) [87,89].

## 3. Materials and Methods

Unless otherwise stated reactions requiring exclusion of oxygen and moisture were carried out in heat-gun dried flasks under argon gas or nitrogen atmosphere using the Schlenk-technique.

All chemicals and solvents were purchased from Sigma-Aldrich Laborchemikalien GmbH, abcr GmbH, Acros Organics and were used without further purification. Deuterated solvents were used from Deutero GmbH. The dry solvents dimethylformamide (DMF), dimethylacetamide (DMA), diethyl ether, methanol and tetrahydrofuran were purchased from Sigma-Aldrich Laborchemikalien GmbH in Sure/Seal™ bottles.

UV-Vis-NIR absorption spectra were measured on a spectrophotometer Specord 50 (Analytik Jena, Jena, Germany) from 300 nm to 800 nm in a quartz cuvette with 1 cm path length. All measurements were performed in dimethyl sulfoxide (DMSO) or phosphate-buffered saline solution (PBS) at room temperature. The fluorescence quantum yields were performed with relative measurements using a reference dye according to the literature [90].

Nile Blue A as a reference standard was used for the determination of the quantum yields [91]. Fluorescence properties were determined using the Perkin Elmer LS-55 fluorescence spectrometer from Perkin Elmer Inc. (Waltham, MA, USA) at room temperature. The excitation wavelength for the samples is account for 600 nm. The data acquired were analysed with OriginPro 2020 (64-bit) SR1.

Photostability experiments were investigated on a Perkin Elmer LS-55 fluorescence spectrometer by using the integrated laser and the irradiation of the samples (c~5 µM for dye **10a**, **10b** or **10c** and c~1 µM for the reference) in a quartz cuvette (1 cm path length) with a pulsed laser of the wavelength of 640 nm (20 kW, pulse width at half peak height < 10 μs) up to a maximum time of two hours. After several time points (10 min, 30 min, 1 h and 2 h) emission spectra were measured on the same device to determine the photostability of the corresponding dye.

High-Performance Liquid Chromatography (HPLC) system was used for semi-preparative purification: Knauer Smartline system (Berlin, Germany) equipped with a Smartline pump 1000, the degasser system Smartline manager 5000 with performance at room temperature, UV-Vis-NIR Smartline detector 2500 for wavelength detection at 254 nm and 650 nm and the C_18_ column from Phenomenex (Gemini^®^, 5 µm, 110 Å, LC Column 250 × 4.6 mm).

The purification was performed with the following systems: (system 1) using the method 10–90% MeCN/H_2_O, linear gradient in 35 min, with constant 0.1% *v/v* trifluoroacetic acid (TFA) additive and a flow rate of 5.30 mL/min or using the method 30–90% MeCN/H_2_O, linear gradient in 35 min, with constant 0.1% *v/v* trifluoroacetic acid (TFA) additive and a flow rate of 5.50 mL/min (system 2).

Radio-HPLC purification of **[^18^F]10a** and **[^18^F]10b** were performed by using a semi-preparative Shimadzu prominence LC20AR equipped with LC-20AR binary gradient module, SIL-10AR sample manager, SPD-M20A PDA detector and gamma-detector LB 500 Herm (Berthold Technologies, Germany). The purification was performed on a Nucleosil^®^ 100-7 C_18_ column (7 µm, 100 Å, LC Column 250 × 16 mm). The data was processed with Labsolution Software V. 5.92. The purification was carried out using an isocratic method with water +0.1% trifluoroacetic acid/acetonitrile 45/55 and a flow rate of 5 mL in 50 min (system 3).

Further, the radio-HPLC purification of **[^123^I]13** was carried out by using a semi-preparative Jasco LC-NetII/ADC interface equipped with a quaternary pump Jasco PU-2089 PLUS with a vacuum degasser, a Jasco UV-2075 detector (Jasco Corporation, Tokyo, Japan) and the gamma spectrometer GABI from Elysia-Raytest GmbH (Straubenhardt, Germany). The purification was performed on a Luna^®^ C_18_ column (10 µm, 100 Å, LC Column 250 × 10 mm). The data was processed through Jasco ChromNAV Software. The purification was carried out using a linear gradient from 10–90% MeCN/0.1% trifluoroacetic acid (TFA) in water H_2_O in 33 min and a flow rate of 5 mL/min (system 4).

(Ultra) High-Performance Liquid Chromatography (UHPLC/HPLC) system was used for analytical purposes: (system 5) Shimadzu Nexera X2 UHPLC system (Shimadzu Corporation, Kyoto, Japan) equipped with a dual pump LC-30AD, on-line degasser DGU-20A_3R_ and DGU-20A_5R_, column oven CTO-20AC with two column switching valves FCV-14AH and a performance at 40 °C, the fluorescence detector RF-20A, an autosampler SIL-30AC, the photodiode array detector (PDA) for detection at wavelengths at 254 nm and 650 nm (SPD-M20A), the communication bus module CBM-20A and the gamma spectrometer GABI from Elysia-Raytest GmbH (Straubenhardt, Germany; detection of fluorine-18: 100–600 keV; detection of iodine-123: 100–200 keV). For the HPLC the analytical C_18_ column Kinetex^®^ from Phenomenex (5 µm, 100 Å, LC Column 250 × 4.6 mm) and for the UHPLC the analytical C_18_ column Kinetex^®^ (1.7 µm, 100 Å, LC Column 50 × 2.1 mm) were used.

The analytical HPLC analysis was performed with the following systems: (system 5) using a linear gradient method with water+0.1% trifluoroacetic acid/acetonitrile at a flow rate of 1 mL/min (HPLC 25–75: *t*_0 min_25/75–*t*_3 min_25/75–*t*_28 min_75/25–*t*_29 min_95/5–*t*_34 min_95/5–*t*_35 min_25/75–*t*_40 min_25/75, total 40 min; HPLC 45–95: *t*_0 min_45/55–*t*_3 min_45/55–*t*_28 min_95/5–*t*_34 min_95/5–*t*_35 min_45/55–*t*_40 min_45/55, total: 40 min) or isocratic mode (HPLC 55 iso: *t*_0 min_45/55–*t*_15 min_45/55).

The analytical UHPLC analysis was performed to determine the radiochemical conversions (RCCs) with system 5 using a linear gradient method with acetonitrile/water+0.1% trifluoroacetic acid (UHPLC 25–75: *t*_0 min_25/75–*t*_0.3 min_25/75–*t*_4.0 min_ 75/25–*t*_4.5 min_ 95/5–*t*_5.5 min_ 95/5–*t*_6.0 min_ 25/75–*t*_7.5 min_25/75; total 7.5 min) at a flow rate of 0.5 mL/min.

The molar activities and the stabilities in saline/human serum of **[^18^F]10a** and **[^18^F]10b** were determined with a HPLC method using an isocratic method (system 5, HPLC 55 iso).

Radiolabeling with fluorine-18 and iodine-123. The [^18^F]KF was produced via a ^18^O(p, n)^18^F reaction by bombardment of enriched [^18^O]water with 18–30 MeV protons using a TR-Flex-Zyklotron (Advanced Cyclotron Systems Inc., ACSI, Richmond, BC, Canada) in the Helmholtz-Center Dresden-Rossendorf [92].

The non-carrier added sodium [^123^I]iodide (Na [^123^I]I) was produced in-house using a TR-Flex cyclotron (Advanced Cyclotron Systems Inc., ACSI, Canada) and the gas target KIPROS 200 from ZAG Zyklotron AG (Eggenstein-Leopoldshafen, Germany) by bombardment of highly enriched ^124^Xe gas with 30 MeV protons via, amongst others, the nuclear reaction ^124^Xe(p, pn)^123^Xe → ^123^I. Concentration of crude [^123^I]iodide and formulation in 0.02 M aqueous sodium hydroxide was performed by ROTOP Pharmaka GmbH at the HZDR campus. Aliquots containing [^123^I]iodide in an activity concentration of ~20–50 MBq/µL were used for further experiments and diluted accordingly with NaOH (0.02 M).

Radio-HPLC was performed using HPLC systems 3, 4 and 5. Radio-TLC was performed as described above and visualized using a Fuji BAS 2000^®^ scanner system and analyzed using advanced image data analyzer (AIDA) software (Version 5.1 SP4, Raytest, Straubenhardt, Germany).

Confocal laser scanning microscopy was performed with the Olympus Fluoview^TM^ 1000 confocal laser scanning microscope (Olympus Fluoview 1000, Melville, NY, USA) using a 60× (numerical aperture (NA) 1.35) oil objective. The confocal images were performed using the standardized DAPI, Alexa Fluor 488 and Alexa Fluor 647 optical filters. The data were analyzed using the FV10-ASW software.

## 4. Conclusions

To summarize, novel radiolabeled NIR dyes belonging to the silicon-rhodamine family were synthesized and their optical properties were studied. The SiRs with far-red to NIR optical properties show comparable high quantum yields in aqueous environment. Furthermore, the high photostability of the synthesized fluorinated SiRs makes them promising for biological applications. Attempts for radiolabeling of boron functionalized SiRs using modern copper-mediated synthesis strategies with the relevant clinical radionuclides fluorine-18 (for PET) and iodine-123 (for SPECT) were intensively studied and the reaction conditions were optimized. Two of the three presented precursors were successfully radiofluorinated and efforts for radioiodination from one and the same precursor was proofed as well. The radiofluorinated SiRs **[^18^F]10a** and **[^18^F]10b** show very high molar activities between 42- and 70 GBq/µmol, whereas the radioiodinated SiR **13** shows a remarkable molar activity of 7.6 TBq/µmol after the end of synthesis. The radiofluorinated SiRs with low molecular weight (>500 g/mol) show high in vitro stabilities in saline and human plasma. However, the general issue of high lipophilicity of mainly all small molecular organic dyes combined with the relatively high lipophilicity of the radiolabeled SiRs **[^18^F]10a** ( $\log D_{\mathrm{pH}=7.4}$ = 2.92), **[^18^F]10b** ($\log D_{\mathrm{pH}=7.4}$ = 3.22) and **[^123^I]13** ( $\log D_{\mathrm{pH}=7.4}$ = 3.48) should be taken in account for further derivatization. Colocalization experiments with the cationic and lipophilic SiRs **10a**,**b** in cancer cells (PC3) also show selective enrichment in mitochondria, which in principle opens up the possibility of studies on imaging of the heart muscle (PET/SPECT). Another feature is the modular concept allowing to switch between fluorine-18 for PET or iodine-123 for SPECT by using the same precursor for radiolabeling. Applying this concept, a bioconjugateable SiR can be synthesized that match the pharmacokinetic properties of the conjugated vector (e.g., longer bio- logical half-life (radioiodination) if SiR is conjugated to an antibody, shorter half-life (radiofluorination) if SiR is ligated to a fast-enriching peptide or a small molecule). For these reasons, it is required that in addition to the boronic acids functioning as radiolabeling precursor moieties further function such as carboxylic acids or amines have to be introduced into the rhodamine scaffold for subsequent biological conjugation with feasible targeting vectors. All these properties make this class of novel radiolabeled dyes very promising for further biological evaluation. Such SiRs that are conjugated to tumor specific vectors can find application in sequential or simultaneous PET/SPECT-NIR imaging, enabling planning and execution of fluorescence-guided surgical resections of solid tumors.

## Figures and Tables

**Table 1:** Overview of the optical properties of the synthesized SiRs in dimethyl sulfoxide (DMSO) and phosphate-buffered saline (PBS; pH = 7.4) at room temperature. Excitation of the samples was performed at *λ*_exc_ = 600 nm.

Dye	Solvent	*λ_abs_* (nm)	$\varepsilon_{\max}$ [M^−1^ cm^−1^]	*λ_em_* (nm)	*Φ*_fl_ (a)
**6a**	DMSO	659	67.800	677	0.149
	PBS (pH = 7.4) (b)	646	61.700	662	0.104
**6b**	DMSO	659	106.600	678	0.094
	PBS (pH = 7.4) (b)	647	61.700	661	0.182
**6c**	HCl (aq, 0.1 M)	644	17.500	667	0.197
**8**	DMSO	660	57.900	677	0.162
	PBS (pH = 7.4) (b)	643	47.600	660	0.135
**10a**	DMSO	663	143.900	682	0.134
	PBS (pH = 7.4) (b)	649	88.700	665	0.115
**10b**	DMSO	666	113.400	682	0.124
	PBS (pH = 7.4) (b)	650	51.100	666	0.075
**10c**	DMSO	671	94.900	687	0.149
	PBS (pH = 7.4) (b)	655	54.200	671	0.221
**13**	DMSO	663	83.900	681	0.118
	PBS (pH = 7.4) (b)	648	41.100	667	0.135

(a) For the determination of the quantum yields Nile Blue A in ethanol was used as reference dye. (b) Measured in a co-injected solution containing 0.5% DMSO.

**Table 2:** Overview of the radiofluorination experiments performed with various boron-functionalized SiRs by varying precursors, elution techniques, copper agents, solvents, temperature, time and the amount of precursor relative to copper agent. The reactions were evaluated by calculating the radiochemical conversion (RCC) and the radiochemical yield (RCY).

Entry	Precursor	Elution Technique	[Cu]	Solvent	Temperature	Time	Equivalents Precursor: [Cu]	RCC	RCY
1	**6a**	DMAPH^+^OTf^−^ (a)	[Cu(OTf)_2_(py)_4_]	DMA	100 °C	20 min	1:4	3% (d)	-
2				DMA	110 °C	20 min	1:4	6% (d)	-
3				DMA	120 °C	20 min	1:4	25% (d)	14.0 ± 0.3% (*n* = 3)
4				DMF	120 °C	20 min	1:4	0% (d)	-
5				DMA	120 °C	20 min	4:1	0% (d)	-
6				DMA	120 °C	40 min	1:4	10% (d)	-
7				DMA	140 °C	20 min	1:4	10% (d)	-
8				DMA	140 °C	40 min	1:4	5% (d)	-
9				DMA	160 °C	20 min	1:4	0% (d)	-
10				DMA	160 °C	40 min	1:4	0% (d)	-
11	**6b**			DMA	120 °C	20 min	1:4	0% (d)	-
12	**6c**			DMA	120 °C	20 min	1:4	0% (d)	-
13	**6a**	*n*-BuOH/TEAHCO_3_ (b)		DMA	120 °C	20 min	1:4	16% (d)	15% (*n* = 1)
14				DMA	120 °C	20 min	1:1	12% (e)	-
15				DMA	120 °C	20 min	4:1	4% (e)	-
16	**6b**			DMA	120 °C	20 min	1:4	0% (d)	-
17	**6c**			DMA	120 °C	20 min	1:4	0% (d)	-
18	**6a**	KOTf/K_2_CO_3_ (c)		DMA	120 °C	20 min	1:4	35% (d)	25 ± 4% (*n* = 3)
19				DMI	120 °C	20 min	1:4	70% (d)	54 ± 1% (*n* = 2)
20	**6b**			DMI	120 °C	20 min	1:4	48% (d)	33% (*n* = 1)
21	**6c**			DMI	120 °C	20 min	1:4	0% (d)	-
22	**6a**		[Cu(OTf)_2_(impy)_4_]	DMA	120 °C	20 min	1:4	18% (d)	-
23	**6b**			DMA	120 °C	20 min	1:4	0% (d)	-
24	**6c**			DMA	120 °C	20 min	1:4	0% (d)	-
25	**8**		[Cu(OTf)_2_(py)_4_]	DMA	120 °C	20 min	1:4	0% (d)	-
26				DMI	120 °C	20 min	1:4	0% (d)	-

(a) Elution was performed with a solution of 4-(dimethylamino)-pyridinium triflate (DMAPH^+^OTf^−^; 6.8 mg in 500 µL DMA/DMF) over a weak anion exchange (WAX) cartridge. (b) Elution was performed with a solution of tetraethylammonium bicarbonate (TEAHCO_3_; 2.7 mg in 400 µL n-butanol) over a quaternary methyl ammonium (QMA) cartridge. (c) Elution was performed with a solution of potassium triflate (5 mg) and potassium carbonate (50 µg) in DI water (500 µL) and acetonitrile (1 mL) over a QMA cartridge followed by a azeotropic drying step. (d) The radiochemical conversion was calculated either via HPLC or (e) via radio-TLC. DMI: 1,3-dimethyl-2-imidazolidinone.

## Data Availability

All the acquired data are available in the article as well as in the Supplementary Information.

## Supplementary Materials

The following are available online at https://www.mdpi.com/article/10.3390/ph14111155/s1, full (radiochemical) synthesis procedures, characterization including NMR, HR-MS, UV-Vis-NIR, IR, HPLC and radiochemical data are available in the supporting information.
