# Supplementary material for: Radiolabeled Silicon-Rhodamines as Bimodal PET/SPECT-NIR Imaging Agents
Source: Pharmaceuticals (Basel). 2021 Nov 12;14(11):1155. doi: 10.3390/ph14111155 (PMC8623702; doi:10.3390/ph14111155)
Supplement: Supplementary file 1 [file pharmaceuticals-14-01155-s001.zip › pharmaceuticals-1410034-supplementary.pdf]

Radiolabeled silicon-rhodamines as bimodal PET/SPECT-NIR imaging agents

Thines Kanagasundaram[1,2,3], Markus Laube[1], Johanna Wodtke[1], Carsten Sven Kramer[3], Sven Stadlbauer[1], Jens Pietzsch[1, 4], Klaus Kopka[1,3,4]*

[1] Helmholtz-Zentrum Dresden-Rossendorf (HZDR) e.V., Institute of Radiopharmaceutical Cancer Research, Bautzner Landstrasse 400, 01328 Dresden, Germany; t.kanagasundaram@hzdr.de (T.K.); m.laube@hzdr.de (M.L.); j.wodtke@hzdr.de (J.W.), s.stadlbauer@hzdr.de (S.S.); j.pietzsch@hzdr.de (J.P.).

[2] Institute of Inorganic Chemistry, Heidelberg University, Im Neuenheimer Feld 270, 69120 Heidelberg, Germany.

[3] Radiopharmaceutical Chemistry, German Cancer Research Center (DKFZ), Im Neuenheimer Feld 223, 69120 Heidelberg, Germany; carsten_kramer@gmx.de (C.S.K.).

[4] Faculty of Chemistry and Food Chemistry, Technische Universität Dresden, Mommsenstrasse 4, 01062 Dresden, Germany.

* Correspondence: k.kopka@hzdr.de (K.K.)

Table of Contents

1. General remarks 4

2. Syntheses 8

2.1 General procedure: the synthesis of protected boronates 8

2.1.1 Synthesis of 2-(4′-Bromophenyl)-6-butyl[1,3,6,2]dioxazaborocan (**2a**) 8

2.1.2 Synthesis of 2-(3′-Bromophenyl)-6-butyl[1,3,6,2]dioxazaborocan (**2b**) 9

2.1.3 Synthesis of 2-(2′-Bromophenyl)-6-butyl[1,3,6,2]dioxazaborocan (**2c**) 10

2.2 Synthesis of 4,4‘-methylenebis(3-bromo-*N,N*-dimethylaniline) (**4**), ... 11

2.3 Synthesis of Si-xanthone **5**, ... 12

2.4 General procedure: syntheses of the boronic acid functionalized silicon-rhodamines 14

2.4.1 Synthesis of Si-rhodamine **6a** 14

2.4.2 Synthesis of Si-rhodamine **6b** 16

2.4.3 Synthesis of Si-rhodamine **6c** 17

2.5 The synthesis of a boronic acid pinacol ester functionalized Si-rhodamine **8** 18

2.6 General procedure: syntheses of the fluorinated silicon-rhodamines 19

2.6.1 Synthesis of Si-rhodamine **10a** 20

2.6.2 Synthesis of Si-rhodamine **10b** 21

2.6.3 Synthesis of Si-rhodamine **10c** 22

2.7 The synthesis of Si-rhodamine **12** 23

2.8 The synthesis of Si-rhodamine **13** 25

2.9 The synthesis of copper complex [$Cu(OTf)_2(impy)_4$] 27

2.10 Determination of the optical properties 27

2.11 Copper-mediated radiofluorination (CMRF) screening experiments 29

2.11.1 CMRF by using WAX cartridges 29

2.11.2 CMRF by using QMA cartridges 32

2.11.3 Determination of the radiochemical yield 34

2.11.4 Determination of the molar activity 39

2.11.5 Determination of the partition coefficient 43

2.11.6 Stability tests of **[^{18}F]10a** and **[^{18}F]10b** in saline and human serum 43

2.12 Copper-mediated radioiodination (CMRI) of SiR **6a** 44

3. Confocal laser scanning microscopy – mitochondrial colocalization experiments 48

4. Analytical Data 49

4.1 NMR Spectra 49

4.2 Optical properties of the silicon-rhodamines 69

4.2.1 UV-Vis-NIR-spectra of the silicon-rhodamines 69

4.2.2 Photostability experiments of silicon-rhodamines and Abberior® STAR635 79

4.3 IR-spectra 82

4.4 HRMS spectra 97

4.5 HPLC chromatograms 103

1. General remarks

Unless otherwise stated reactions requiring exclusion of oxygen and moisture were carried out in heat-gun dried flasks under argon gas or nitrogen atmosphere using the Schlenk-technique.

All **chemicals** and **solvents** were purchased from Sigma-Aldrich Laborchemikalien GmbH, abcr GmbH, Acros Organics and were used without further purification. Deuterated solvents were used from Deutero GmbH. The dry solvents dimethylformamide (DMF), dimethylacetamide (DMA), diethyl ether, methanol and tetrahydrofuran were purchased from Sigma-Aldrich Laborchemikalien GmbH in Sure/Seal™ bottles.

NMR spectra were recorded at room temperature on the following spectrometers: Bruker Avance III 400 MHz, Agilent DD2-400 MHz and Agilent DD2-600 MHz (ProbeOne NMR probe) for ^{1}H-NMR spectra and 101 MHz and 151 MHz for ^{13}C-NMR spectra, respectively. Chemical shifts are reported in δ units relative to chloroform-*d* (δ_H= 7.26 ppm; δ_C= 77.2 ppm) or methanol-d_4 (δ_H= 3.31 ppm; δ_C= 49.0 ppm).[1] Analyses followed first order and the following abbreviations were used throughout: s= singlet, d= doublet, t= triplet, dd= doublet of doublet etc., m= multiplet. Coupling constants (*J*) are given in Hz and refer to H, H-couplings. The ^{11}B-NMR experiments were carried out with Wilmad® quartz NMR tubes.

High Resolution Mass spectra (HR-MS) were obtained on a Q-TOF MS using electrospray ionization: Agilent 1260 Infinity II HPLC (Santa Clara, California, USA; pump G7111B, autosampler G7129A, column oven G7116N, UV detector G7717C, eluent MeCN/water acidified with 0.1% formic acid, bypass mode) coupled to UHD Accurate Mass Q-TOF LC MS G6538A. Furthermore, HR-MS data were determined by using electrospray ionization and the spectrometer BrukerApexQe hybrid 9.4 T FT-ICR. The molecule ions are reported as mass to charge (*m/z*) relation.

UV-Vis-NIR absorption spectra were measured on a spectrophotometer Specord 50 (Analytik Jena, Germany) from 300 to 800 nm in a quartz cuvette with 1 cm path length. All measurements were performed in dimethyl sulfoxide (DMSO) or phosphate-buffered saline solution (PBS) at room temperature. The fluorescence quantum yields were performed with relative measurements using a reference dye according to the literature.[2]

---

[1] G. R. Fulmer, A. J. M. Miller, N. H. Sherden, H. E. Gottlieb, A. Nudelman, B. M. Stoltz, J. E. Bercaw, K. I. Goldberg, *Organometallics* **2010**, *29*, 2176–2179.
[2] C. Würth, M. Grabolle, J. Pauli, M. Spieles, U. Resch-Genger, *Nat. Prot.* **2013**, *8*, 1535–1550.

Nile Blue A as a reference standard was used for the determination of the quantum yields.[3] Fluorescence properties were determined using the Perkin Elmer LS-55 fluorescence spectrometer from Perkin Elmer Inc. (Waltham, Massachusetts, USA) at room temperature. The excitation wavelength for the samples is account for 600 nm. The data acquired were analysed with OriginPro 2020 (64-bit) SR1.

Photostability experiments were investigated on a Perkin Elmer LS-55 fluorescence spectrometer by using the integrated laser and the irradiation of the samples (c~5 µM for dye **10a**, **10b** or **10c** and c~1 µM for the reference) in a quartz cuvette (1 cm path length) with a pulsed laser of the wavelength of 640 nm (20 kW, pulse width at half peak height < 10 µs) up to a maximum time of two hours. After several time points (10 min, 30 min, 1 h and 2 emission spectra were measured on the same device to determine the photostability of the corresponding dye.

Infrared spectra were conducted using the FT-IR-spectrometer Nicolet™ iS5 ATR from Thermo Scientific™ (Waltham, Massachusetts, USA). The samples were measured in ATR modus with a ZnSe crystal. The spectra were measured in the range from 500 cm^{-1} to 4000 cm^{-1} and the relevant type of signals were indicated as very strong (vs), s (strong), w (weak), and br (broad).

Analytical Thin Layer Chromatography (TLC) was carried out on polygram-TLC-plates produced by Machery-Nagel (40 x 80 mm, SIL G/UV254, 0.2 mm layer thickness). Detection was carried out using UV-light (254 nm or 366 nm).

Flash column chromatography was carried out on silica gel (0.032–0.062 mm, produced by Macherey-Nagel) using manual techniques. Organic mobile phase mixtures of different solvents (hexanes/ethyl acetate, dichloromethane/methanol and chloroform/methanol) were used for purification.

High-Performance Liquid Chromatography (HPLC) system was used for semi-preparative purification: Knauer Smartline system (Berlin, Germany) equipped with a Smartline pump 1000, the degasser system Smartline manager 5000 with performance at room temperature, UV-Vis-NIR Smartline detector 2500 for wavelength detection at 254 nm and 650 nm and the C_{18} column from Phenomenex (Gemini®, 5 µm, 110 Å, LC Column 250 x 4.6 mm).

[3] R. Sens, K. H. Drexhage, *Journal of Luminescence* **1981**, *24-5*, 709–712.

The purification was performed with the following systems: (*system* 1) using the method 10–90% MeCN/H_2O, linear gradient in 35 minutes, with constant 0.1% v/v trifluoroacetic acid (TFA) additive and a flow rate of 5.30 mL/min or using the method 30–90% MeCN/H_2O, linear gradient in 35 minutes, with constant 0.1% v/v trifluoroacetic acid (TFA) additive and a flow rate of 5.50 mL/min (*system* 2).

Radio-HPLC purification of **[^{18}F]10a** and **[^{18}F]10b** were performed by using a semi-preparative Shimadzu prominence LC20AR equipped with LC-20AR binary gradient module, SIL-10AR sample manager, SPD-M20A PDA detector and gamma-detector LB 500 Herm (Berthold Technologies, Germany). The purification was performed on a Nucleosil® 100-7 C_{18} column (7 µm, 100 Å, LC Column 250 x 16 mm). The data was processed with Labsolution Software V. 5.92. The purification was carried out using an isocratic method with water+0.1% trifluoroacetic acid/acetonitrile 45/55 and a flow rate of 5 mL in 50 minutes (*system* 3).

Further, the radio-HPLC purification of **[^{123}I]13** was carried out by using a semi-preparative Jasco LC-NetII/ADC interface equipped with a quaternary pump Jasco PU-2089 PLUS with a vacuum degasser, a Jasco UV-2075 detector (Jasco Corporation, Tokyo, Japan) and the gamma spectrometer GABI from Elysia-Raytest GmbH (Straubenhardt, Germany). The purification was performed on a Luna® C_{18} column (10 µm, 100 Å, LC Column 250 x 10 mm). The data was processed through Jasco ChromNAV Software. The purification was carried out using a linear gradient from 25–75% MeCN/0.1% trifluoroacetic acid (TFA) with H_2O/0.1% trifluoroacetic acid in 33 minutes and a flow rate of 4 mL/min (*system* 4).

(Ultra) High-Performance Liquid Chromatography (UHPLC/HPLC) system was used for analytical purposes: (*system* 5) Shimadzu Nexera X2 UHPLC system (Shimadzu Corporation, Kyoto, Japan) equipped with a dual pump LC-30AD, on-line degasser DGU-20A$_{3R}$ and DGU-20A$_{5R}$, column oven CTO-20AC with two column switching valves FCV-14AH and a performance at 40 °C, the fluorescence detector RF-20A, an autosampler SIL-30AC, the photodiode array detector (PDA) for detection at wavelengths at 254 nm and 650 nm (SPD-M20A), the communication bus module CBM-20A and the gamma spectrometer GABI from Elysia-Raytest GmbH (Straubenhardt, Germany; detection of fluorine-18: 100–600 keV; detection of iodine-123: 100–200 keV). For the HPLC the analytical C_{18} column Kinetex® from Phenomenex (5 µm, 100 Å, LC Column 250 x 4.6 mm) and for the UHPLC the analytical C_{18} column Kinetex® (1.7 µm, 100 Å, LC Column 50 x 2.1 mm) were used.

The analytical HPLC analysis was performed with the following systems: (*system* 5) using a linear gradient method with water+0.1% trifluoroacetic acid/acetonitrile at a flow rate of 1 mL/min (HPLC 25–75: $t_{0\,min}$25/75–$t_{3\,min}$25/75–$t_{28\,min}$75/25–$t_{29\,min}$95/5–$t_{34\,min}$95/5–$t_{35\,min}$25/75–$t_{40\,min}$25/75, total 40 min; HPLC 45–95: $t_{0\,min}$45/55–$t_{3\,min}$45/55–$t_{28\,min}$95/5–$t_{34\,min}$95/5–$t_{35\,min}$45/55–$t_{40\,min}$45/55, total: 40 min) or isocratic mode (HPLC 55 iso: $t_{0\,min}$45/55–$t_{15\,min}$45/55)

The analytical UHPLC analysis was performed to determine the radiochemical conversions (RCCs) with *system* 5 using a linear gradient method with acetonitrile/water+0.1% trifluoroacetic acid (UHPLC 25–75: $t_{0\ min}$25/75–$t_{0.3\ min}$25/75–$t_{4.0\ min}$ 75/25–$t_{4.5\ min}$ 95/5– $t_{5.5\ min}$ 95/5–$t_{6.0\ min}$ 25/75–$t_{7.5\ min}$25/75; total 7.5 min) at a flow rate of 0.5 mL/min.

The molar activities and the stabilities in saline/human serum of **[^{18}F]10a** and **[^{18}F]10b** were determined with a HPLC method using an isocratic method (*system* 5, HPLC 55 iso).

Melting points were carried out on a Boetius hot microscope table (VEB Wägetechnik Rapido Radebeul/VEB Kombinat NAGEMA, Germany).

Chemical formulas were drawn with ChemDraw Professional 19.1.1.21

NMR data were analyzed with MestReNova 10.0.1-14719.

Radiolabeling with fluorine-18 and iodine-123. The [^{18}F]KF was produced via a ^{18}O(p, n)^{18}F reaction by bombardment of enriched [^{18}O]water with 18–30 MeV protons using a TR-Flex-Zyklotron (Advanced Cyclotron Systems Inc., ACSI, Canada) in the Helmholtz-Center Dresden-Rossendorf.[4]

The non-carrier added sodium [^{123}I]iodide (Na[^{123}I]I) was produced in-house using a TR-Flex cyclotron (Advanced Cyclotron Systems Inc., ACSI, Canada) and the gas target KIPROS 200 from ZAG Zyklotron AG (Eggenstein-Leopoldshafen, Germany) by bombardment of highly enriched ^{124}Xe gas with 30 MeV protons via*,* amongst others, the nuclear reaction ^{124}Xe(p, pn)^{123}Xe→^{123}I. Concentration of crude [^{123}I]iodide and formulation in 0.02 M aqueous sodium hydroxide was performed by ROTOP Pharmaka GmbH at the HZDR campus. Aliquots containing [^{123}I]iodide in an activity concentration of ~20-50 MBq/µL were used for further experiments and diluted accordingly with NaOH (0.02 M).

Radio-HPLC was performed using HPLC *systems* 3, 4 and 5. Radio-TLC was performed as described above and visualized using a Fuji BAS 2000® scanner system and analyzed using advanced image data analyzer (AIDA) software (Version 5.1 SP4, Raytest, Straubenhardt, Germany).

Confocal laser scanning microscopy was performed with the Olympus Fluoview™ 1000 confocal laser scanning microscope (Olympus Fluoview 1000, Melville, NY, USA) using a 60x (numerical aperture (NA) 1.35) oil objective. The confocal images were performed using the

[4] M. Kreller, H. J. Pietzsch, M. Walther, H. Tietze, P. Kaever, T. Knieß, F. Füchtner, J. Steinbach, S. Preusche, *Instruments* **2019**, *3*, 1–7.

standardized DAPI, Alexa Fluor 488 and Alexa Fluor 647 optical filters. The data were analyzed using the FV10-ASW software.

2. Syntheses

2.1 General procedure: the synthesis of protected boronates[5]

Under an argon atmosphere the brominated boronic acid (2.00 g, 9.96 mmol, 1.0 eq.) was placed in a flame dried round-bottom flask. Anhydrous toluene (50 mL) was added to the white powder. The white slurry was warmed up to 60 °C. Afterwards *N*-butyldiethanolamine (1.77 g, 11.0 mmol, 1.1 eq.) was added via a syringe to the suspension. The resulting colorless solution was stirred at 60 °C for two hours. Subsequently the solution was allowed to cool to room temperature and the toluene was removed under reduced pressure. After complete removal of the solvent the colorless solid was washed with *n*-hexane and diethyl ether. The colorless powder was dried under high vacuum overnight.

2.1.1 Synthesis of 2-(4′-Bromophenyl)-6-butyl[1,3,6,2]dioxazaborocan (2a)

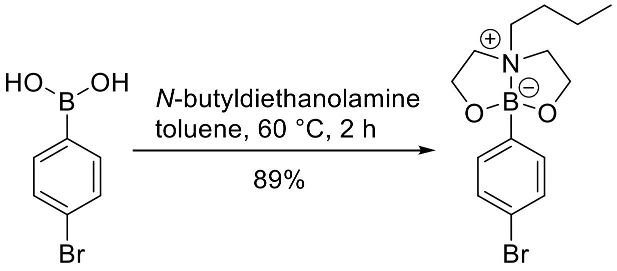

1a **2a**

2-(4′-Bromophenyl)-6-butyl[1,3,6,2]dioxazaborocan (**2a**) was synthesized from 4-bromophenyl boronic acid (**1a**) according to the general procedure 2.1. **2a** was obtained as colorless powder (2.89 g, 8.86 mmol, 89%).

^{1}H-NMR (400 MHz, $CDCl_3$, 300 K):

δ (ppm)= 7.48 (d, *J*= 8.3 Hz, 2 H, H_{arom}), 7.40 (d, *J*= 8.3 Hz, 2 H, H_{arom}), 4.21–4.04 (m, 4 H, CH_2), 3.12–2.97 (m, 4 H, CH_2), 2.32–2.22 (m, 2 H, CH_2), 1.54–1.46 (m, 2 H, CH_2), 1.14 (h, *J*= 7.3 Hz, 2 H, CH_2), 0.83 (t, *J*= 7.3 Hz, 3 H, CH_3).

^{13}C-NMR (101 MHz, $CDCl_3$, 300 K):

δ (ppm)= 135.3, 130.5, 122.3, 64.1, 59.2, 56.7, 28.0, 20.3, 14.5.

Note: the C_{arom}–B signal was not observed.

[5] K. Durka, M. Urban, M. Dąbrowski, P. Jankowski, T. Kliś, S. Luliński, *ACS Omega* **2019**, *4*, 2482−2492.

^{11}B-NMR (128 MHz, $CDCl_3$, 300 K):
δ (ppm)= 12.4.

IR (ATR, neat):
υ [cm^{-1}]= 3207 (br), 3036 (vw), 2961 (s), 2929 (s), 2875 (s), 1576 (s).

HR-ESI-MS:

$C_{14}H_{21}B^{79}Br^{81}BrN_2O_2$	Calculated *m/z*	found *m/z*
$[2M+Na]^+$	675.1570	675.1569

Melting point: *T*= 106 °C.

The chemical shifts of the ^{1}H-NMR and ^{13}C-NMR are in accordance with the literature.[5]

2.1.2 Synthesis of 2-(3′-Bromophenyl)-6-butyl[1,3,6,2]dioxazaborocan (2b)

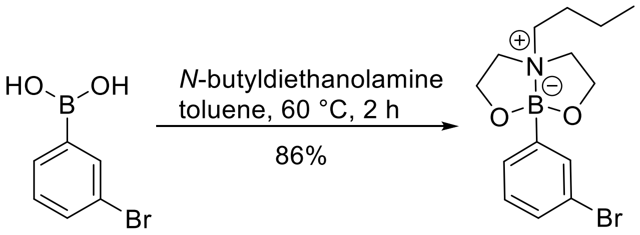

1b **2b**

2-(3′-Bromophenyl)-6-butyl[1,3,6,2]dioxazaborocan (**2b**) was synthesized from 3-bromophenyl boronic acid (**1b**) according to the general procedure 2.1. **2b** was obtained as colorless powder (2.79 g, 8.57 mmol, 86%).

^{1}H-NMR (400 MHz, $CDCl_3$, 300 K):
δ (ppm)= 7.80 (dd, *J*= 7.4 Hz, 2.0 Hz, 1 H, H_{arom}), 7.49 (d, *J*= 7.9 Hz, 1 H, H_{arom}), 7.23 (dd, *J*= 14.7 Hz, 7.3 Hz, 1 H, H_{arom}), 7.09 (td, *J*= 7.5 Hz, 1.9 Hz, 1 H, H_{arom}), 4.26–4.11 (m, 4 H, CH_2), 3.31 (dd, 4 H, *J*= 17.1 Hz, 10.3 Hz, 1 H, CH_2), 2.74–2.51 (m, 2 H, CH_2), 1.60–1.51 (m, 2 H, CH_2), 1.18 (h, *J*= 7.4 Hz, 2 H, CH_2), 0.85 (t, *J*= 7.3 Hz, 3 H, CH_3).

^{13}C-NMR (101 MHz, $CDCl_3$, 300 K):
δ (ppm)= 135.8, 132.4, 130.7, 129.3, 122.6, 63.2, 60.0, 55.7, 24.7, 20.3, 13.9.
Note: the C_{arom}–B signal was not observed.

^{11}B-NMR (128 MHz, $CDCl_3$, 300 K):
δ (ppm)= 12.4.

IR (ATR, neat):
υ [cm^{-1}]= 3047 (br), 2957 (s), 2928 (s), 2903 (s), 2873 (s), 2841 (s), 2698 (w), 1581 (vs), 1533 (vs).

Melting point: *T*= 116–117 °C.

HR-ESI-MS:

$C_{14}H_{21}B^{79}Br^{81}BrN_2O_2$	Calculated *m/z*	found *m/z*
$[2M+Na]^+$	675.1570	675.1566

The chemical shifts of the ^{1}H-NMR and the melting point are in accordance with the literature.[5]

2.1.3 Synthesis of 2-(2′-Bromophenyl)-6-butyl[1,3,6,2]dioxazaborocan (2c)

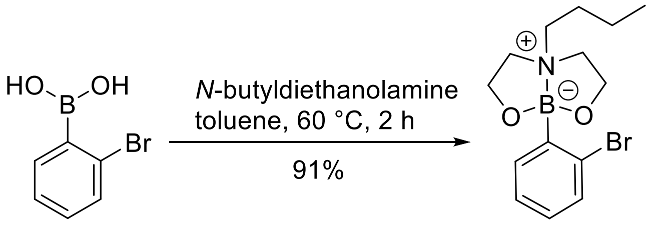

2-(2′-Bromophenyl)-6-butyl[1,3,6,2]dioxazaborocan (**2c**) was synthesized from 2-bromophenyl boronic acid (**1c**) according to the general procedure 2.1. **2c** was obtained as colorless powder (2.96 g, 9.06 mmol, 91%).

^{1}H-NMR (400 MHz, $CDCl_3$, 300 K):
δ (ppm)= 7.74 (s, 1 H, H_{arom}), 7.52 (d, *J*= 7.3 Hz, 1 H, H_{arom}), 7.37 (d, *J*= 8.7Hz, 1 H, H_{arom}), 7.15 (t, *J*= 7.6 Hz, 1 H, H_{arom}), 4.20–4.09 (m, 4 H, CH_2), 3.11–2.97 (m, 4 H), 2.32–2.28 (m, 2 H, CH_2), 1.60–1.51 (m, 2 H, CH_2), 1.18 (h, *J*= 7.4 Hz, 2 H, CH_2), 0.85 (t, *J*= 7.3 Hz, 3 H, CH_3).

^{13}C-NMR (101 MHz, $CDCl_3$, 300 K):
δ (ppm)= 137.0, 134.2, 130.7, 129.5, 126.4, 65.0, 58.4, 26.1, 18.9, 14.7.
Note: the C_{arom}–B signal was not observed.

^{11}B-NMR (128 MHz, $CDCl_3$, 300 K):
δ (ppm)= 12.5.

IR (ATR, neat):
υ [cm^{-1}]= 3054 (w), 2957 (s), 2932 (s), 2888 (s), 1547 (w).

Melting point: *T*= 94 °C.

HR-ESI-MS:

$C_{14}H_{21}B^{79}Br^{81}BrN_2O_2$	Calculated *m/z*	found *m/z*
$[2M+Na]^+$	675.1570	675.1568

The chemical shifts of the ^{1}H-NMR and the melting point are in accordance with the literature.[5]

2.2 Synthesis of 4,4'-methylenebis(3-bromo-*N,N*-dimethylaniline) (4)[6, 7]

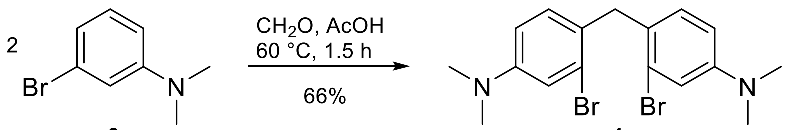

3-Bromo-*N*,*N*-dimethylaniline (**3**) (10.0 g, 50.0 mmol, 1.0 eq.) was dissolved in concentrated acetic acid (80 mL). Formaldehyde (37% in water, containing 10–15% methanol, 10.0 mL, 134 mmol, 2.7 eq.) was added to the colorless solution. The reaction mixture was heated to 60 °C and stirred for 1.5 hours. After complete conversion (monitored by TLC), the solution was allowed to cool down. The excess of acetic acid was evaporated under reduced pressure. A saturated solution of sodium bicarbonate (20 mL) was added carefully to the reaction mixture. The suspension was extracted with ethyl acetate (4 x 50 mL). The combined organic phases were washed with a saturated solution of sodium chloride (brine). Afterwards the organic phases were dried over sodium sulfate and filtered off. The solvent was removed by evaporation under reduced pressure. The crude product was purified by flash column chromatography (silica gel, *n*-hexane/ethyl acetate 95:5) to afford pale 4,4'-methylenebis(3-bromo-*N*,*N*-dimethylaniline) (**4**) (6.81 g, 16.5 mmol, 66%) as a colorless solid.

^{1}H-NMR (400 MHz, $CDCl_3$, 300 K):
δ (ppm)= 6.97 (s, 2 H, H_{arom}), 6.86 (d, *J*= 8.5 Hz, 2 H, H_{arom}), 6.62 (d, *J*= 6.8 Hz, 2 H, H_{arom}), 4.01 (s, 2 H, CH_2), 2.92 (s, 12 H, CH_3).

---

[6] G. Lukinavičius, K. Umezawa, N. Olivier, A. Honigmann, G. Yang, T. Plass, V. Mueller, L. Reymond, I. R. Corrêa, Z. G. Luo, C. Schultz, E. A. Lemke, P. Heppenstall, C. Eggeling, S. Manley, K. Johnsson, *Nat. Chem.* **2013**, *5*, 132–139.
[7] J. H. Gorvin, *J. Chem. Soc.* **1953**, 1237–1241.

^{13}**C-NMR** (101 MHz, $CDCl_3$, 300 K):

δ (ppm)= 150.1, 130.9, 127.2, 125.7, 116.4, 112.0, 40.7, 40.0.

IR (ATR, neat):

υ [cm^{-1}]= 2882 (br), 2805 (br), 1607 (vs), 1544 (s), 1504 (vs).

***R**f* value (*n*-hexane/ethyl acetate 95:5)= 0.30.

Melting point: *T*= 101–103 °C

HR-ESI-MS:

$C_{17}H_{20}{}^{79}Br^{81}BrN_2$	Calculated *m/z*	found *m/z*
$[M+H]^+$	413.0046	413.0050

The analytical data are in accordance with the literature.[6, 7]

2.3 Synthesis of Si-xanthone 5[8, 9]

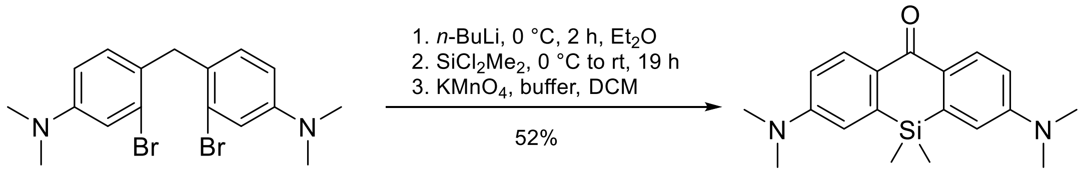

4 **5**

Under an argon atmosphere **4** (1.50 g, 3.64 mmol, 1.0 eq.) was dissolved in dry diethyl ether (80 mL) in a heat-dried round-bottom flask. The colorless solution was cooled down to 0 °C and *n*-BuLi (4.00 mL, 2.5 M in *n*-hexane, 10.2 mmol, 2.8 eq.) was added dropwise. The bright yellow solution was stirred for two hours at 0 °C. Subsequently dichlorodimethylsilane (470 mg, 3.64 mmol, 1.0 eq.) was added dropwise via a syringe to the reaction mixture at 0 °C. A colorless precipitate was observed within few minutes. The solution was allowed to warm up to room temperature and was stirred for further 19 h. After complete conversion (monitored by TLC), the pale yellow mixture was quenched with water (50 mL) and extracted with diethyl ether (3 x 150 mL). The combined organic phases were washed with a saturated solution of sodium chloride (brine) and were dried over sodium sulfate. After filtration the solvent was removed under reduced pressure. The subsequent benzylic oxidation was conducted using a procedure published by Bertozzi et al.[9] The residual brown solid was

---

[8] T. Kanagasundaram, C. S. Kramer, E. Boros, K. Kopka, *Dalton Trans.* **2020**, *49*, 7294–7298.

[9] C. Bertozzi, P. Shieh, (2015). Alkyne-activated fluorogenic azide compounds and methods of use thereof. US9410958B2.

dissolved in DCM (40 mL) and cooled down to 0 °C. Then a mixture of potassium permanganate (1.73 g, 10.9 mmol, 3.0 eq.) in water (30.0 mL), 1 M potassium hydroxide solution (7.50 mL) and tetrabutylammonium bisulfate (510 mg, 1.50 mmol, 0.4 eq.) was freshly prepared in a separate flask. The dark violet solution was added carefully to the blue DCM solution at 0 °C. The reaction mixture was stirred for 30 minutes at room temperature. The solution was quenched by addition of acetic acid (7.50 mL). Then sodium sulfite (2.85 g, 22.7 mmol, 6.0 eq.) was added in one portion. The color of the solution changed from brown to dark green immediately. The reaction mixture was diluted with water and the organic phase was separated. The aqueous phase was extracted with ethyl acetate (2 x 100 mL) and with DCM (3 x 100 mL) afterwards. The combined organic phases were washed with brine and dried over sodium sulfate. After filtration the solvent was removed under reduced pressure. The crude product was purified by flash column chromatography (silica gel, *n*-hexane/ethyl acetate 95:5 to 60:40) to afford Si-xanthone **5** (614 mg, 1.89 mmol, 52%) as a yellow solid.

^{1}H-NMR (400 MHz, $CDCl_3$, 300 K):
δ (ppm)= 8.40 (d, *J*= 9.0 Hz, 2 H, H_{arom}), 6.85 (d, *J*= 2.1 Hz, 2 H, H_{arom}), 6.80 (dd, *J*= 8.7 Hz, 2.6 Hz, 2 H, H_{arom}), 3.10 (s, 12 H, N-CH_3), 0.47 (s, 6 H, Si-CH_3).

^{13}C-NMR (101 MHz, $CDCl_3$, 300 K):
δ (ppm)= 185.4, 151.5, 140.6, 131.8, 129.9, 114.4, 113.3, 40.2, –0.9.

IR (ATR, neat):
υ [cm^{-1}]= 2949 (br, s), 2808 (br), 2635 (w), 1682 (s), 1573 (vs), 1505 (s).

R_f value (*n*-hexane/ethyl acetate 8:2)= 0.18.

Melting point: *T*= 221–223 °C

HR-ESI-MS:

$C_{19}H_{24}N_2OSi$	Calculated *m/z*	found *m/z*
$[2M+Na]^+$	671.3208	671.3218

The analytical data are in accordance with the literature.[10]

[10] J. L. Bachman, P. R. Escamilla, A. J. Boley, C. I. Pavlich, E. V. Anslyn, *Org. Lett.* **2019**, *21*, 206–209.

2.4 General procedure: syntheses of the boronic acid functionalized silicon-rhodamines

Under an argon atmosphere the brominated phenyl boronates **2a–2c** (300 mg, 920 µmol, 4.0 eq.) were dissolved in anhydrous tetrahydrofuran (9.20 mL) in a flame-dried round-bottom flask. The brown solution was cooled down to –78 °C and was stirred for 10 minutes at this temperature. At –78 °C *tert*-butyllithium (1.6 M in pentane, 1.18 mL, 1.89 mmol, 8.2 eq.) was added dropwise to the solution. *CAUTION*: *solutions* of *tert-butyllithium react explosively* with *water and may ignite in moist air*. The orange solution was stirred for 30 minutes at –78 °C. Subsequently the Si-xanthone **5** (74.6 mg, 230 µmol, 1.0 eq.) dissolved in dry tetrahydrofuran (8 mL) was added via a syringe to the reaction mixture at –78 °C. The color of the solution turned to a bright orange. After complete addition of **5**, the cooling bath was immediately removed and the orange solution was stirred for at least four hours which led to almost complete conversion of **5** (monitored by TLC; DCM:MeOH 9:1). However complete consumption of **5** was not observed (monitored by TLC).

Then hydrochloric acid (3 M, 5.00 mL) and deionized water (50 mL) were added to the orange solution and the solution turned gradually dark blue. The blue solution was stirred for further 30 minutes. The dark solution was extracted with DCM (3 x 250 mL) until the aqueous solution became nearly colorless. The combined organic phases were washed with brine and were dried over sodium sulfate. After filtration the solvent was removed under reduced pressure. Afterwards the crude blue product was purified by flash column chromatography (silica gel, DCM:MeOH 99:1 to 80:20 with constant 2% v/v acetic acid additive) to afford the Si-rhodamines as a dark blue solid. The concentrated and combined fractions from the column were filtrated over cotton wool. For high purity and to verify the counter ion as a trifluoroacetate ion (presence of trifluoroacetate was proofed by ^{19}F-NMR), the product was purified by reverse phase HPLC (10–90% MeCN/H_2O, linear gradient in 35 minutes, with constant 0.1% v/v trifluoroacetic acid (TFA) additive, *system* 1) to afford the corresponding Si-rhodamines.

2.4.1 Synthesis of Si-rhodamine 6a

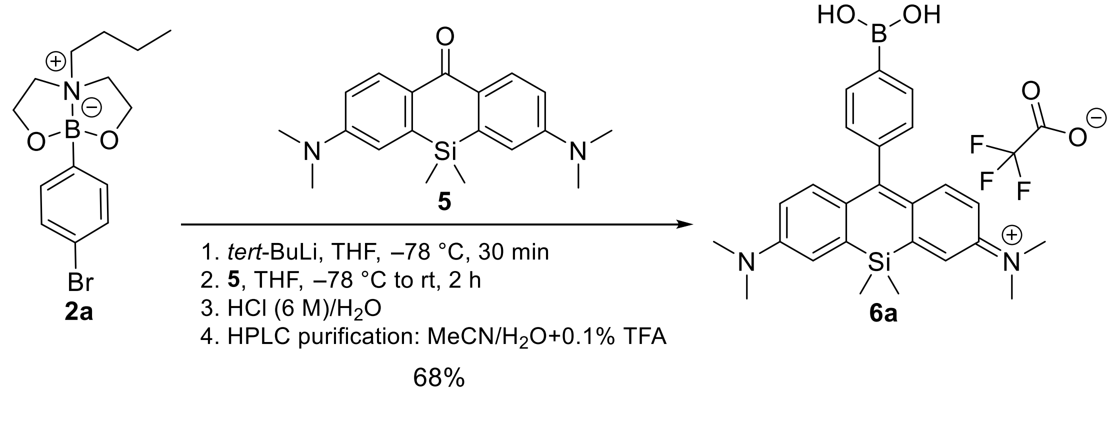

The Si-rhodamine **6a** was synthesized from 2-(4′-Bromophenyl)-6-butyl[1,3,6,2]dioxazaborocan (**2a**, 300 mg, 920 µmol, 4.0 eq.) and Si-xanthone **5** (74.6 mg, 230 µmol, 1.0 eq.) according to the general procedure 2.3 and **6a** was obtained as dark blue solid (84.8 mg, 156 µmol, 68%).

^{1}H-NMR (400 MHz, MeOD-*d_4*, 300 K):
δ (ppm)= 7.91 (s, 1 H, H_{arom}), 7.83 (s, 1 H, H_{arom}), 7.35 (d, *J*= 2.9 Hz, 2 H, H_{arom}), 7.26 (s, 2 H, H_{arom}), 7.14 (d, *J*= 9.7 Hz, 2 H, H_{arom}), 6.76 (d, *J*= 8.3 Hz, 2 H, H_{arom}), 3.34 (s, 12 H, N-CH_3), 0.60 (s, 6 H, Si-CH_3).

^{13}C-NMR (101 MHz, MeOD-*d_4*, 300 K):
δ (ppm)= 163.1, 155.7, 152.1, 149.6, 143.2, 134.5, 129.6, 128.8, 122.1, 114.9, 40.9, –1.1.
Note: the C_{arom}–B signal was not observed.

^{11}B-NMR (192 MHz, MeOD-*d_4*, 300 K):
δ (ppm)= 28.5.

^{19}F-NMR (376 MHz, MeOD-*d_4*, 300 K):
δ (ppm)= –77.4 (CF_3COO^-).

IR (ATR, neat):
υ [cm^{-1}]= 3227 (br), 2933 (br), 1673 (s), 1603 (s), 1573 (vs).

R_f value (dichloromethane/methanol 9:1)= 0.08.

Retention time (*system* 5, HPLC 45–95)= 6.63 min.

HR-ESI-MS:

$C_{25}H_{30}BN_2O_2Si^+$	Calculated *m/z*	found *m/z*
$[M]^+$	429.2165	429.2175

2.4.2 Synthesis of Si-rhodamine 6b

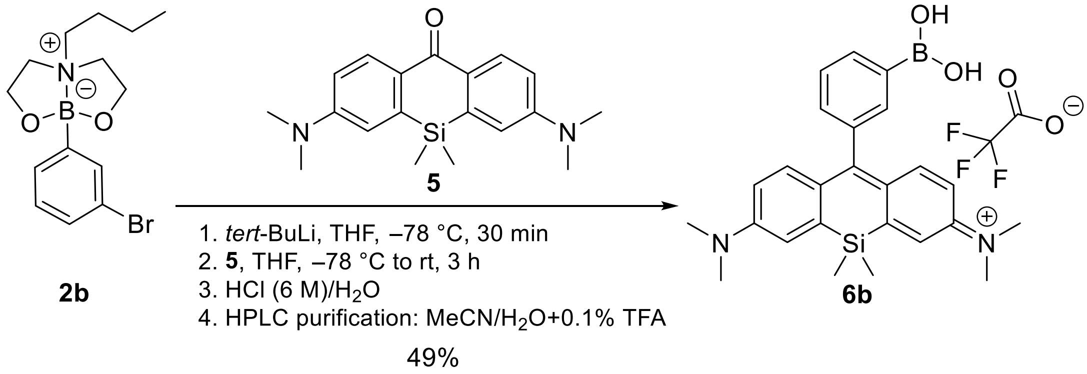

The Si-rhodamine **6b** was synthesized from 2-(3′-Bromophenyl)-6-butyl[1,3,6,2]dioxazaborocan (**2b**, 300 mg, 920 µmol, 4.0 eq.) and Si-xanthone **5** (74.6 mg, 230 µmol, 1.0 eq.) according to the general procedure 2.3 and **6b** was obtained as dark blue solid (61.1 mg, 113 µmol, 49%).

^{1}H-NMR (400 MHz, MeOD-d_4, 300 K):

δ (ppm)= 7.87 (s, 1 H, H_{arom}), 7.61–7.43 (m, 2 H, H_{arom}), 7.41–7.26 (m, 3 H, H_{arom}), 7.15 (d, *J*= 9.5 Hz, 2 H, H_{arom}), 6.76 (d, *J*= 9.8 Hz, 2 H, H_{arom}), 3.34 (s, 12 H, N-CH_3), 0.58 (s, 6 H, Si-CH_3).

^{13}C-NMR (151 MHz, MeOD-d_4, 300 K):

δ (ppm)= 171.5, 155.7, 149.6, 143.3, 139.9, 135.2, 134.8, 131.5, 129.0, 128.5, 122.1, 114.9, 40.8, –2.6.

Note: the C_{arom}–B signal was not observed.

^{11}B-NMR (192 MHz, MeOD-d_4, 300 K):

δ (ppm)= 28.1.

^{19}F-NMR (376 MHz, MeOD-d_4, 300 K):

δ (ppm)= –76.7 (CF_3COO^-).

IR (ATR, neat):

υ [cm^{-1}]= 3226 (br), 2932 (br), 1674 (s), 1573 (vs).

R_f value (dichloromethane/methanol 9:1)= 0.12.

Retention time (*system* 5, HPLC 45–95) = 7.24 min.

HR-ESI-MS:

$C_{25}H_{30}BN_2O_2Si^+$	Calculated *m/z*	found *m/z*
$[M]^+$	429.2165	429.2164
$[M+OMe]^+$	443.2321	443.2321
$[M+2OMe]^+$	457.2477	457.2477

2.4.3 Synthesis of Si-rhodamine 6c

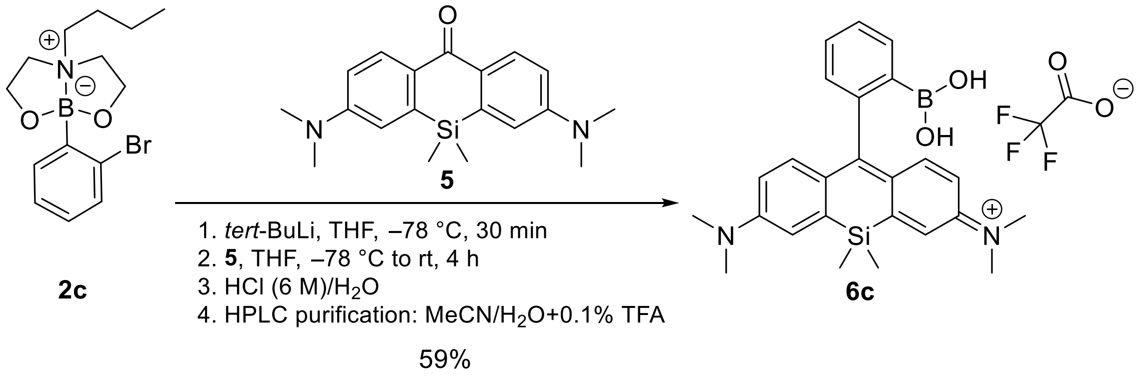

The Si-rhodamine **6c** was synthesized from 2-(2′-Bromophenyl)-6-butyl[1,3,6,2]dioxazaborocan (**2c**, 300 mg, 920 µmol, 4.0 eq.) and Si-xanthone **5** (74.6 mg, 230 µmol, 1.0 eq.) according to the general procedure 2.3 and **6c** was obtained as dark blue solid (73.6 mg, 136 µmol, 59%).

^{1}H-NMR (400 MHz, MeOD-*d₄*, 300 K):
δ (ppm)= 7.72–7.61 (m, 1 H, H_{arom}), 7.59–7.53 (m, 2 H, H_{arom}), 7.33 (d, *J*= 2.9 Hz, 2 H, H_{arom}), 7.29–7.22 (m, 1 H, H_{arom}), 7.20–7.05 (m, 2 H, H_{arom}), 6.88–6.62 (m, 2 H, H_{arom}), 3.33 (s, 12 H, N-CH_3), 0.64 (s, 3 H, Si-CH_3), 0.54 (s, 3 H, Si-CH_3).

^{13}C-NMR (101 MHz, MeOD-d_4, 300 K):
δ (ppm)= 171.8, 155.5, 149.4, 144.3, 143.6, 143.3, 134.5, 133.3, 132.1, 130.7, 130.3, 129.8, 129.5, 129.4, 128.8, 122.6, 121.5, 119.1, 115.3, 40.8, –0.2, –2.1.
Note: the C_{arom}–B signal was not observed.

^{11}B-NMR (192 MHz, MeOD-d_4, 300 K):
δ (ppm)= 26.5.

^{19}F-NMR (564 MHz, MeOD-d_4, 300 K):
δ (ppm)= –77.1 (CF_3COO^-).

IR (ATR, neat):
υ [cm^{-1}]= 3076 (br), 2929 (br), 1673 (s), 1572 (vs).

***R**$_f$* value (dichloromethane/methanol 96:4)= 0.47.

Retention time (*system* 5, HPLC 45–95)= 3.36 min.

HR-ESI-MS:

$C_{25}H_{30}BN_2O_2Si^+$	Calculated *m/z*	found *m/z*
$[M]^+$	429.2165	429.2173

2.5 The synthesis of a boronic acid pinacol ester functionalized Si-rhodamine 8

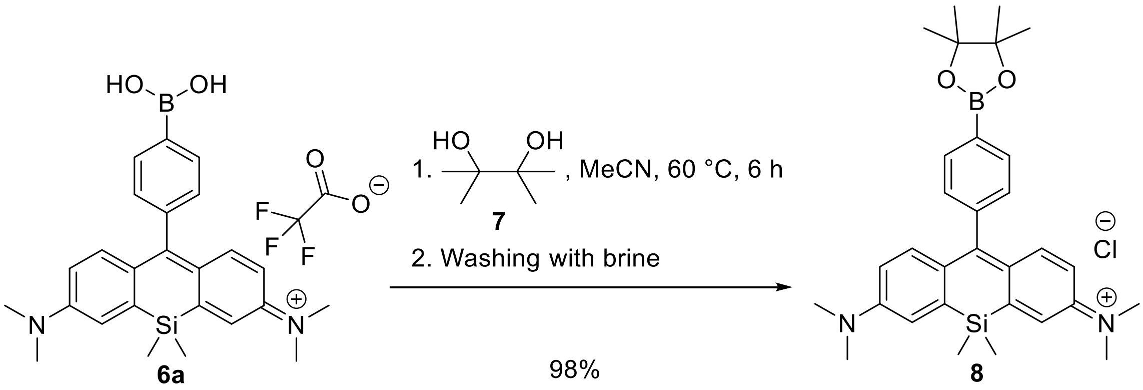

Under ambient conditions Si-rhodamine **6a** (5 mg, 9.22 µmol, 1 eq.) was dissolved in dry acetonitrile (200 µL) and the commercially available pinacol (1.14 mg, 9.68 µmol, 1.05 eq.) dissolved in acetonitrile (100 µL) was added to the blue solution. The reaction mixture was stirred for six hours at 60 °C. After complete consumption of the starting material (monitored by TLC: DCM:MeOH 9:1 and ESI-MS in positive mode) the solvent was removed under reduced pressure. The dark blue residue was dissolved in DCM (5 mL) and was transferred into a separation funnel. Then DCM (50 mL) and deionized water (50 mL) were added. Subsequently the dark solution was extracted with DCM (3 x 20 mL) until the aqueous solution became nearly colorless. The combined organic phases were washed with brine and were dried over sodium sulfate. After filtration, the solvent was removed under reduced pressure. After purification by microscale flash column chromatography (silica gel, DCM:MeOH 99:1 to 92:8) using a Pasteur pipette, the pinacol ester **8** was obtained as a dark blue solid (5.64 mg, 9.04 µmol, 98%). *Note: HPLC purification with solvents containing TFA or formic acid was avoided due to the instability of **8** in acidic media caused by protodeborylation. There was no*

^{19}F-NMR signal observed for the corresponding trifluoroacetate counter ion which indicates the presence of the chloride counter ion.

^{1}H-NMR (400 MHz, $CDCl_3$, 300 K):
δ (ppm)= 7.94 (d, *J*= 6.7 Hz, 2 H, H_{arom}), 7.26–7.17 (m, 4 H, H_{arom}), 7.09 (d, *J*= 11.0 Hz, 2 H, H_{arom}), 6.58 (dd, *J*= 9.6 Hz, 2.9 Hz, 2 H, H_{arom}), 3.39 (s, 12 H, N-CH_3), 1.39 (s, 12 H, CH_3), 0.61 (s, 6 H, Si-CH_3).

^{13}C-NMR (101 MHz, $CDCl_3$, 300 K):
δ (ppm)= 169.6, 154.2, 148.7, 142.5, 141.9, 134.5, 128.6, 128.0, 121.2, 113.8, 84.4, 41.3, 25.1, –0.5.
Note: the C_{arom}–B signal was not observed.

^{11}B-NMR (128 MHz, $CDCl_3$, 300 K):
δ (ppm)= 31.7.

IR (ATR, neat):
υ [cm^{-1}]= 3226 (br), 2925 (br), 1605 (s), 1574 (vs), 1512 (s).

R_f value (dichloromethane/methanol 9:1)= 0.52.

HR-ESI-MS:

$C_{31}H_{40}BN_2O_2Si^+$	Calculated *m/z*	found *m/z*
$[M]^+$	511.2947	511.2945

2.6 General procedure: syntheses of the fluorinated silicon-rhodamines

Under an argon atmosphere the halogenated benzenes **10a-10c** (100 mg, 571 µmol, 2.0 eq.) were dissolved in anhydrous tetrahydrofuran (5.70 mL) in a heat-dried round-bottom flask. The brown solution was cooled down to –78 °C and was stirred for 10 minutes at this temperature. At –78 °C *tert*-butyllithium (1.6 M in pentane, 714 µL, 1.14 mmol, 4.0 eq.) was added dropwise to the solution. *CAUTION*: *solutions* of *tert-butyllithium react explosively* with *water and may ignite in moist air*. The orange solution was stirred for 30 minutes at –78 °C. Subsequently the Si-xanthone **5** (92.6 mg, 286 µmol, 1.0 eq.) dissolved in dry tetrahydrofuran (10 mL) was added via a syringe to the reaction mixture at –78 °C. The color of the solution turned to a bright red. After complete addition of **5**, the cooling bath was removed and the reddish solution was stirred for two hours. Monitored by TLC (DCM:MeOH 9:1), complete

conversion of **5** was not observed. Then hydrochloric acid (1 N, 2.00 mL) and deionized water (50 mL) were added to the now orange solution and the solution turned gradually dark blue. The dark solution was extracted with DCM (3 x 100 mL) until the aqueous solution became nearly colorless. The combined organic phases were washed with brine and were dried over sodium sulfate. After filtration the solvent was removed under reduced pressure. Then the blue crude product was purified by flash column chromatography (silica gel, DCM:MeOH 99:1 to 92:8) to afford the Si-rhodamines as a dark blue solid. The possibility to reisolate **5** was given with isocratic elution on column (DCM:MeOH 99:1). For high purity and to verify the counter ion as a trifluoroacetate ion (presence of trifluoroacetate was proofed by ^{19}F-NMR), the product was purified by reverse phase HPLC (30–90% MeCN/H_2O, linear gradient in 35 minutes, with constant 0.1% v/v trifluoroacetic acid (TFA) additive, *system* 2) to obtain the corresponding Si-rhodamines. The reported yields are based on reisolated starting material of Si-xanthone **5** (brsm).

2.6.1 Synthesis of Si-rhodamine 10a

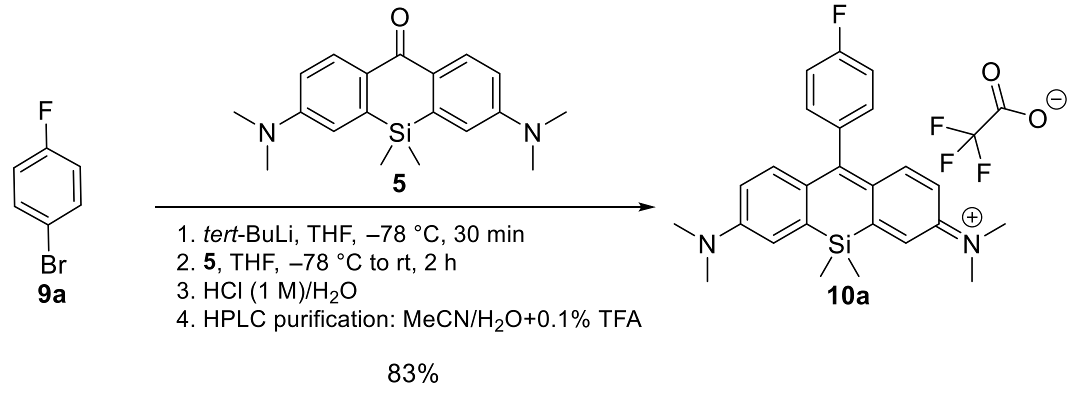

The Si-rhodamine **10a** was synthesized from commercially available 4-bromofluorobenzene (**9a**, 100 mg, 571 µmol, 2.0 eq.) and Si-xanthone **5** (92.6 mg, 286 µmol, 1.0 eq.) according to the general procedure 2.5 and **10a** was obtained as a dark blue solid (123 mg, 237 µmol, 83%).

^{1}H-NMR (400 MHz, MeOD-d_4, 300 K):
δ (ppm)= 7.40–7.36 (m, 2 H, H_{arom}), 7.34–7.27 (m, 4 H, H_{arom}), 7.15 (d, J= 9.7 Hz, 2 H, H_{arom}), 6.80 (dd, J= 9.7 Hz, 2.9 Hz, 2 H, H_{arom}), 3.35 (s, 12 H, N-CH_3), 0.61 (s, 6 H, Si-CH_3).

^{13}C-NMR (101 MHz, MeOD-d_4, 300 K):
δ (ppm)= 169.6, 164.2 (d, ^1J= 247.5 Hz), 155.6, 149.6, 143.0, 136.6 (d, ^4J= 3.7 Hz), 132.6, (d, ^3J= 8.3 Hz), 129.0, 122.3, 116.3 (d, ^2J= 22 Hz), 115.0, 40.9, –1.1.

^{19}F-NMR (376 MHz, MeOD-d_4, 300 K):
δ (ppm)= –78.0 (CF_3COO^-), –115.5.

IR (ATR, neat):
υ [cm^{-1}]= 2924 (br), 2811 (br), 1573 (vs).

R$_f$ value (dichloromethane/methanol 9:1)= 0.31.

Retention time (*system* 5, HPLC 45–95)= 12.34 min.

HR-ESI-MS:

$C_{25}H_{28}FN_2Si^+$	Calculated *m/z*	found *m/z*
$[M]^+$	403.2001	403.2011

2.6.2 Synthesis of Si-rhodamine 10b

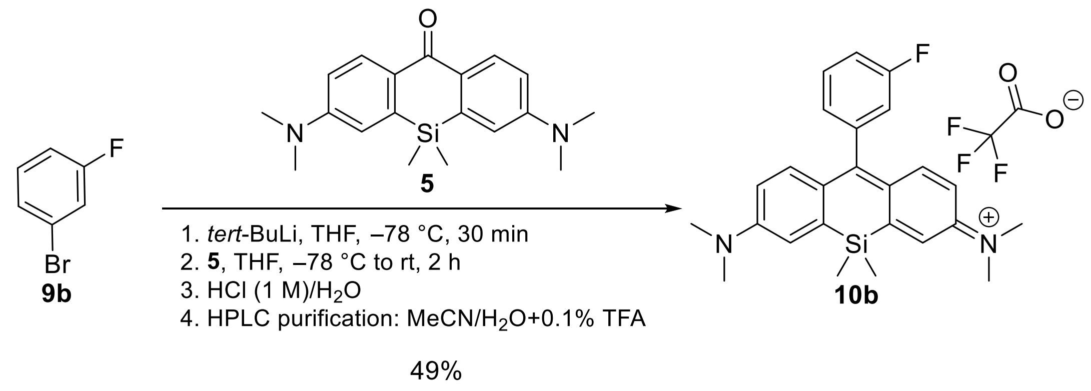

The Si-rhodamine **10b** was synthesized from commercially available 3-bromofluorobenzene (**9b**, 100 mg, 571 µmol, 2.0 eq.) and Si-xanthone **5** (92.6 mg, 286 µmol, 1.0 eq.) according to the general procedure 2.5 and **10b** was obtained as a dark blue solid (72.4 mg, 140 µmol, 49%).

^{1}H-NMR (400 MHz, MeOD-d_4, 300 K):
δ (ppm)= 7.59 (td, J= 8.2 Hz, 5.7 Hz, 1 H, H_{arom}), 7.36 (s, 2 H, H_{arom}), 7.33 (td, J= 8.3 Hz, 2.6 Hz, 1 H, H_{arom}), 7.20–7.07 (m, 4 H, H_{arom}), 6.80 (dd, J= 9.7 Hz, 2.9 Hz, 2 H, H_{arom}), 3.35 (s, 12 H, N-CH_3), 0.60 (s, 6 H, Si-CH_3).

^{13}C-NMR (101 MHz, MeOD-d_4, 300 K):

δ (ppm)= 172.4, 168.7, 163.5 (d, J= 148.8 Hz), 155.7, 149.6 (d, J= 5.0 Hz), 143.7, 143.0, 142.1, 132.3 (d, J= 8.4 Hz), 130.8 (d, J= 30.1 Hz), 128.6, 127.3, 125.8, 123.1 (d, J= 4.6 Hz), 121.6 (d, J= 4.6 Hz), 118.3, 117.3, 115.9, 114.2, 41.6, –0.5, –1.7.

^{19}F-NMR (376 MHz, MeOD-d_4, 300 K):

δ (ppm)= –77.4 (CF_3COO^-), –114.5.

IR (ATR, neat):

υ [cm^{-1}]= 2933 (br), 2815 (br), 1780 (s), 1737 (vs), 1605 (s), 1572 (vs).

***R**$_f$* value (dichloromethane/methanol 9:1)= 0.26.

Retention time (*system* 5, HPLC 25–75)= 22.21 min.

HR-ESI-MS:

$C_{25}H_{28}FN_2Si^+$	Calculated *m/z*	found *m/z*
$[M]^+$	403.2001	403.2001

2.6.3 Synthesis of Si-rhodamine 10c

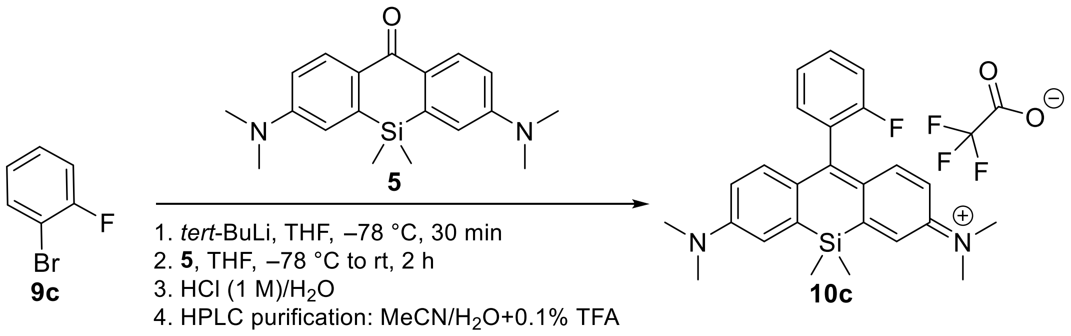

56%

The Si-rhodamine **10c** was synthesized from commercially available 2-bromofluorobenzene (**9c**, 100 mg, 571 µmol, 2.0 eq.) and Si-xanthone **5** (92.6 mg, 286 µmol, 1.0 eq.) according to the general procedure 2.5 and **10c** was obtained as dark blue solid (82.7 mg, 160 µmol, 56%).

^{1}H-NMR (400 MHz, $CDCl_3$, 300 K):

δ (ppm)= 7.55 (q, *J*= 8.1 Hz, 7.7 Hz, 1 H, H_{arom}), 7.34 (t, *J*= 7.5 Hz, 1 H, H_{arom}), 7.25–7.10 (m, 6 H, H_{arom}), 6.64 (d, *J*= 9.6 Hz, 2 H, H_{arom}), 3.35 (s, 12 H, N-CH_3), 0.59 (s, 6 H, Si-CH_3).

^{13}C-NMR (101 MHz, $CDCl_3$, 300 K):

δ (ppm)= 163.4, 160.8, 160.7, 158.1, 154.3, 148.6, 142.4, 140.8, 132.2 (d, *J*= 8.4 Hz), 130.5, 127.9 (d, *J*= 7.8 Hz), 126.3 (d, *J*= 21.7 Hz), 125.2 (d, *J*= 6.9 Hz), 123.6, 120.3, 118.0, 116.9, 115.1, 113.4, 41.8, –0.2, –1.5.

^{19}F-NMR (376 MHz, $CDCl_3$, 300 K):

δ (ppm)= –75.5 (CF_3COO^-), –113.9.

IR (ATR, neat):

υ [cm^{-1}]= 3372 (br), 2925 (br), 1572 (vs).

R_f value (dichloromethane/methanol 9:1)= 0.14.

Retention time (*system* 5, HPLC 45–95)= 4.56 min.

HR-ESI-MS:

$C_{25}H_{28}FN_2Si^+$	Calculated *m/z*	found *m/z*
$[M]^+$	403.2001	403.2007

2.7 The synthesis of Si-rhodamine 12

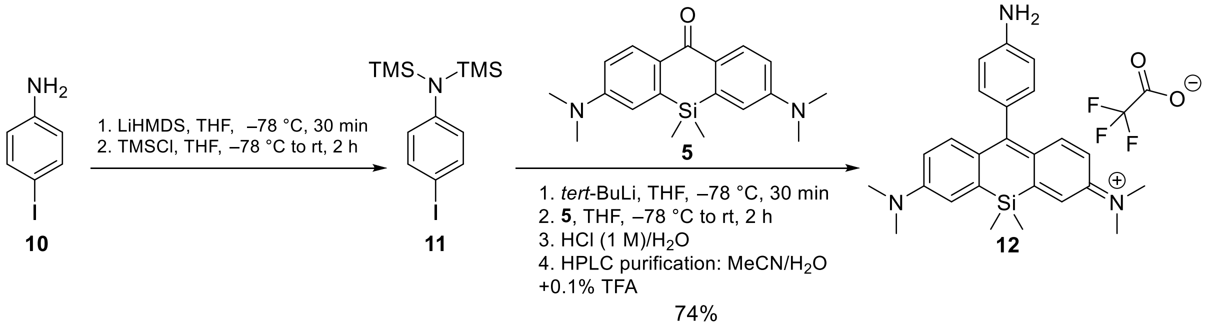

Under an argon atmosphere, 4-iodoaniline (**10**, 290 mg, 1.32 mmol, 1.0 eq.) was dissolved in a heat-dried round-bottom flask in anhydrous tetrahydrofuran (10 mL). The colorless solution was cooled down to –78 °C and lithiumbis(trimethylsilyl)amide (1 M in THF, 2.78 mL, 2.78 mmol, 2.1 eq.) was added dropwise to the solution. After complete addition, a dark brown solution was observed. The reaction mixture was stirred for 15 minutes at –78 °C and

afterwards the cooling bath was removed for five minutes to guarantee complete deprotonation, before cooling down again to –78 °C. After 10 minutes trimethylsilyl chloride (302 mg, 2.78 mmol, 2.1 eq.) was added dropwise via a syringe to the now dark orange solution. After complete addition, the solution was warmed up to room temperature and was stirred for further two hours under an argon atmosphere. Then the solvent was removed under reduced pressure. The brown oil was dissolved in dry *n*-hexane (50 mL) and afterwards the solution was filtrated. Afterwards the solvent was removed under reduced pressure and the brown oil **5** was used directly without further purification and purity control for the next reaction step.

Under an argon atmosphere the TMS-protected aniline **11** (400 mg, 1.10 mmol, 3.27 eq.) was dissolved in a heat-dried round-bottom flask in anhydrous tetrahydrofuran (5.5 mL). The brown solution was cooled down to –78 °C and was stirred for further 20 minutes at this temperature. At –78 °C *tert*-butyllithium (1.6 M in pentane, 1.40 mL, 2.24 mmol, 6.67 eq.) was added dropwise to the solution. *CAUTION*: *solutions* of *tert-butyllithium react explosively with* water *and may ignite in moist air.* The red solution was stirred for 30 minutes at –78 °C. Afterwards the Si-xanthone **5** (100 mg, 336 µmol, 1.0 eq.) was dissolved in dry tetrahydrofuran (10 mL) and was added via a syringe within seconds to the reaction mixture at –78 °C. The color of the solution turned to a dark orange. After complete addition of **5**, the cooling bath was quickly removed and the reddish solution was stirred for three hours which led to almost complete conversion of **5** as monitored by TLC (DCM:MeOH: 9:1). Then hydrochloric acid (1 N, 5 mL) and deionized water (100 mL) were added to the red solution and the solution turned gradually dark blue. The dark solution was extracted with DCM (3 x 250 mL) until the aqueous solution became nearly colorless. The combined organic phases were washed with brine and were dried over sodium sulfate. After filtration, the solvent was removed by evaporating. Then the blue crude product was purified by flash column chromatography (silica gel, DCM:MeOH 99:1 to 88:12). The concentrated and combined fractions from the column were filtrated over cotton wool to afford the Si-rhodamine **12** as a dark blue solid. For high purity and to verify the counter ion as a trifluoroacetate ion the product was purified by reverse-phase HPLC (10–90% MeCN/H_2O, linear gradient in 40 minutes, with constant 0.1% v/v trifluoroacetic acid (TFA) additive, *system* 1) to obtain the corresponding Si-rhodamine **12** (128 mg, 249 µmol, 74%) as a gleaming dark blue solid.

^{1}H-NMR (400 MHz, MeOD-d_4, 300 K):
δ (ppm)= 7.39–7.13 (m, 8 H, H_{arom}), 6.78 (dd, *J*= 9.7 Hz, 2.9 Hz, 2 H, H_{arom}), 3.33 (s, 12 H, N-CH_3), 0.58 (s, 6 H, Si-CH_3).

^{13}C-NMR (101 MHz, MeOD-d_4, 300 K):

δ (ppm)= 171.4, 155.6, 149.5, 143.4, 137.8, 132.3, 131.3, 129.2, 122.0, 118.6, 114.7, 40.8, –1.1.

^{19}F-NMR (376 MHz, MeOD-d_4, 300 K):

δ (ppm)= –76.9 (CF_3COO^-).

IR (ATR, neat):

υ [cm^{-1}]= 2931 (br), 1733 (s), 1688 (s), 1572 (vs).

R_f value (dichloromethane/methanol 90:10)= 0.52.

HR-ESI-MS:

$C_{25}H_{30}N_3Si^+$	Calculated *m/z*	found *m/z*
$[M]^+$	400.2204	400.2204

Table S1: Optical properties of **12** in different solvents and the quantum yield using Nile Blue A as reference.

**Solvent**	$\lambda_{abs, max}$	λ_{em}	ε_{max}	**Quantum Yield**
DMSO	655 nm	683 nm	84.200 $M^{-1}cm^{-1}$	<0.01
PBS (pH= 7.4)	644 nm	667 nm	69.100 $M^{-1}cm^{-1}$	<0.01

2.8 The synthesis of Si-rhodamine 13

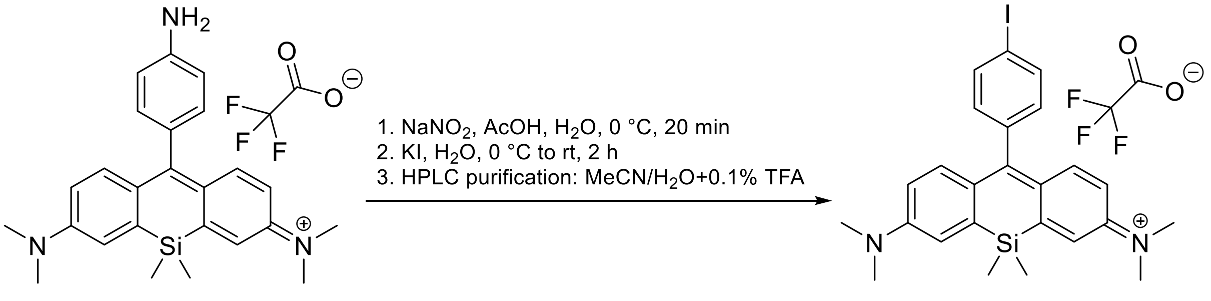

12 92% **13**

The amine-functionalized Si-rhodamine **12** (20 mg, 38.9 µmol, 1.0 eq.) was dissolved in a 2:1 ratio of acetic acid (2.00 mL) and deionized water (1 mL). The dark blue solution was cooled down to 0 °C and a solution of sodium nitrite (4.03 mg, 58.4 µmol, 1.5 eq.) in deionized water (100 µL) was added. The color changed from dark blue to a slightly green-blue solution. After 20 minutes, potassium iodide (12.9 mg, 77.8 µmol, 2.0 eq.) in deionized water (100 µL) was added at 0 °C. After 20 minutes, the cooling bath was removed. A color change back to a dark

blue combined with bubbling was observed. The reaction mixture was stirred for one hour at room temperature. Afterwards the solvents were carefully removed under reduced pressure. The blue solution was diluted with water and dichloromethane (50 mL). Then the organic phase was separated. The aqueous phase was extracted with dichloromethane (3 x 100 mL). The combined organic phases were washed with brine and dried over sodium sulfate. After filtration, the solvent was removed under vacuum. The crude product was purified by flash column chromatography (silica gel, dichloromethane/methanol 99:1 to 95:5) to obtain Si-rhodamine **13** as a dark blue solid. For high purity and to verify the counter ion as a trifluoroacetate ion the product was purified by reverse-phase HPLC (30–90% MeCN/H_2O, linear gradient in 35 minutes, with constant 0.1% v/v trifluoroacetic acid additive, *system* 2) to receive the corresponding Si-rhodamine **13** (22.4 mg, 35.8 µmol, 92%) as a dark blue solid.

^{1}H-NMR (400 MHz, $CDCl_3$, 300 K):
δ (ppm)= 7.87 (d, *J*= 7.7 Hz, 2 H, H_{arom}), 7.19–7.08 (m, 4 H, H_{arom}), 6.99 (d, *J*= 7.8 Hz, 2 H, H_{arom}), 6.61 (d, *J*= 9.3 Hz, 2.9 Hz, 2 H, H_{arom}), 3.34 (s, 12 H, N-CH_3), 0.57 (s, 6 H, Si-CH_3).

^{13}C-NMR (101 MHz, $CDCl_3$, 300 K):
δ (ppm)= 168.3, 154.2, 148.8, 142.3, 138.5, 137.6, 131.2, 127.8, 121.4, 114.0, 94.8, 41.6, –0.3.

^{19}F-NMR (376 MHz, $CDCl_3$, 300 K):
δ (ppm)= –75.6 (CF_3COO^-).

IR (ATR, neat):
υ [cm^{-1}]= 3076 (br), 2930 (br), 1687 (s), 1574 (vs).

R_f value (dichloromethane/methanol 90:10)= 0.65.
R_f value (dichloromethane/methanol 95:5)= 0.12.

Retention time (*system* 5, HPLC 25–75)= 25.04 min.

HR-ESI-MS:

$C_{25}H_{28}IN_2Si^+$	Calculated *m/z*	found *m/z*
$[M]^+$	511.1061	511.1066

2.9 The synthesis of copper complex [Cu(OTf)$_2$(impy)$_4$][11]

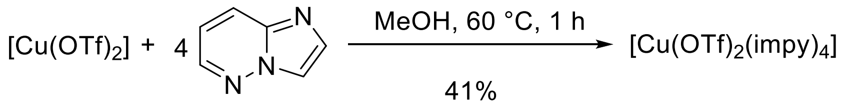

Under an argon atmosphere the copper(II)triflate complex (500 mg, 1.38 mmol, 1.0 eq.) was dissolved in dry methanol (3 mL). The slightly blue solution was warmed up to 60 °C. Then the ligand imidazo[1,2-*b*]pyridazine (663 mg, 5.57 mmol, 4.0 eq.) in dry methanol (1 mL) was added and the reaction mixture turned immediately blue. The blue solution was stirred for one hour at 60 °C. Afterwards the blue reaction mixture was allowed to cool down to room temperature and the solvent was removed under reduced pressure. Finally the blue residue was washed with diethyl ether (50 mL), DCM (10 mL) and DCM/MeOH: 9:1 (5 mL). The blue solid was dried for nine hours at 60 °C as well as overnight at room temperature at high vacuum. Tetrakis(imidazo[1,2-*b*]pyridazine)copper(II)triflate (477 mg, 569 µmol, 41%) was obtained as a pale blue solid.

Elemental Analysis

$C_{26}H_{20}CuF_6N_{12}O_6S_2 \cdot H_2O$ (*M*= 856.20 g/mol)

Calculated: C: 36.47%, H: 2.59%, N: 19.63%, S: 7.49%

Found: C: 36.71%, H: 2.40%, N: 19.70%, S: 7.58%.

IR (ATR, neat):

υ [cm^{-1}]= 3168 (br), 3117 (br), 3066 (w), 1619 (s), 1541 (s), 1504 (s).

2.10 Determination of the optical properties

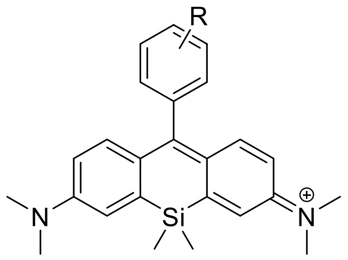

Each dye was dissolved in dry dimethyl sulfoxide (DMSO) to afford a general stock solution and was stored under argon atmosphere. The concentrations of the stock solutions were kept between 1.8 mM to 2.0 mM. For determination of the optical properties, the samples were measured directly in DMSO or mixtures of DMSO in PBS with pH 7.4 starting from the prepared stock solutions. The final concentrations of all the samples were kept between

[11] T. C. Wilson, M. Xavier, J. Knight, S. Verhoog, J. B. Torres, M. Mosley, S. L. Hopkins, S. Wallington, P. D. Allen, V. Kersemans, R. Hueting, S. Smart, V. Gouverneur, B. Cornelissen, *J. Nucl. Med.* **2019**, *60*, 504–510.

0.2 µM and 1.2 µM. The samples in PBS of the final solutions did not exceed 1% v/v of DMSO. The extinction coefficient ε_{max} was calculated according to the Lambert-Beer equation (1) for the absorbance maximum while the maximum concentration of the dilution series was kept below 4.90 µM:

$$E = c \cdot \varepsilon \cdot d \qquad (1)$$

E means the extinction, *c* stands for the concentration, *ε* gives the molar absorption coefficient and *d* describes the thickness of the used cuvette (*d*= 1 cm). An example plot for the determination of the molar absorption coefficient of Si-rhodamine **10a** in DMSO is shown in Figure S1.

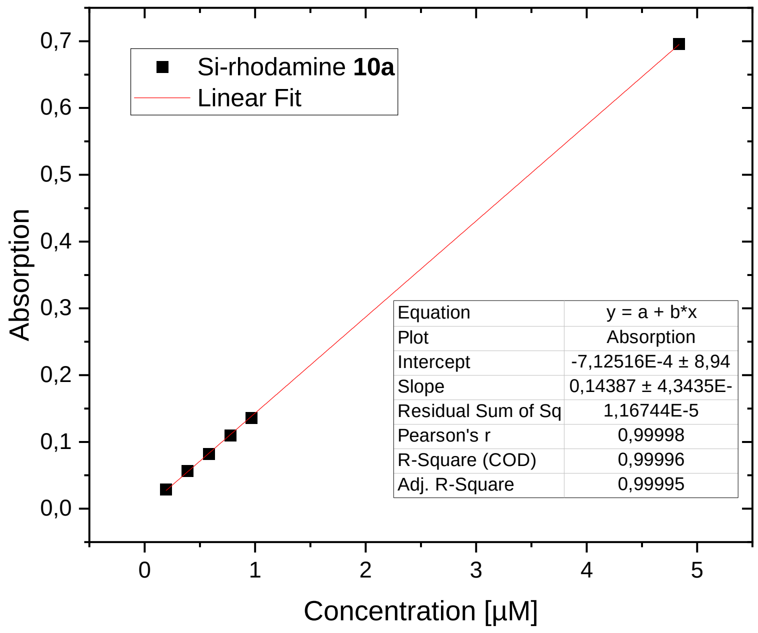

Figure S1: Illustration of the determination of the molar absorption coefficient ε_{max} for Si-rhodamine **10a** in DMSO and a dilution series of 5.

For determination of the quantum yield, the dyes were diluted in a dilution series leading to an absorbance between 0.01 and 0.1 to avoid non-linear effects (re-absorption) effects for all the presented dyes.[12] The absorbance at maximum was plotted versus the integrated fluorescence intensity and fitted by linear regression. The fit values of the slopes were used to calculate the fluorescence quantum yield according to the following equation:

$$QY_X = QY_S \cdot \frac{m}{m_{ref}} \cdot \frac{\eta}{\eta_{ref}} \qquad (2)$$

According to equation (2), QY_x is the unknown quantum yield, QY_s the quantum yield of the reference substance Nile Blue A in ethanol (QY_s= 0.27), *m* and m_{ref} are the slopes of the linear

[12] S. Dhami, A. J. de Mello, G. Rumbles, S. M. Bishop, D. Phillips, A. Beeby, *Photochem. Photobiol* **1995**, *61*, 341–346.

fit of the integrated emission and η and η_{ref} are belonging to the refractive index of the corresponding solvent.[3] An example plot to obtain the required data for the quantum yield of Si-rhodamine **10a** in DMSO is shown in Figure S2.

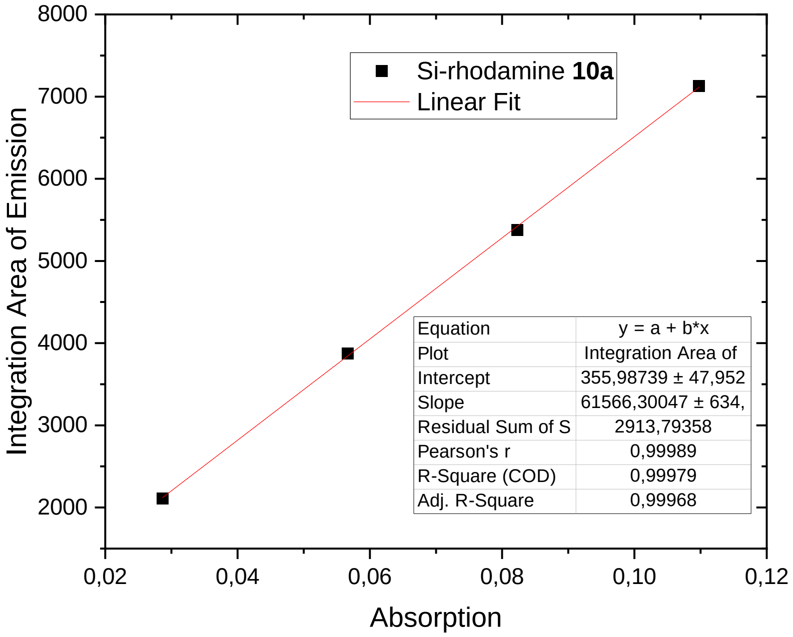

Figure S2: Illustration of the determination of the quantum yield for Si-rhodamine **10a** in DMSO and a dilution series of 4.

2.11 Copper-mediated radiofluorination (CMRF) screening experiments

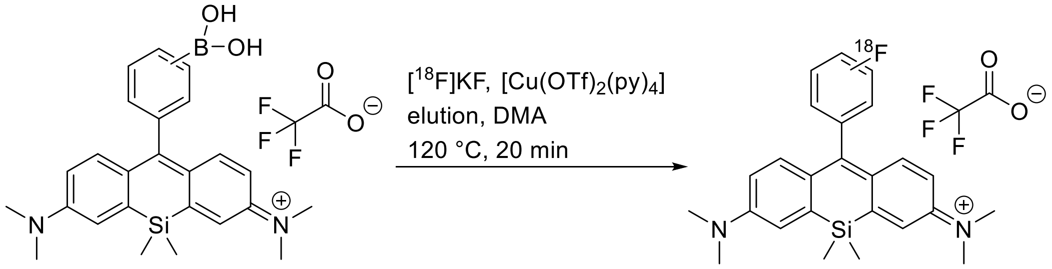

2.11.1 CMRF by using WAX cartridges

The weak anion exchange cartridge (WAX 1cc, 30 mg, oasis®, waters™) was preconditioned with 2-propanol (1 mL) and dried after flushing with nitrogen for two minutes. [^{18}F]Fluoride was trapped on WAX cartridges by passing the aqueous [^{18}F]fluoride solution (~100 MBq in 0.2 mL) from the female side through the cartridge. The cartridge was washed with isopropanol (1 mL) and dried by a stream of nitrogen for 3 min. The radioactivity was eluted

from the WAX cartridge from the female side using a solution of 4-(dimethylamino)-pyridinium triflate ($DMAPH^+OTf^-$, 6.8 mg, 25 µmol) in DMA or DMF (500 µL; elution efficiency: >80%).[13] Aliquots of the eluate were added to HPLC vials followed by addition of the precursor (0.4–1.6 µmol, final concentration: 10–40 mM) and the copper agent $[Cu(OTf)_2(py)_4]$ (0.4–1.6 µmol; final concentration: 10–40 mM) dissolved in DMA or DMF, respectively. The total reaction volume was either 40 µL, 50 µL or 160 µL. The vial was sealed and the dark blue reaction mixture was heated up to 100–160 °C for 20–40 minutes.

Afterwards the reaction mixture was allowed to cool down to room temperature and the solution was diluted with a 1:1 mixture of water and acetonitrile. The radiochemical conversions (RCCs) were determined by using UHPLC (water+0.1% trifluoroacetic acid/ acetonitrile) and radio-TLC (TLC-plates: SiO_2; mobile phase: dichloromethane/methanol 85:15). The RCCs were determined by dividing the integrated peak area of **[^{18}F]10a** by the sum of all peak areas originating from ^{18}F-containing substances in the chromatogram as demonstrated in Figure S3. The respective non-radioactive Si-rhodamine **10a** was used as reference for the fluorine-18 labeled compound **[^{18}F]10a** in UHPLC (*system* 5, UHPLC 25–75) and radio-TLC analyses.

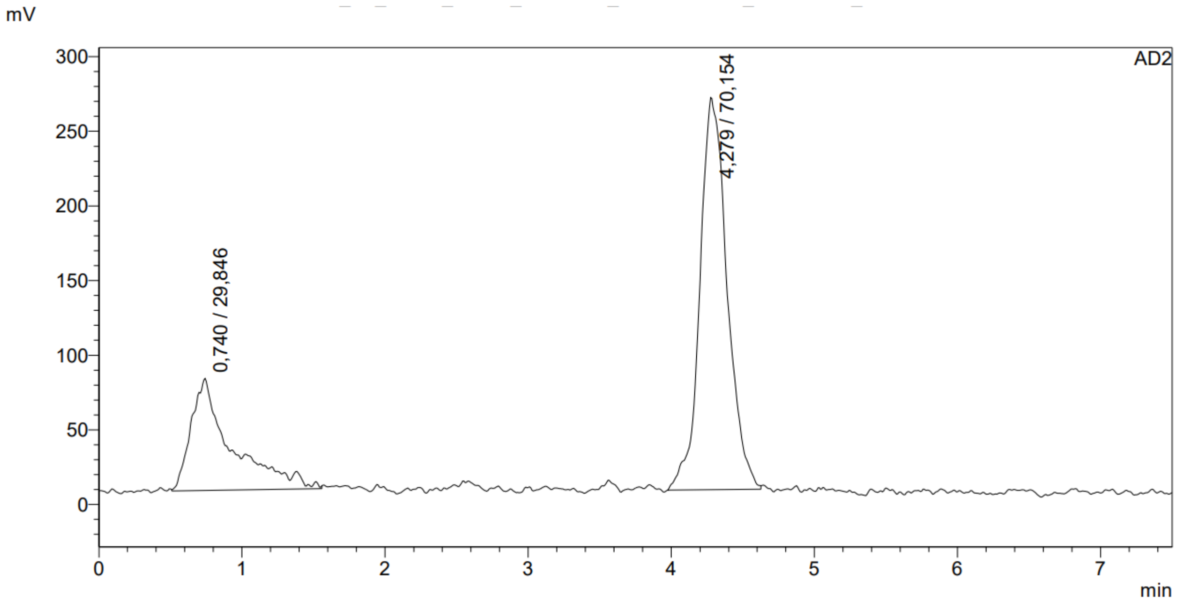

Figure S3: Exemplary radioactivity signal of the UHPLC chromatogram corresponding to radiofluorination of Si-rhodamine of **6a** (table S6, entry: 2). The integrated area under the curve from the free [^{18}F]fluoride and the product signal from **[^{18}F]10a** was used for calculation of RCC by UHPLC (*system* 5, UHPLC 25–75).

[13] D. Antuganov, M. Zykov, V. Timofeev, K. Timofeeva, Y. Antuganova, V. Orlovskaya, O. Fedorova, R. Krasikova, *Eur. J. Org. Chem.* **2019**, *5*, 918–922.

Table S2: Overview of the radiofluorination screening experiments for the precursors **6a**, **6b** and **6c** after elution with 4-(dimethylamino)-pyridinium triflate over a WAX cartridge by using different reaction time, temperatures, various amounts of copper agent and precursor. The RCCs were determined by using UHPLC (*system* 5, UHPLC 25–75) or TLC (SiO_2; mobile phase: dichloromethane/methanol 85:15).[13]

Entry	Precursor	Solvent	Time [min]	Temperature [°C]	Total reaction volume [µL]	Precursor: Copper(II) complex [amount]	RCC (HPLC)	RCC (TLC)
1	**6a**	DMA	20	100	40	1 (0.4 µmol):4 (1.6 µmol)	3%	6%
**2**	**6a**	DMA	20	110	40	1 (0.4 µmol):4 (1.6 µmol)	6%	8%
**3**	**6a**	DMA	20	120	50	7:4	12%	-
**4**	**6a**	DMA	20	120	40	4 (1.6 µmol):1 (0.4 µmol)	0%	0%
**5**	**6a**	DMA	20	120	40	1 (0.4 µmol):4 (1.6 µmol)	25%	23%
**6**	**6a**	DMF	20	120	40	1 (0.4 µmol):4 (1.6 µmol)	0%	0%
**7**	**6a**	DMA	20	120	160	1 (0.4 µmol):4 (1.6 µmol)	22%	21%
**8***	**6a**	DMA	20	120	40	1 (0.4 µmol):4 (1.6 µmol)	15%	13%
**9**	**6a**	DMA	40	120	40	1 (0.4 µmol):4 (1.6 µmol)	10%	8%
**10**	**6a**	DMA	20	140	40	1 (0.4 µmol):4 (1.6 µmol)	10%	6%
**11**	**6a**	DMA	40	140	40	1 (0.4 µmol):4 (1.6 µmol)	5%	3%
**12**	**6a**	DMA	20	160	40	1 (0.4 µmol):4 (1.6 µmol)	0%	1%
**13**	**6a**	DMA	40	160	40	1 (0.4 µmol):4 (1.6 µmol)	0%	1%
**14**	**6b**	DMA	20	120	40	1 (0.4 µmol):4 (1.6 µmol)	0%	-
**15**	**6b**	DMA	20	120	40	1 (0.4 µmol):4 (1.6 µmol)	0%	-
**16**	**6c**	DMA	20	120	40	1 (0.4 µmol):4 (1.6 µmol)	0%	-
**17**	**6c**	DMA	20	120	40	1 (0.4 µmol):4 (1.6 µmol)	0%	-

Table S3: Dependency of reaction temperature and reaction time on radiochemical conversion (RCC, determined by UHPLC (*system* 5, UHPLC 25–75)) for the radiofluorination of **6a** after eluting with 4-(dimethylamino)-pyridinium triflate over a WAX cartridge by using the precursor **6a** in a 1:4 ratio compared to the copper agent in a reaction volume of 40 µL.[13]

Temperature	RCC after 20 min	RCC after 40 min
100 °C	1,5±2,1% (*n*=2)	-
110 °C	4,9±2,1% (*n*=2)	-
120 °C	16,7±3,8% (*n*=7)	10,4% (*n*=1)
140 °C	10,4% (*n*=1)	4,9% (*n*=1)
160 °C	0% (*n*=1)	0% (*n*=1)

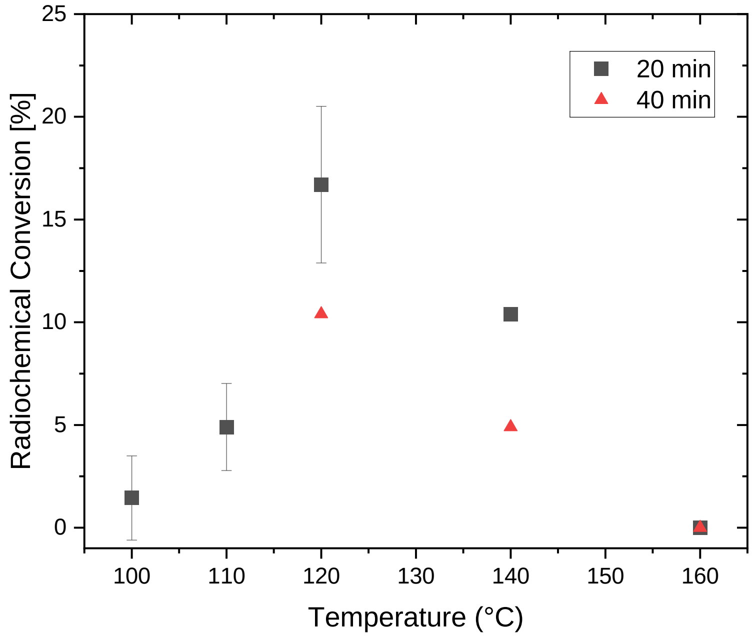

Figure S4: Dependency of reaction temperature and reaction time on radiochemical conversion (RCC, determined by UHPLC (*system* 5, UHPLC 25–75)) for the radiofluorination of **6a** by using the precursor **6a** in a 1:4 ratio compared to the copper agent in a reaction volume of 40 µL.

2.11.2 CMRF by using QMA cartridges

The quaternary methyl ammonium (QMA light, 46 mg, waters™) cartridge was preconditioned with $NaHCO_3$ (1 M, 10 mL) and water (10 mL). [^{18}F]Fluoride was trapped on QMA light cartridges by passing the aqueous [^{18}F]fluoride solution (~100 MBq in 0.2 mL) from the female side through the cartridge. The radioactivity was eluted from the QMA cartridge with a solution of potassium triflate (KOTf, 5 mg, 26.6 µmol) and potassium carbonate (K_2CO_3, 50 µg, 362 nmol) in acetonitrile (1 mL) and water (0.5 mL).[14,15] The elution efficiency was >88%. Then aliquots (60 µL) were added to HPLC vials and then the vials were sealed and dried at 130 °C for 3 min under a helium flow. The HPLC vials were allowed to cool down to room temperature. Subsequently a premixed solution (premix time 5 min) of precursor (10 µL, 0.4 µmol, final concentration: 10 mM), copper agent [$Cu(OTf)_2(py)_4$] or [$Cu(OTf)_2(impy)_4$] (10 µL, 1.6 µmol; final concentration: 40 mM) and DMA or 1,3-dimethyl-2-imidazolidinone (DMI) (20 µL) were added to the fluorine-18 containing HPLC vial.

Alternatively, the elution was performed with tetraethylammonium bicarbonate (2.7 mg, 14.1 µmol) in *n*-butanol (400 µL). Afterwards aliquots (20 µL) were added to HPLC vials.

[14] M. Tredwell, S. M. Preshlock, N. J. Taylor, S. Gruber, M. Huiban, J. Passchier, J. Mercier, C. Genicot, V. Gouverneur, *Angew. Chem. Int. Ed. Engl.* **2014**, *53*, 7751–7755.
[15] A. V. Mossine, A. F. Brooks, K. J. Makaravage, J. M. Miller, N. Ichiishi, M. S. Sanford, P. J. Scott, *Org. Lett.* **2015**, *17*, 5780–5783.

Then premixed solutions (premix time 5 min) of precursor (10 µL, 0.4 µmol, final concentration: 10 mM) and copper agent $[Cu(OTf)_2(py)_4]$ (10 µL, 1.6 µmol; final concentration: 40 mM) in DMA were added to the fluorine-18 containing HPLC vial and finally pure DMA (20 µL) was added.

Another elution strategy was performed with tetraethylammonium bicarbonate (0.8 mg, 4.18 µmol) in methanol (800 µL). Afterwards aliquots (45 µL) were added to HPLC vials. The methanol was removed under a gentle helium flow at 70 °C. Then premixed solutions (premix time 5 min) of precursor **6a** in DMA and copper agent $[Cu(OTf)_2(py)_4]$ in DMA were added to the fluorine-18 containing HPLC vial and finally pure DMA (total volume: 45 µL) was added.

The reaction mixture was sealed and the dark blue solution was heated up to 120 °C for 20 minutes.

Finally, the reaction mixture was allowed to cool down to room temperature and the solution was five-fold diluted with a 1:1 mixture of water and acetonitrile. The radiochemical conversions (RCCs) were determined by using UHPLC (*system* 5, UHPLC 25–75) and radio-TLC (TLC-plates: SiO_2; mobile phase: dichloromethane/methanol 85:15). The RCCs were determined as described above. The respective non-radioactive Si-rhodamine **10a** or **10b** served as references for the fluorine-18 labeled compound **[^{18}F]10a** and **[^{18}F]10b** in UHPLC (*system* 5, UHPLC 25–75) and radio-TLC analyses (tables S4–S8).

Table S4: Elution with tetraethylammonium bicarbonate in *n*-butanol.

Entry	Precursor	Solvent	Time [min]	Temperature [°C]	Total reaction volume [µL]	Precursor: Copper(II) complex [amount]	RCC (HPLC)	RCC (TLC)
1	**6a**	DMA	20	120	45	1 (0.4 µmol):1 (0.4 µmol)	-	12%
**2**	**6a**	DMA	20	120	45	1 (0.4 µmol):4 (1.6 µmol)	16%	12%
**3**	**6a**	DMA	20	120	45	1 (0.4 µmol):5 (2.0 µmol)	19%	16%
**4**	**6a**	DMA	20	120	45	4 (1.6 µmol):1 (0.4 µmol)	-	4%
**5**	**6a**	DMA	20	120	30	1 (0.4 µmol):4 (1.6 µmol)	-	15%
**6**	**6b**	DMA	20	120	30	1 (0.4 µmol):4 (1.6 µmol)	-	0%
**7**	**6c**	DMA	20	120	30	1 (0.4 µmol):4 (1.6 µmol)	-	0%

Table S5: Elution with tetraethylammonium bicarbonate in methanol and removal of solvent.

Entry	Precursor	Solvent	Time [min]	Temperature [°C]	Total reaction volume [µL]	Precursor: Copper(II) complex [amount]	RCC (HPLC)	RCC (TLC)
1	**6a**	DMA	20	120	45	1 (0.4 µmol):1 (0.4 µmol):	-	0%
**2**	**6a**	DMA	20	120	45	1 (0.4 µmol):4 (1.6 µmol)	6%	8%
**3**	**6a**	DMA	20	120	45	1 (0.4 µmol):5 (2.0 µmol)	10%	10%
**4**	**6a**	DMA	20	120	45	4 (1.6 µmol):1 (0.4 µmol)	-	0%

Table S6: Elution with KOTf/K_2CO_3 and azeotropic drying.

Entry	Precursor	Solvent	Time [min]	Temperature [°C]	Total reaction volume [µL]	Precursor: Copper(II) complex [amount]	RCC (HPLC)	RCC (TLC)
**1**	**6a**	DMA	20	120	40	1 (0.4 µmol):4 (1.6 µmol)	35%	-
**2**	**6a**	DMI	20	120	40	1 (0.4 µmol):4 (1.6 µmol)	70%	70%
**3**	**6b**	DMI	20	120	40	1 (0.4 µmol):4 (1.6 µmol)	48%	-
**4**	**6b**	DMA	20	120	40	1 (0.4 µmol):4 (1.6 µmol)	0%	-
**5**	**6c**	DMA	20	120	40	1 (0.4 µmol):4 (1.6 µmol)	0%	-
**6**	**6c**	DMA	20	120	40	1 (0.4 µmol):4 (1.6 µmol)	0%	-
**7**	**6c**	DMI	20	120	40	1 (0.4 µmol):4 (1.6 µmol)	0%	-

Table S7: Elution with KOTf/K_2CO_3 and azeotropic drying with [Cu(OTf)$_2$(impy)$_4$].

Entry	Precursor	Solvent	Time [min]	Temperature [°C]	Total reaction volume [µL]	Precursor: Copper(II) complex [amount]	RCC (HPLC)	RCC (TLC)
**1**	**6a**	DMA	20	120	40	1 (0.4 µmol):4 (1.6 µmol)	18%	23%
**2**	**6a**	DMI	20	120	40	1 (0.4 µmol):4 (1.6 µmol)	14%	24%
**3**	**6b**	DMI	20	120	40	1 (0.4 µmol):4 (1.6 µmol)	0%	-

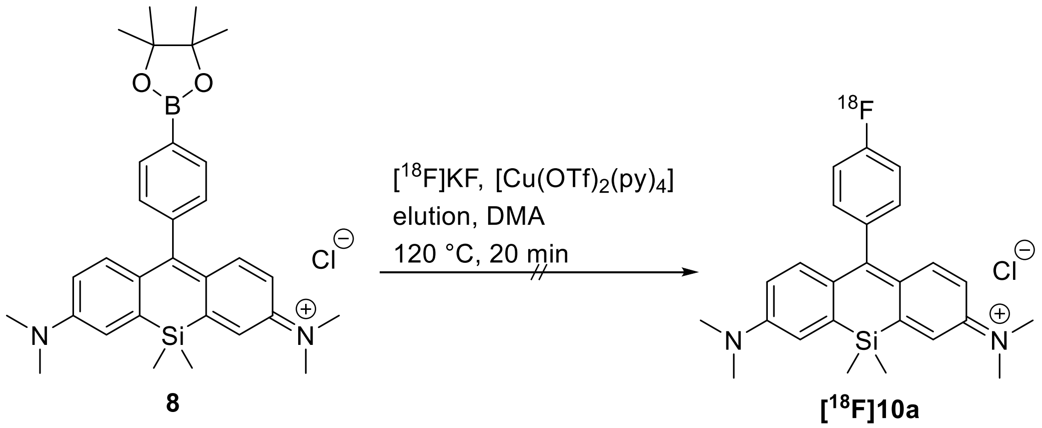

Table S8: Elution with KOTf/K_2CO_3 and azeotropic drying.

Entry	Precursor	Solvent	Time [min]	Temperature [°C]	Total reaction volume [µL]	Precursor: Copper(II) complex [amount]	RCC (HPLC)	RCC (TLC)
**1**	**8**	DMA	20	120	40	1 (0.4 µmol):4 (1.6 µmol)	0%	-
**2**	**8**	DMI	20	120	40	1 (0.4 µmol):4 (1.6 µmol)	0%	-

2.11.3 Determination of the radiochemical yield

Elution with 4-(dimethylamino)-pyridinium triflate:

The weak anion exchange cartridge (WAX 1cc, 30 mg, oasis®, waters™) was preconditioned with 2-propanol (1 mL) and was dried after flushing with nitrogen for two minutes. [^{18}F]Fluoride was trapped on WAX cartridges by passing the aqueous [^{18}F]fluoride solution (~400 MBq in 0.4 mL) from the female side through the cartridge. The cartridge was washed with isopropanol (1 mL) and dried by a stream of nitrogen for 3 min. The radioactivity was eluted from the WAX cartridge from the female side using a solution of 4-(dimethylamino)-pyridinium triflate (DMAPH$^+$Otf$^-$, 6.8 mg, 25 µmol) in DMA (500 µL; elution efficiency: >80%). Then an aliquot (20 µL) of the eluate was added to an HPLC vial, followed by the precursor (0.4 µmol,

final concentration: 10 mM, 10 µL) and the copper agent [Cu(Otf)$_2$(py)$_4$] (1.6 µmol; final concentration: 40 mM, 10 µL) dissolved in DMA. The total reaction volume was 40 µL. The reaction mixture was sealed and the dark blue solution was heated up to 120 °C for 20 minutes.

Afterwards the reaction mixture was allowed to cool down to room temperature and the solution was diluted with a 1:1 mixture of water and acetonitrile (2 mL). Then the dark blue solution was injected into the semi-preparative HPLC. The purification was performed with an isocratic method with water+0.1% trifluoroacetic acid (45%) and acetonitrile (55%) in 50 minutes (HPLC method: *system* 3; **[^{18}F]10a** (R_t= 38.0–40.0 min)). Afterwards the product containing fractions of **[^{18}F]10a** or **[^{18}F]10b** were collected, diluted with water, and passed through a Chromafix® C_{18} ec (s) cartridge (270 mg, conditioned with ethanol (10 mL) and water (10 mL)). Then the cartridge was washed with deionized water (ca. 8 mL). The elution of **[^{18}F]10a** or **[^{18}F]10b** was carried out with acetonitrile containing additional 0.1% trifluoroacetic acid (2 mL). The solvent was evaporated at 80 °C for **[^{18}F]10a** and at 55 °C for **[^{18}F]10b** under reduced pressure and a gentle flow of helium to complete dryness.

Table S9: Elution with 4-(dimethylamino)-pyridinium triflate in DMA.

Entry	Precursor	Solvent	Time [min]	Temperature [°C]	Total reaction volume [µL]	Precursor: Copper(II) complex [amount]	RCY±SD (number of experiments)
**1**	**6a**	DMA	20	120	40	1 (0.4 µmol):4 (1.6 µmol)	14.0±0.3% (*n*=3)

<u>Elution with KOTf/K_2CO_3 and azeotropic drying:</u>

The quaternary methyl ammonium (QMA light, 46 mg, waters™) cartridge was preconditioned with $NaHCO_3$ (1 M, 10 mL) and water (10 mL). [^{18}F]Fluoride was trapped on QMA light cartridges by passing the aqueous [^{18}F]fluoride solution (~500 MBq in 0.5 mL) from the female side through the cartridge. The radioactivity was eluted from the QMA cartridge with a solution of potassium triflate (KOTf, 5 mg, 26.6 µmol) and potassium carbonate (K_2CO_3, 50 µg, 362 nmol) in acetonitrile (1 mL) and water (0.5 mL). The elution efficiency was >88%. Then the aqueous aliquots (60 µL) were taken and were added to HPLC vials. Under a helium flow the aqueous solution was dried at 130 °C for 3 min in sealed vials. The HPLC vial was allowed to cool down to room temperature. Subsequently a premixed solution (premix time 5 min) of precursor (10 µL, 0.4 µmol, final concentration: 10 mM), copper agent [Cu(Otf)$_2$(py)$_4$] or [Cu(Otf)$_2$(impy)$_4$] (10 µL, 1.6 µmol; final concentration: 40 mM) and DMA or 1,3-dimethyl-2-imidazolidinone (DMI) (20 µL) was added to the fluorine-18 containing HPLC vial.

Afterwards the reaction mixture was allowed to cool down to room temperature and the solution was diluted with a 1:1 mixture of water and acetonitrile (2 mL). Then the dark blue solution was directly injected into the semi-preparative HPLC. The purification was performed

with an isocratic method containing water+0.1% trifluoroacetic acid (55%) and acetonitrile (45%) in 50 minutes (HPLC method: *system* 3; **[^{18}F]10a** (R_t= 38.0–40.0 min) or **[^{18}F]10b** (R_t= 39.0–41.5 min)). The product containing fractions of **[^{18}F]10a** or **[^{18}F]10b** were collected, diluted with water, and passed through a Chromafix® C_{18} ec (s) cartridge (270 mg, conditioned with ethanol (10 mL) and water (10 mL)). The cartridge was washed with deionized water (ca. 8 mL). The elution of **[^{18}F]10a** or **[^{18}F]10b** was carried out with acetonitrile containing additional 0.1% trifluoroacetic acid (2 mL). The solvent was evaporated at 80 °C for **[^{18}F]10a** and at 55 °C for **[^{18}F]10b** under reduced pressure and a gentle flow of helium to complete dryness. For in vitro stability studies, **[^{18}F]10a** or **[^{18}F]10b** were dissolved in pure ethanol.

Table S10: Elution with KOTf/K_2CO_3 and azeotropic drying.

Entry	Precursor	Solvent	Time [min]	Temperature [°C]	Total reaction volume [µL]	Precursor: Copper(II) complex [amount]	RCY±SD (number of experiments)
**1**	**6a**	DMA	20	120	40	1 (0.4 µmol):4 (1.6 µmol)	25±4% (*n*=3)
**2**	**6a**	DMI	20	120	40	1 (0.4 µmol):4 (1.6 µmol)	34±1% (*n*=2)
**3**	**6a**	DMI	20	120	200	1 (0.4 µmol):4 (1.6 µmol)	54±1% (*n*=2)
**4**	**6b**	DMI	20	120	120	1 (0.4 µmol):4 (1.6 µmol)	33% (*n*=1)

Proof of identity of **[^{18}F]10a** or **[^{18}F]10b** was performed by comparing the HPLC traces from the radioactive with the non-radioactive reference compounds **10a** and **10b** (figure S5).

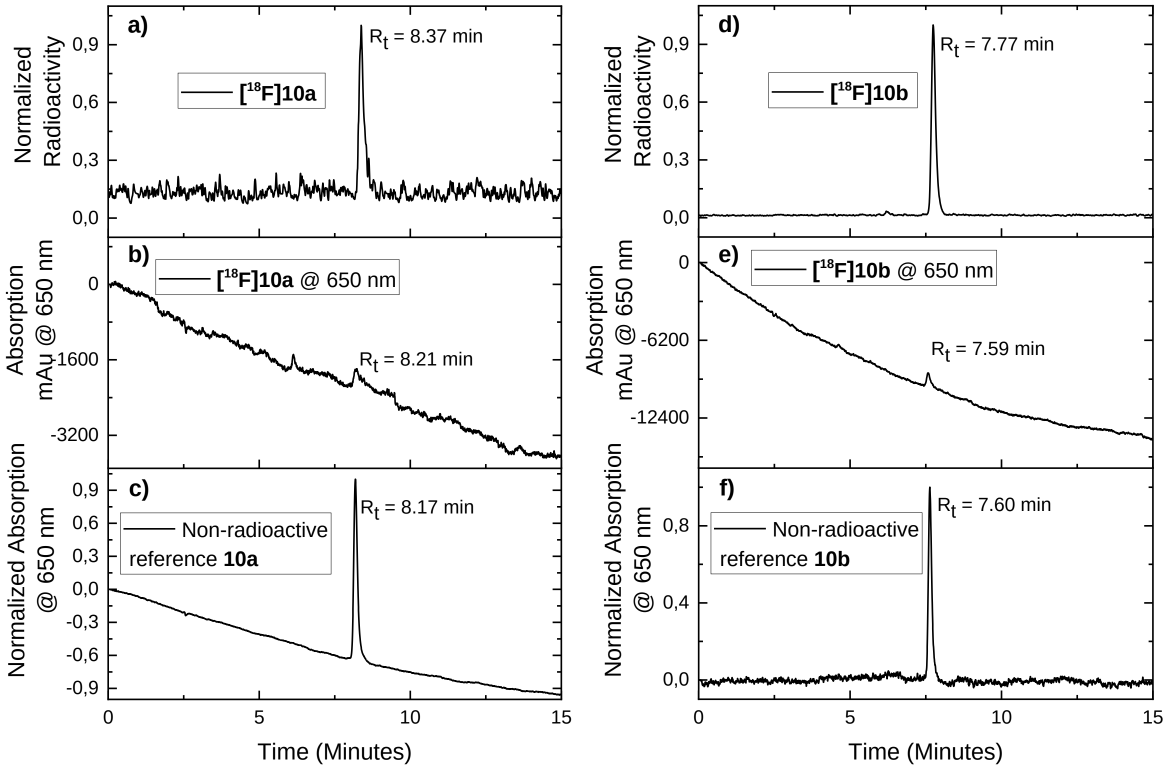

Figure S5: Confirmation of identity of SiR **[18F]10a** and **[18F]10b** applying an isocratic HPLC (*system* 5, HPLC 55 iso). A) Normalized HPLC chromatogram of the radiofluorinated SiR **[18F]10a** ([γ-Detection], retention time: 8.37 min). b) HPLC chromatogram of the corresponding radioactive SiR **[18F]10a** in NIR channel ([650 nm], retention time: 8.21 min). c) Normalized HPLC chromatogram of the non-radioactive reference SiR **10a** ([NIR, 650 nm], retention time: 8.17 min). d) Normalized HPLC chromatogram of the radiofluorinated SiR **[18F]10b** ([γ-Detection], retention time: 7.77 min). e) HPLC chromatogram of the corresponding radioactive SiR **[18F]10b** in NIR channel ([650 nm], retention time: 7.59 min). f) Normalized HPLC chromatogram of the non-radioactive reference SiR **10b** ([NIR, 650 nm], retention time: 7.60 min).

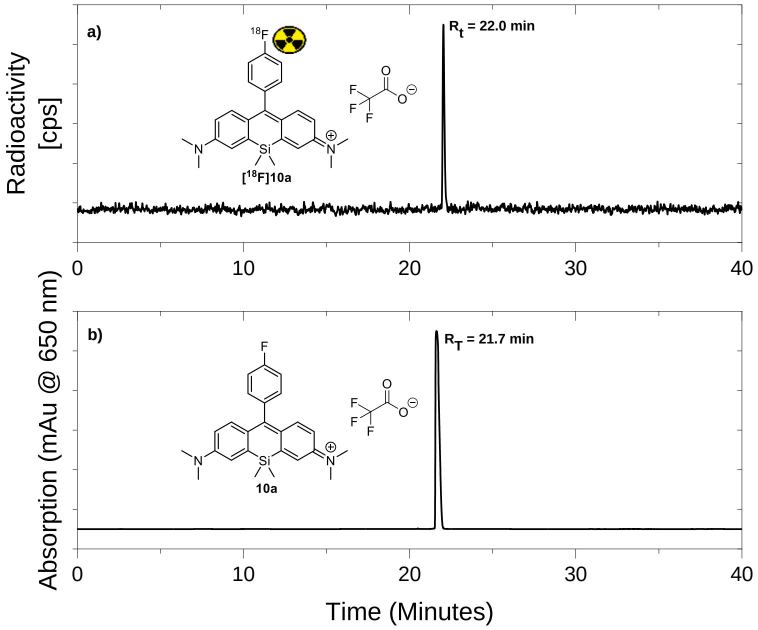

Figure S6: Confirmation of identity of SiR **[18F]10a** utilizing an HPLC method with gradient elution (HPLC method: *system* 5 25–75). A) HPLC chromatogram of the radiofluorinated SiR **[18F]10a** ([γ-Detection; intensity (cps)], retention time: 22.0 min). b) HPLC chromatogram of the non-radioactive reference SiR **10a** ([NIR, 650 nm], retention time: 21.7 min). The absorption was detected at a wavelength of 650 nm.

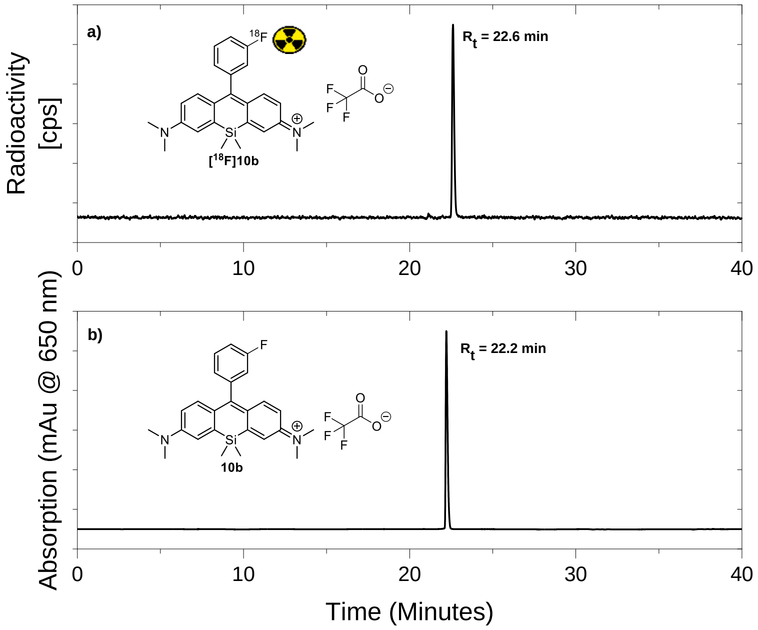

Figure S7: Confirmation of identity of SiR **[18F]10b** utilizing a HPLC method with gradient elution (HPLC method: *system* 5 25–75). A) HPLC chromatogram of the radiofluorinated SiR **[18F]10a** ([γ-Detection; intensity (cps)], retention time: 22.6 min). b) HPLC chromatogram of the non-radioactive reference SiR **10b** ([NIR, 650 nm], retention time: 22.2 min). The absorption was detected at a wavelength of 650 nm.

2.11.4 Determination of the molar activity

To determine the molar activity of the radiolabeled Si-rhodamines **[^{18}F]10a**, **[^{18}F]10b**, and **[^{123}I]13**, calibration lines for the corresponding non-radioactive reference compounds **10a**, **10b** or **13** were prepared by injection of an amount of 10–5000 femtomole on column into the HPLC. The area under the curve for fluorescence signals of the HPLC (excitation: λ=600 nm; emission: λ=640 nm) was correlated to the injected amount of substance and fitted to a linear regression model. The measurements were performed with an isocratic HPLC method (*system* 5, HPLC 55 iso). The molar activity of **[^{18}F]10a**, **[^{18}F]10b** or **[^{123}I]13** in the final product was obtained by dividing radioactivity which was injected into the HPLC by the calculated amount of the non-radioactive substance as derived from the respective fluorescence signal and the calibration curve.

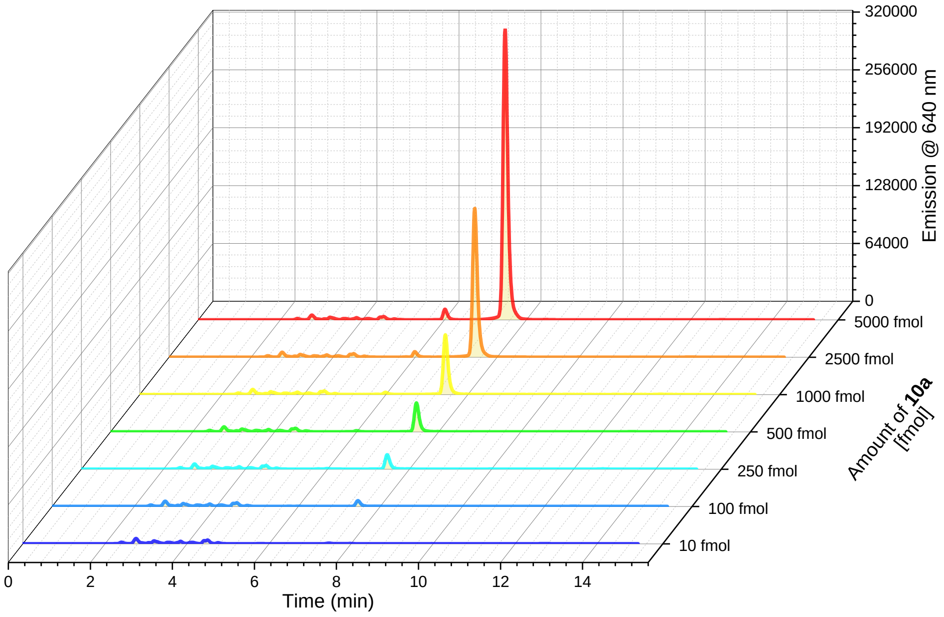

Figure S8: Dilution series and HPLC chromatograms of the emission from the non-radioactive SiR **10a** in molar amounts between 10 fmol and 5000 fmol for the determination of the molar activity in acetonitrile. The HPLC spectra were recorded by using the fluorescence detection (λ_{exc}= 600 nm; λ_{em}= 640 nm) with an isocratic HPLC method (*system* 5, HPLC 55 iso).

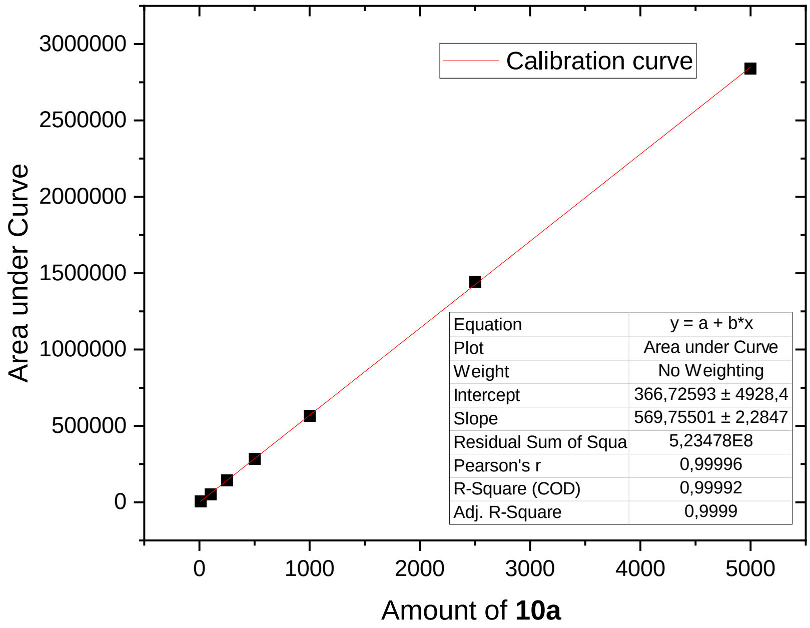

Figure S9: Calibration line for the determination of the molar activity of **10a**. Measured and integrated fluorescence detection (area under curve) of various dilution series in an amount of femtomoles and the corresponding linear fit.

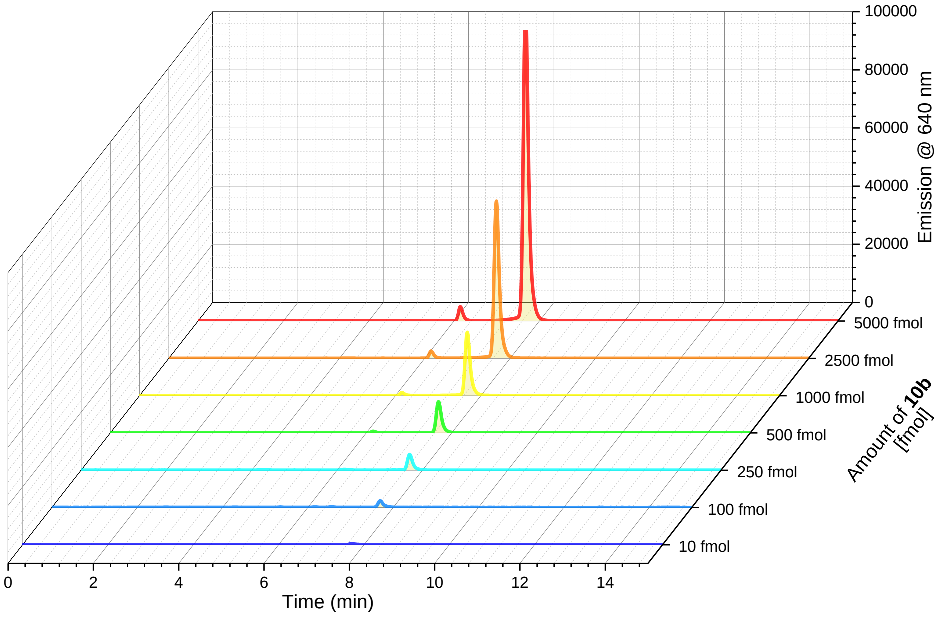

Figure S10: Dilution series and HPLC chromatograms of the emission from the non-radioactive SiR **10b** in molar amounts between 10 fmol and 5000 fmol for the determination of the molar activity in acetonitrile. The HPLC spectra were recorded by using the fluorescence detection (λ_{exc}= 600 nm; λ_{em}= 640 nm) with an isocratic HPLC method (*system* 5, HPLC 55 iso).

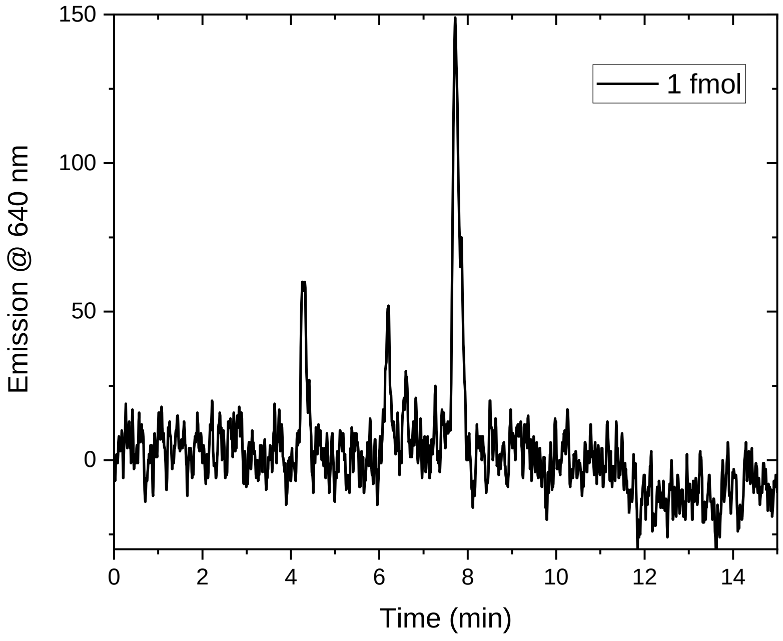

Figure S11: HPLC chromatogram of emission from 1 fmol on column of the non-radioactive SiR **10b** for the determination of the molar activity in acetonitrile. The HPLC spectra were recorded by using the fluorescence modus (λ_{exc}= 600 nm; λ_{em}= 640 nm) with an isocratic HPLC method (*system* 5, HPLC 55 iso).

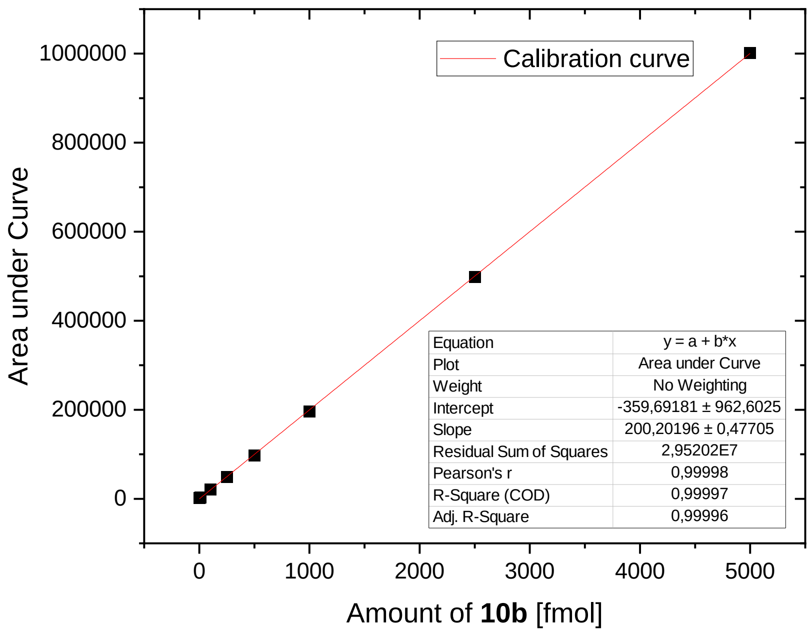

Figure S12: Calibration line for the determination of the molar activity of **10b**. Measured and integrated fluorescence detection (area under curve) of various dilution series in an amount of femtomoles and the corresponding linear fit.

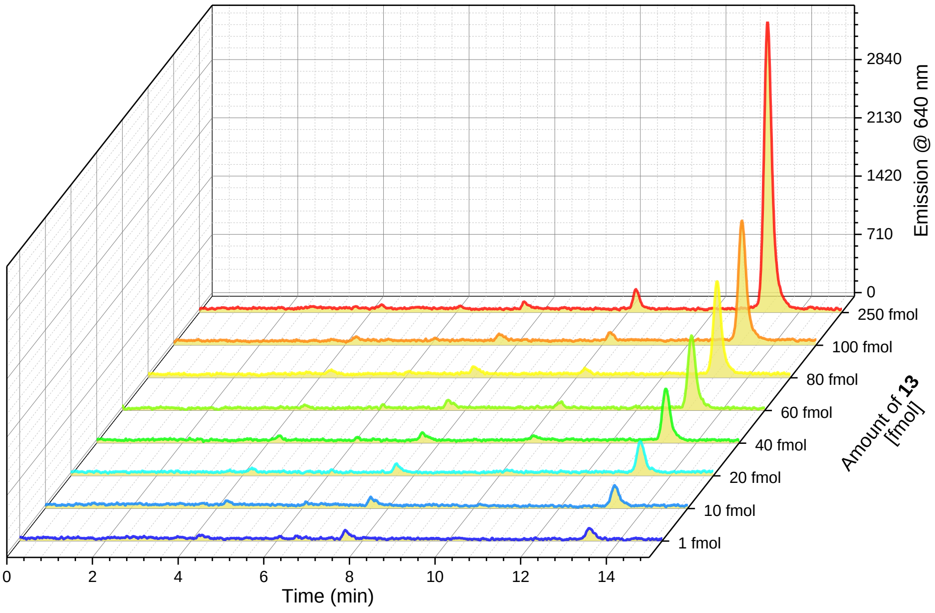

Figure S13: Dilution series and HPLC chromatograms of the emission from the non-radioactive SiR **13** in molar amounts between 10 fmol and 5000 fmol for the determination of the molar activity in acetonitrile. The HPLC spectra were recorded by using the fluorescence detection (λ_{exc}= 600 nm; λ_{em}= 640 nm) with an isocratic HPLC method (*system* 5, HPLC 55 iso).

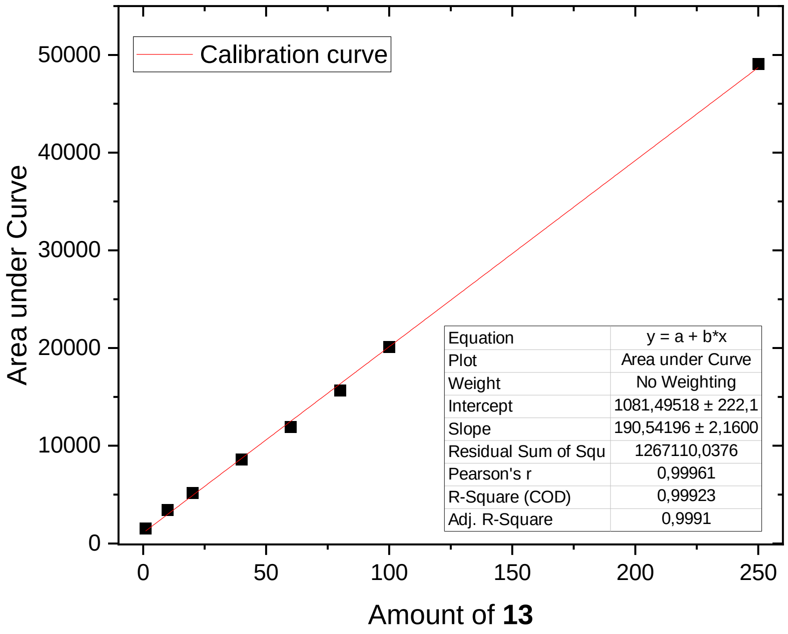

Figure S14: Calibration line for the determination of the molar activity of **13**. Measured and integrated fluorescence detection (area under curve) of various dilution series in an amount of femtomoles and the corresponding linear fit.

2.11.5 Determination of the partition coefficient

The partition coefficient of the purified **[^{18}F]10a**, **[^{18}F]10b** and **[^{123}I]13** was determined in PBS (*pH*= 7.4) and *n*-octanol using the shake flask method.[16, 17] For this purpose **[^{18}F]10a**, **[^{18}F]10b** or **[^{123}I]13** (1 MBq in 1 µL ethanol) were added into an Eppendorf vial containing phosphate-buffered saline (PBS, 600 µL) and *n*-octanol (600 µL). The mixture was vortexed for one minute at room temperature. The organic and aqueous phases were separated by using a centrifuge (5 minutes, 4 °C; 13.200 rpm). Then an aliquot from the *n*-octanol phase was withdrawn (400 µL) and was added to another Eppendorf vial containing PBS (400 µL). Again the vial was vortexed for two minutes. Afterwards the phases were separated by using the centrifuge (5 minutes, 4 °C; 13.200 rpm). Subsequently two aliquots (250 µL) from each phase were taken and the radioactive decay was counted with a borehole detector. The log *D* value at *pH*= 7.4 was calculated by using the following equation:

$$\log D_{\mathrm{ph=7.4}} = \log_{10}\frac{\mathrm{A_{oct}}}{\mathrm{A_{PBS}}} \qquad (2)$$

While A_{oct} describes the decay corrected counts per second from the *n*-octanol phase, the A_{PBS} stands for the decay corrected counts per second from the aqueous phosphate-buffered-saline (PBS) phase.

The reported value is the average of two or three measurements:

log $D_{\mathrm{ph=7.4}}$ (**[^{18}F]10a**)= 2.92±0.32 (*n*=3)

log $D_{\mathrm{ph=7.4}}$ (**[^{18}F]10b**)= 3.22±0.18 (*n*=2)

log $D_{\mathrm{ph=7.4}}$ (**[^{123}I]13**)= 3.48±0.29 (*n*=3).

2.11.6 Stability tests of [^{18}F]10a and [^{18}F]10b in saline and human serum

The *in vitro* stability of **[^{18}F]10a** and **[^{18}F]10b** was determined either in a solution of isotonic saline (0.9%) or in human serum. The Si-rhodamine **[^{18}F]10a** or **[^{18}F]10b** dissolved in ethanol (20 µL) was added to saline (380 µL) or human serum (380 µL) and incubated at room temperature or 37 °C for up to 120 minutes. For samples incubated in 5% ethanol in saline, aliquots were withdrawn at indicated time points, diluted with acetonitrile and analyzed by HPLC (*system* 5, HPLC 55 iso).

For samples incubated in human serum, samples were withdrawn after indicated time points, 5-fold diluted with acetonitrile, centrifuged and supernatants were separated and analyzed by HPLC. The stability was analyzed by analytical radio-HPLC (*system* 5, HPLC 55 iso). The degree of degradation was analyzed by integration of the area of **[^{18}F]10a** or **[^{18}F]10b** and the

[16] A. A. Wilson, L. Jin, A. Garcia, J. N. DaSilva, S. Houle, *Appl. Radiat. Isot.* **2001**, *54*, 203–208.
[17] M. Laube, M. Frizler, R. Wodtke, C. Neuber, B. Belter, T. Kniess, M. Bachmann, M. Gutschow, J. Pietzsch, R. Loser, *J. Label. Compd. Rad.* **2019**, *62*, 448–459.

corresponding peaks of newly formed byproducts. The formed byproducts were not further characterized.

Table S11: Overview of the stability test results from the radio-HPLC of **[^{18}F]10a** in a solution of saline (0.9%, *n*= 1) at room temperature or human serum (*n*= 3) at an incubation temperature of 37 °C after several time points.

Time	Percentage of intact compound after incubation in saline (5% EtOH) at rt	Percentage of intact compound after incubation in human serum at 37 °C
0 min	>99%	>99%
10 min	-	98±1%
30 min	-	90±2%
60 min	-	84±4%
120 min	>99%	80±8%

Table S12: Overview of the stability test results from the radio-HPLC of **[^{18}F]10b** in a solution of saline (0.9%, *n*= 1) at room temperature or human serum (*n*= 2) at an incubation temperature of 37 °C after several time points.

Time	Percentage of intact compound after incubation in saline (5% EtOH) at rt	Percentage of intact compound after incubation in human serum at 37 °C
0 min	>99%	>99%
10 min	-	96±1%
30 min	-	92±2%
60 min	-	83±1%
120 min	>99%	74±1%

2.12 Copper-mediated radioiodination (CMRI) of SiR 6a

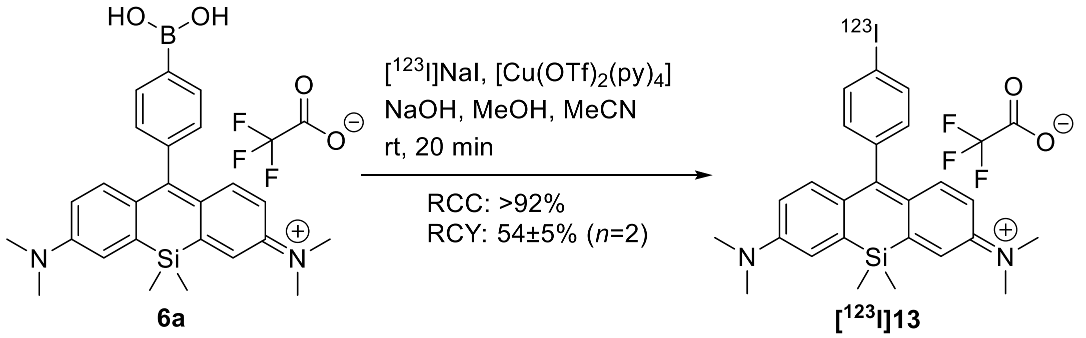

CAUTION: *the radiolabeling and working with iodine-123 was performed in specific radionuclide hoods for iodine-123 with enhanced exhausting filters to prevent iodine-123 spreading and contamination.*

The copper-mediated radioiodination was performed according to a previously reported method.[18, 19] A solution of precursor **6a** (130 µg, 240 nmol, final concentration: 0.8 mM) in acetonitrile (60 µL) and [Cu(Otf)$_2$(py)$_4$] (814 µg, 1.20 µmol; final concentration: 4 mM) in

[18] T. C. Wilson, G. McSweeney, S. Preshlock, S. Verhoog, M. Tredwell, T. Cailly, V. Gouverneur, *Chem. Commun.* **2016**, *52*, 13277–13280.

[19] M. Laube, F. Brandt, K. Kopka, H.-J. Pietzsch, J. Pietzsch, R. Loeser, R. Wodtke, *Nucl. Med. Biol.* **2021**, *96*, S79–S80.

methanol (240 µL) were mixed and added within 2 min to a solution of [^{123}I]iodide (180 MBq Na[^{123}I]I in 10 µL NaOH (0.02 M)). Without stirring, the reaction mixture was allowed to react for 20 minutes at room temperature. Afterwards the solution was diluted with a 1:1 mixture of water and acetonitrile (1.6 mL). Then the blue reaction mixture was directly injected into the semi-preparative HPLC (*system* 4). The purification was performed with a linear gradient method containing 75% water with 0.1% v/v trifluoroacetic acid (TFA) and 25% acetonitrile with 0.1% v/v trifluoroacetic acid (TFA) to 25% water with 0.1% v/v trifluoroacetic acid (TFA) and 75% acetonitrile with 0.1% v/v trifluoroacetic acid (TFA) in 33 min (*system* 4; R_t= 30–31 min). Subsequently the fraction containing **[^{123}I]13** was diluted with DI water (20 mL) and was trapped on a Chromafix® C_{18} ec (s) cartridge (270 mg, conditioned with ethanol (10 mL) and water (10 mL)). Afterwards the cartridge was washed with DI water (ca. 5 mL). The elution of **[^{123}I]13** was carried out with acetonitrile containing additional 0.1% trifluoroacetic acid (2 mL). The solvent was evaporated at 80 °C under reduced pressure and a gentle flow of nitrogen to complete dryness. **[^{123}I]13** (with radiochemical purity of 96%) was redissolved in ethanol for determination of molar activity and partition coefficient log*D*.[20, 21] The radiochemical purity was between 80% and 96% with a radiochemical yield of 54±5% (*n*=2).

Note: It is important to know that the radiochemical purity after HPLC purification as well as the SPE was over 99%. However, after evaporation of the solution in acetonitrile containing 0.1% TFA at different temperatures (50°C and 70°C) showed a decrease in radiochemical purity indicating instability during evaporation at higher temperatures (figures S15–S-18).

[20] A. A. Wilson, L. Jin, A. Garcia, J. N. DaSilva, S. Houle, *Appl. Radiat. Isot.* **2001**, *54*, 203–208.
[21] M. Laube, M. Frizler, R. Wodtke, C. Neuber, B. Belter, T. Kniess, M. Bachmann, M. Gutschow, J. Pietzsch, R. Loser, *J. Label. Compd. Rad.* **2019**, *62*, 448–459.

Analytical radio-HPLC analysis after semi-preparative HPLC purification (*system* 5, HPLC 25–75) with RCP>99%:

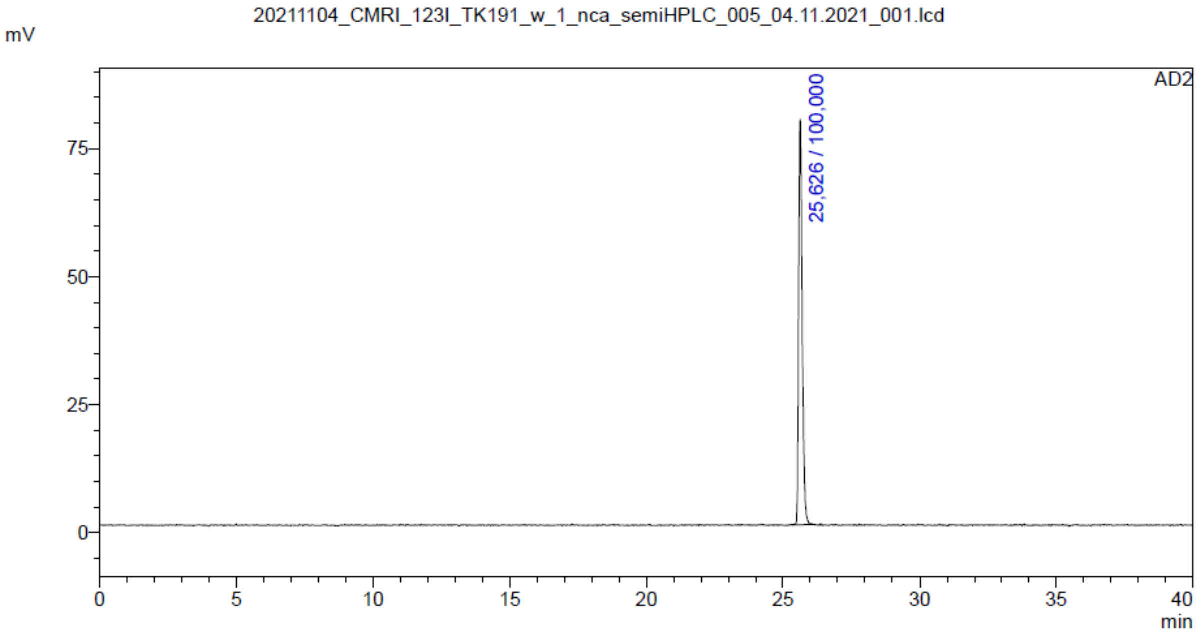

Figure S15: Analytical radio-HPLC chromatogram of **[^{123}I]13** ([γ-Detection], R_t=25.6 min) after semi-preparative HPLC purification with a radiochemical purity over 99%. HPLC-Method (*system* 5, HPLC 25–75).

Analytical radio-HPLC analysis after SPE (*system* 5, HPLC 25–75) with RCP>99%:

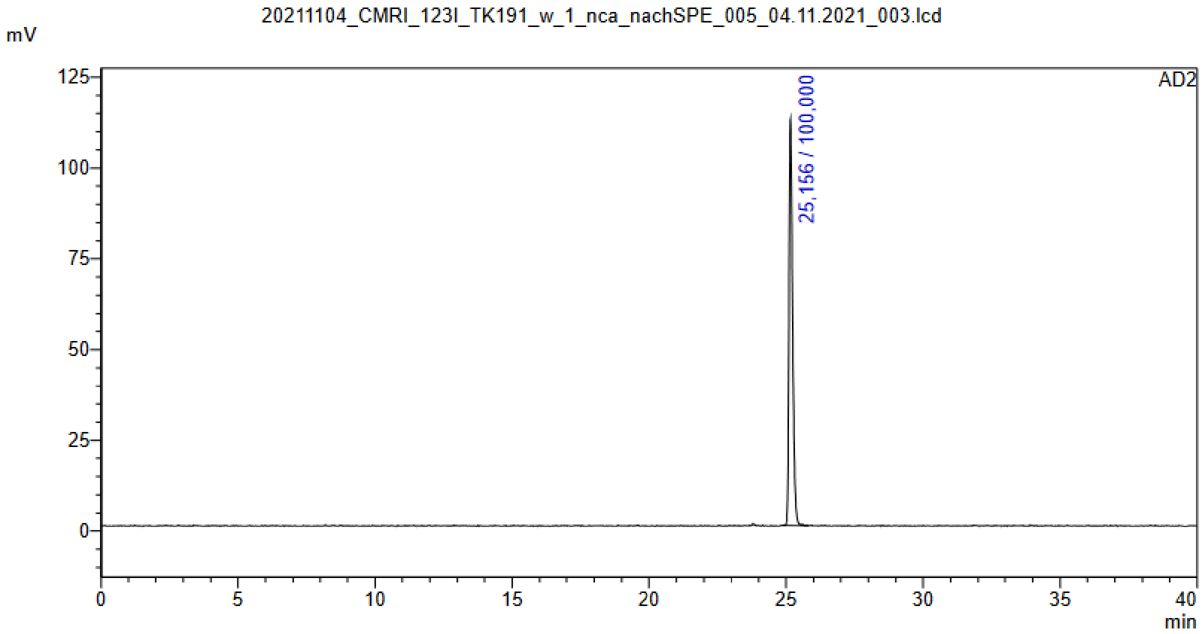

Figure S16: Analytical radio-HPLC chromatogram of **[^{123}I]13** ([γ-Detection], R_t=25.2 min) after SPE with a radiochemical purity over 99%. HPLC-Method (*system* 5, HPLC 25–75).

Analytical radio-HPLC analysis after evaporation at 50 °C (*system* 5, HPLC 25–75) with RCP of 92%:

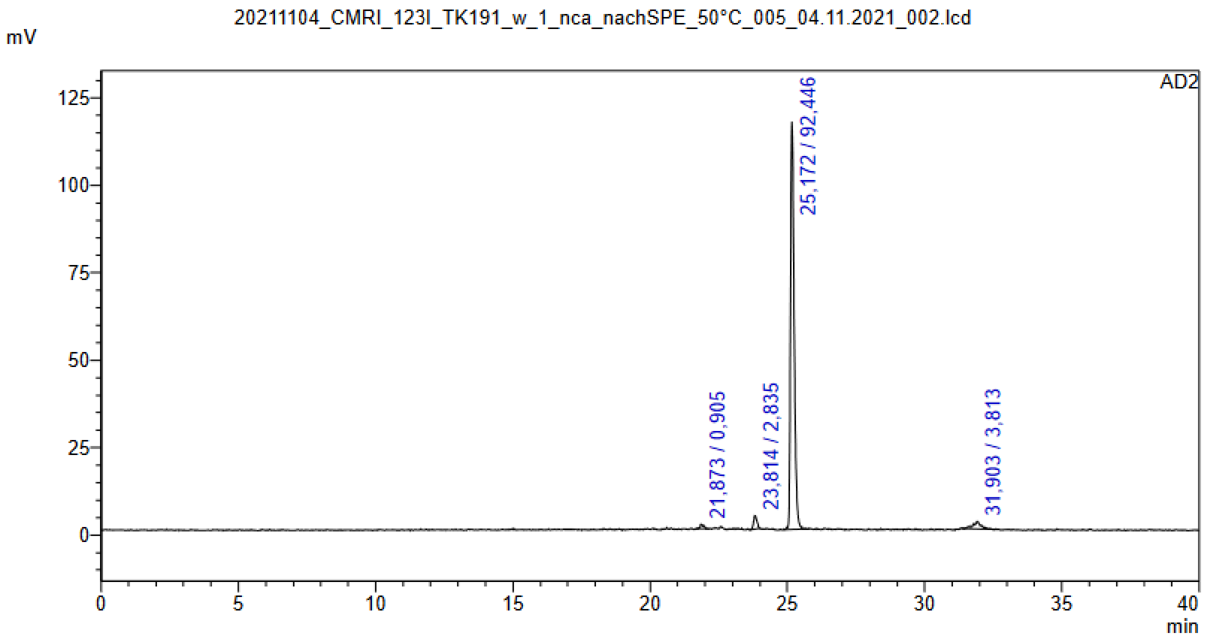

Figure S17: Analytical radio-HPLC chromatogram of **[^{123}I]13** ([γ-Detection], R_t=25.2 min) after an evaporation step at 50 °C with a radiochemical purity of 92% and minor byproducts. HPLC-Method (*system* 5, HPLC 25–75).

Analytical radio-HPLC analysis after evaporation at 70 °C (*system* 5, HPLC 25–75) with RCP of 81%:

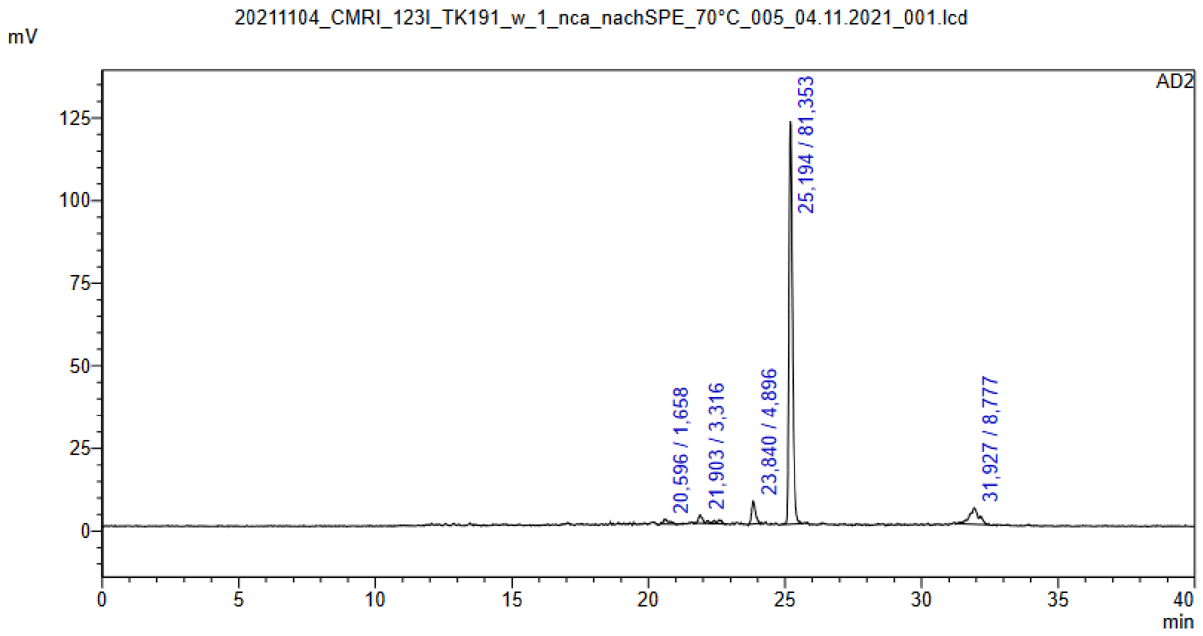

Figure S18: Analytical radio-HPLC chromatogram of **[^{123}I]13** ([γ-Detection], R_t=25.2 min) after an evaporation step at 70 °C with a radiochemical purity of 81% and minor byproducts. HPLC-Method (*system* 5, HPLC 25–75).

3. Confocal laser scanning microscopy – mitochondrial colocalization experiments

Confocal laser scanning microscopy was performed using PC-3 cells. The cells were cultured in Glutamax™ high glucose Dulbecco's Modified Eagle's Medium (DMEM; Gibco™, purchased from Thermo Fisher Scientific, Massachusetts, USA) supplemented in 10% fetal bovine serum (FBS; F7524, Sigma-Aldrich Corporation, St. Louis, USA) and penicillin/streptomycin (Biochrom A2213, 10,000 U/mL) at 37 °C in humidified air containing 5% CO_2. A day prior to the confocal laser scanning experiments the cells were seeded onto an 8-well chamber slide (µ-Slide 8 Well Plates™, ibidi®; Gräfelfing, Germany) with $1.3{\cdot}10^5$ cells per well. The next day, the cells were washed once with DMEM followed by a staining process with **10a** and **10b** (1 µM in DMEM, additive: 0.1% DMSO) for 30 minutes at 37 °C and 5% CO_2. Afterwards staining solution was removed gently and the cells were washed with DMEM. Then the cells were stained with Mitotracker Green FM™ (100 nM in DMEM, additive: 0.1% DMSO; Thermo Fisher Scientific, Massachusetts, USA) for 20 minutes at 37 °C and 5% CO_2. After another removal and washing step with DMEM the cell nuclei were stained with Invitrogen™ Hoechst 33342 (trihydrochloride, trihydrate solution in water; 10 mg/mL; 16.2 mM). For this purpose, Hoechst 33342 was diluted for cell experiments in a ratio of 1:20.000 with DMEM, purchased from Thermo Fisher Scientific, Massachusetts, USA) and cells were incubated for 30 minutes at 37 °C and 5% CO_2. The staining solution was removed again and washed with DMEM and then the samples were measured with confocal laser scanning microscopy within one hour. The excitation wavelength for the SiRs was 635 nm (laser power: 5% for SiR **10a** and 6% for **10b**), for the Mitotracker Green FM™ it was 488 nm (laser power: 17%) and for Hoechst 33342 405 nm (laser power: 3%). Images were taken in sequential mode using Kalman average of three scans. The Pearson's correlation coefficients (PCC) were calculated according to the ImageJ plugin JACoP.[22]

[22] C. A. Schneider, W. S. Rasband, K. W. Eliceiri, Nat. Methods 2012, 9, 7, 671–675.

4. Analytical Data

4.1 NMR Spectra

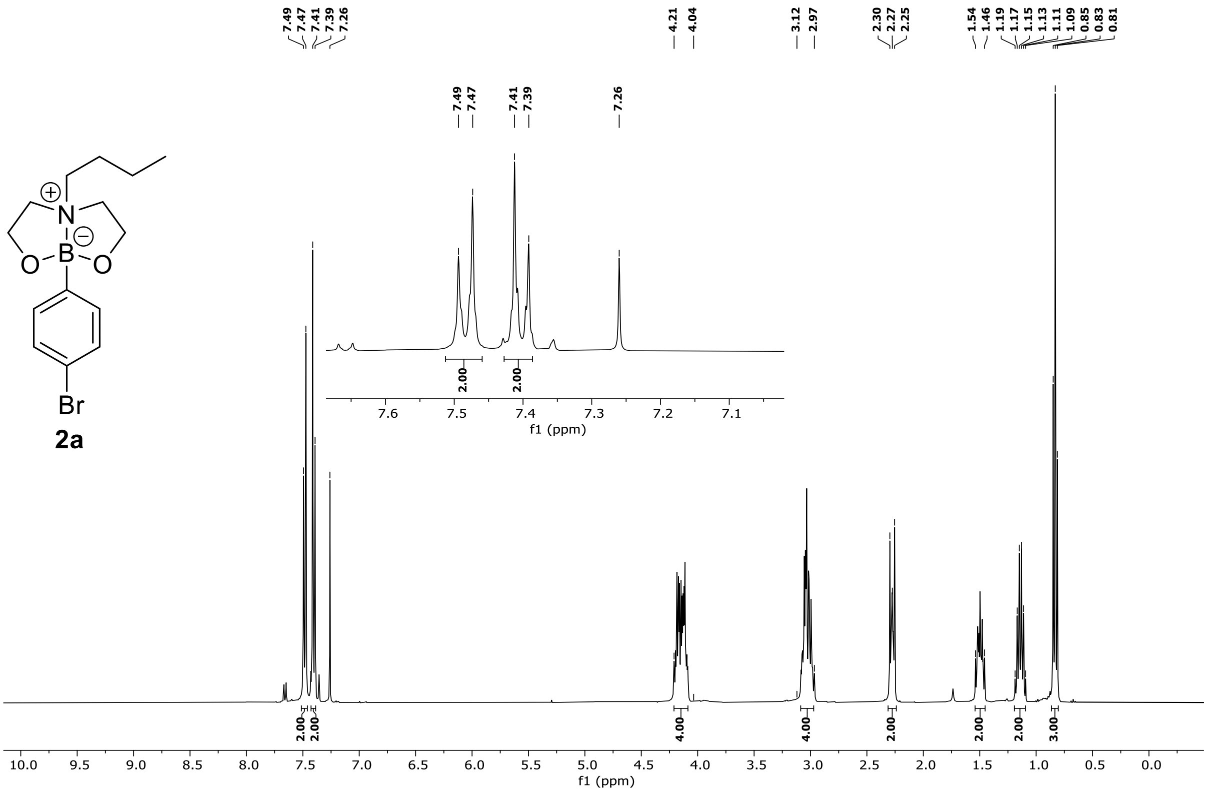

Figure S19: ^{1}H-NMR-spectrum of **2a** in chloroform-*d* (400 MHz, 300 K).

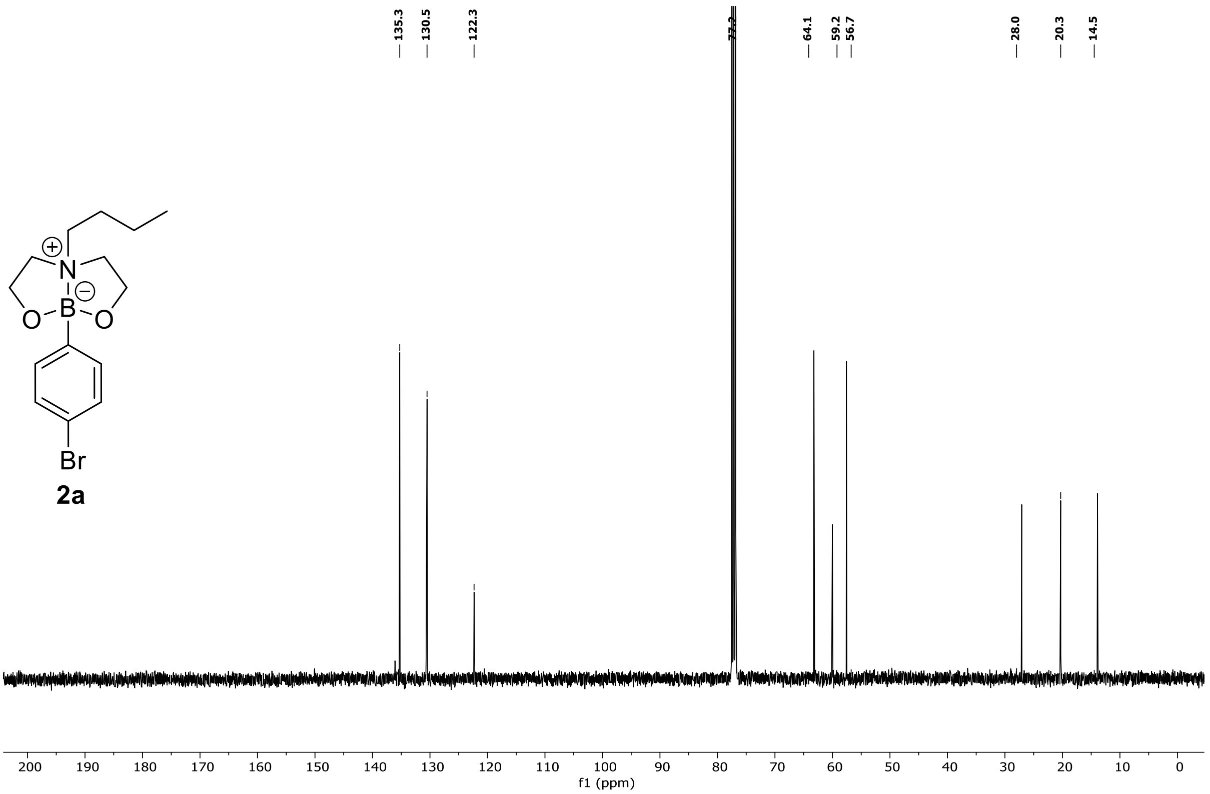

Figure S20: ^{13}C{^{1}H}-NMR-spectrum of **2a** in chloroform-*d* (101 MHz, 300 K).

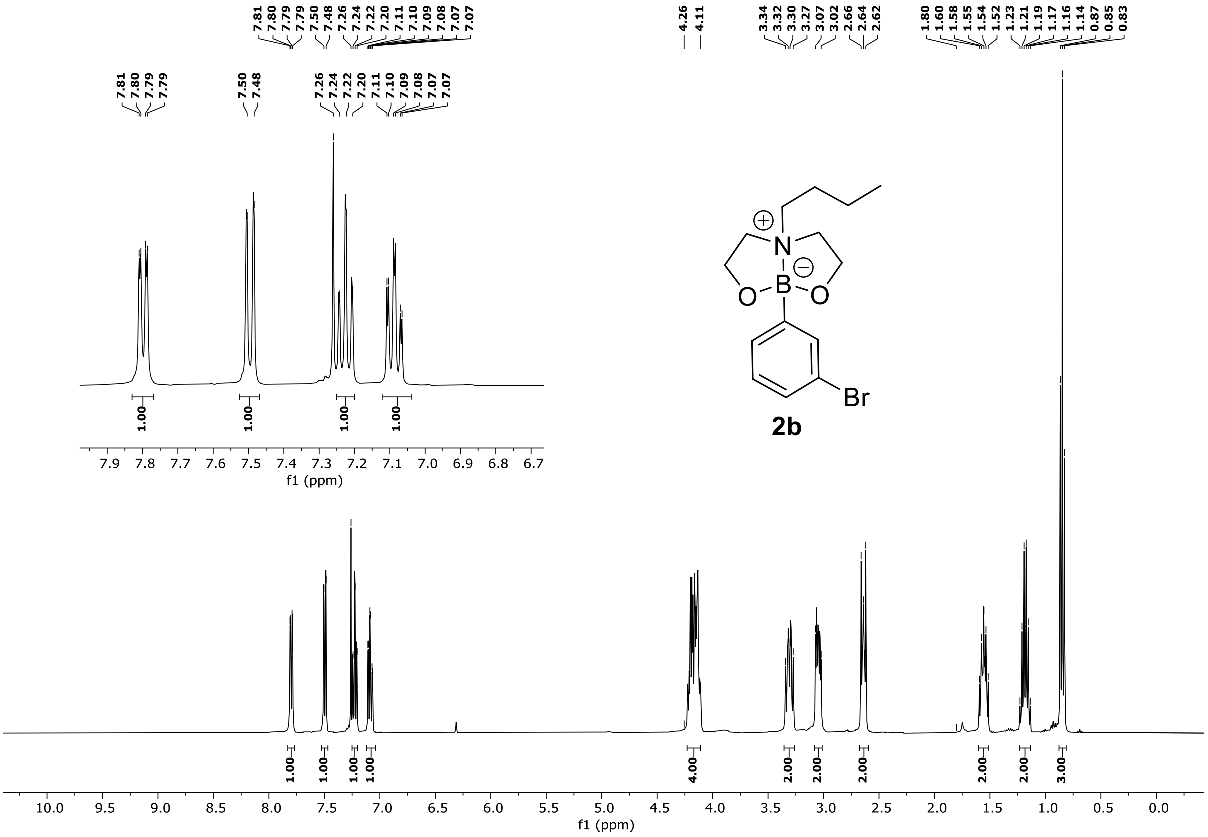

Figure S21: ^{1}H-NMR-spectrum of **2b** in chloroform-*d* (400 MHz, 300 K).

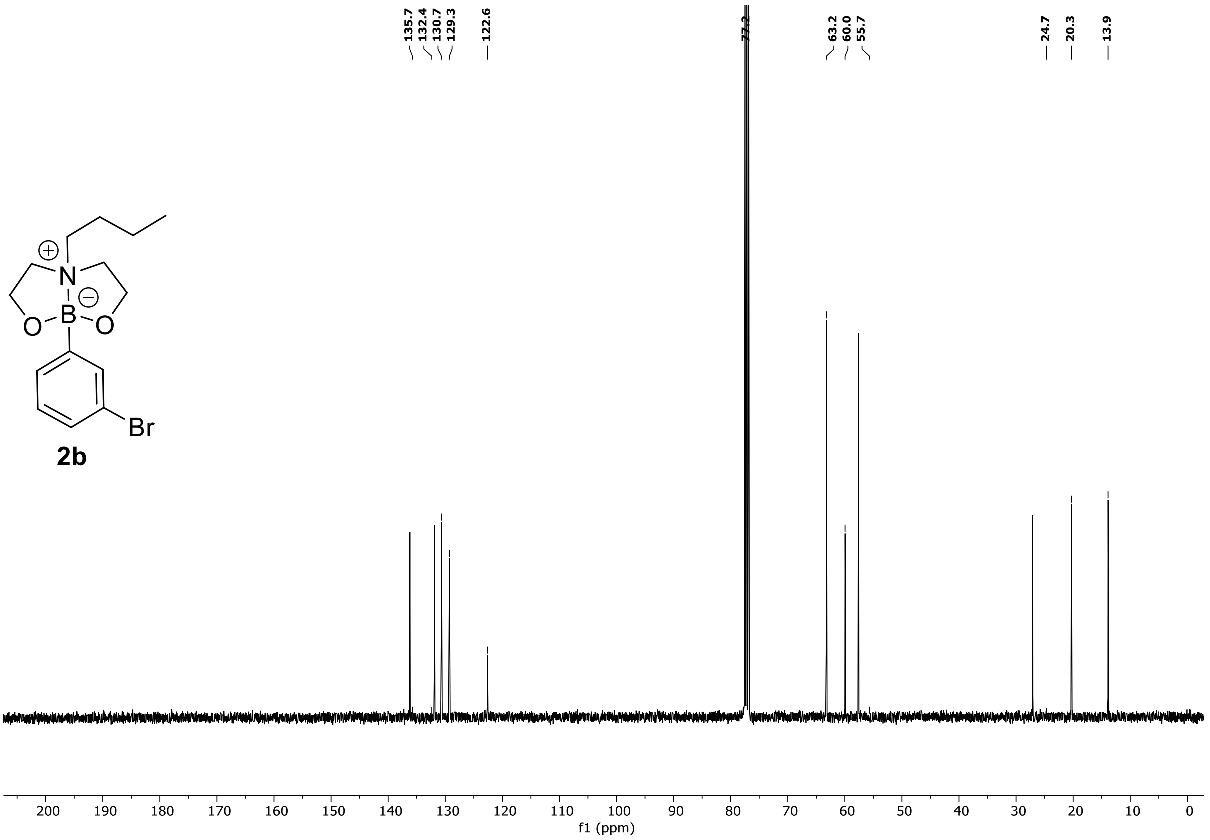

Figure S22: ^{13}C{^{1}H}-NMR-spectrum of **2b** in chloroform-*d* (101 MHz, 300 K).

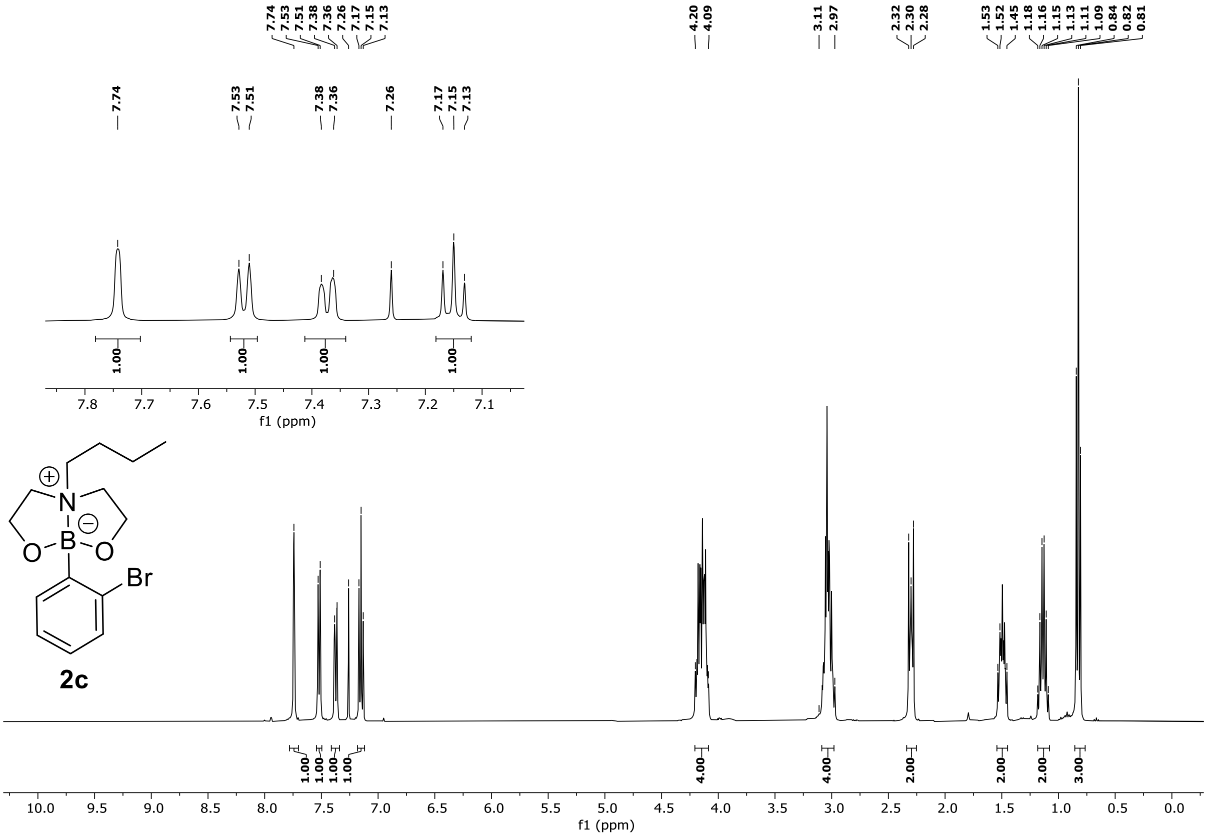

Figure S23: ^{1}H-NMR-spectrum of **2c** in chloroform-*d* (400 MHz, 300 K).

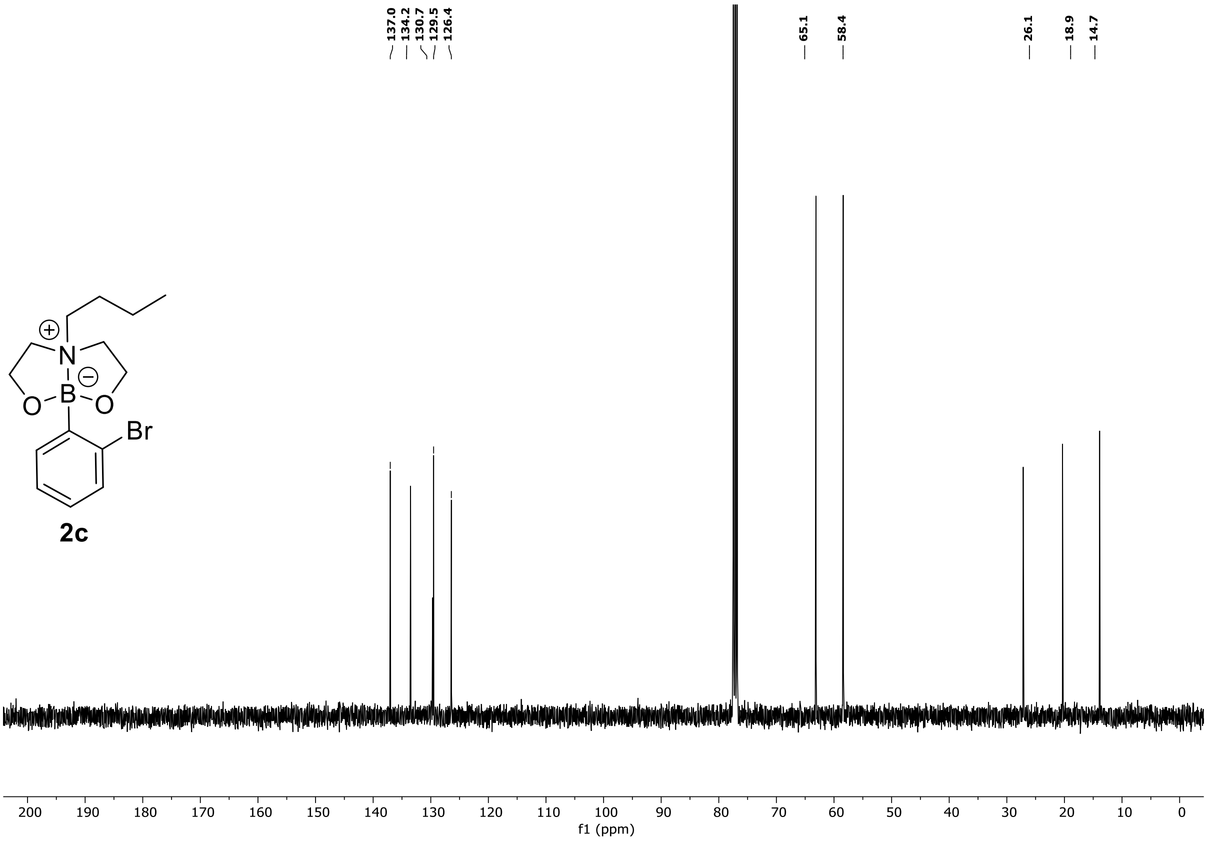

Figure S24: ^{13}C{^{1}H}-NMR-spectrum of **2c** in chloroform-*d* (101 MHz, 300 K).

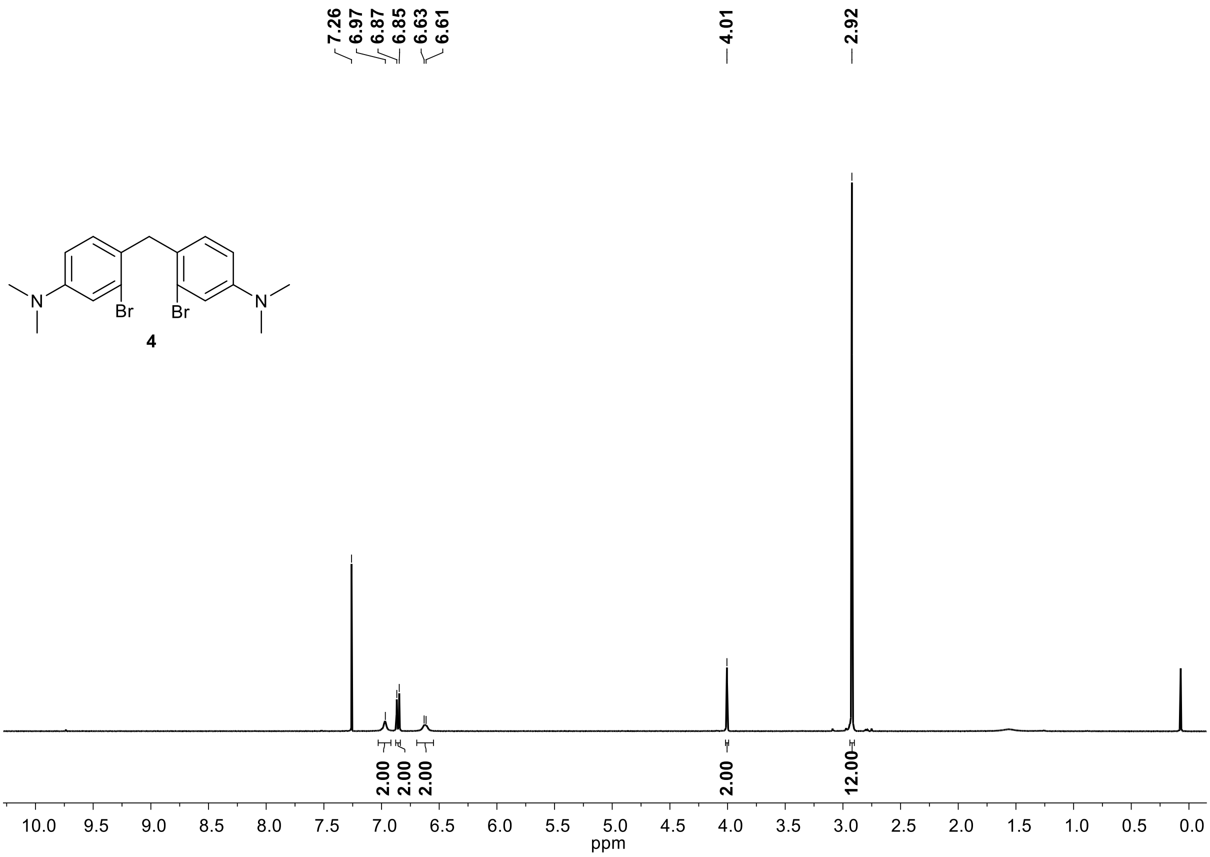

Figure S25: ^{1}H-NMR-spectrum of **4** in chloroform-*d* (400 MHz, 300 K).

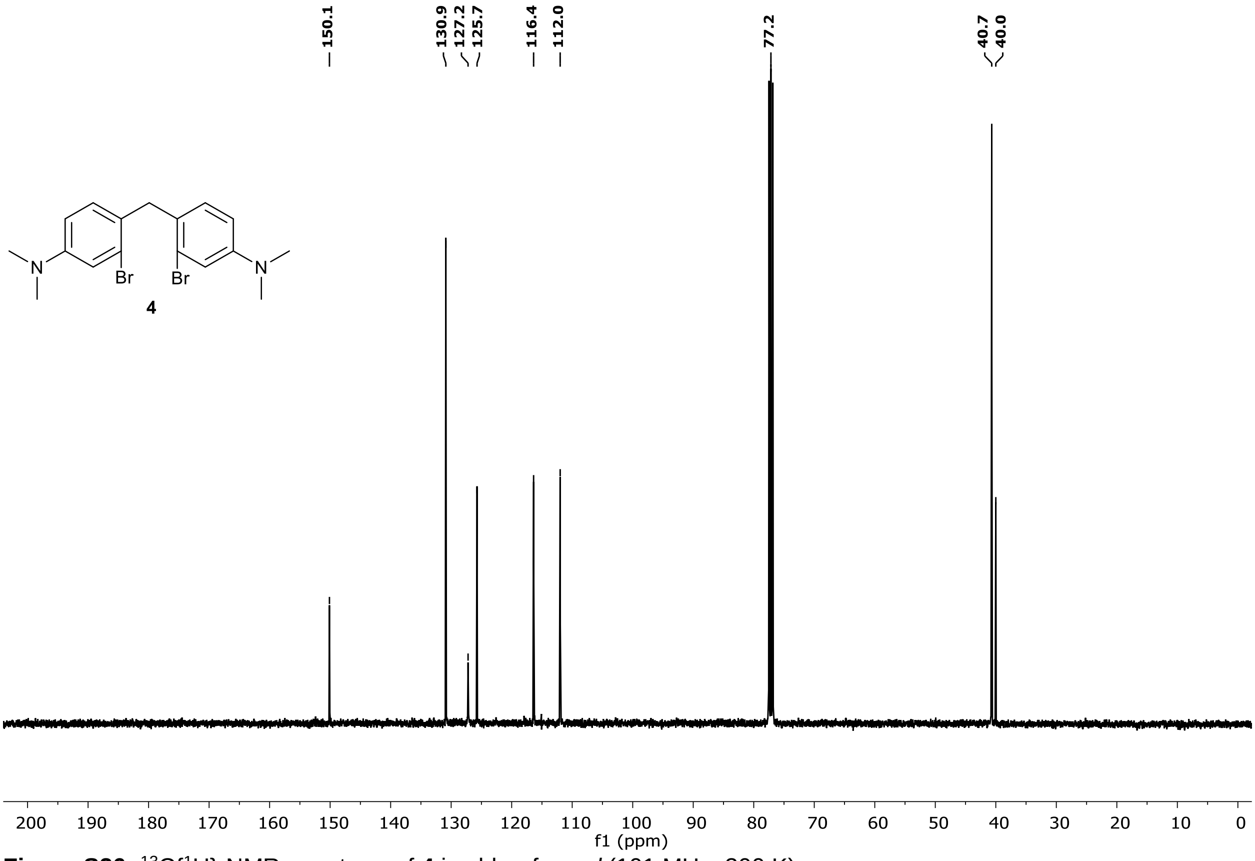

Figure S26: ^{13}C{^{1}H}-NMR-spectrum of **4** in chloroform-*d* (101 MHz, 300 K).

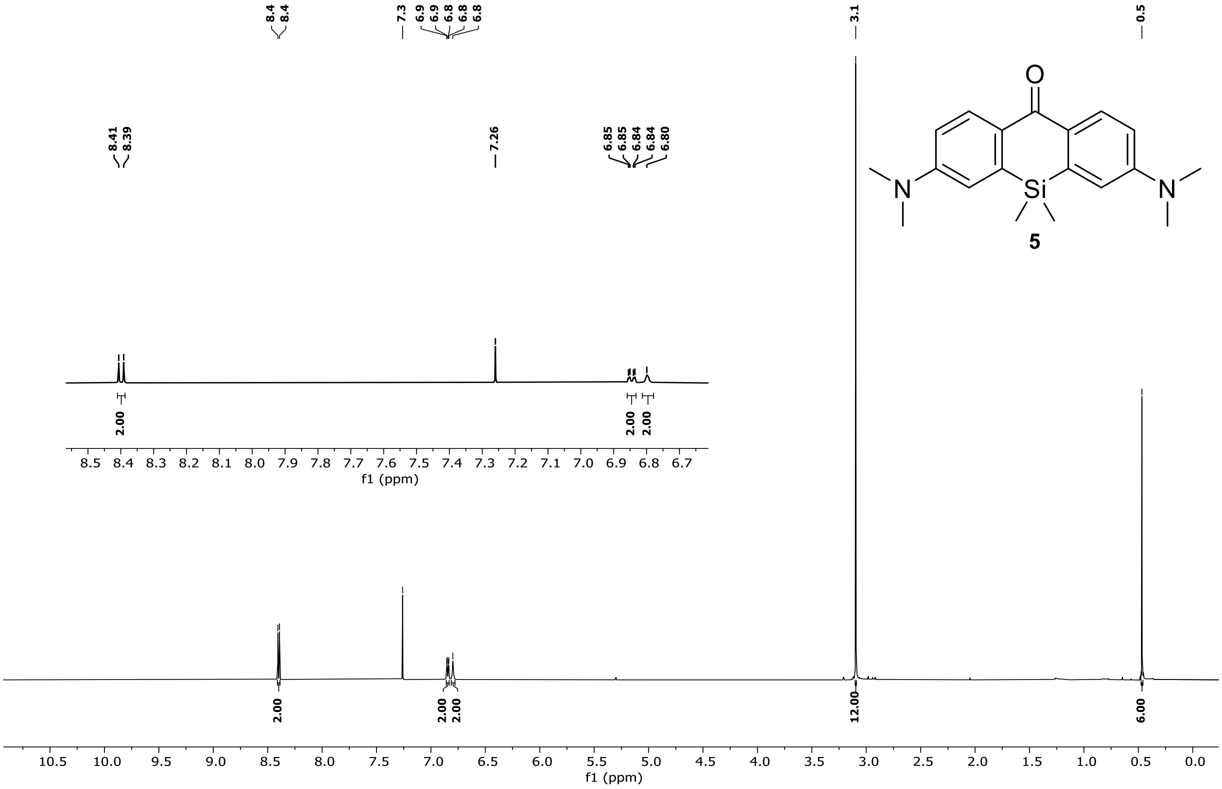

Figure S27: ^{1}H-NMR-spectrum of **5** in chloroform-*d* (400 MHz, 300 K).

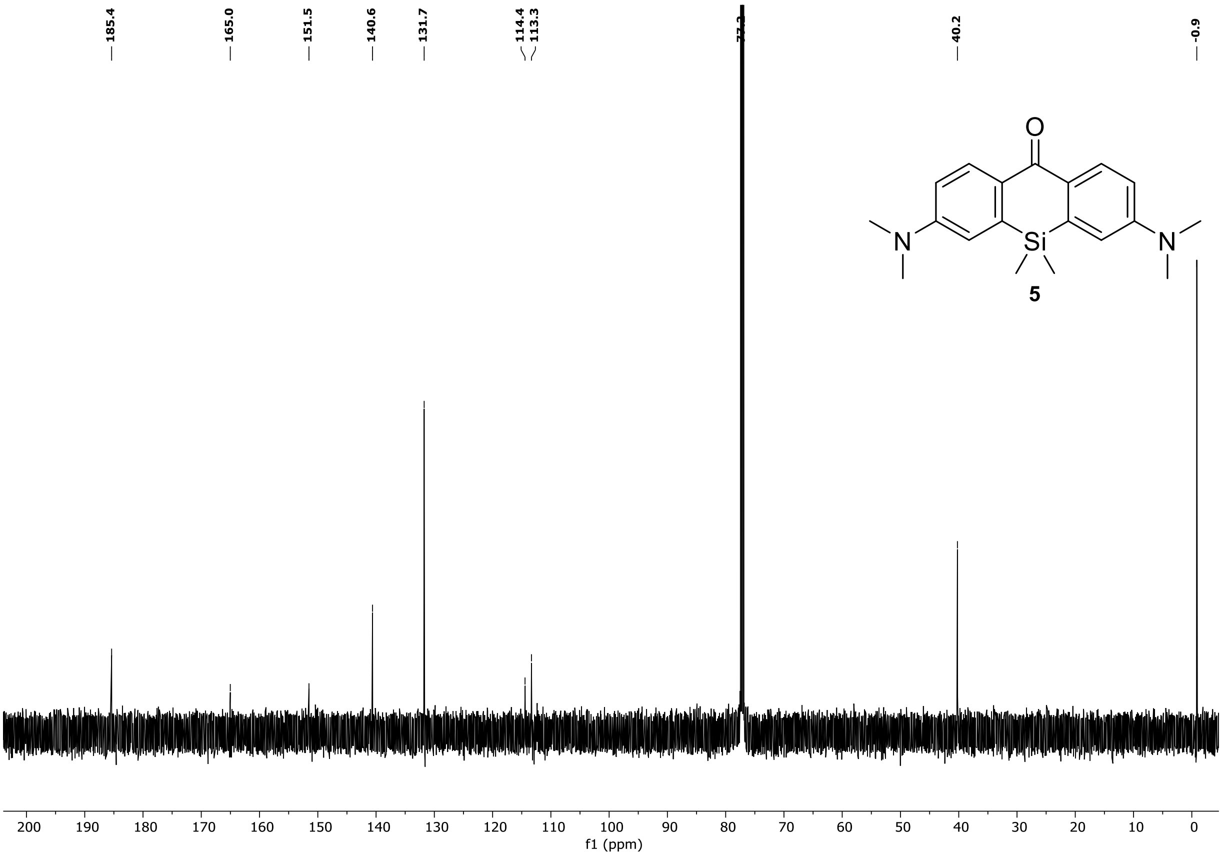

Figure S28: ^{13}C{^{1}H}-NMR-spectrum of **5** in chloroform-*d* (151 MHz, 300 K).

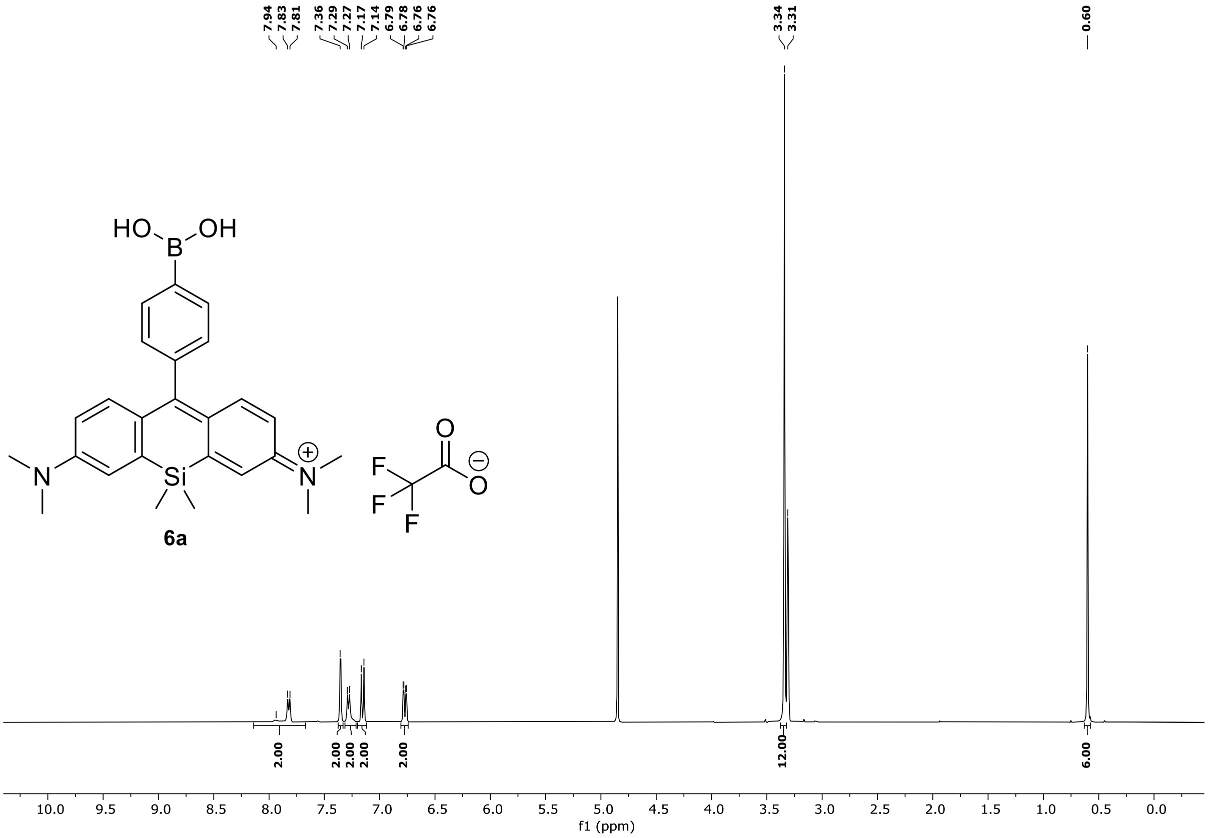

Figure S29: ^{1}H-NMR-spectrum of **6a** in methanol-d_4 (400 MHz, 300 K).

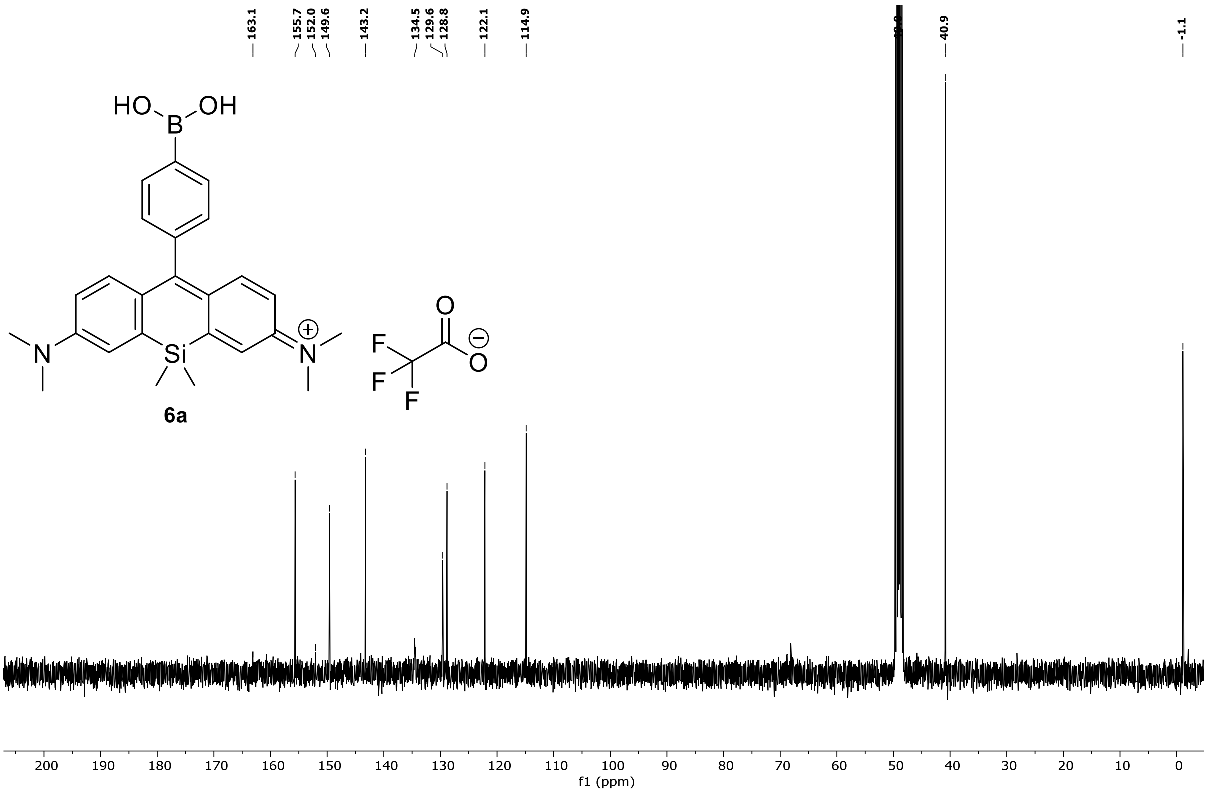

Figure S30: ^{13}C{^{1}H}-NMR-spectrum of **6a** in methanol-d_4 (101 MHz, 300 K).

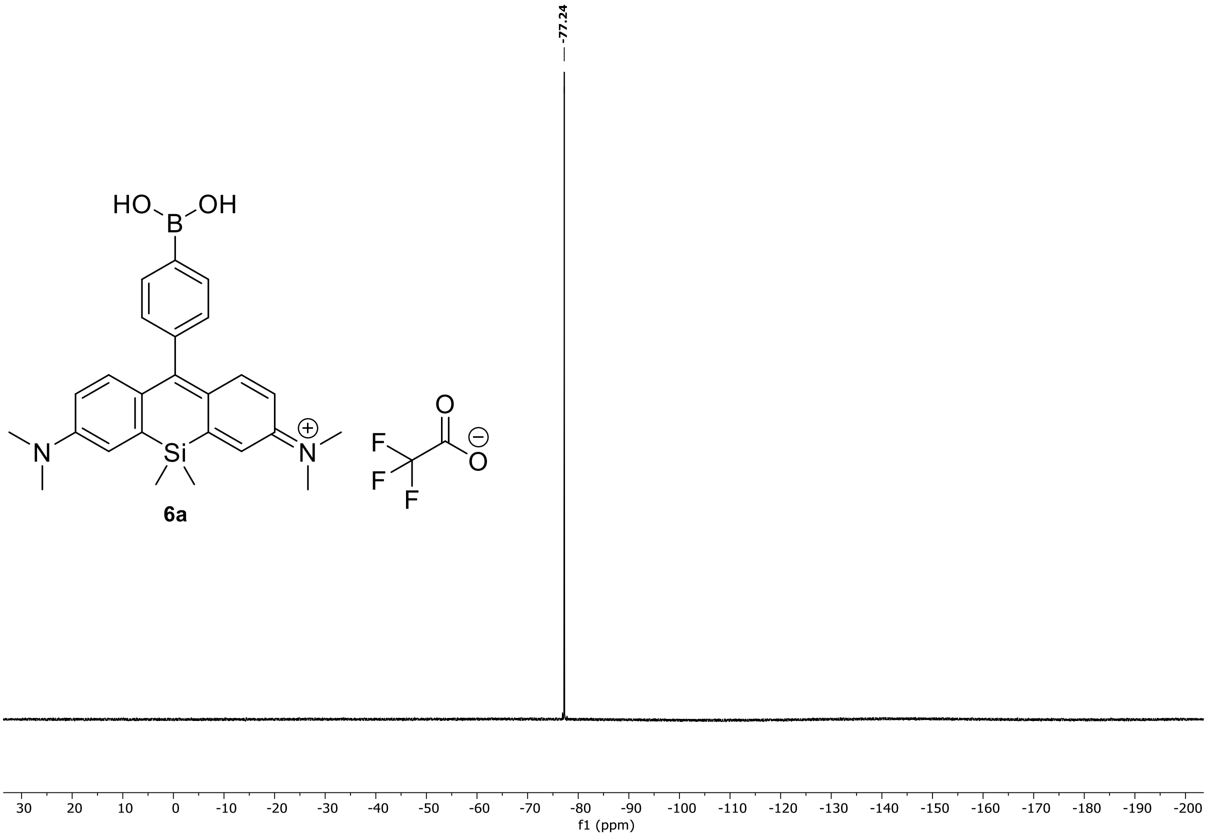

Figure S31: ^{19}F-NMR-spectrum of **6a** in methanol-d_4 (376 MHz, 300 K).

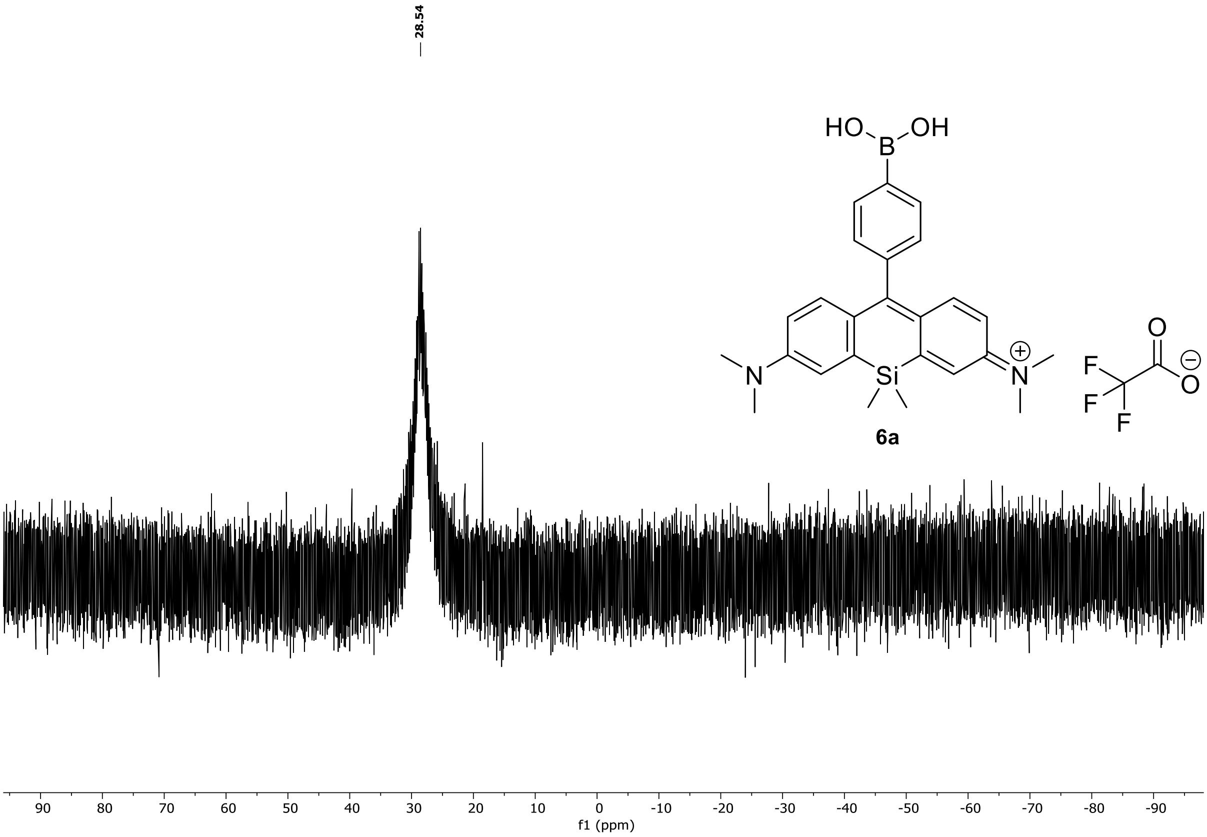

Figure S32: ^{11}B-NMR-spectrum of **6a** in methanol-d_4 (192 MHz, 300 K).

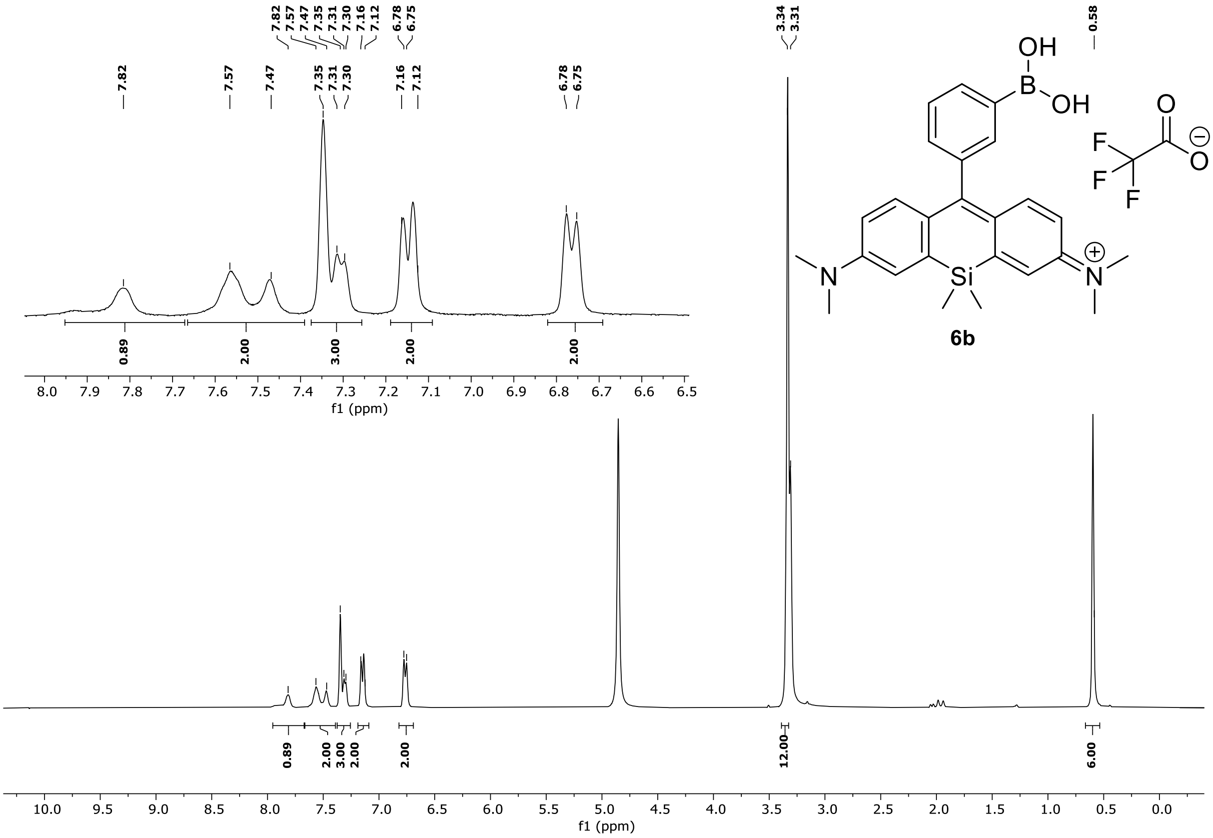

Figure S33: ^{1}H-NMR-spectrum of **6b** in methanol-d_4 (400 MHz, 300 K).

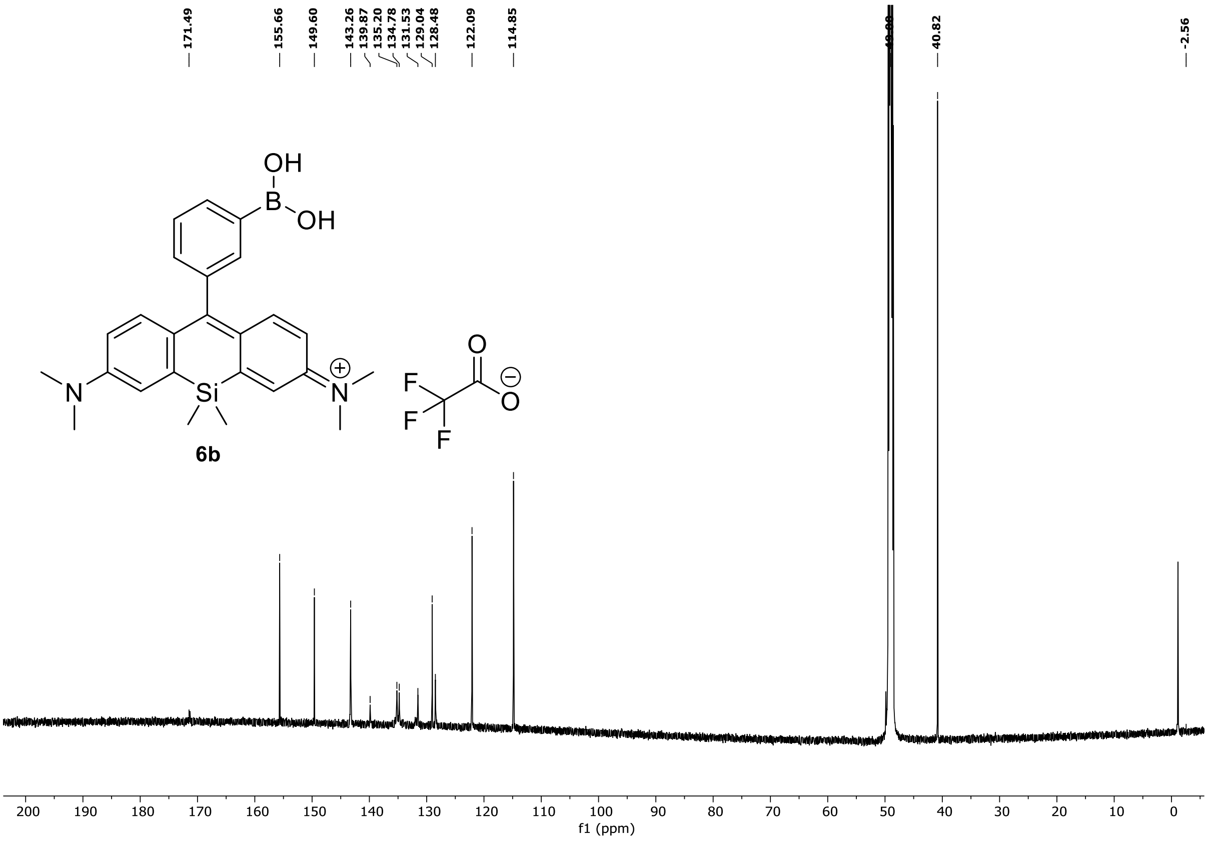

Figure S34: ^{13}C{^{1}H}-NMR-spectrum of **6b** in methanol-d_4 (151 MHz, 300 K).

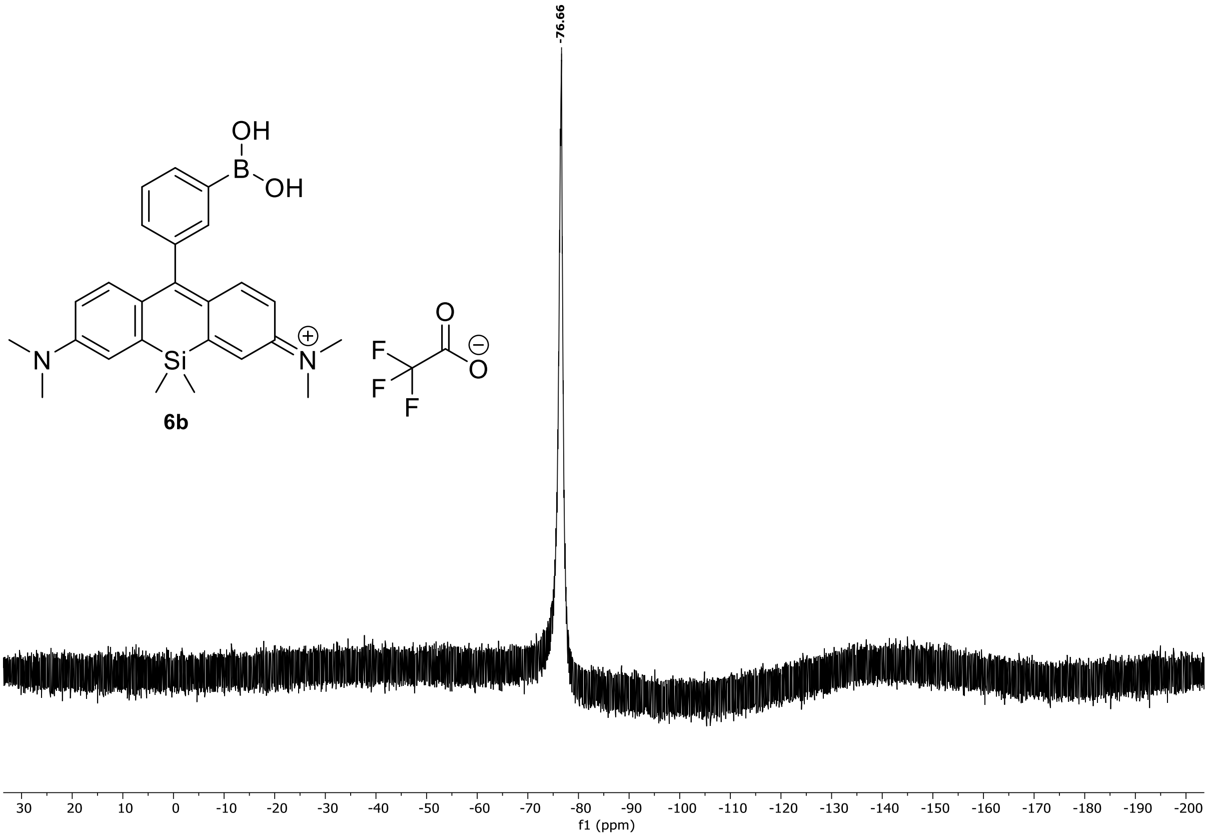

Figure S35: ^{19}F-NMR-spectrum of **6b** in methanol-d_4 (376 MHz, 300 K).

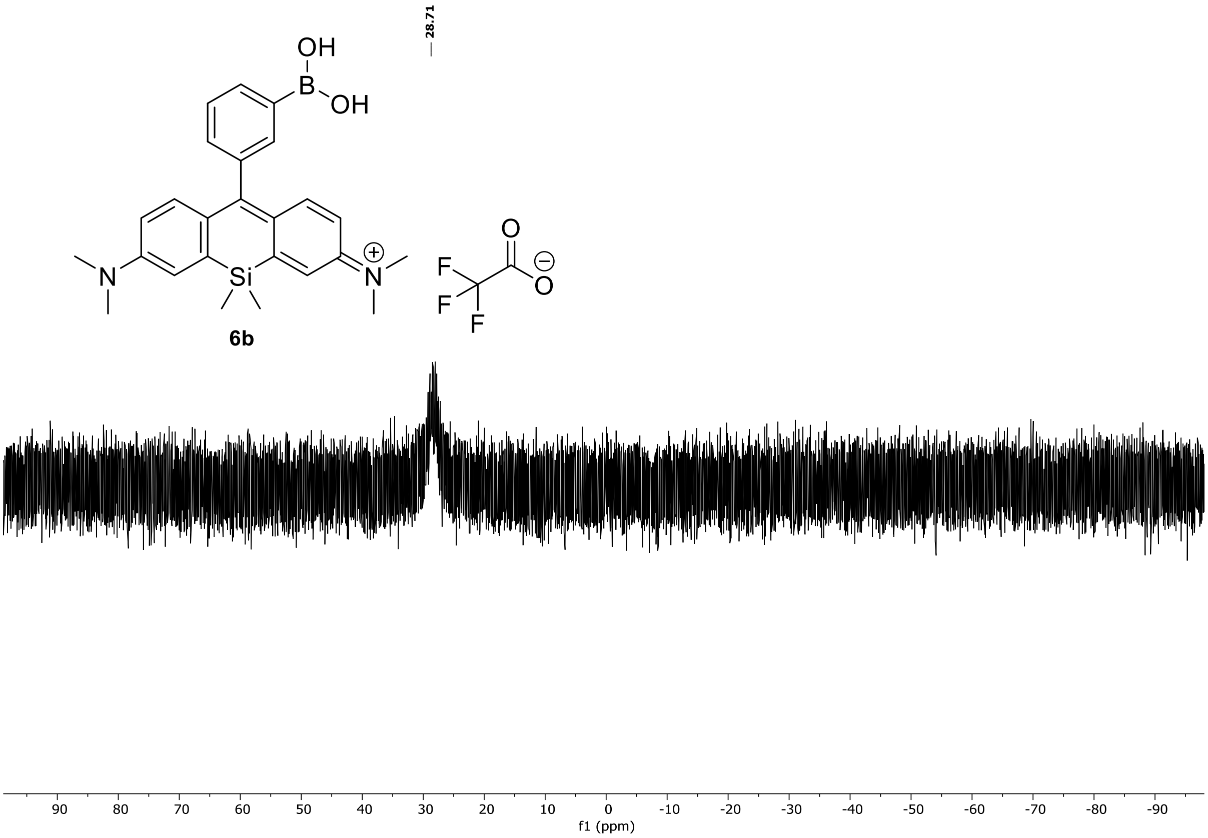

Figure S36: ^{11}B-NMR-spectrum of **6b** in methanol-d_4 (192 MHz, 300 K).

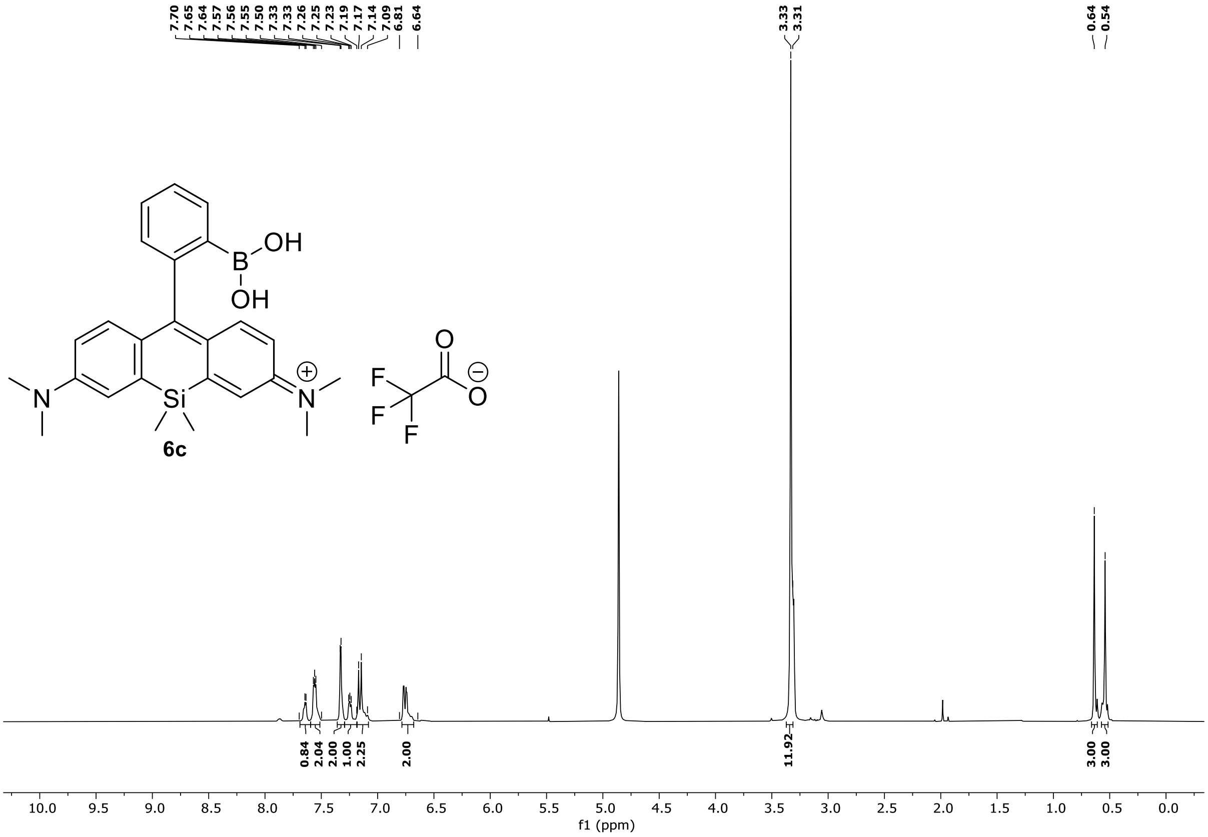

Figure S37: ^{1}H-NMR-spectrum of **6c** in methanol-d_4 (400 MHz, 300 K).

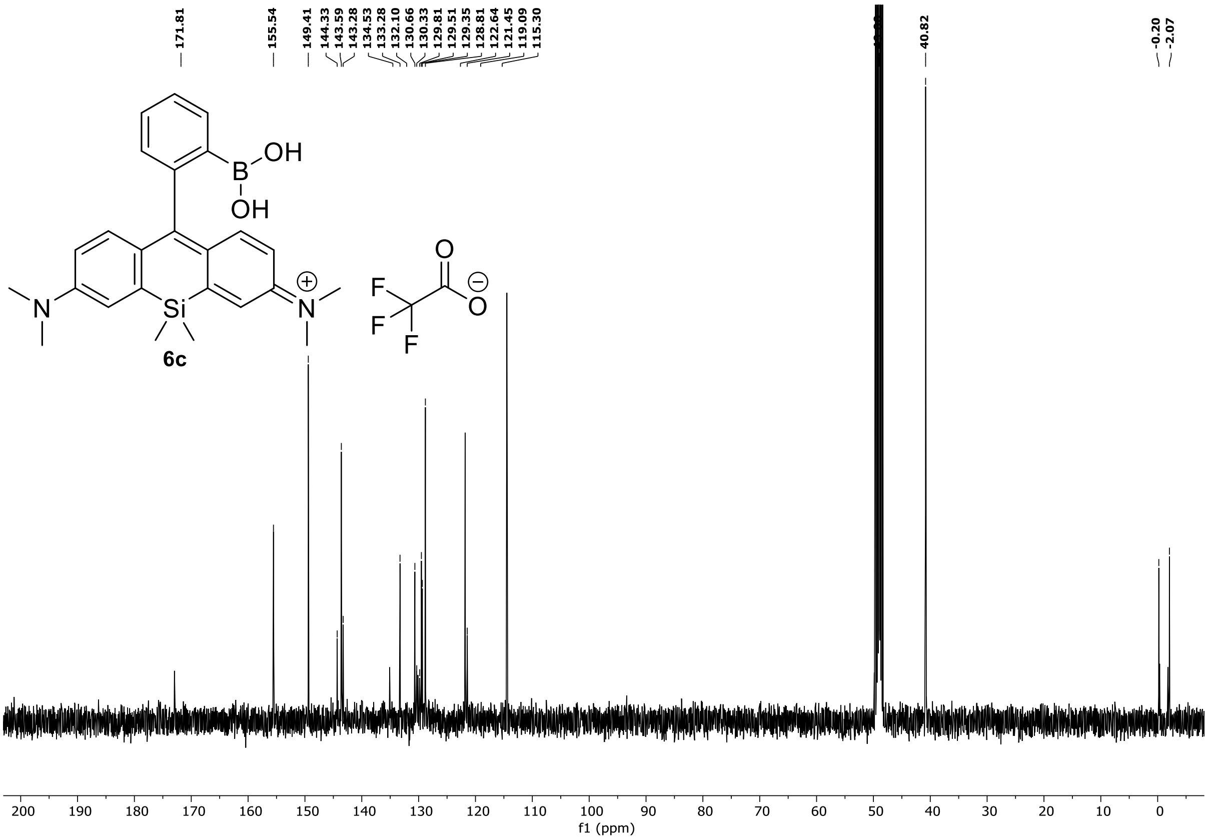

Figure S38: ^{13}C{^{1}H}-NMR-spectrum of **6c** in methanol-d_4 (101 MHz, 300 K).

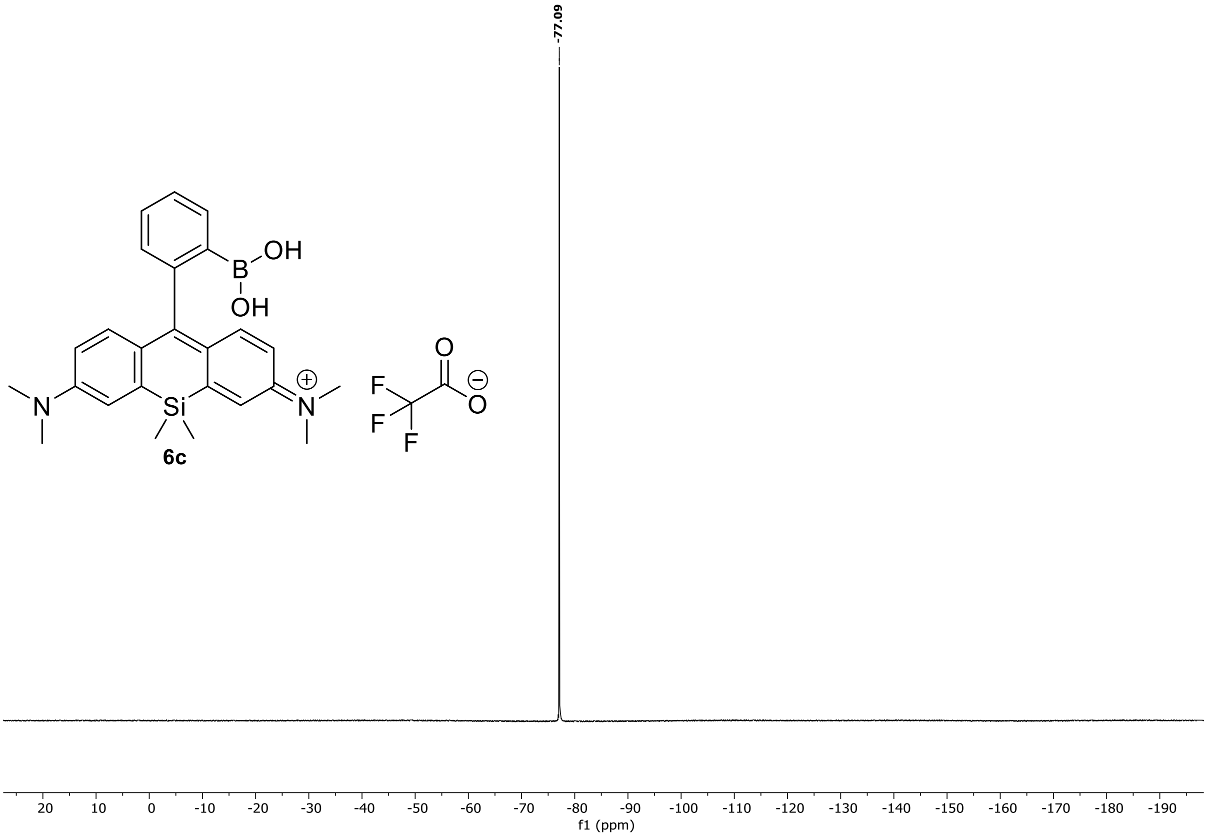

Figure S39: ^{19}F-NMR-spectrum of **6c** in methanol-d_4 (564 MHz, 300 K).

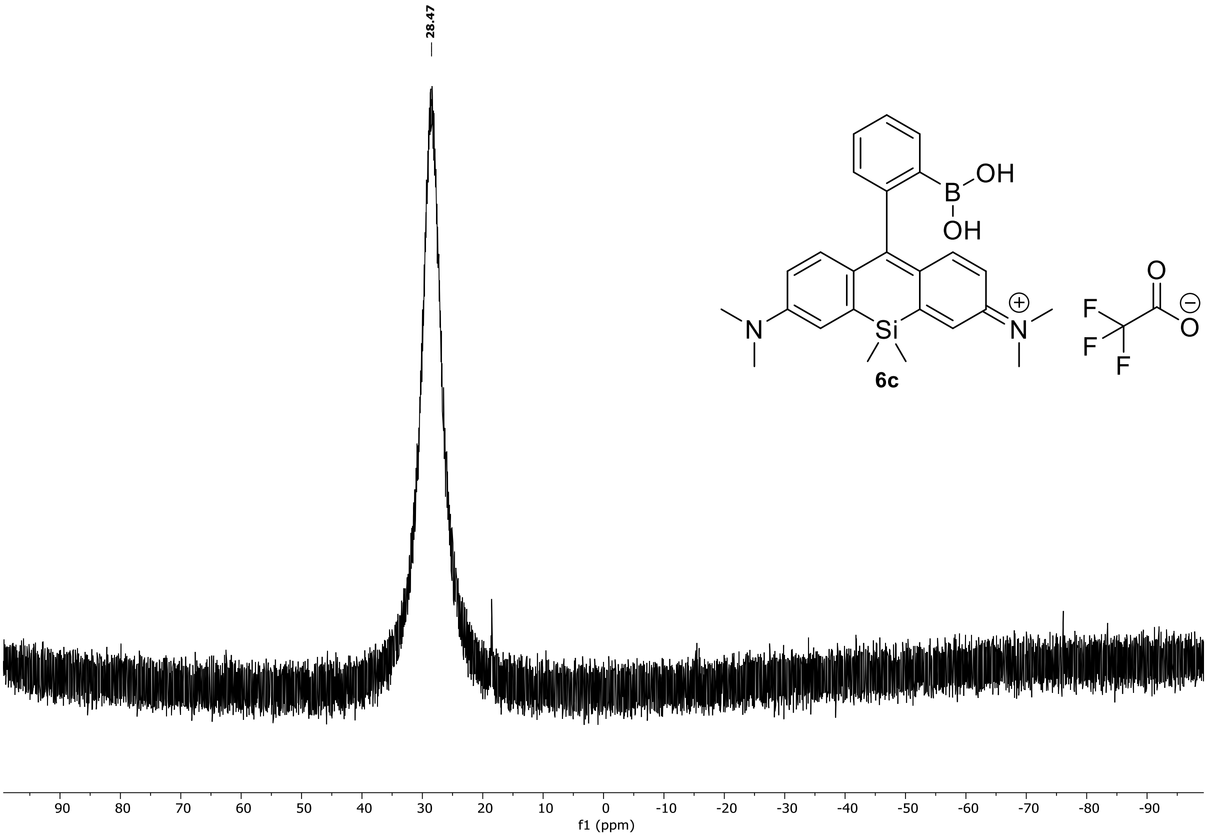

Figure S40: ^{11}B-NMR-spectrum of **6c** in methanol-d_4 (192 MHz, 300 K).

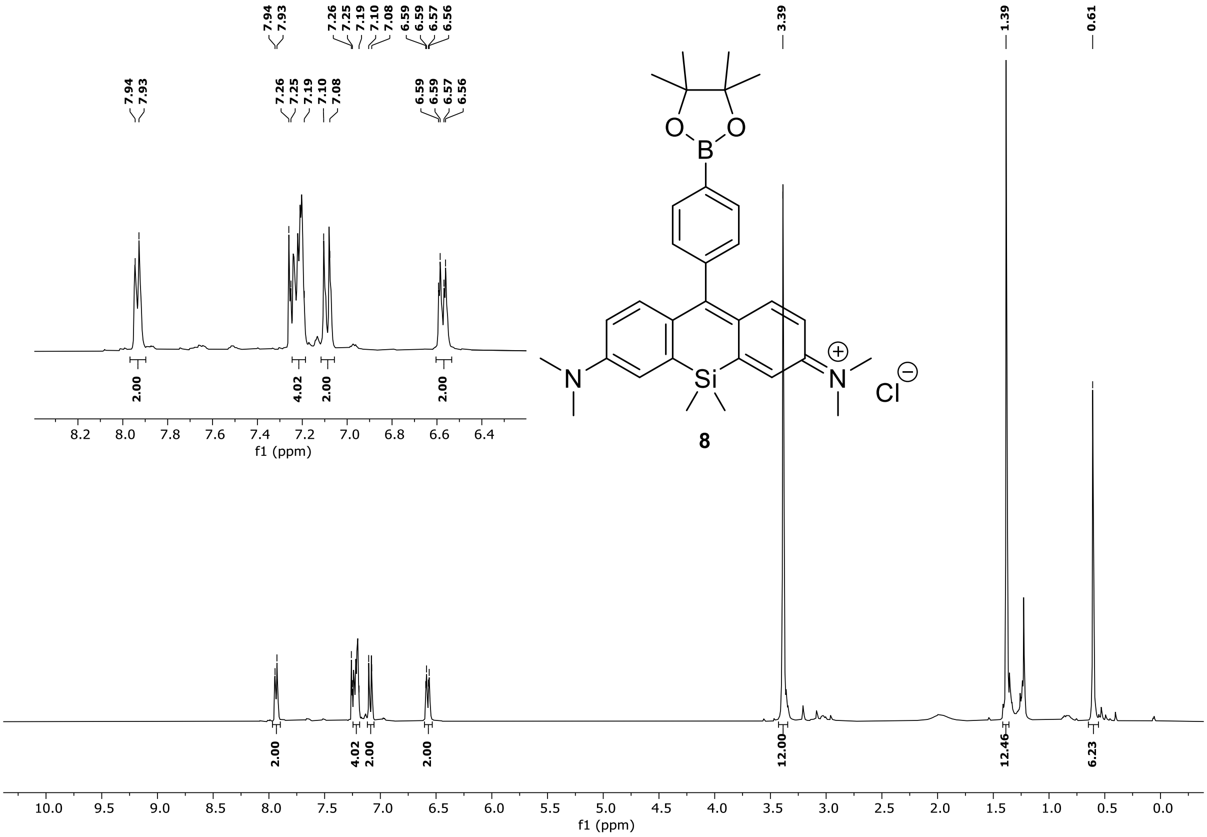

Figure S41: ^{1}H-NMR-spectrum of **8** in chloroform-*d* (400 MHz, 300 K).

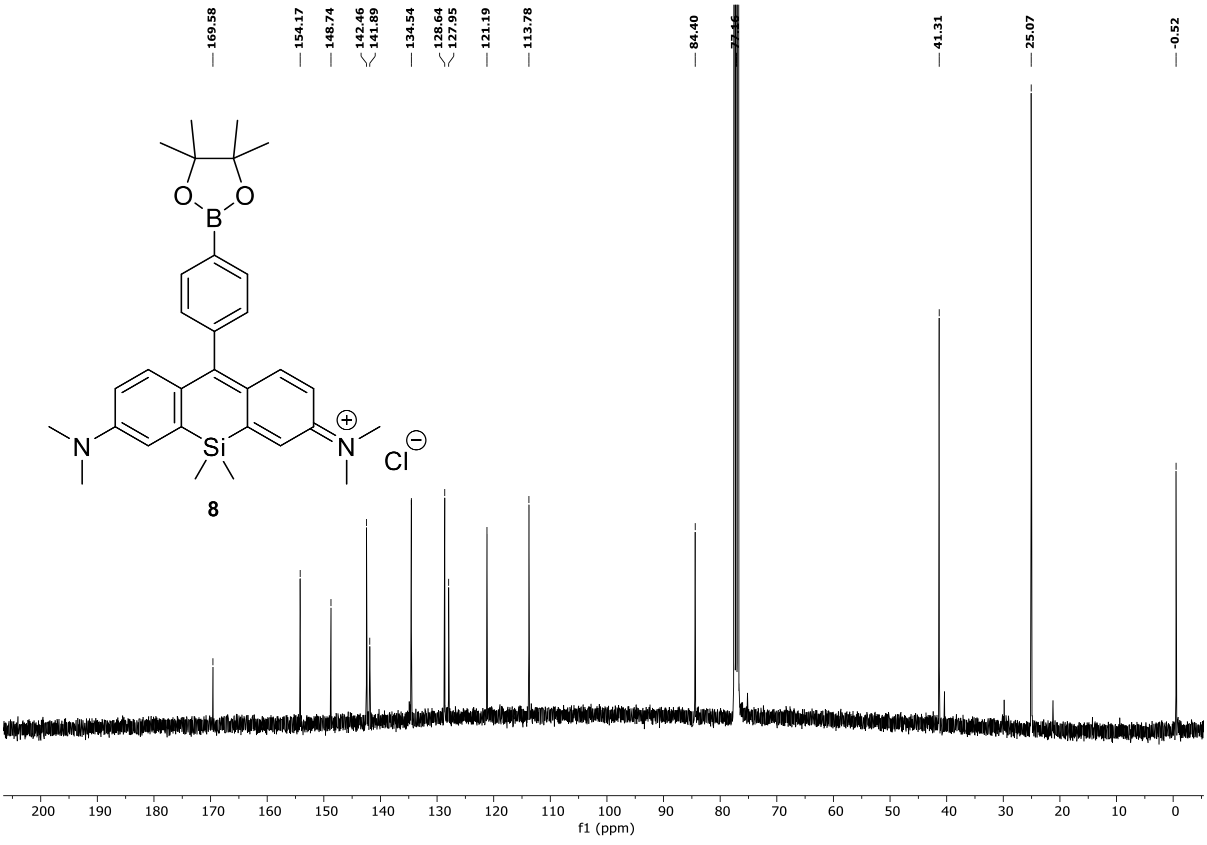

Figure S42: ^{13}C{^{1}H}-NMR-spectrum of **8** in chloroform-*d* (151 MHz, 300 K).

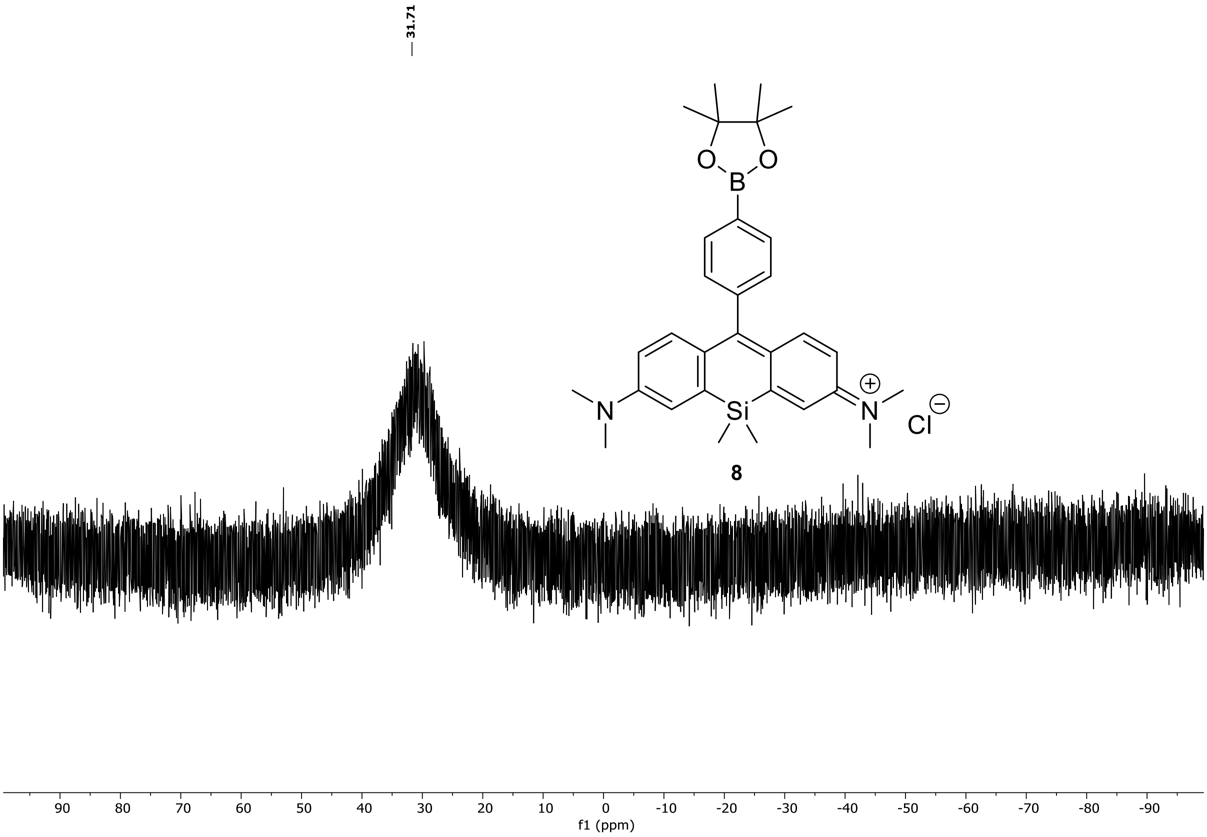

Figure S43: ^{11}B-NMR-spectrum of **8** in chloroform-*d* (128 MHz, 300 K).

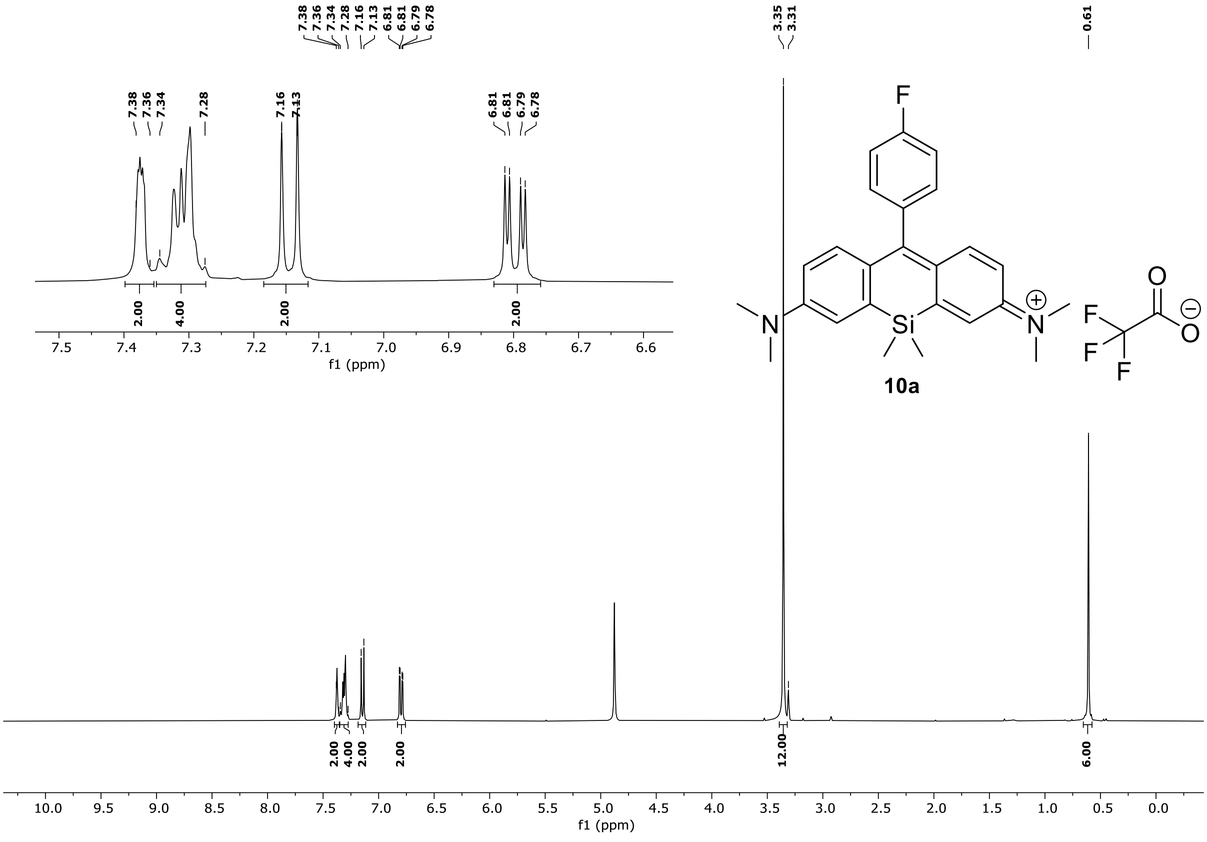

Figure S44: ^{1}H-NMR-spectrum of **10a** in methanol-d_4 (400 MHz, 300 K).

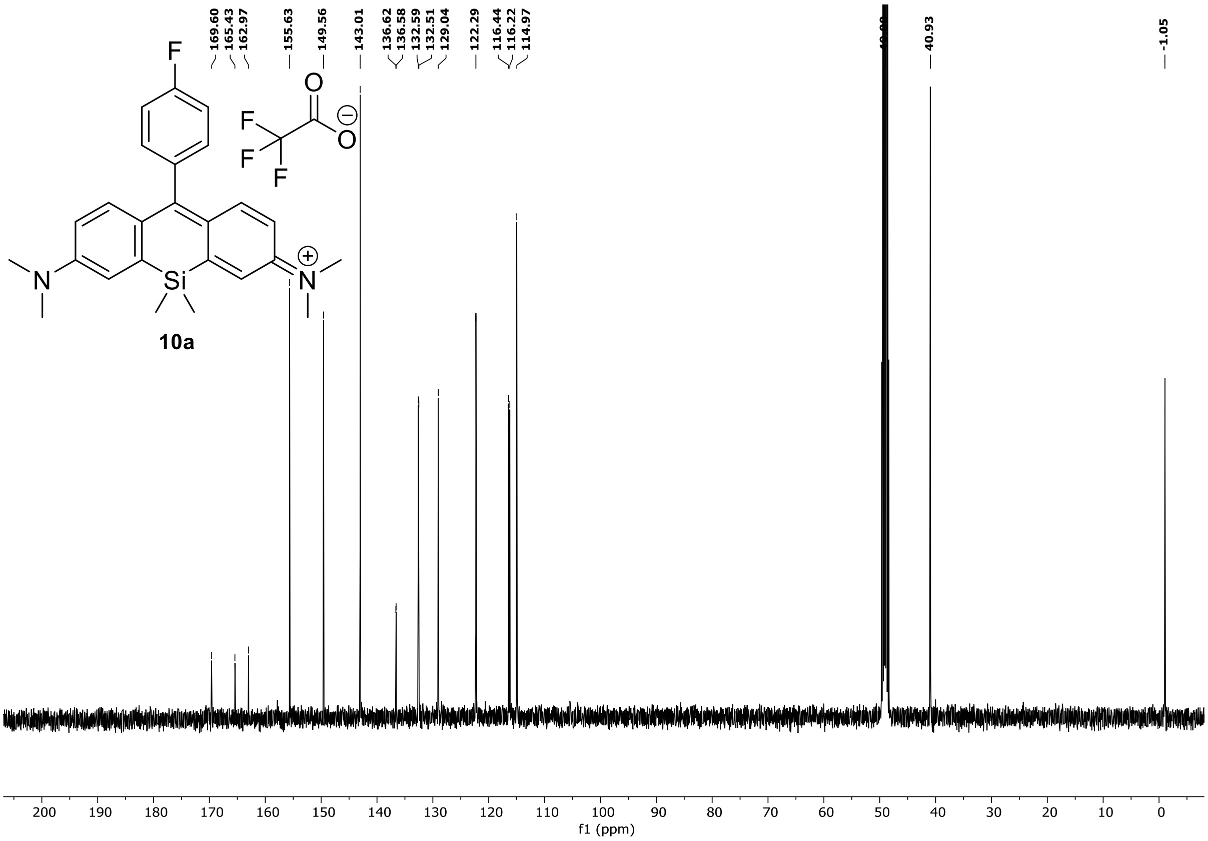

Figure S45: $^{13}C\{^{1}H\}$-NMR-spectrum of **10a** in methanol-*d4* (101 MHz, 300 K).

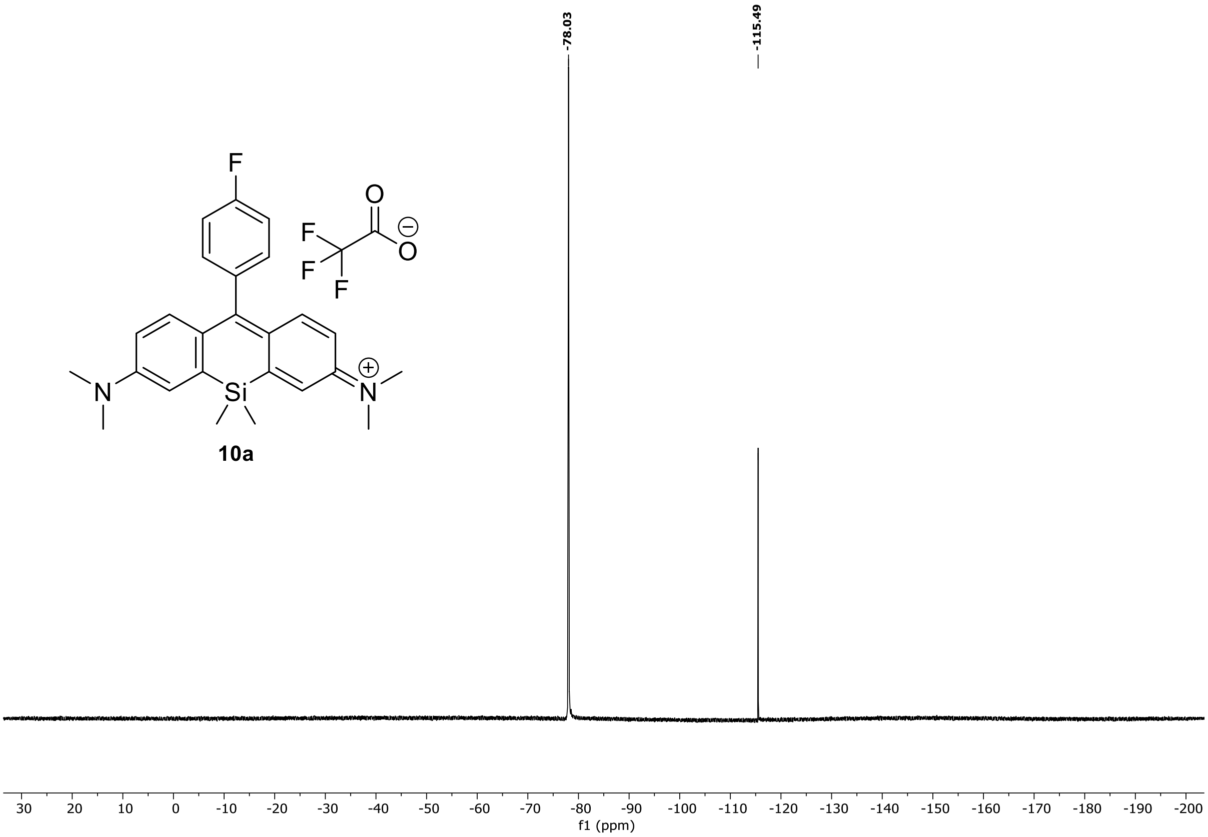

Figure S46: ^{19}F-NMR-spectrum of **10a** in methanol-*d4* (376 MHz, 300 K).

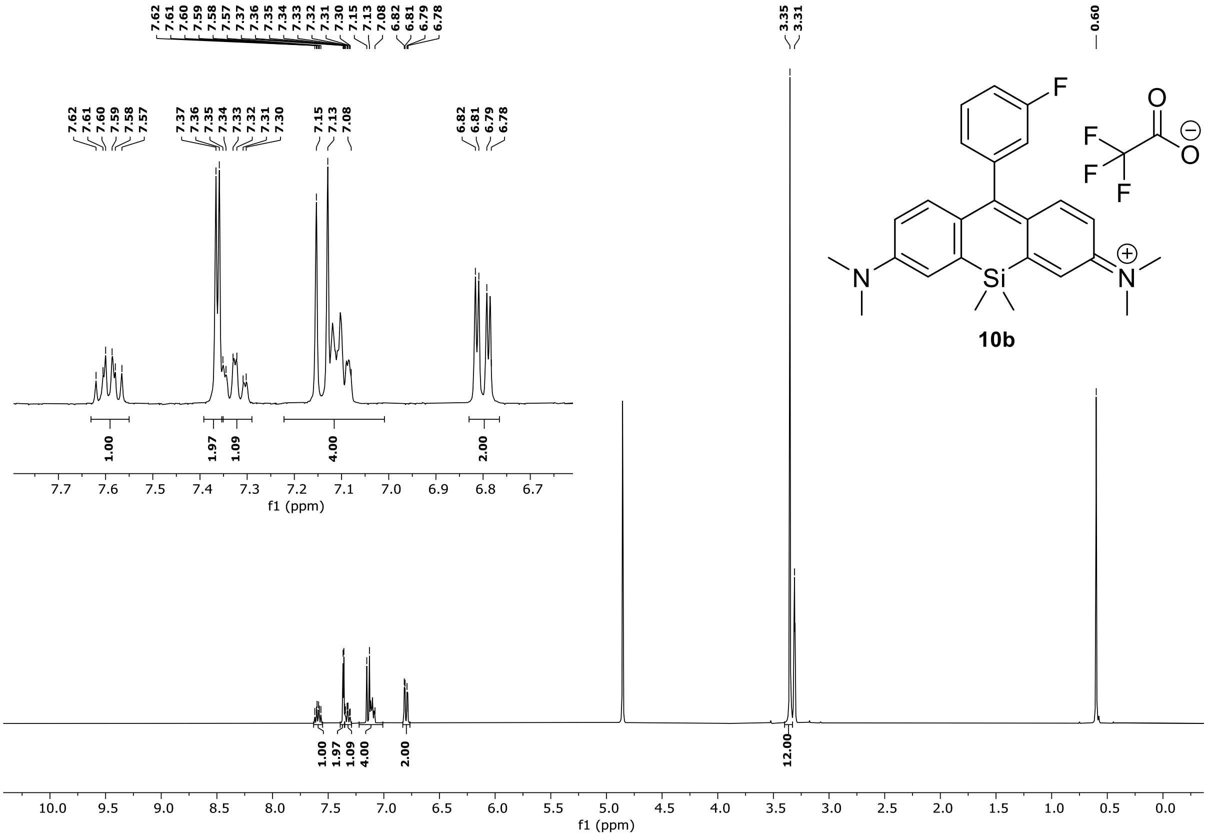

Figure S47: ^{1}H-NMR-spectrum of **10b** in methanol-d_4 (400 MHz, 300 K).

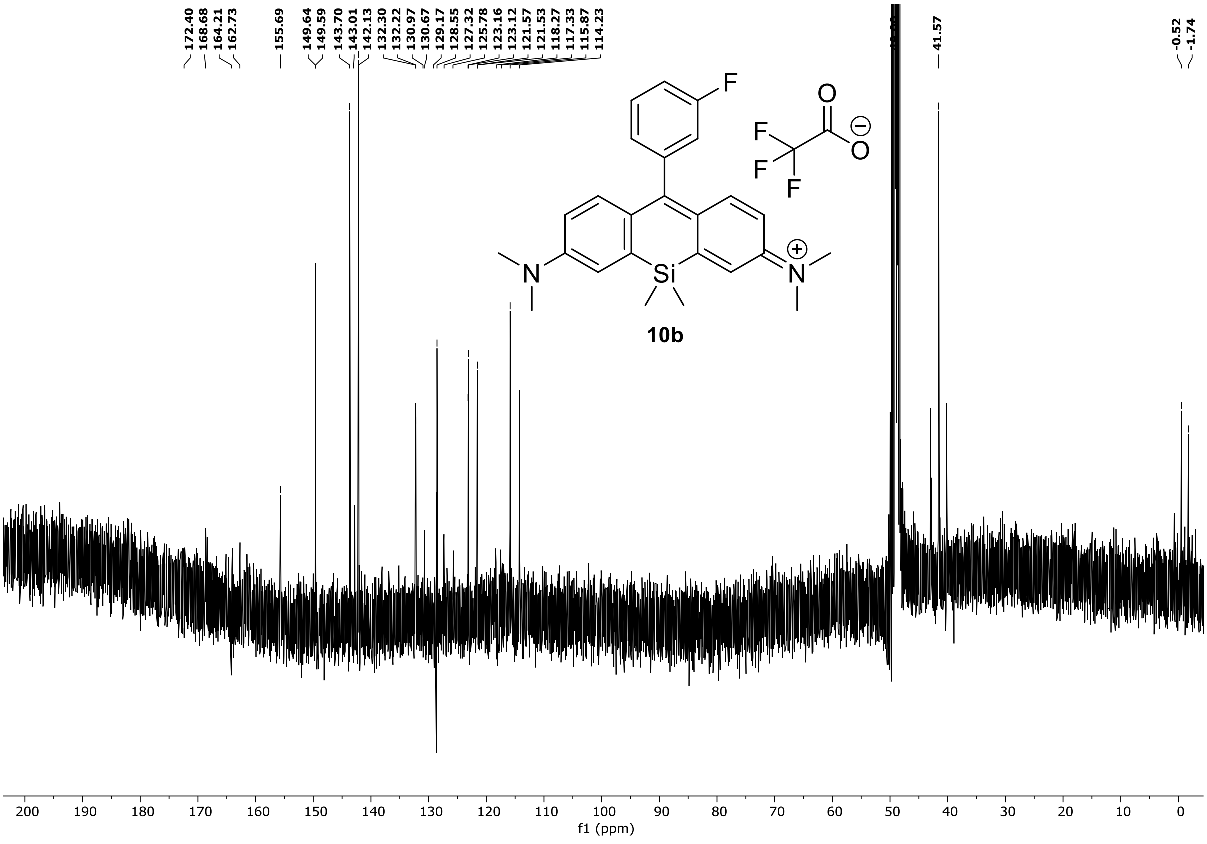

Figure S48: ^{13}C{^{1}H}-NMR-spectrum of **10b** in methanol-d_4 (101 MHz, 300 K).

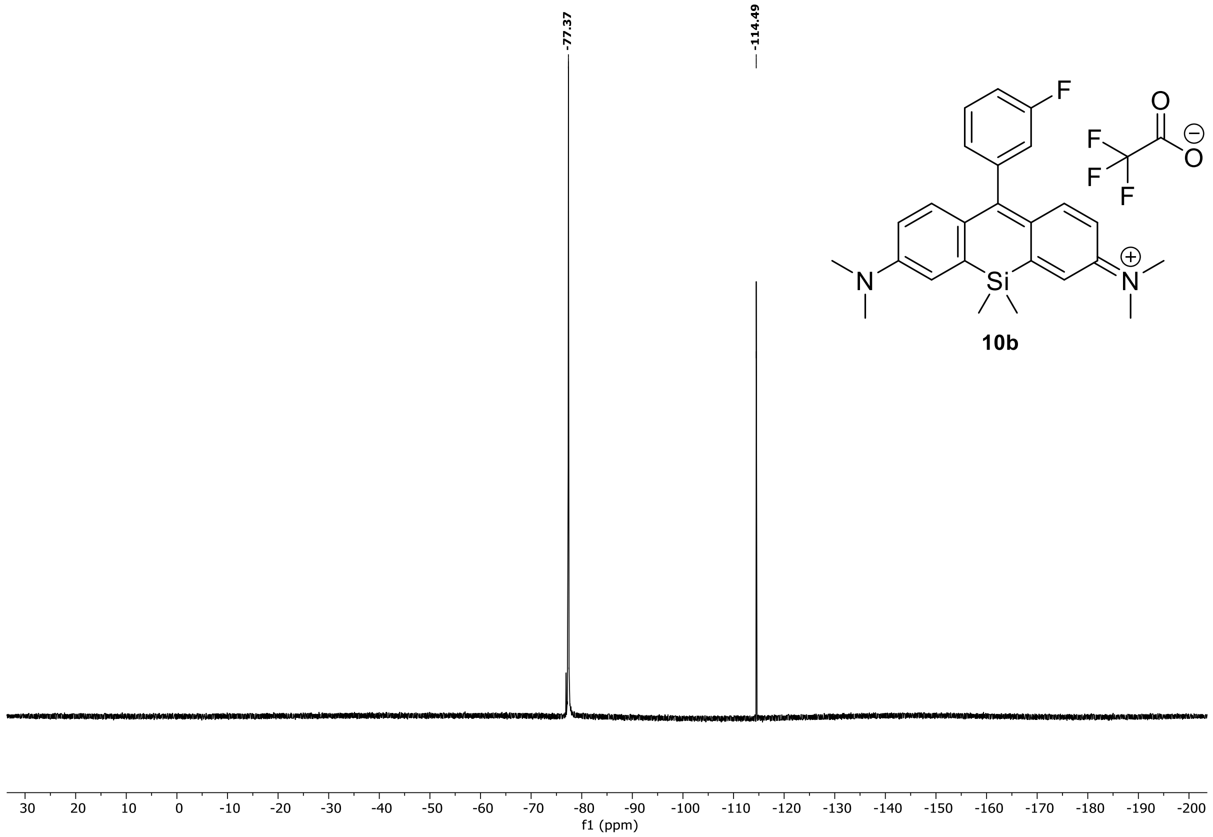

Figure S49: ^{19}F-NMR-spectrum of **10b** in methanol-d_4 (376 MHz, 300 K).

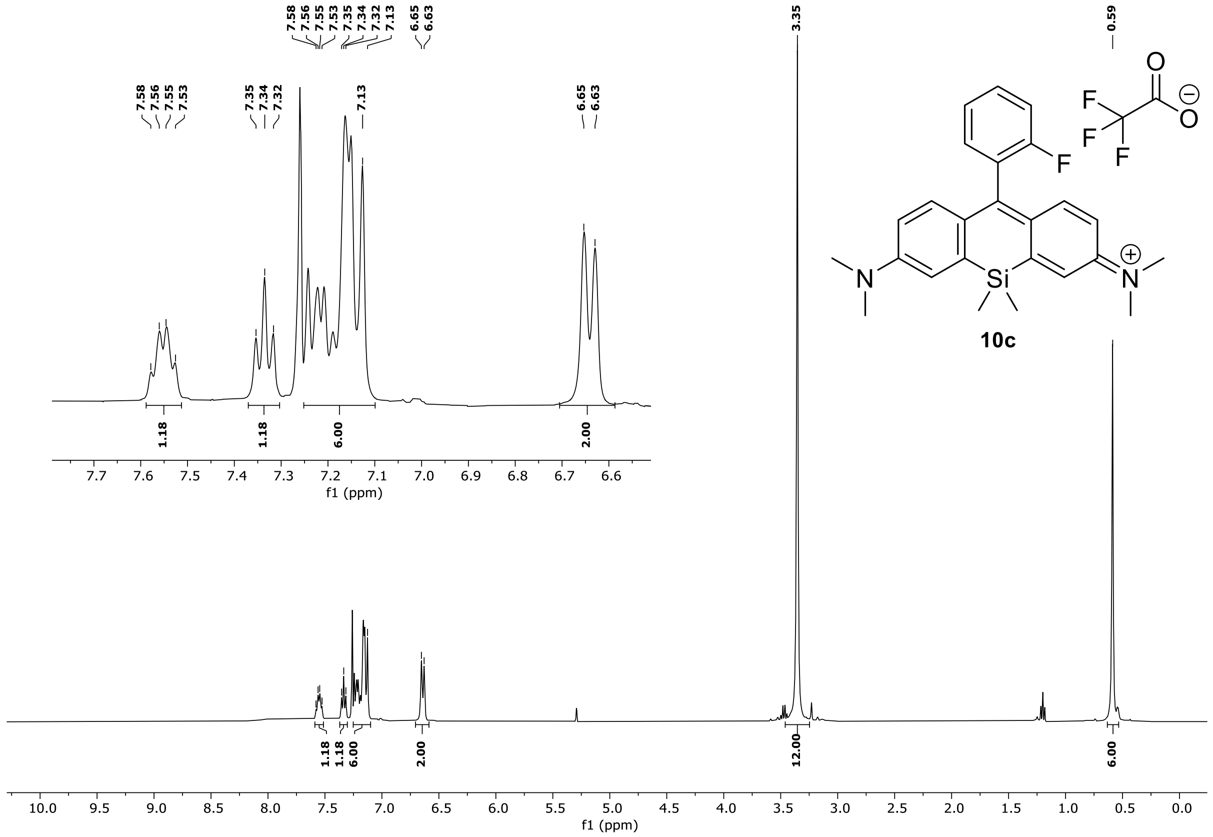

Figure S50: ^{1}H-NMR-spectrum of **10c** in chloroform-d (400 MHz, 300 K).

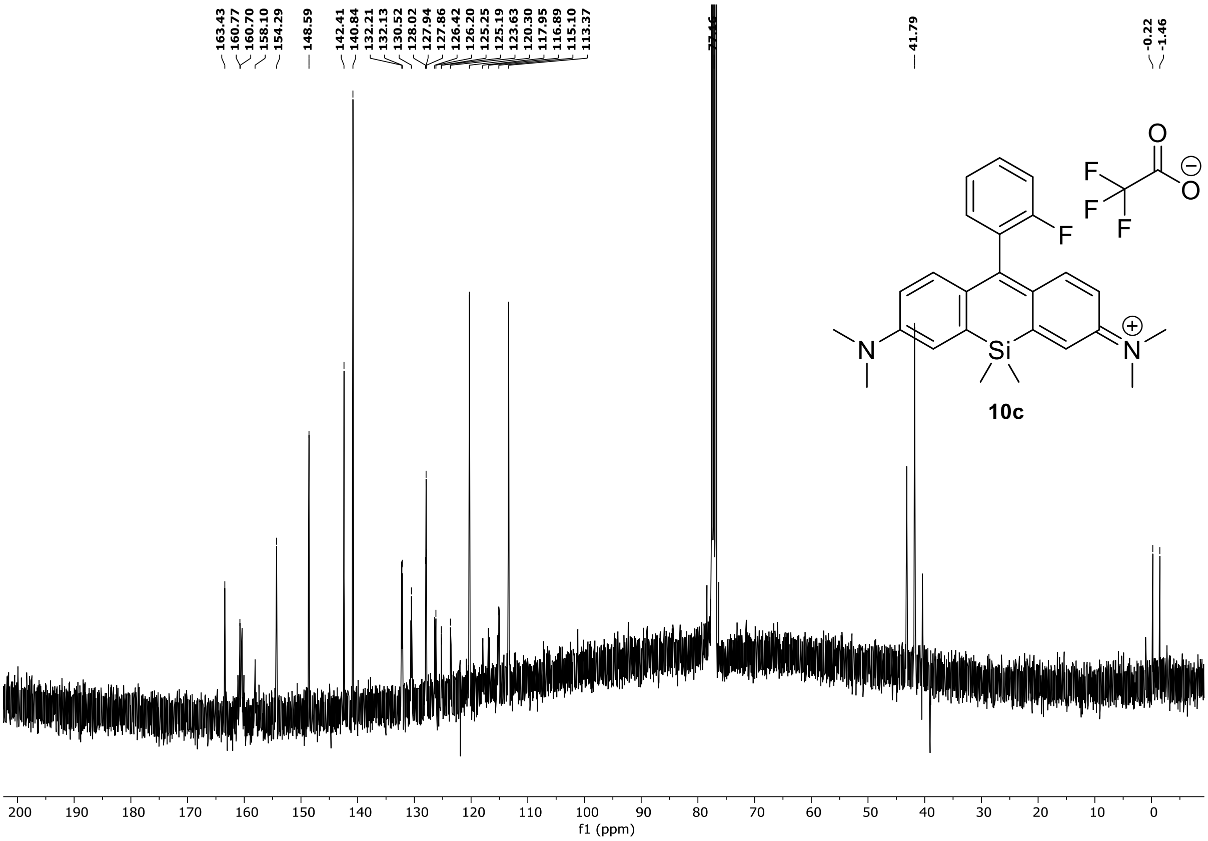

Figure S51: $^{13}C\{^{1}H\}$-NMR-spectrum of **10c** in chloroform-*d* (101 MHz, 300 K).

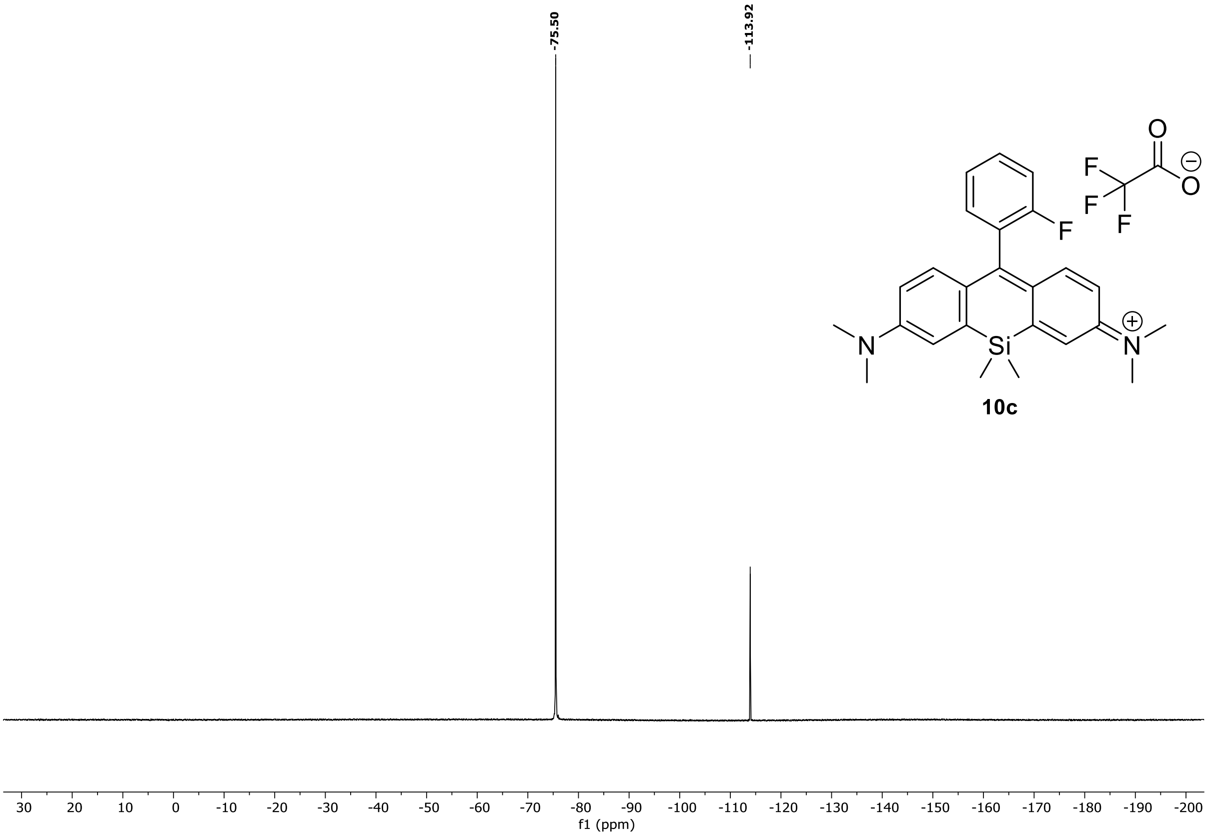

Figure S52: ^{19}F-NMR-spectrum of **10c** in chloroform-*d* (376 MHz, 300 K).

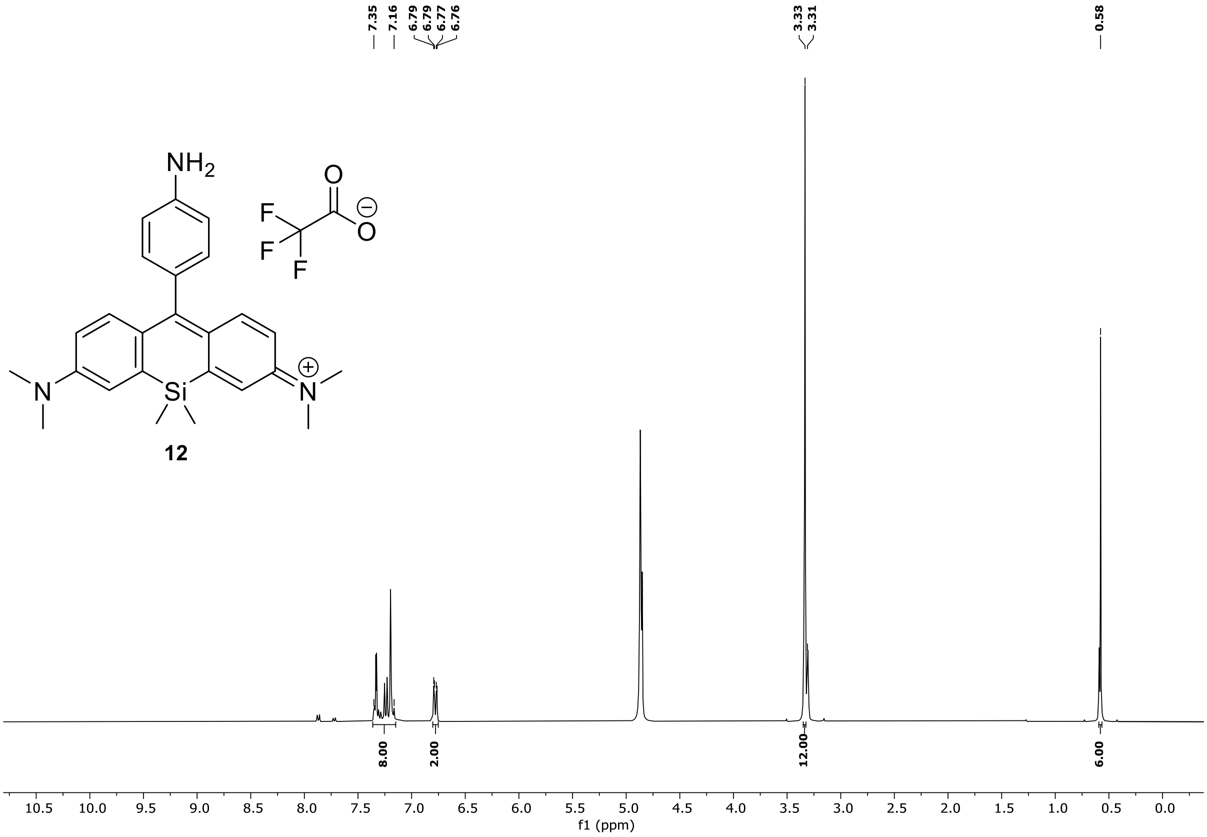

Figure S53: ^{1}H-NMR-spectrum of **12** in methanol-d_4 (400 MHz, 300 K).

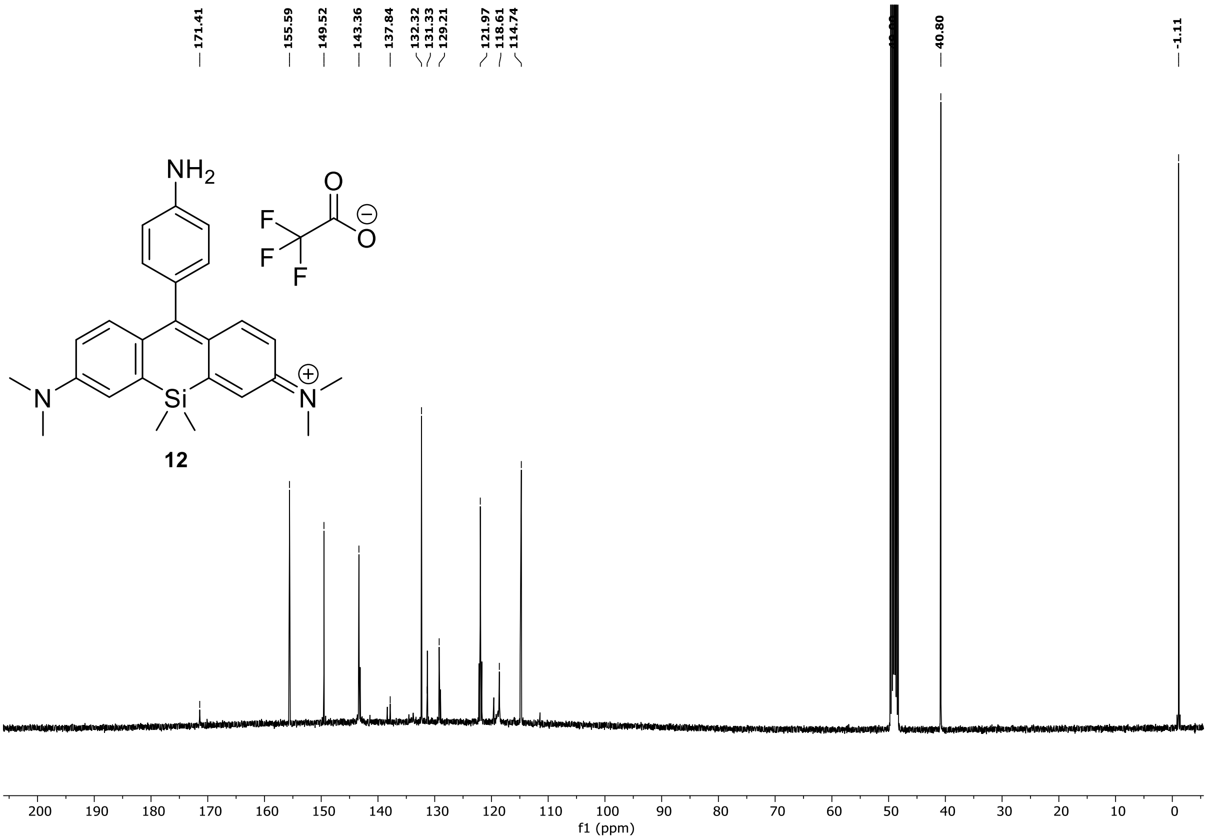

Figure S54: ^{13}C{^{1}H}-NMR-spectrum of **12** in methanol-d_4 (101 MHz, 300 K).

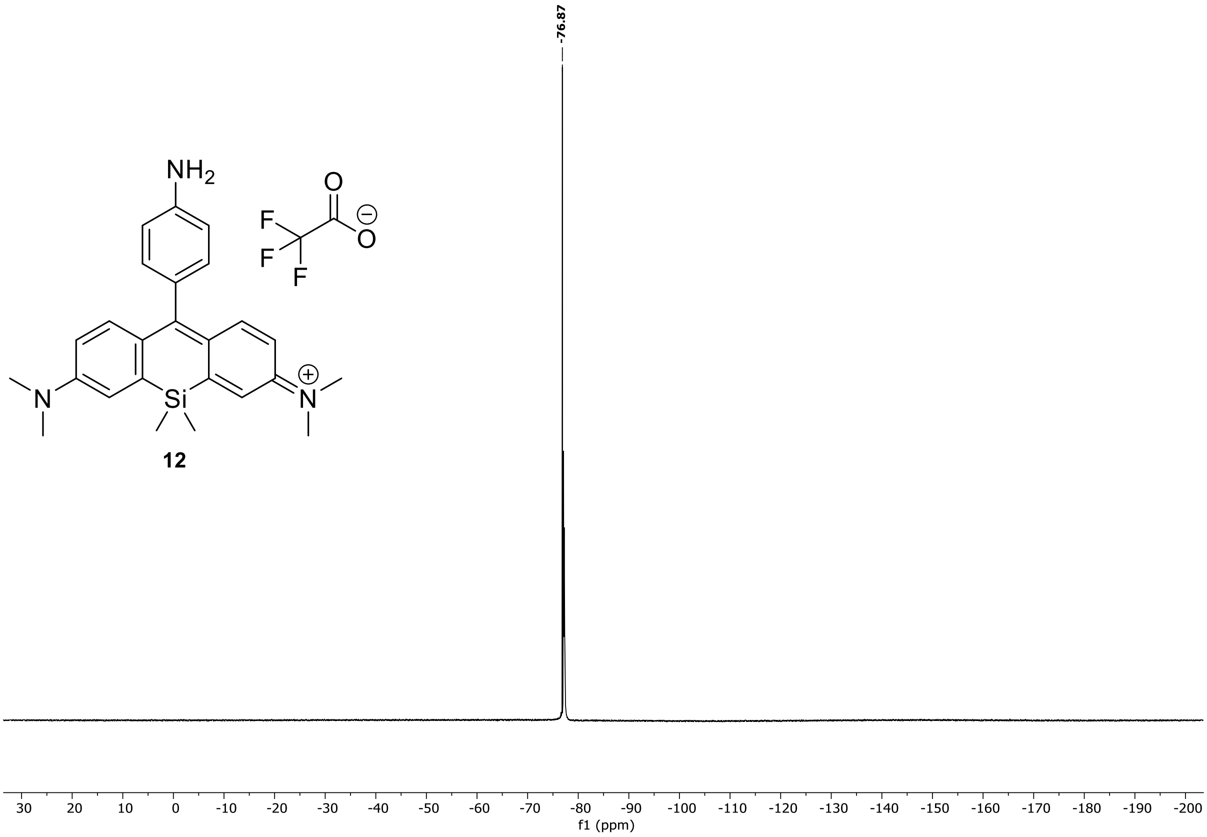

Figure S55: ^{19}F-NMR-spectrum of **12** in methanol-*d₄* (376 MHz, 300 K).

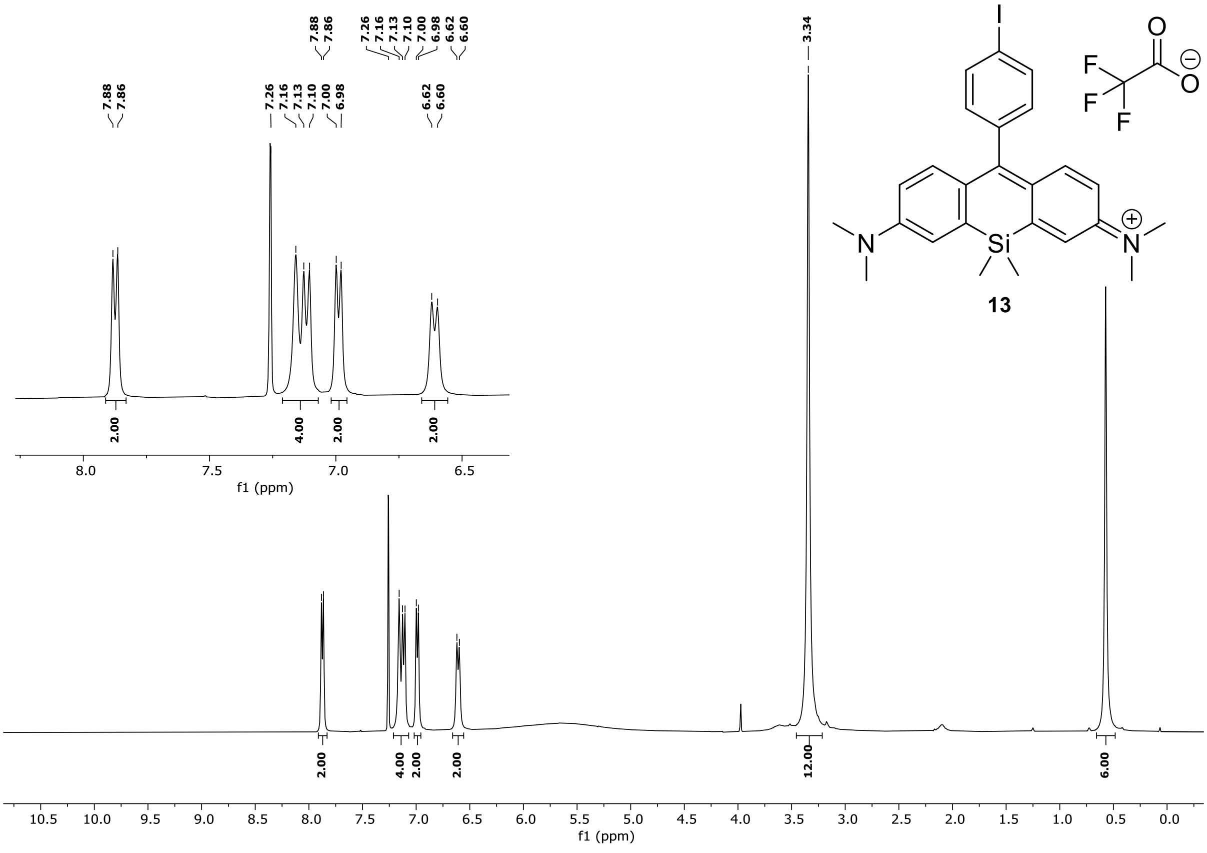

Figure S56: ^{1}H-NMR-spectrum of **13** in chloroform-*d* (400 MHz, 300 K).

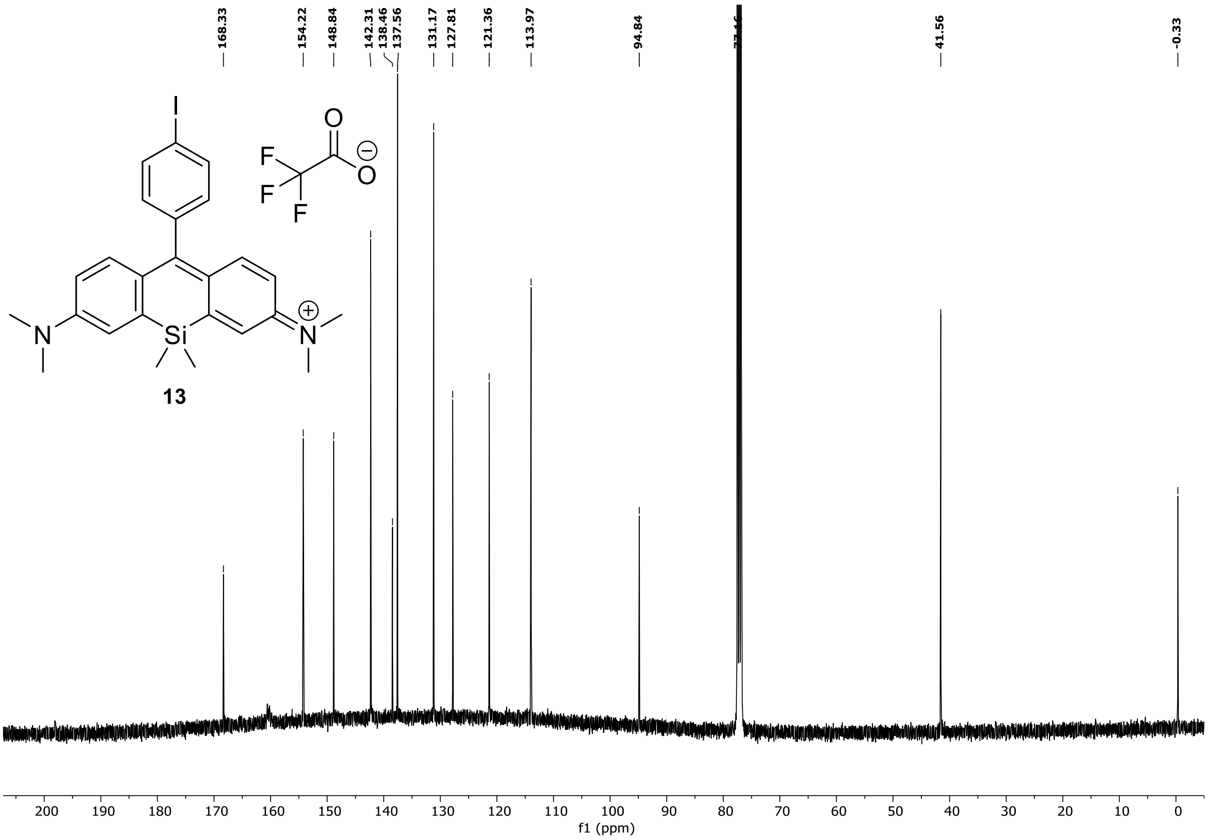

Figure S57: $^{13}C\{^{1}H\}$-NMR-spectrum of **13** in chloroform-*d* (101 MHz, 300 K).

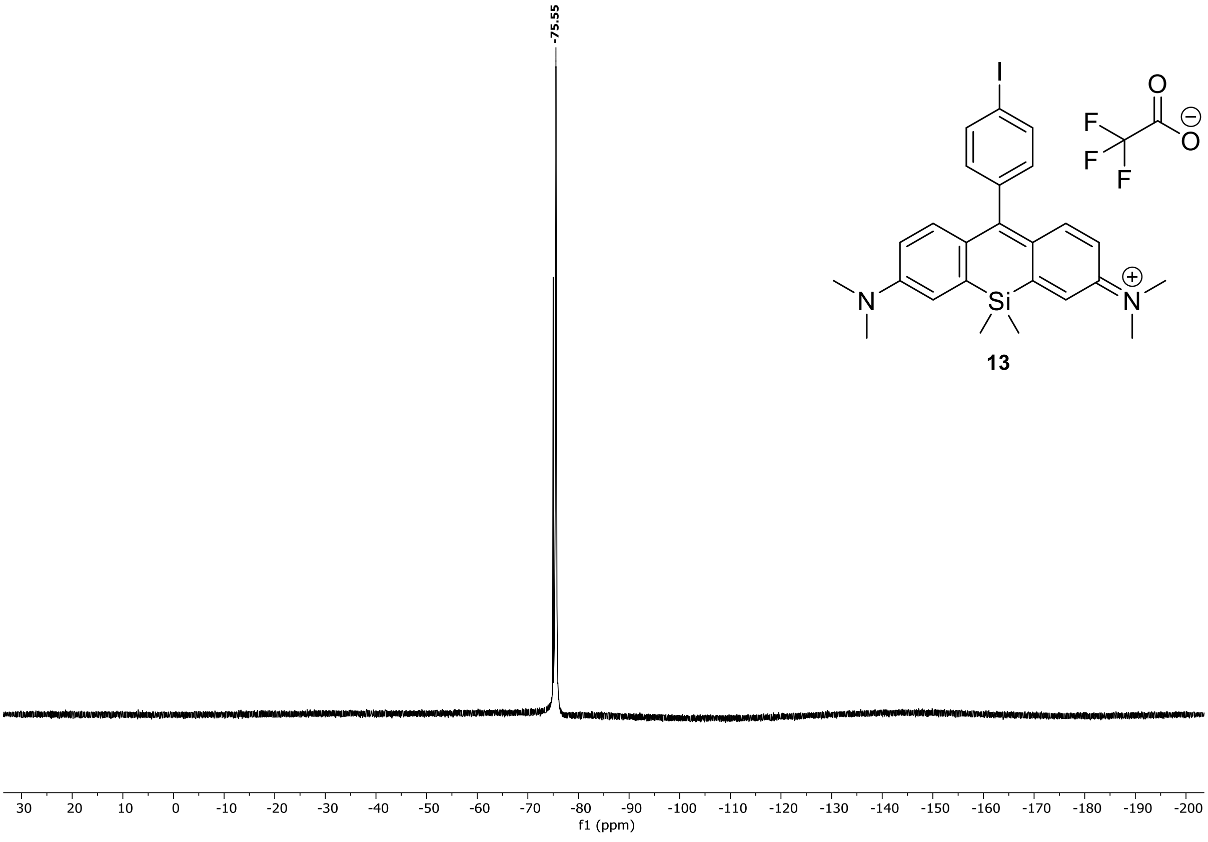

Figure S58: ^{19}F-NMR-spectrum of **13** in chloroform-*d* (376 MHz, 300 K).

4.2 Optical properties of the silicon-rhodamines

4.2.1 UV-Vis-NIR-spectra of the silicon-rhodamines

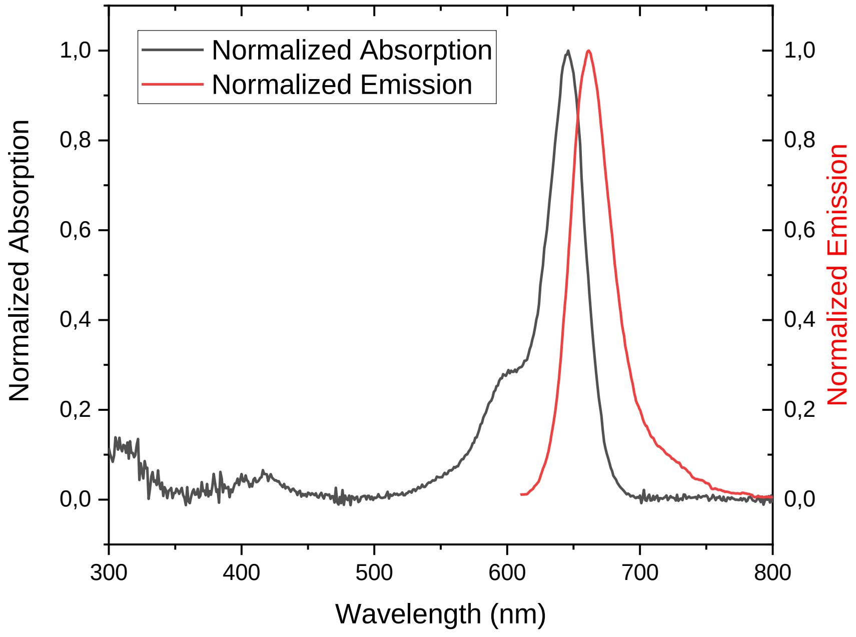

Figure S59: Normalized absorption and emission UV-Vis-NIR-spectrum of **6a** in PBS at room temperature (λ_{ex}= 600 nm).

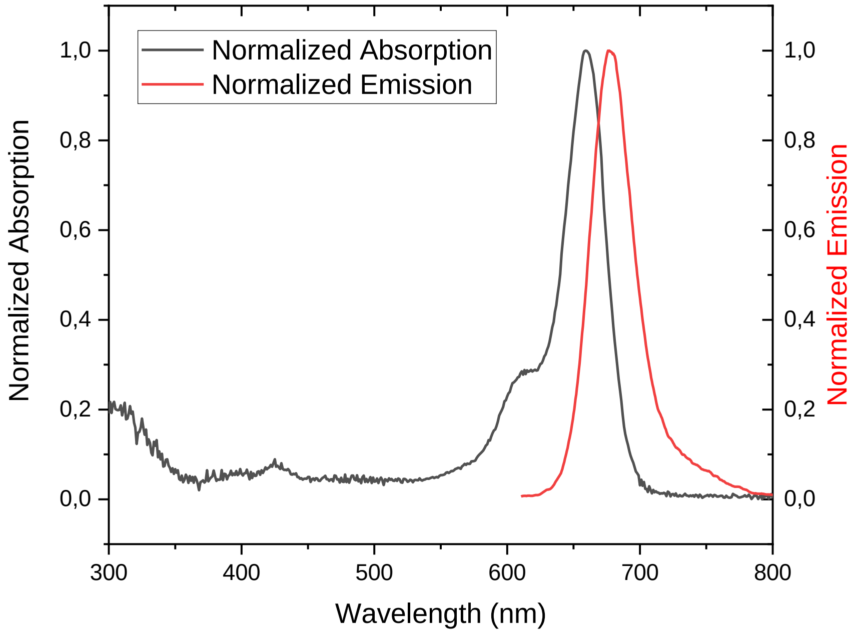

Figure S60: Normalized absorption and emission UV-Vis-NIR-spectrum of **6a** in DMSO at room temperature (λ_{ex}= 600 nm).

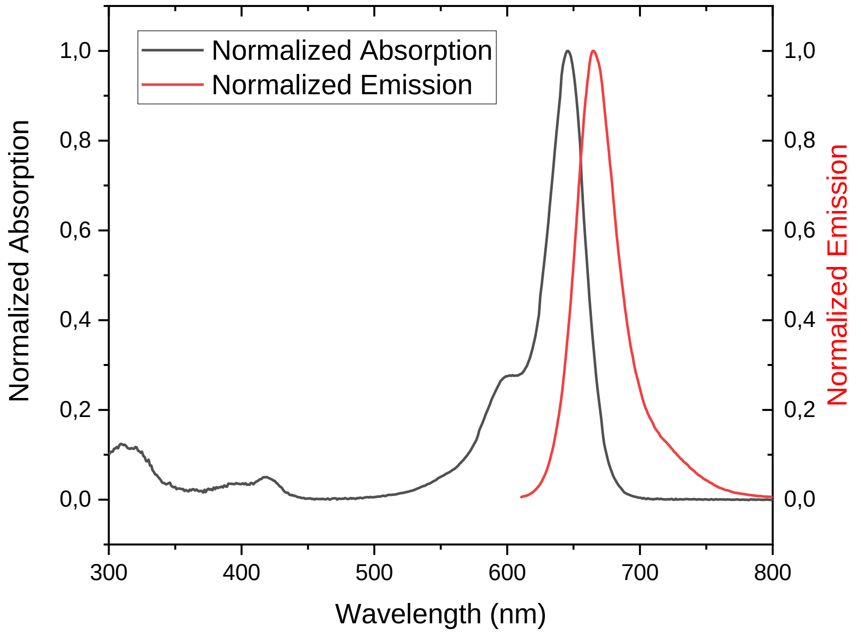

Figure S61: Normalized absorption and emission UV-Vis-NIR-spectrum of **6b** in PBS at room temperature (λ_{ex}= 600 nm).

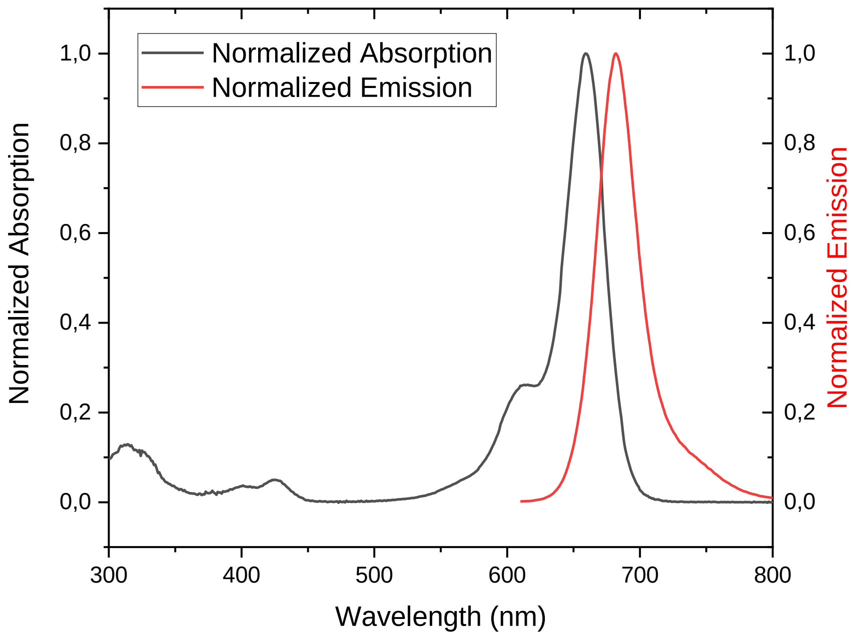

Figure S62: Normalized absorption and emission UV-Vis-NIR-spectrum of **6b** in DMSO at room temperature (λ_{ex}= 600 nm).

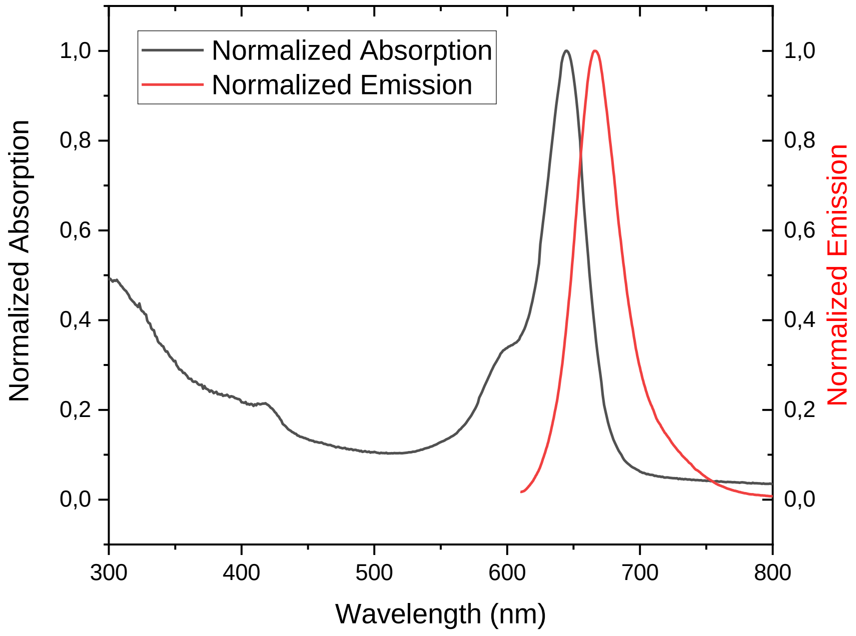

Figure S63: Normalized absorption and emission UV-Vis-NIR-spectrum of **6c** in HCl (0.1 M) at room temperature (λ_{ex}= 600 nm).

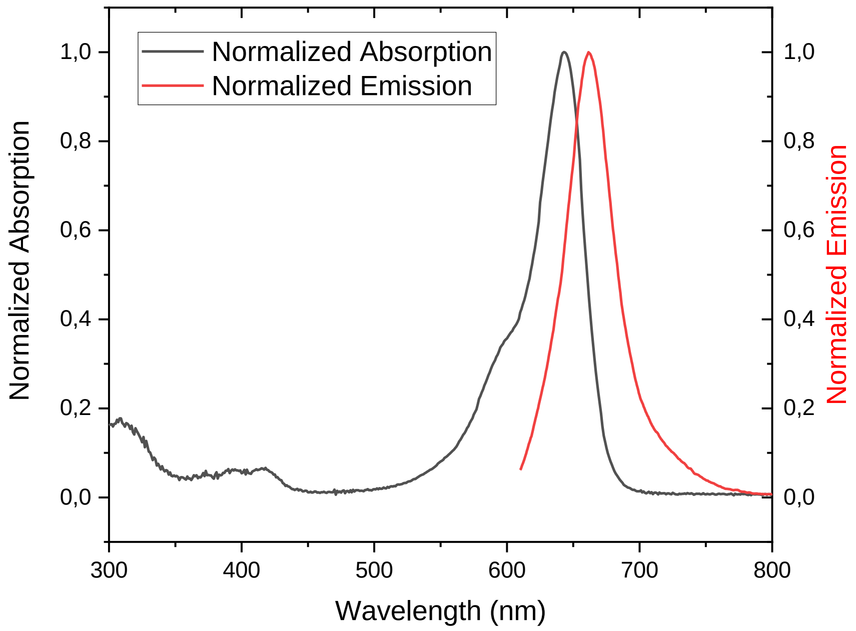

Figure S64: Normalized absorption and emission UV-Vis-NIR-spectrum of **8** in PBS at room temperature (λ_{ex}= 600 nm).

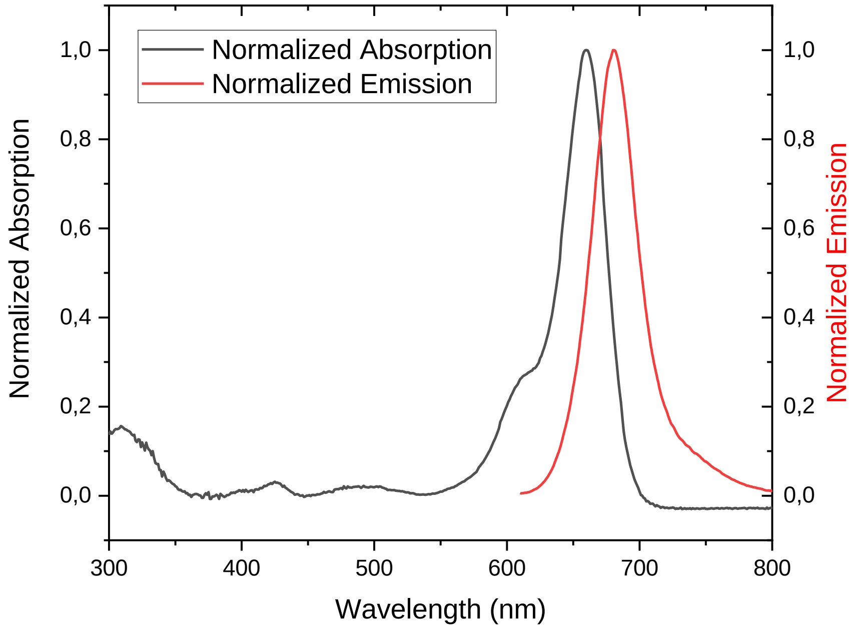

Figure S65: Normalized absorption and emission UV-Vis-NIR-spectrum of **8** in DMSO at room temperature (λ_{ex}= 600 nm).

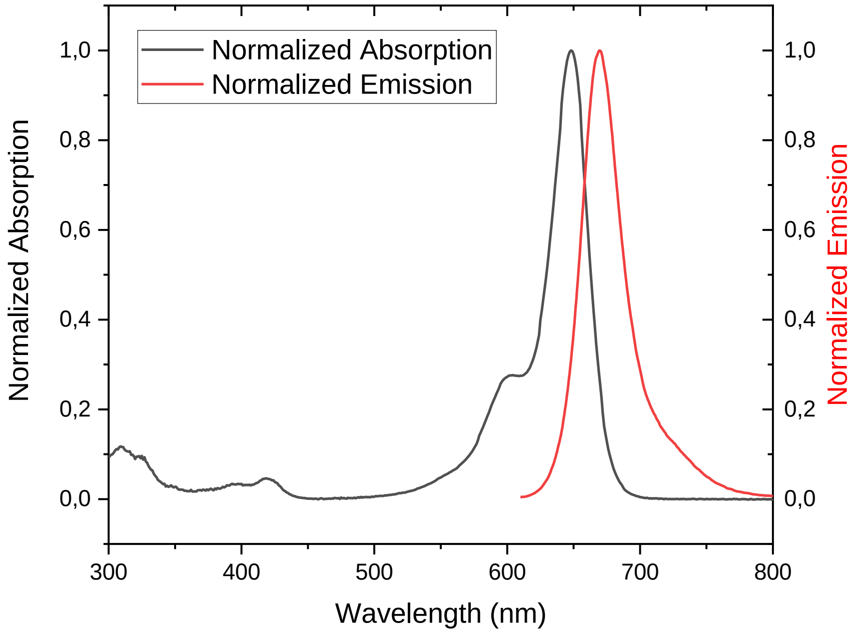

Figure S66: Normalized absorption and emission UV-Vis-NIR-spectrum of **10a** in PBS at room temperature (λ_{ex}= 600 nm).

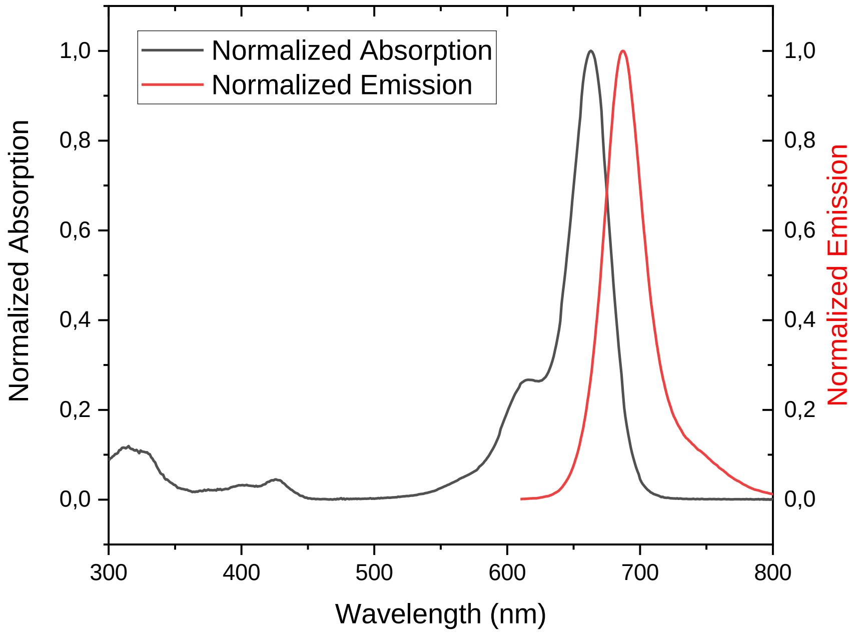

Figure S67: Normalized absorption and emission UV-Vis-NIR-spectrum of **10a** in DMSO at room temperature (λ_{ex}= 600 nm).

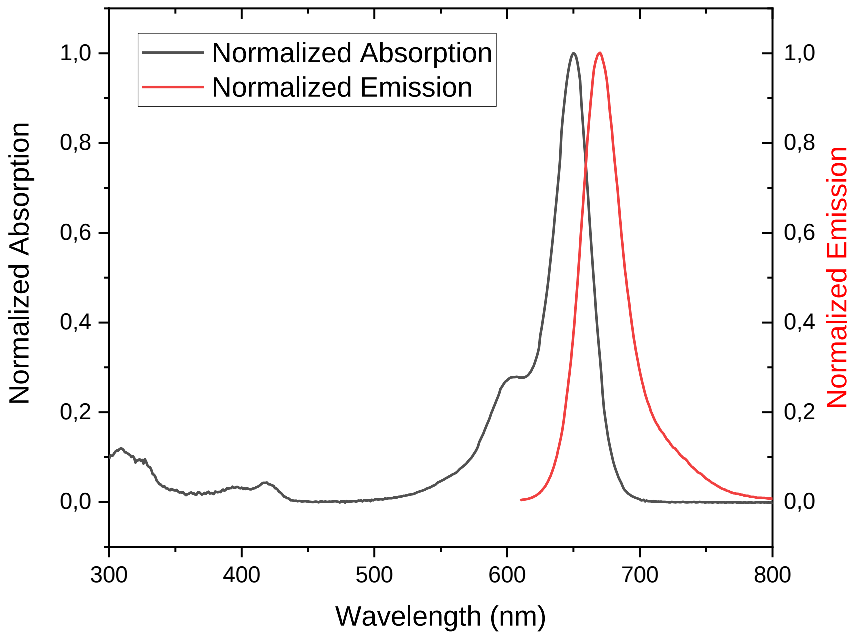

Figure S68: Normalized absorption and emission UV-Vis-NIR-spectrum of **10b** in PBS at room temperature (λ_{ex}= 600 nm).

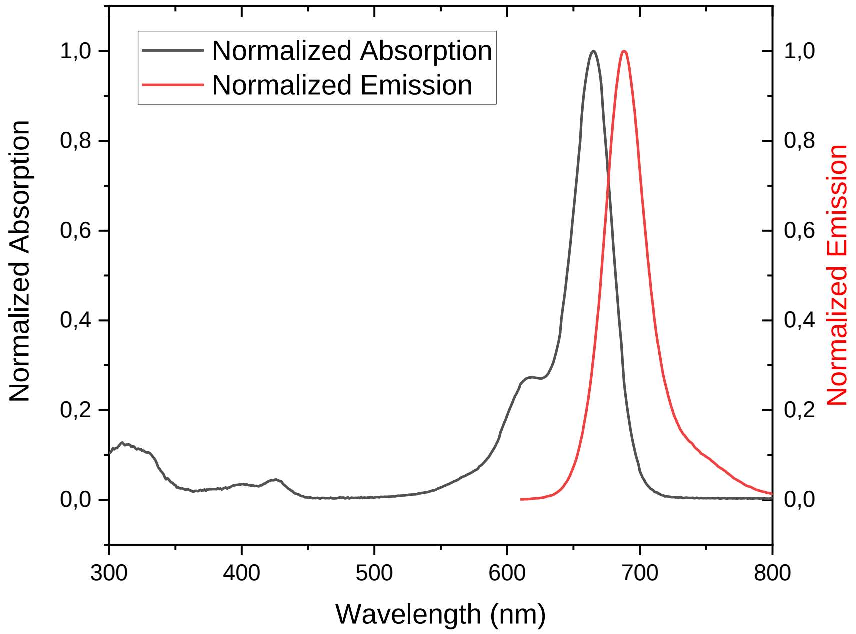

Figure S69: Normalized absorption and emission UV-Vis-NIR-spectrum of **10b** in DMSO at room temperature (λ_{ex}= 600 nm).

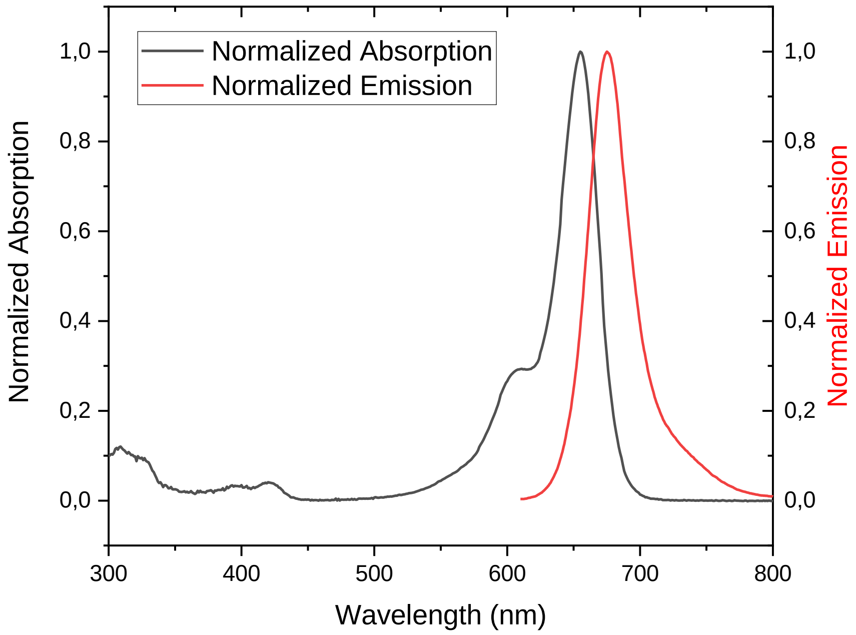

Figure S70: Normalized absorption and emission UV-Vis-NIR-spectrum of **10c** in PBS at room temperature (λ_{ex}= 600 nm).

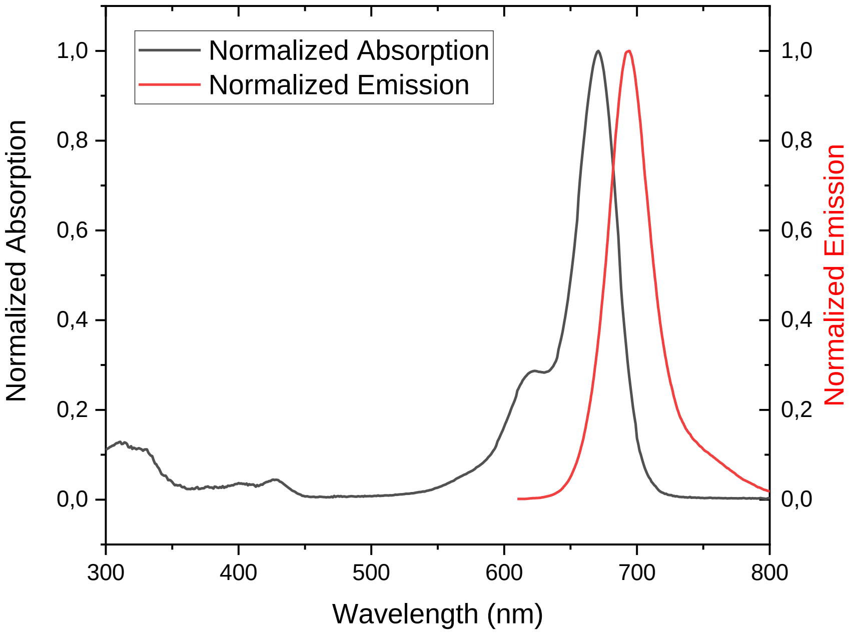

Figure S71: Normalized absorption and emission UV-Vis-NIR-spectrum of **10c** in DMSO at room temperature (λ_{ex}= 600 nm).

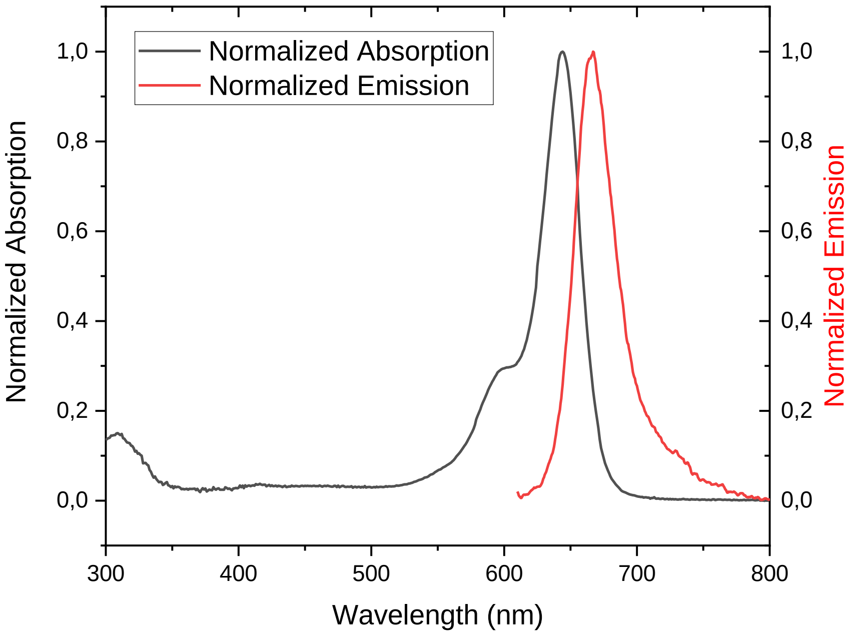

Figure S72: Normalized absorption and emission UV-Vis-NIR-spectrum of **12** in PBS at room temperature (λ_{ex}= 600 nm).

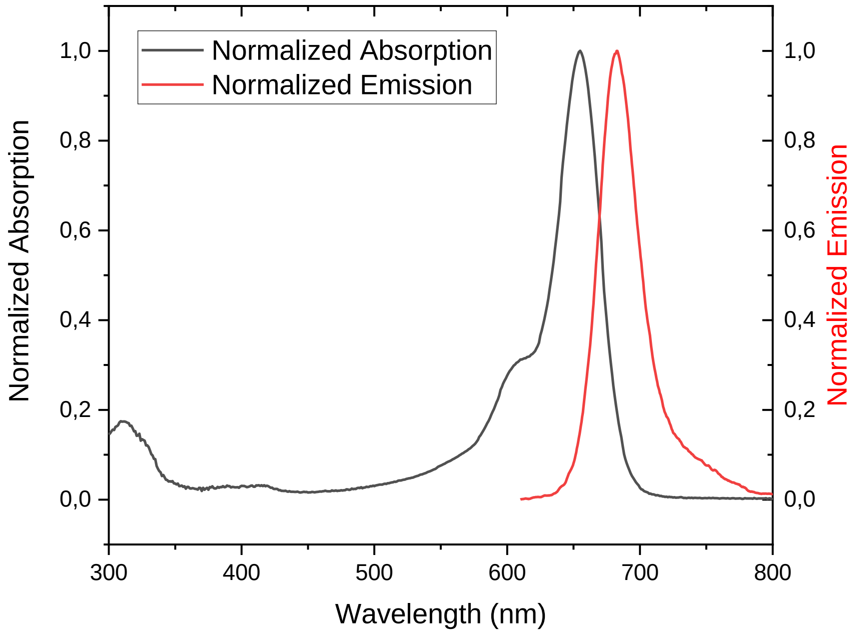

Figure S73: Normalized absorption and emission UV-Vis-NIR-spectrum of **12** in DMSO at room temperature (λ_{ex}= 600 nm).

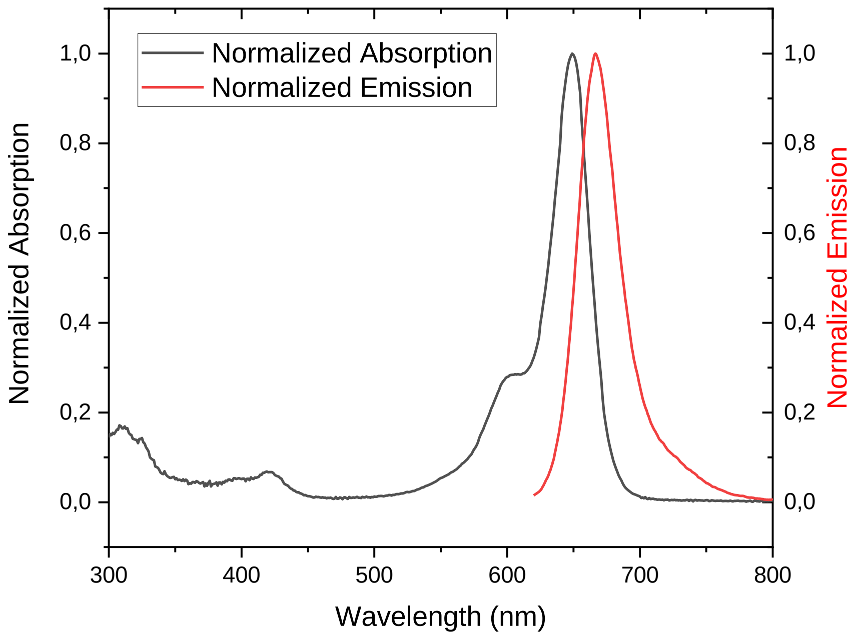

Figure S74: Normalized absorption and emission UV-Vis-NIR-spectrum of **13** in PBS at room temperature (λ_{ex}= 600 nm).

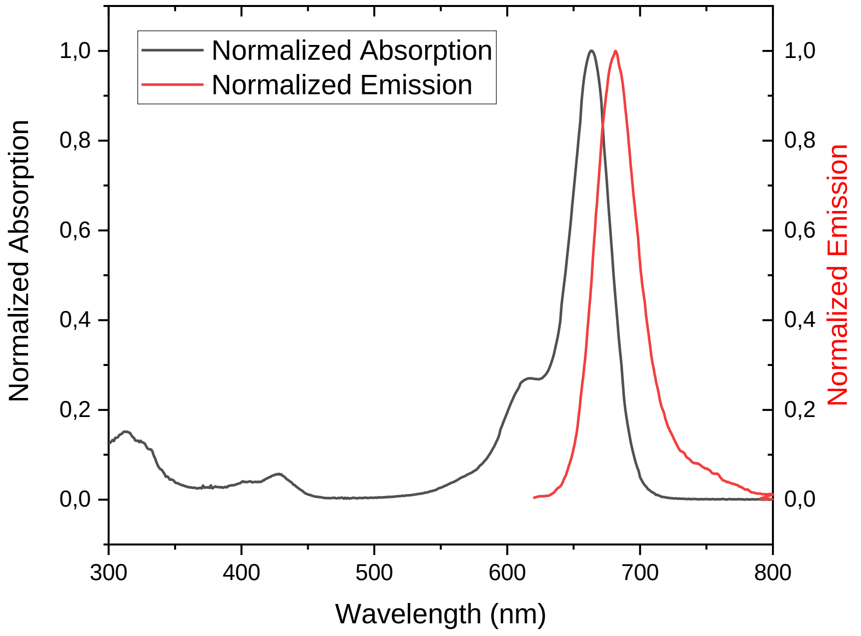

Figure S75: Normalized absorption and emission UV-Vis-NIR-spectrum of **13** in DMSO at room temperature (λ_{ex}= 600 nm).

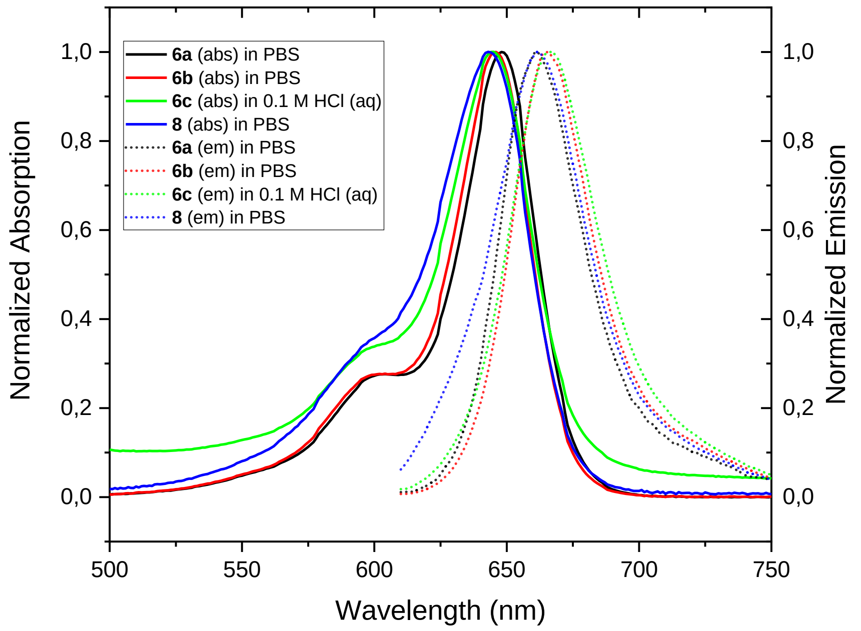

Figure S76: Overview of the normalized absorption and emission UV-Vis-NIR-spectra of **6a–6c** in PBS and **8** in 0.1 M HCl (aq) at room temperature (λ_{ex}= 600 nm).

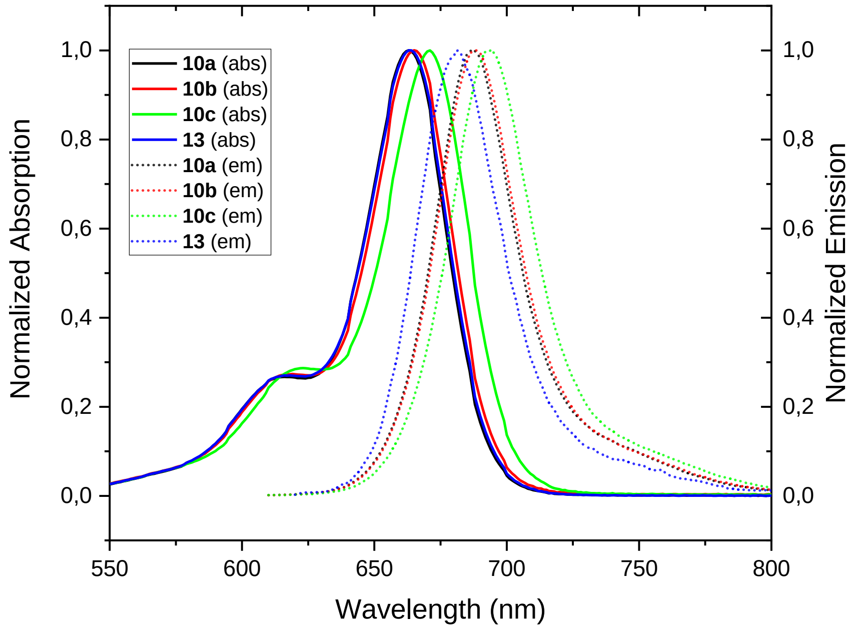

Figure S77: Overview of the normalized absorption and emission UV-Vis-NIR-spectra of **10a–10c** and **13** in DMSO at room temperature (λ_{ex}= 600 nm).

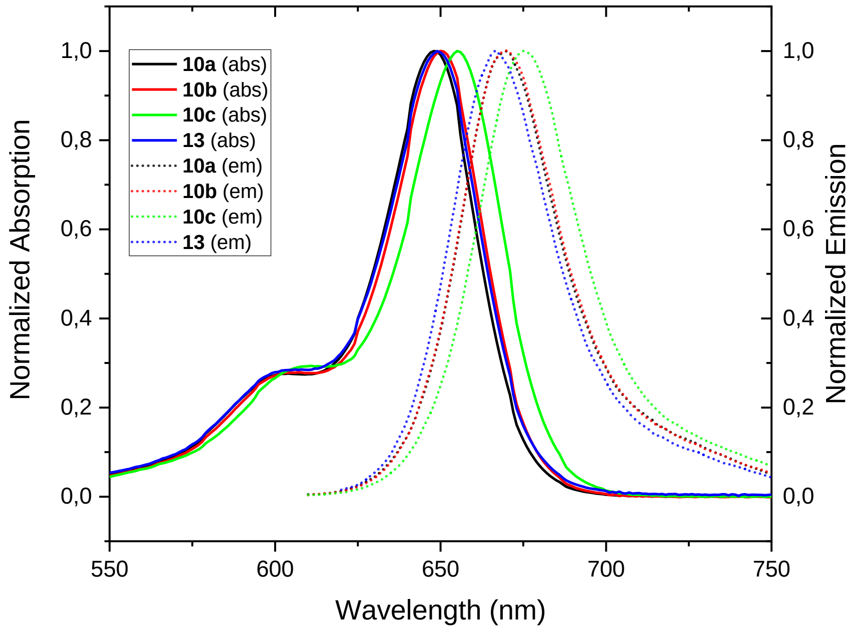

Figure S78: Overview of the normalized absorption and emission UV-Vis-NIR-spectra of **10a–10c** and **13** in PBS at room temperature (λ_{ex}= 600 nm).

4.2.2 Photostability experiments of silicon-rhodamines and Abberior® STAR635

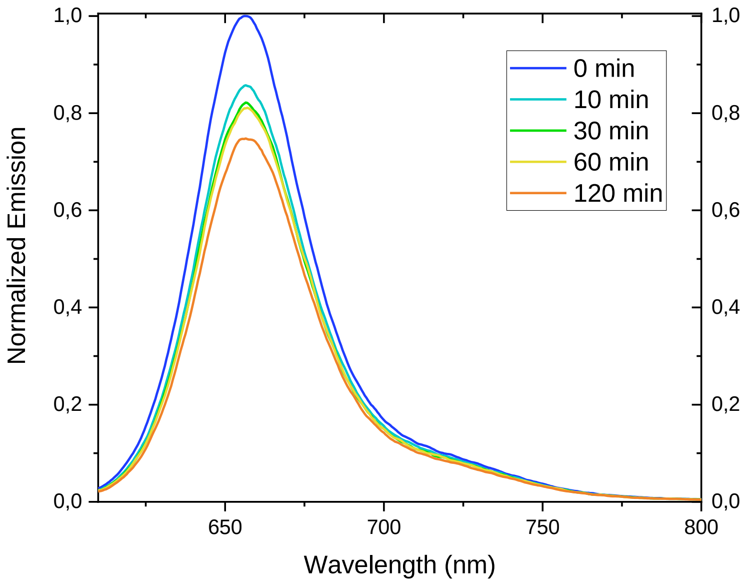

Figure S79: Photostability experiments. Emission spectra of Abberior® STAR 635P between a wavelength of 610 nm and 800 nm after irradiation with a wavelength of 640 nm from a pulsed laser (20 kW, pulse width at half peak height < 10 µs) up to two hours in DMSO. The emission spectrum for 0 min was normalized and the emission spectra for the following time points are referenced to the spectrum at 0 min. The excitation wavelength for recording the emission spectra was λ_{ex}= 600 nm.

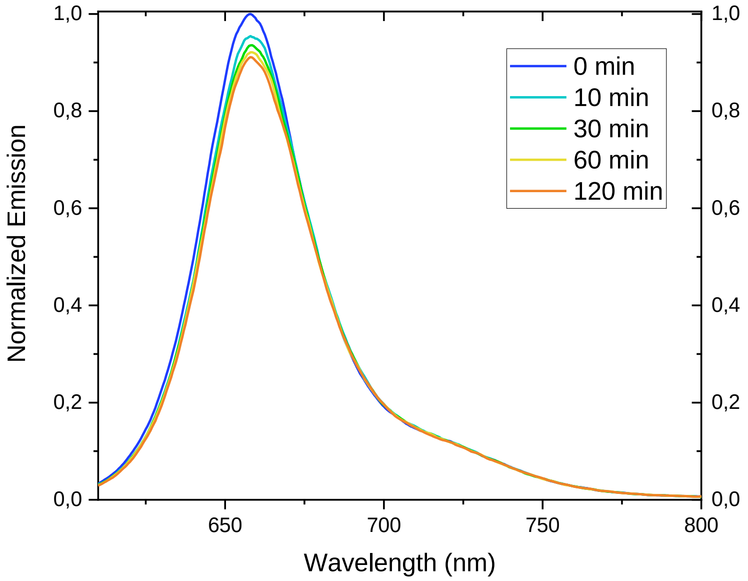

Figure S80: Photostability experiments. Emission spectra of Abberior® STAR 635P between a wavelength of 610 nm and 800 nm after irradiation with a wavelength of 640 nm from a pulsed laser (20 kW, pulse width at half peak height < 10 µs) up to two hours in PBS (+1% DMSO). The emission spectrum for 0 min was normalized and the emission spectra for the following time points are referenced to the spectrum at 0 min. The excitation wavelength for recording the emission spectra was λ_{ex}= 600 nm.

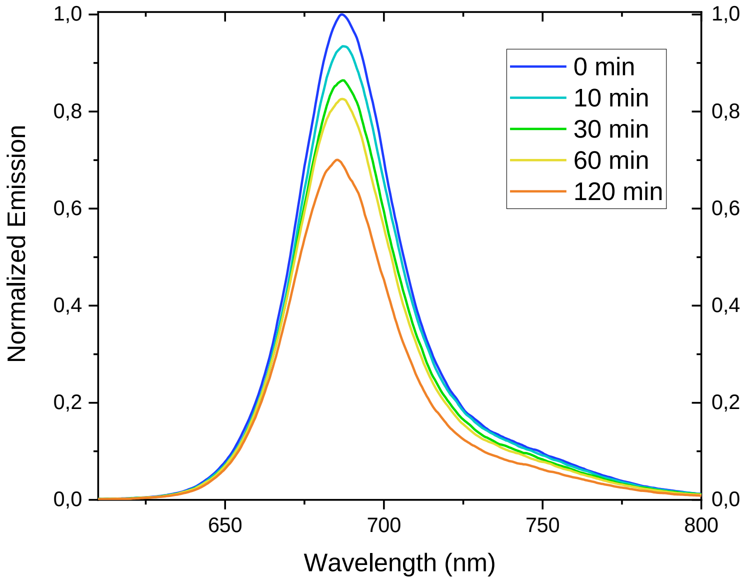

Figure S81: Photostability experiments. Emission spectra of **10a** between a wavelength of 610 nm and 800 nm after irradiation with a wavelength of 640 nm from a pulsed laser (20 kW, pulse width at half peak height < 10 µs) up to two hours in DMSO. The emission spectrum for 0 min was normalized and the emission spectra for the following time points are referenced to the spectrum at 0 min. The excitation wavelength for recording the emission spectra was λ_{ex}= 600 nm.

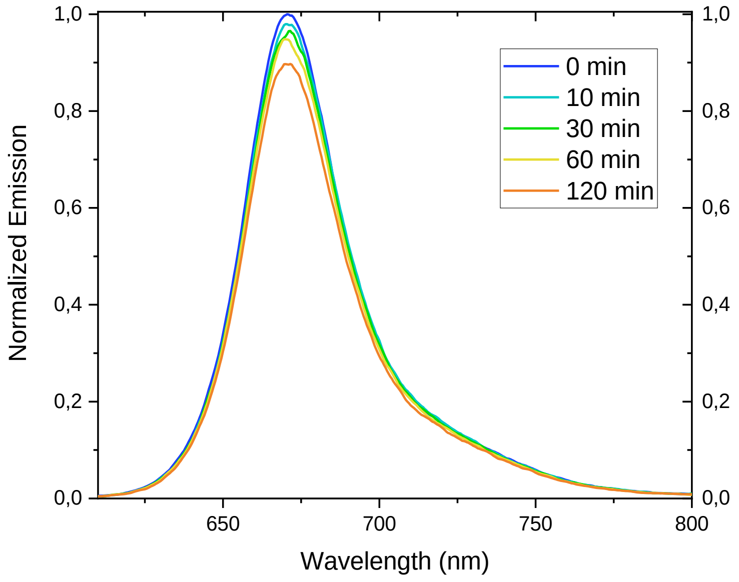

Figure S82: Photostability experiments. Emission spectra of **10a** between a wavelength of 610 nm and 800 nm after irradiation with a wavelength of 640 nm from a pulsed laser (20 kW, pulse width at half peak height < 10 µs) up to two hours in PBS (+1% DMSO). The emission spectrum for 0 min was normalized and the emission spectra for the following time points are referenced to the spectrum at 0 min. The excitation wavelength for recording the emission spectra was λ_{ex}= 600 nm.

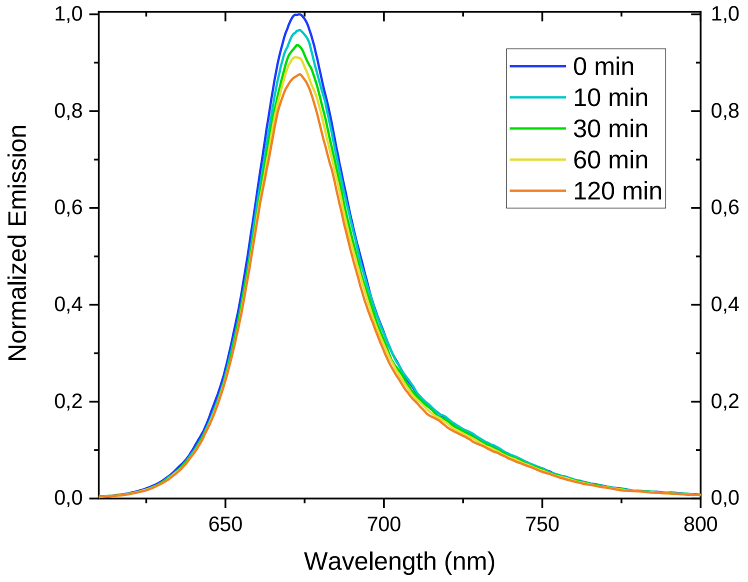

Figure S83: Photostability experiments. Emission spectra of **10b** between a wavelength of 610 nm and 800 nm after irradiation with a wavelength of 640 nm from a pulsed laser (20 kW, pulse width at half peak height < 10 µs) up to two hours in PBS (+1% DMSO). The emission spectrum for 0 min was normalized and the emission spectra for the following time points are referenced to the spectrum at 0 min. The excitation wavelength for recording the emission spectra was λ_{ex}= 600 nm.

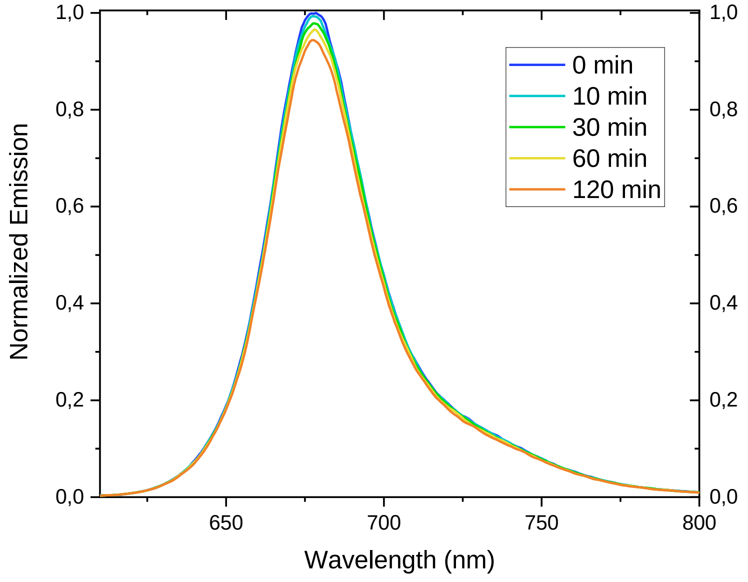

Figure S84: Photostability experiments. Emission spectra of **10c** between a wavelength of 610 nm and 800 nm after irradiation with a wavelength of 640 nm from a pulsed laser (20 kW, pulse width at half peak height < 10 µs) up to two hours in PBS (+1% DMSO). The emission spectrum for 0 min was normalized and the emission spectra for the following time points are referenced to the spectrum at 0 min. The excitation wavelength for recording the emission spectra was λ_{ex}= 600 nm.

4.3 IR-spectra

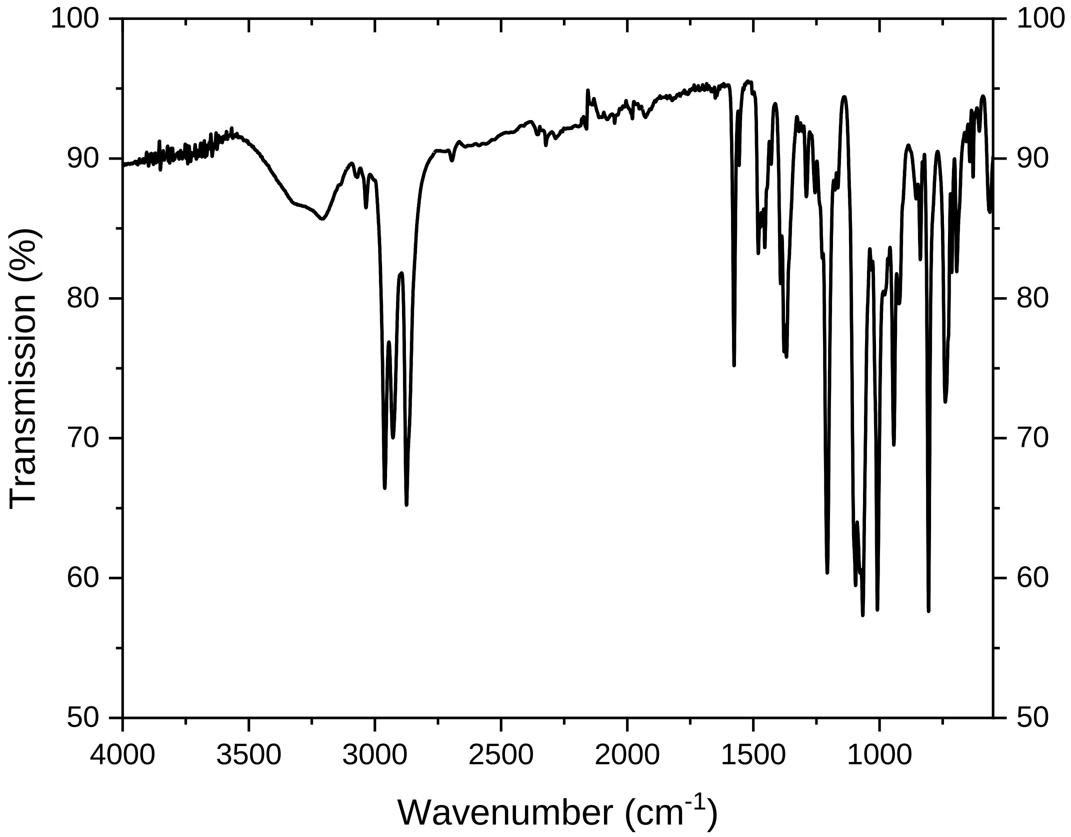

Figure S85: IR-spectrum of **2a**.

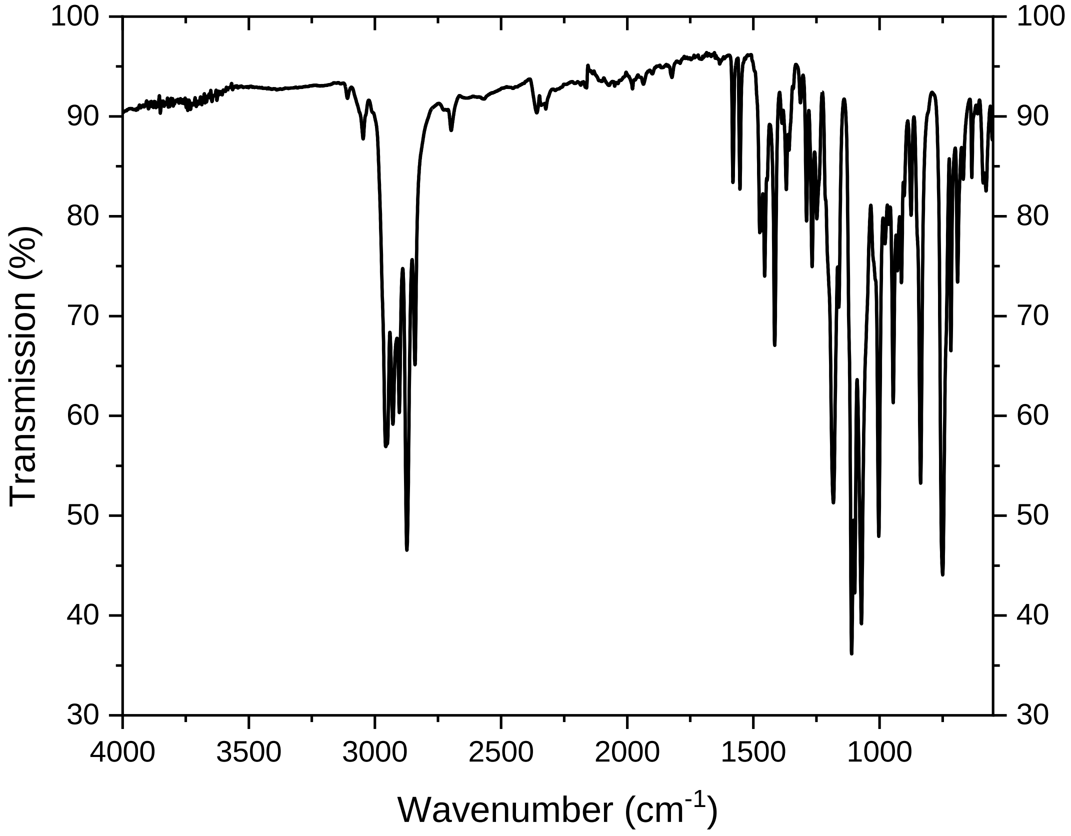

Figure S86: IR-spectrum of **2b**.

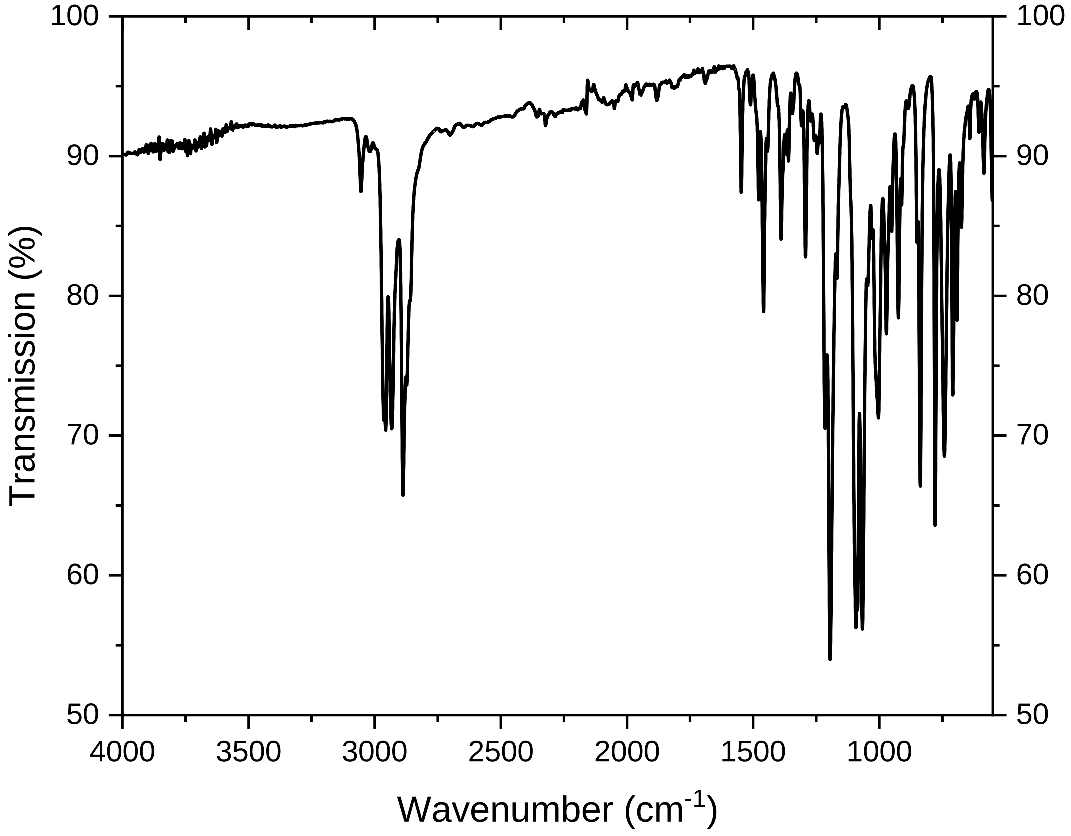

Figure S87: IR-spectrum of **2c**.

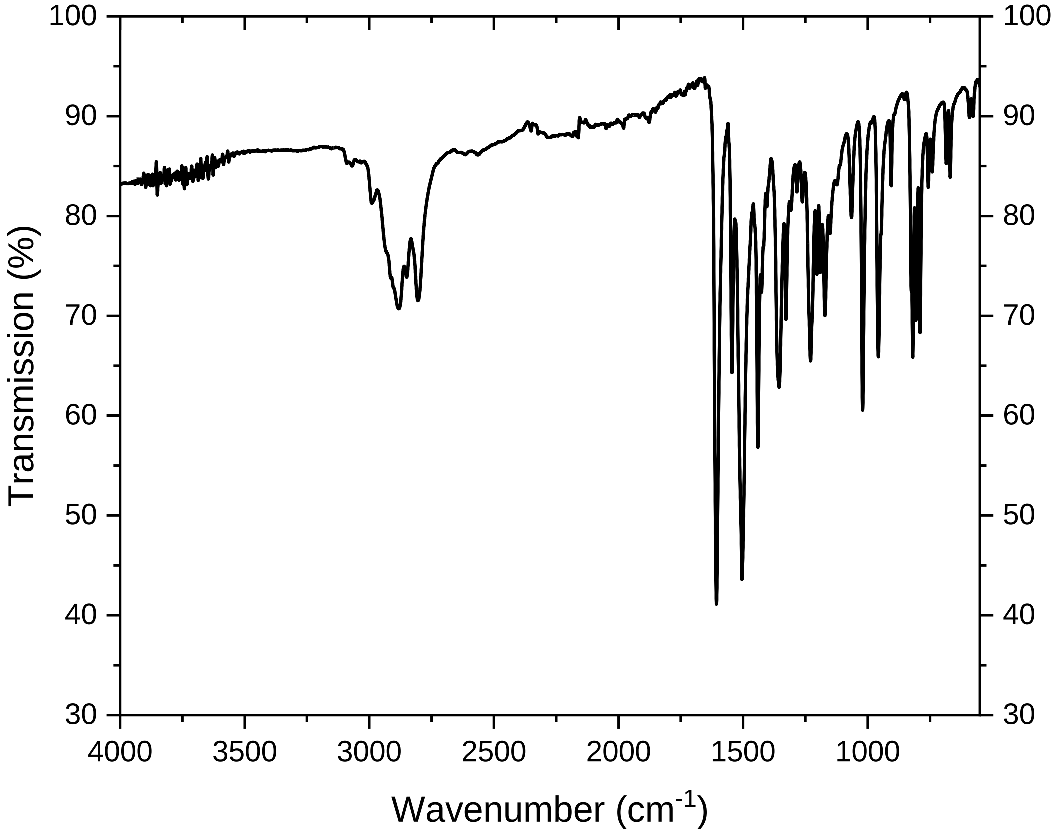

Figure S88: IR-spectrum of **4.**

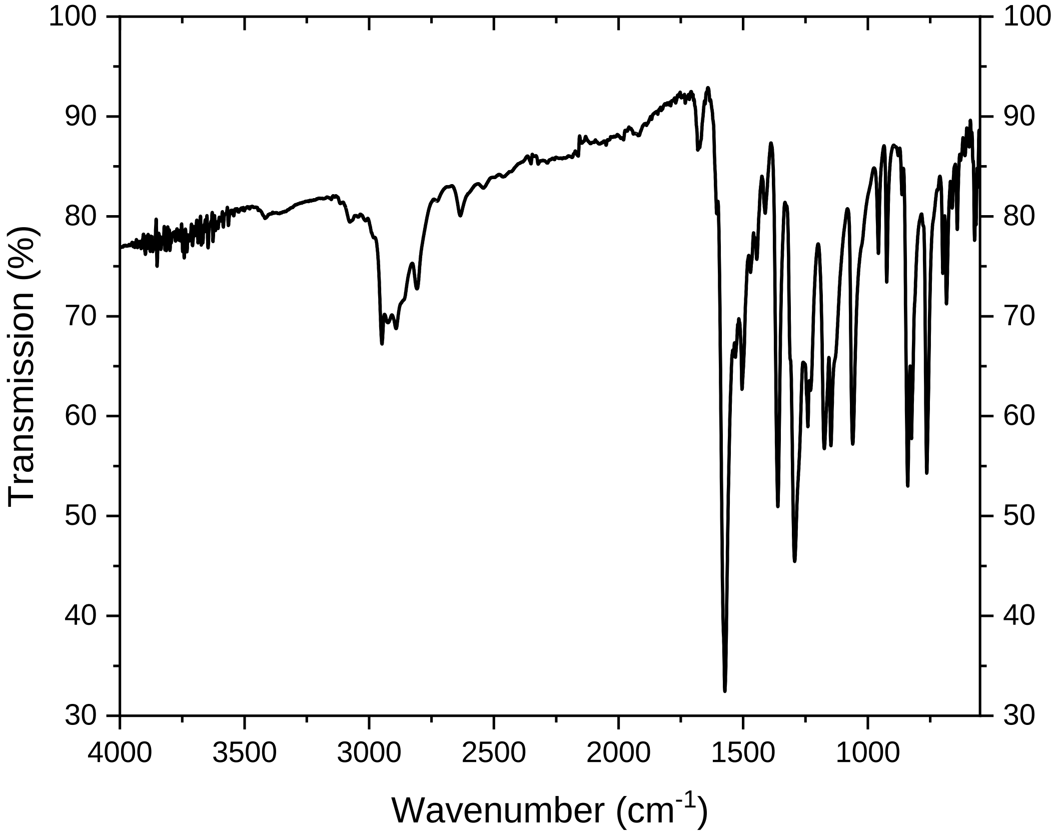

Figure S89: IR-spectrum of **5.**

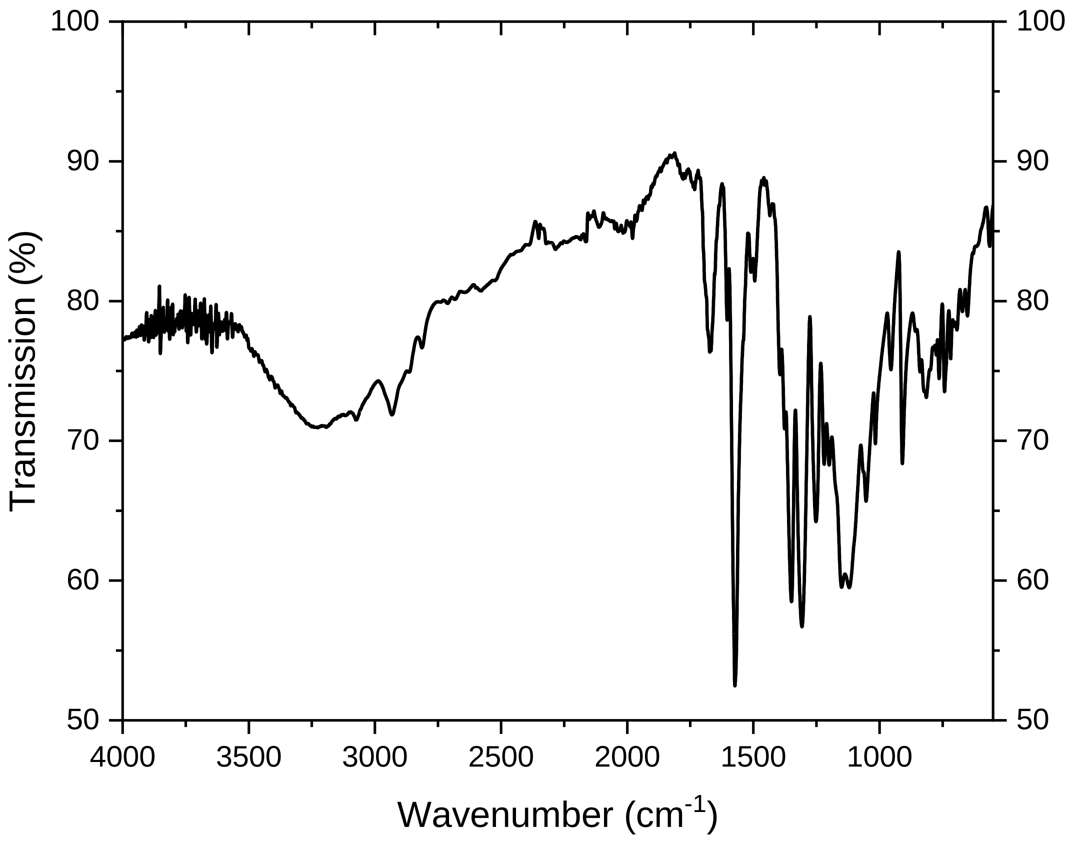

Figure S90: IR-spectrum of **6a**.

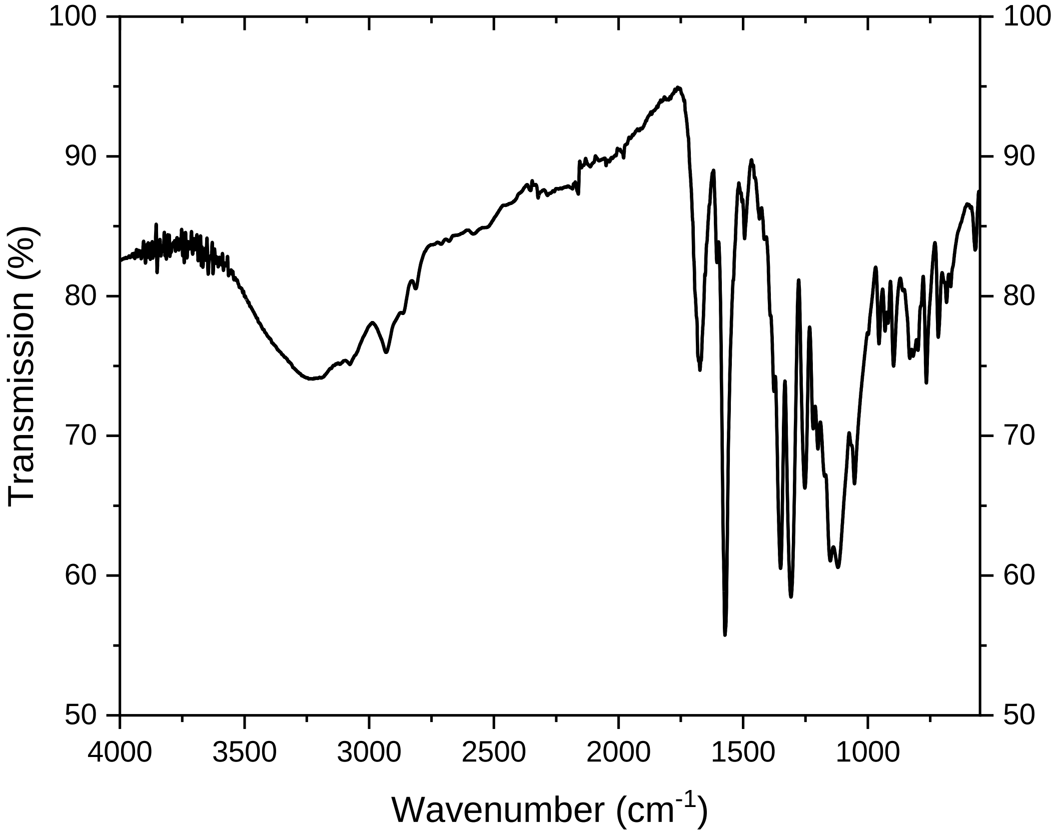

Figure S91: IR-spectrum of **6b**.

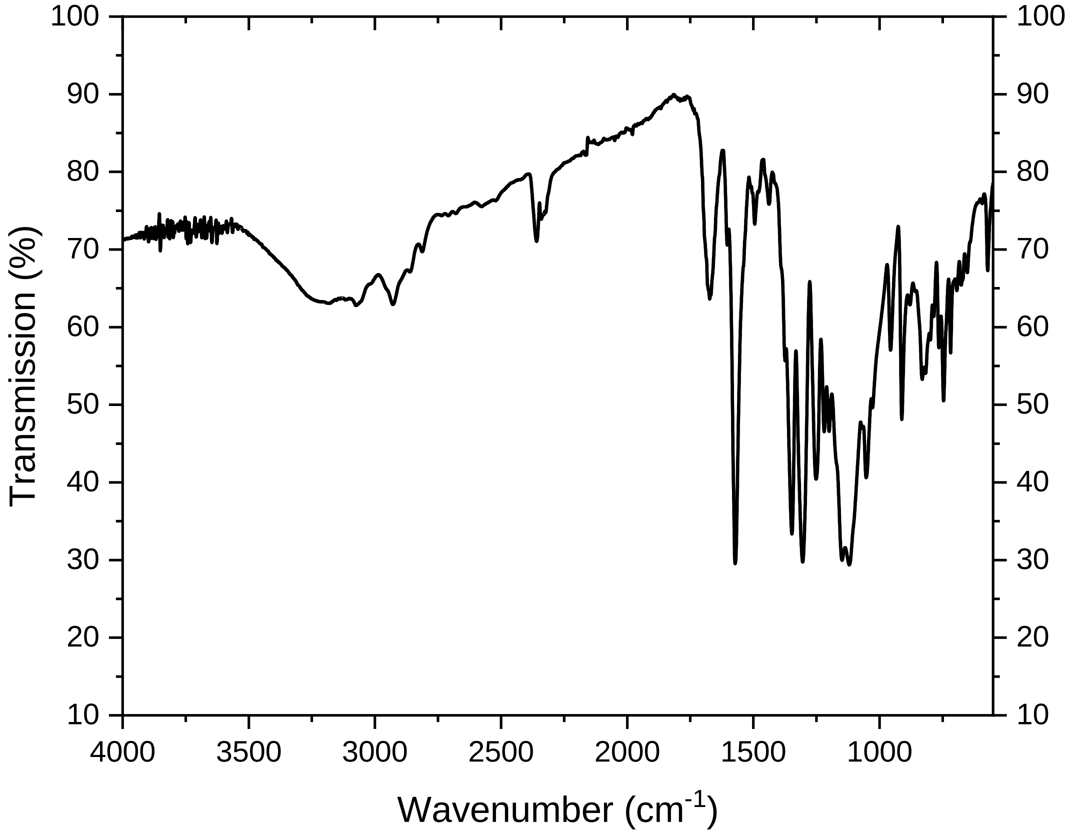

Figure S92: IR-spectrum of **6c**.

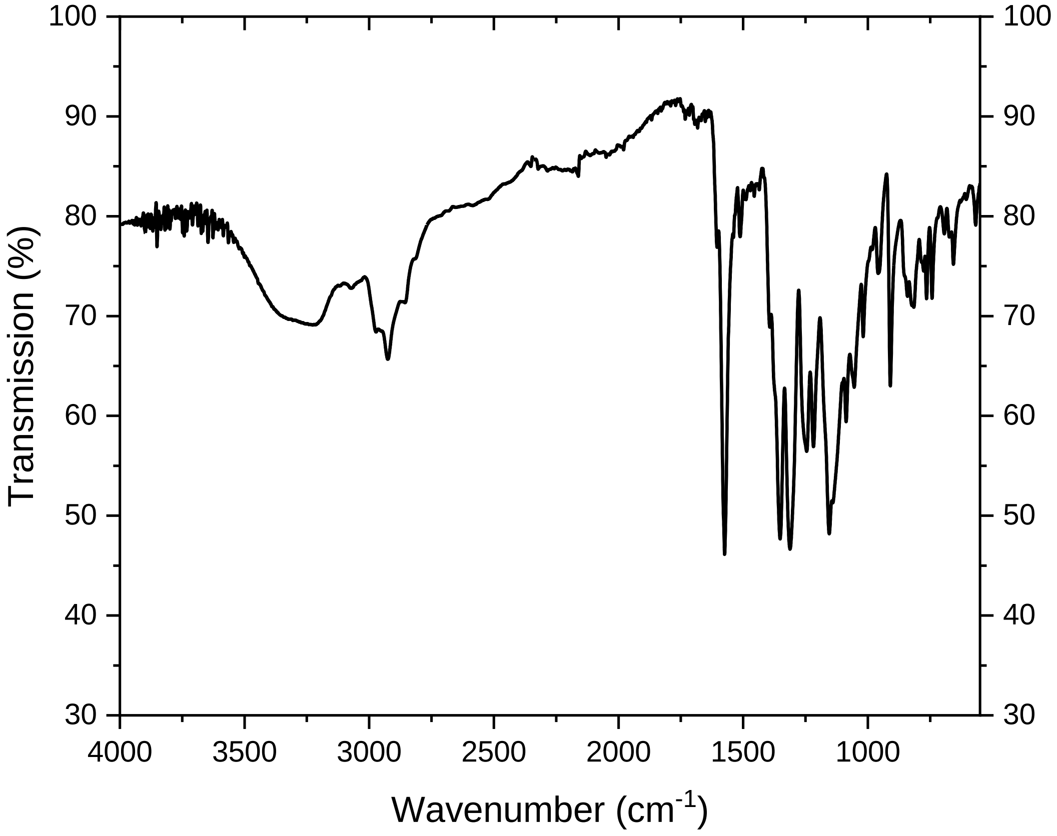

Figure S93: IR-spectrum of **8**.

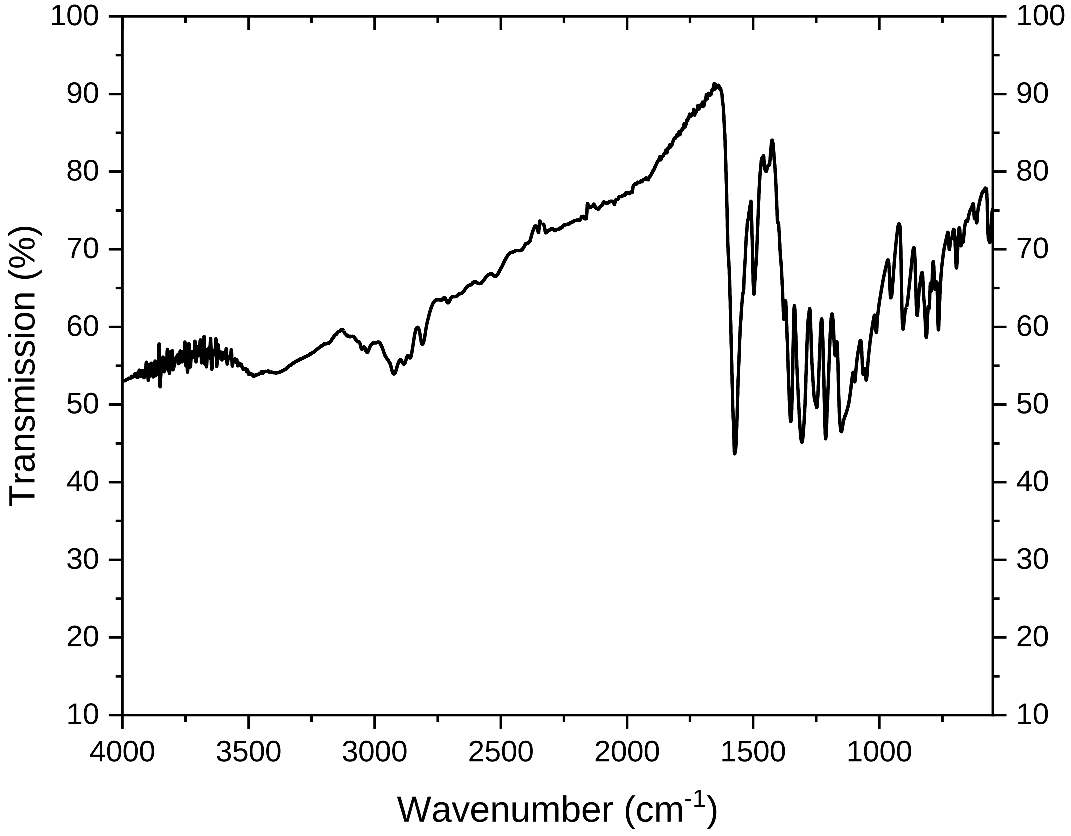

Figure S94: IR-spectrum of **10a**.

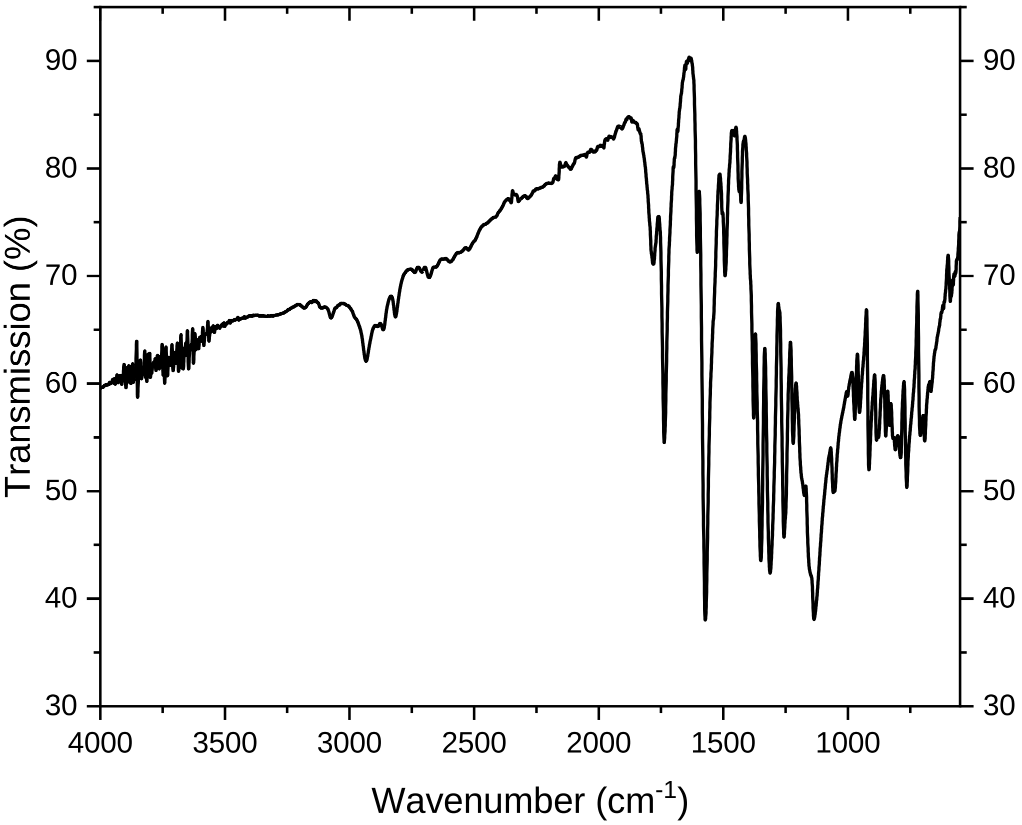

Figure S95: IR-spectrum of **10b**.

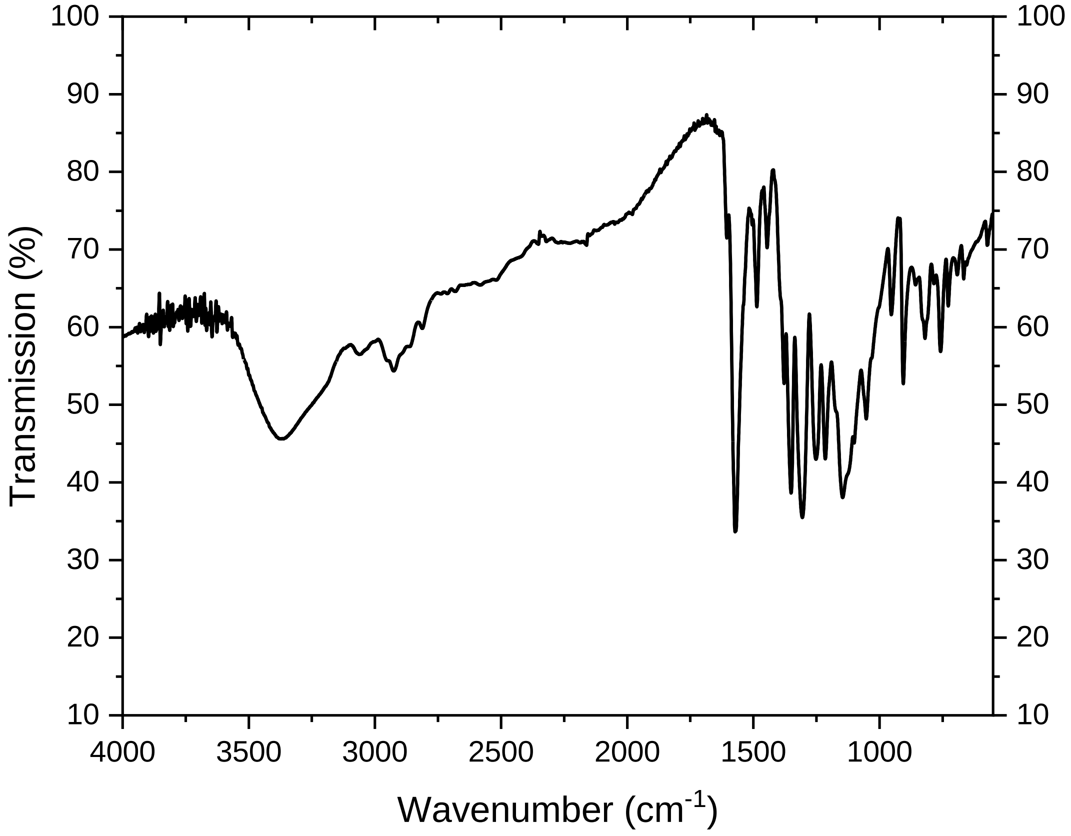

Figure S96: IR-spectrum of **10c**.

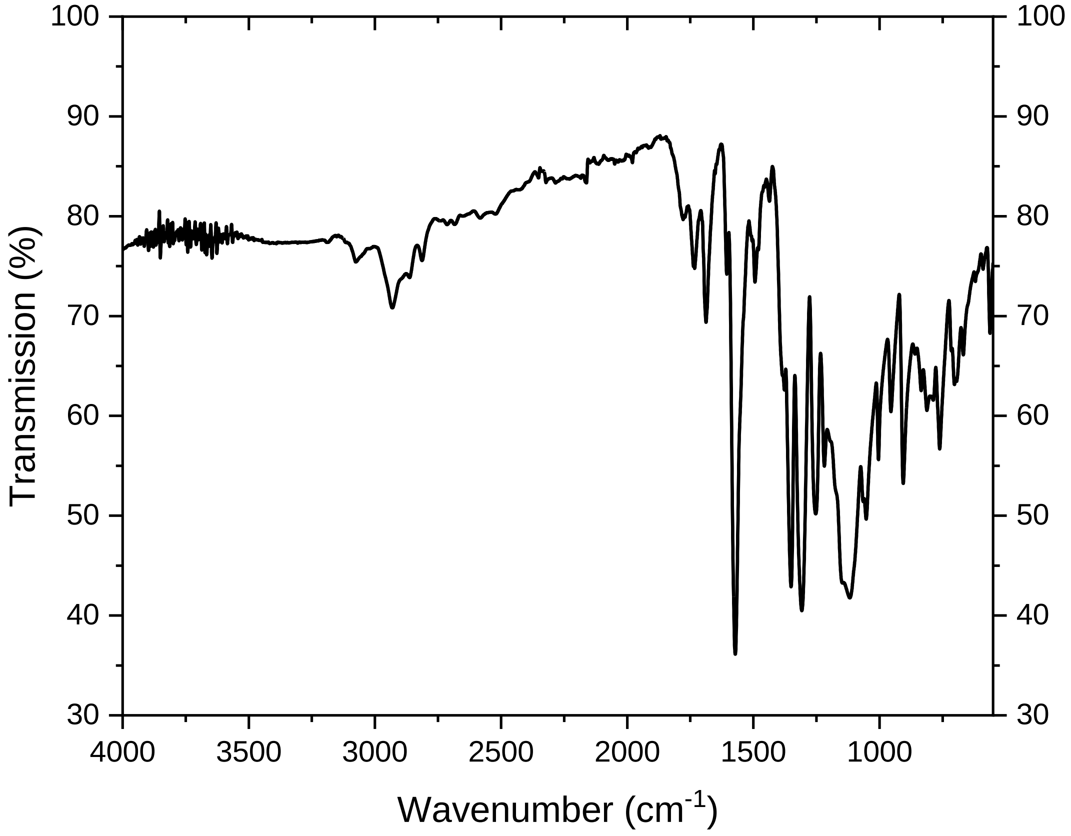

Figure S97: IR-spectrum of **12.**

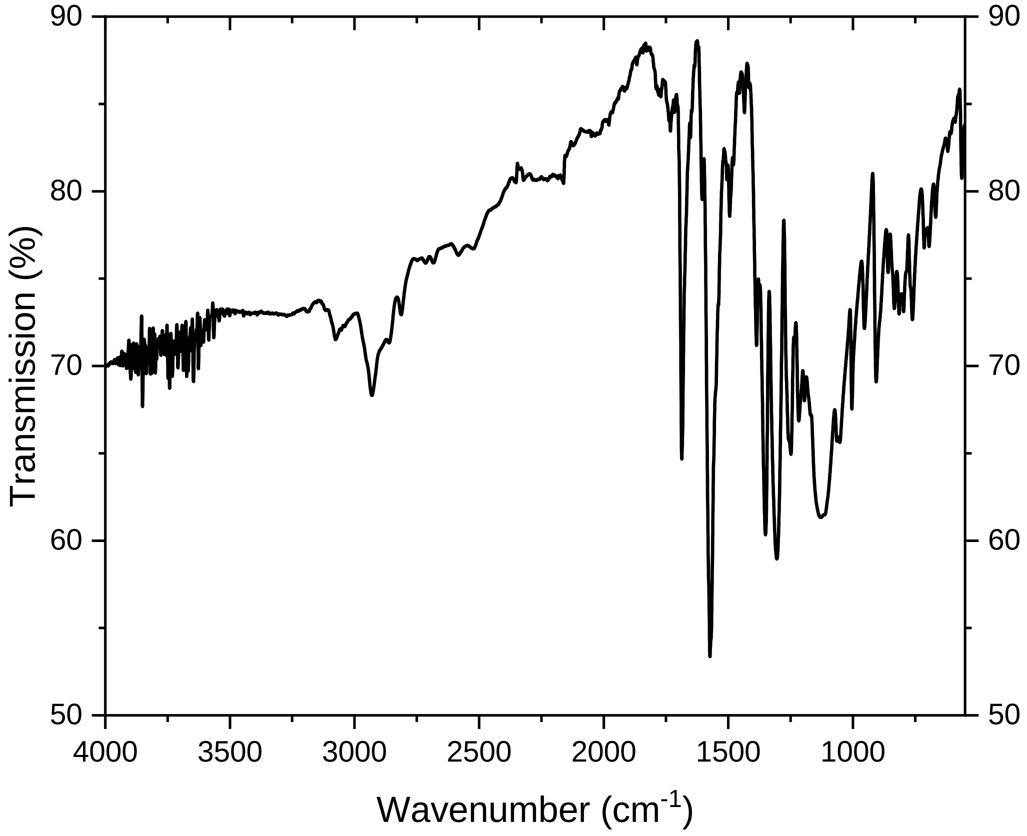

Figure S98: IR-spectrum of **13.**

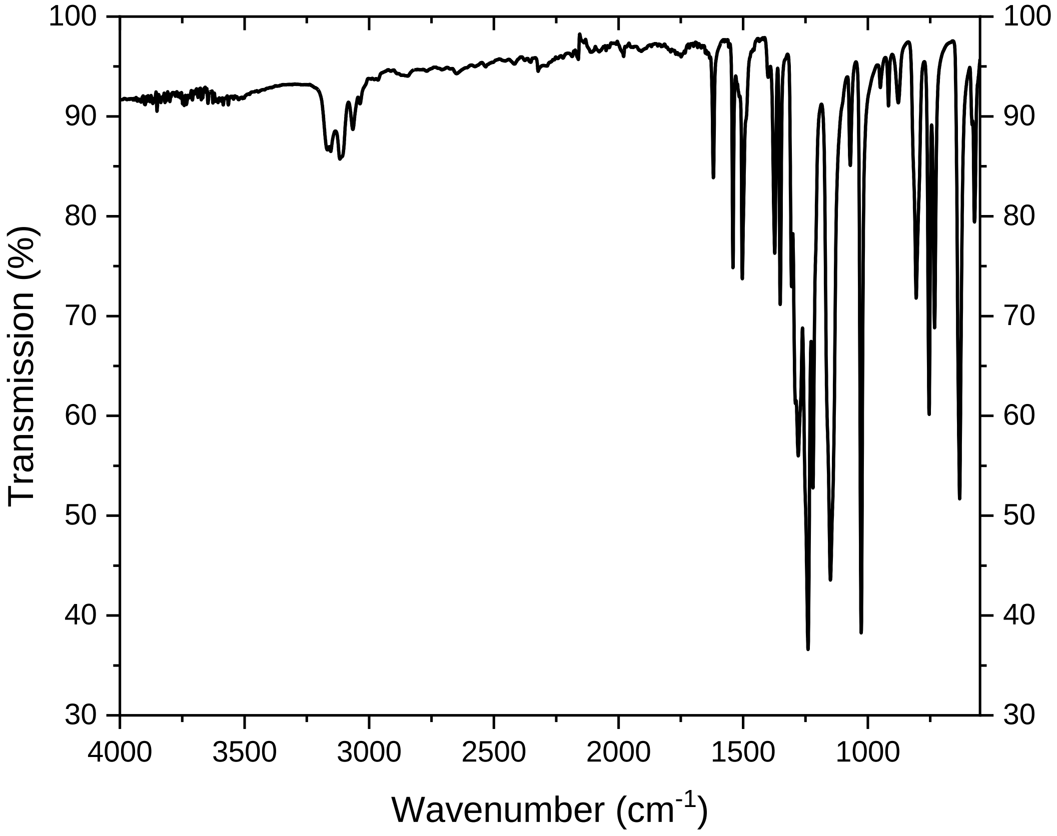

Figure S99: IR-spectrum of copper complex [Cu(OTf)$_2$(impy)$_4$].

4.4 HRMS spectra

Analysis Info

Analysis Name	D:\Data_2020\DKFZKopka\icr37432_000001.d	Acquisition Date	2/7/2020 2:15:11 PM
Method	ESI pos HPmix 200-1800	Instrument	ICR Apex-Qe
Sample Name	TK164	Operator	I.Mitsch
Comment	Kanagasundaram, DKFZ/E030: TK164 in DCM/MeOH		

Acquisition Parameters

Accumulations	16	Collision Gas Flow Rate	0.5 L/sec	Capillary Entrance	4200.0 V
Broadband Low Mass	173.2 m/z	Collision Energy	0.5 eV	Calibration Date	Tue Jan 14 02:13:10 2020
Broadband High Mass	2500.0 m/z	Collision Cell RF	1200.0 V		
Data Acquisition Size	2097152	Q1 Resolution	5.0		
		Q1 Mass	200.000 m/z		

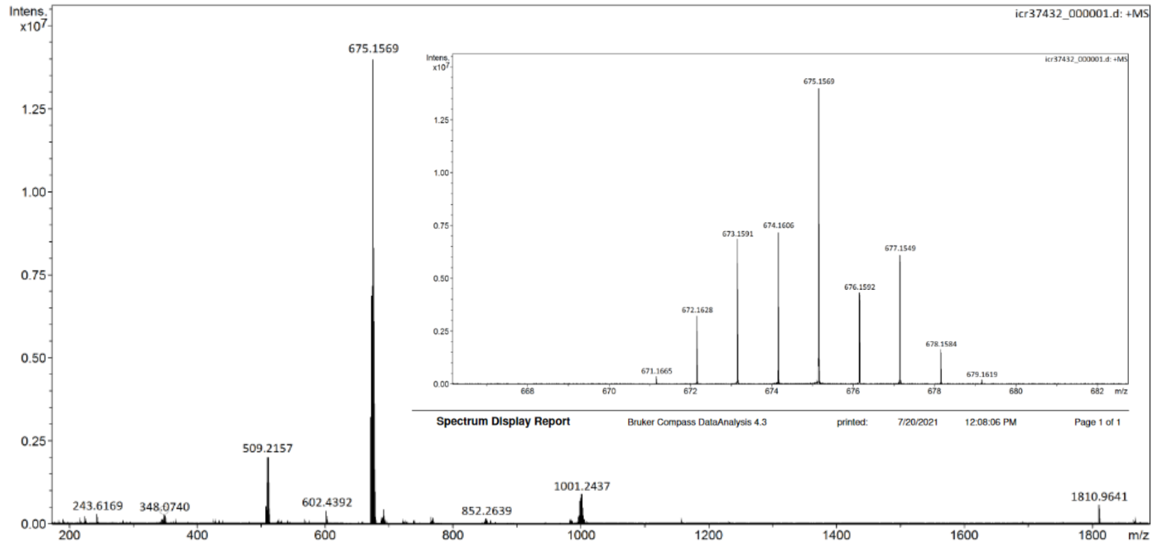

Spectrum Display Report Bruker Compass DataAnalysis 4.3 printed: 7/20/2021 1:50:36 PM Page 1 of 1

Figure S100: HR-ESI-MS of **2a** in DCM/MeOH.

Analysis Info

Analysis Name	D:\Data_2020\Kopka DKFZ\icr37182_000001.d	Acquisition Date	1/15/2020 11:25:51 AM
Method	ESI pos HPmix 200-1800	Instrument	ICR Apex-Qe
Sample Name	TK153	Operator	I.Mitsch
Comment	Kanagasundaram, DKFZ/Kopka: TK153 in DCM/MeOH		

Acquisition Parameters

Accumulations	16	Collision Gas Flow Rate	0.5 L/sec	Capillary Entrance	4200.0 V
Broadband Low Mass	173.2 m/z	Collision Energy	0.5 eV	Calibration Date	Tue Jan 14 02:13:10 2020
Broadband High Mass	2500.0 m/z	Collision Cell RF	1200.0 V		
Data Acquisition Size	2097152	Q1 Resolution	5.0		
		Q1 Mass	200.000 m/z		

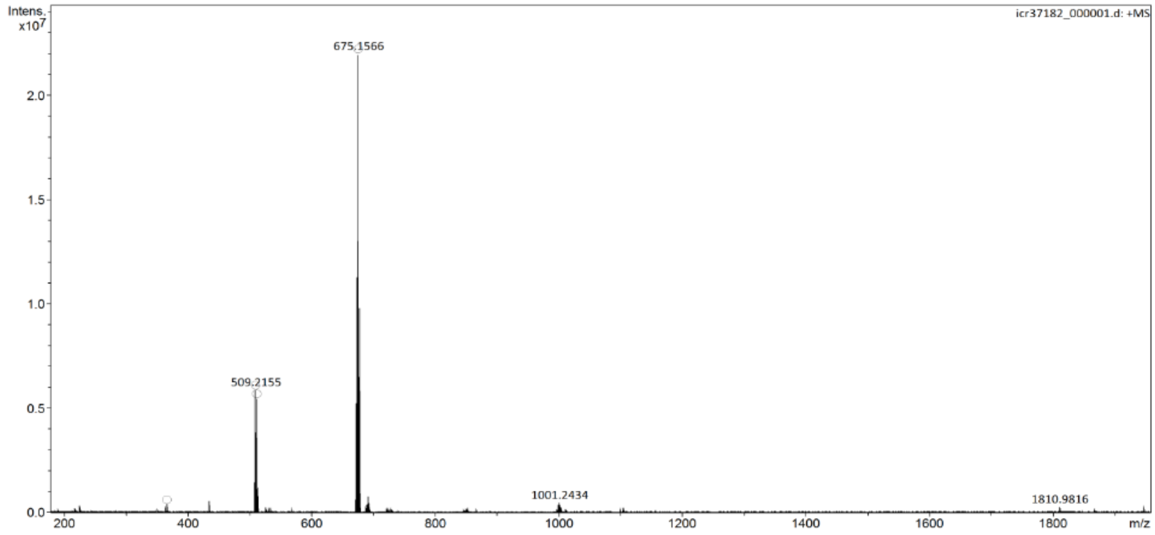

Spectrum Display Report Bruker Compass DataAnalysis 4.3 printed: 7/20/2021 11:48:49 AM Page 1 of 1

Figure S101: HR-ESI-MS of **2b** in DCM/MeOH.

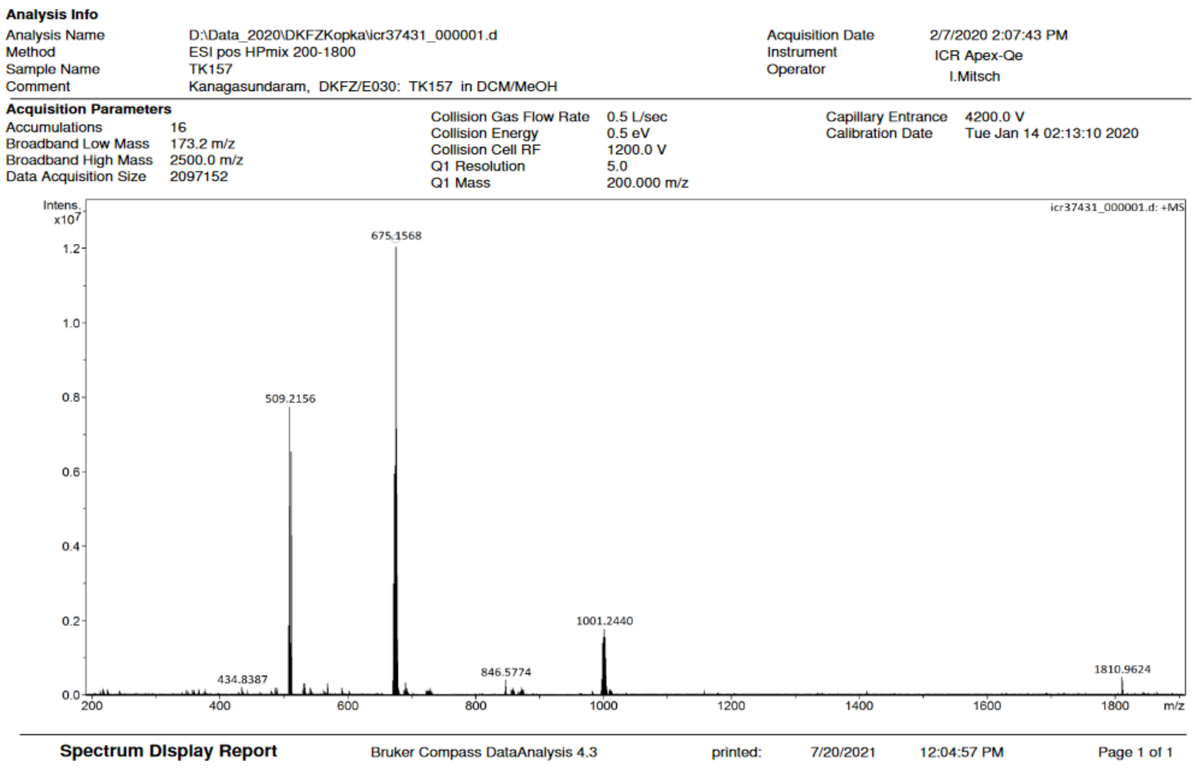

Figure S102: HR-ESI-MS of **2c** in DCM/MeOH.

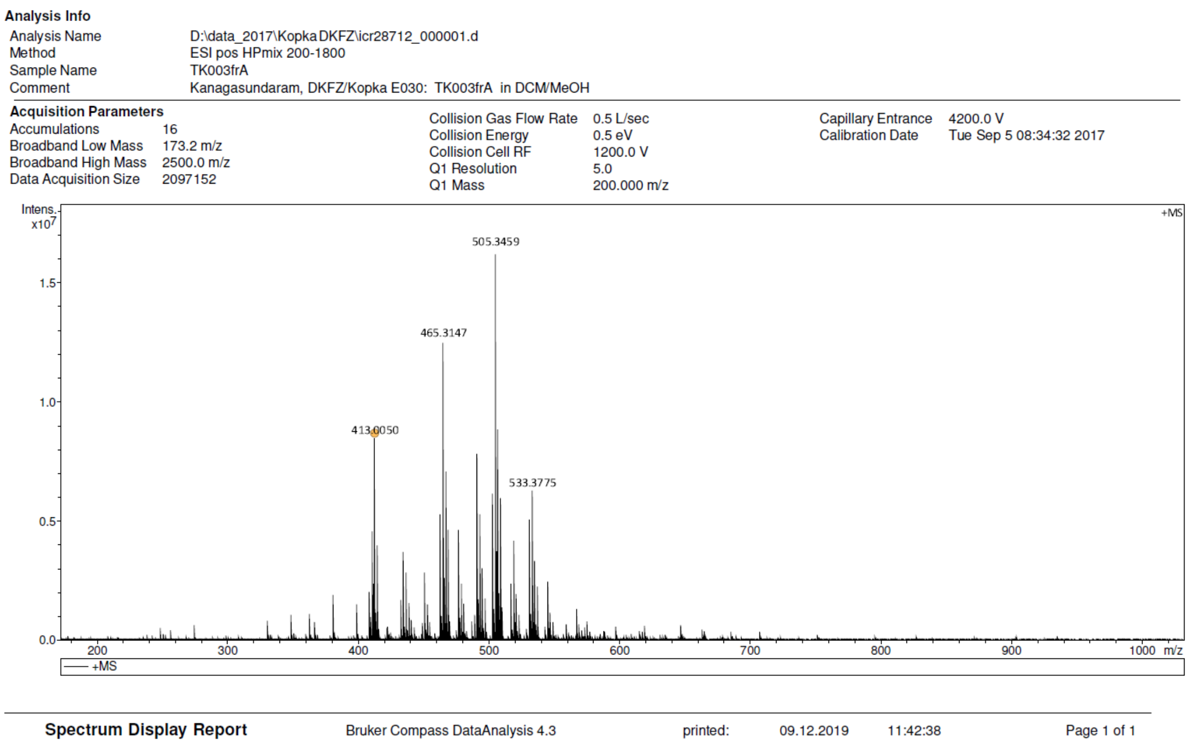

Figure S103: HR-ESI-MS of **4** in DCM/MeOH.

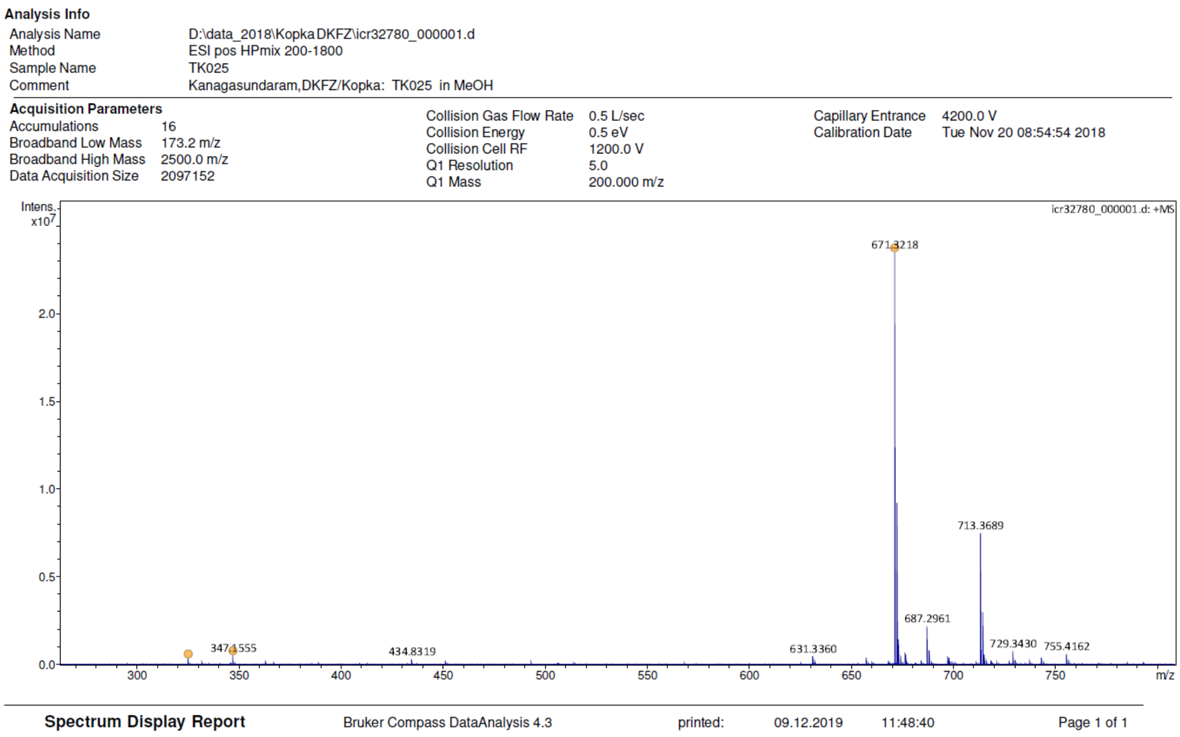

Figure S104: HR-ESI-MS of **5** in MeOH.

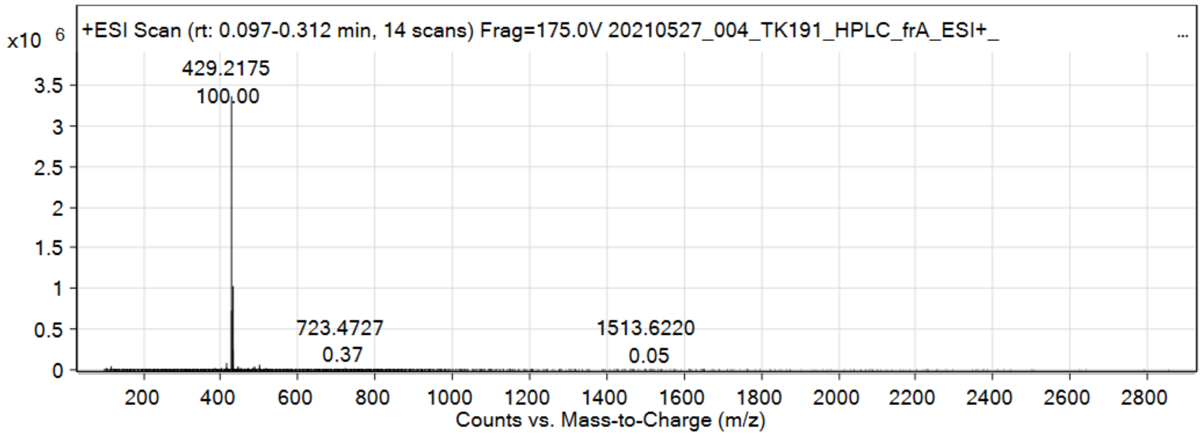

Figure S105: HR-ESI-MS in positive modus of **6a** in acetonitrile.

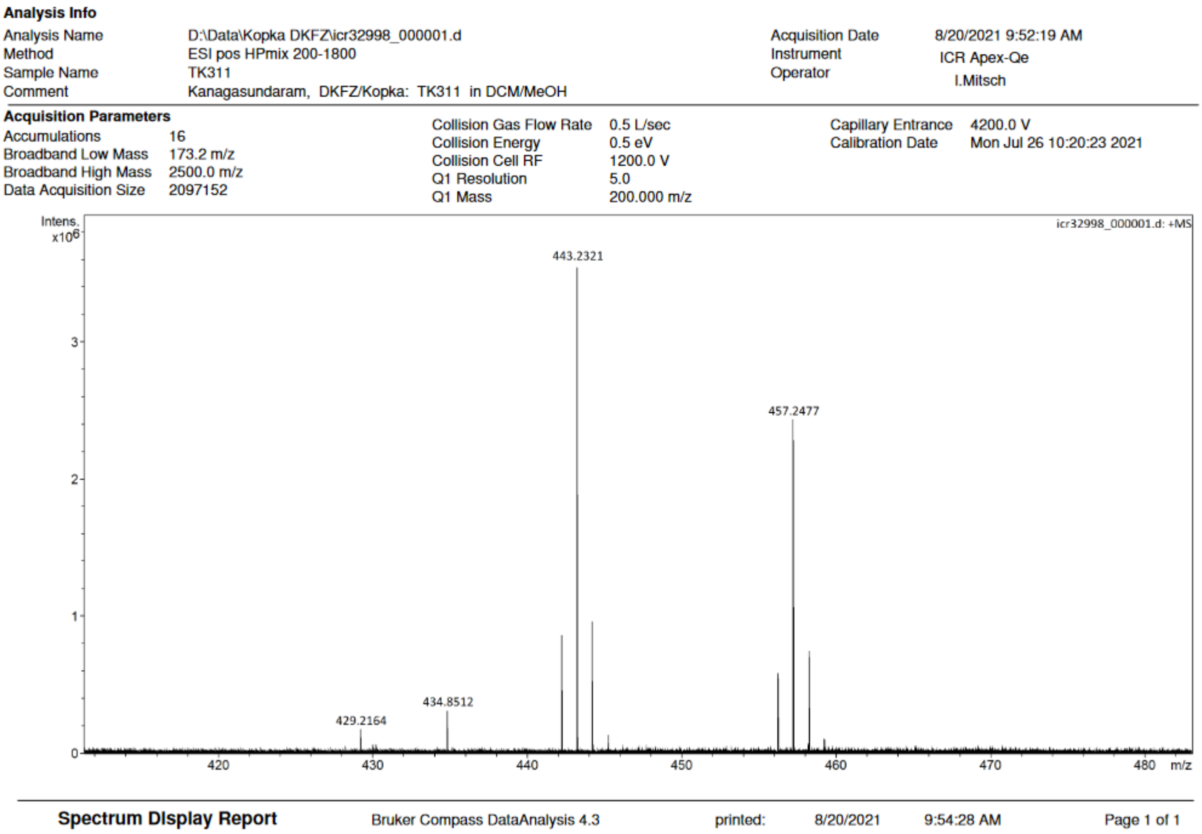

Figure S106: HR-ESI-MS of **6b** in DCM/MeOH.

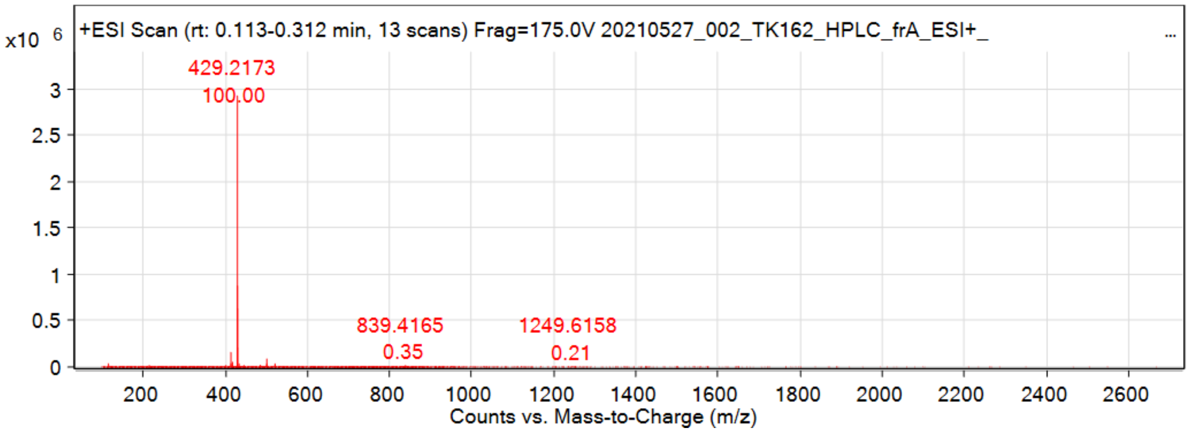

Figure S107: HR-ESI-MS in positive modus of **6c** in acetonitrile.

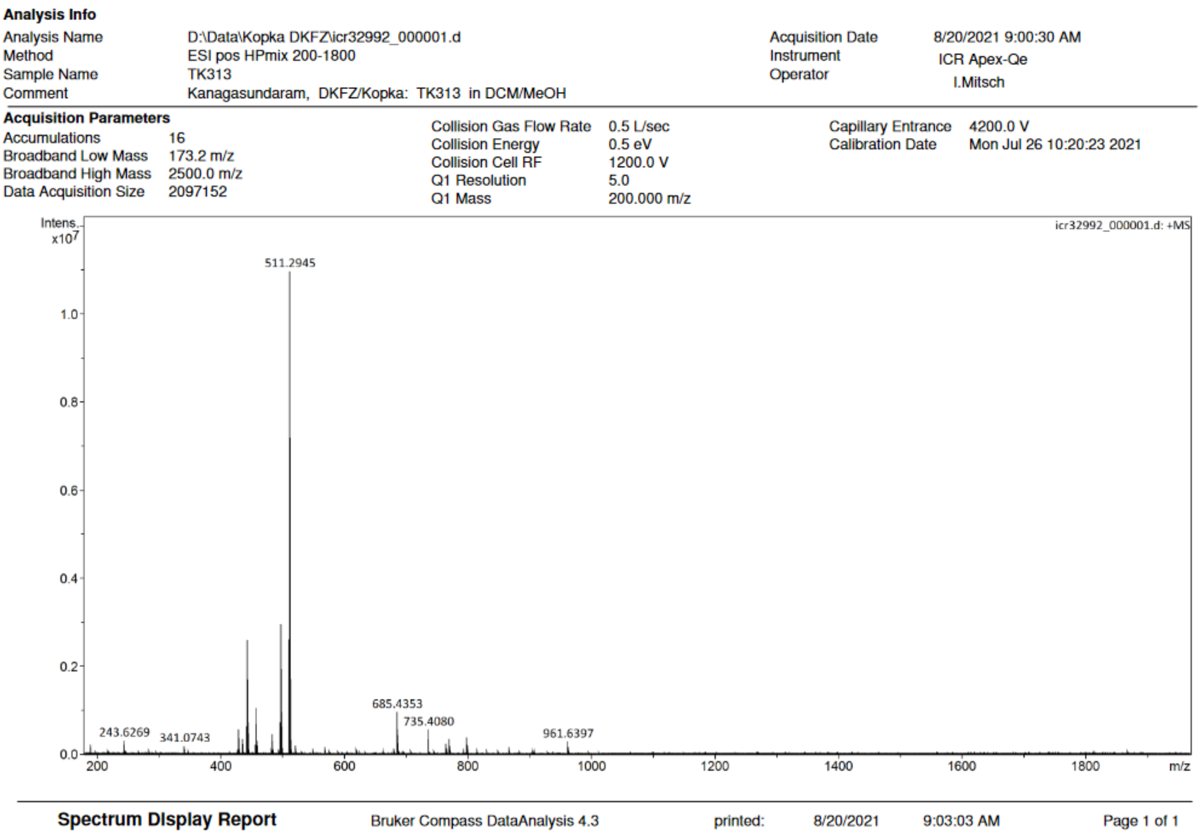

Figure S108: HR-ESI-MS of **8** in DCM/MeOH.

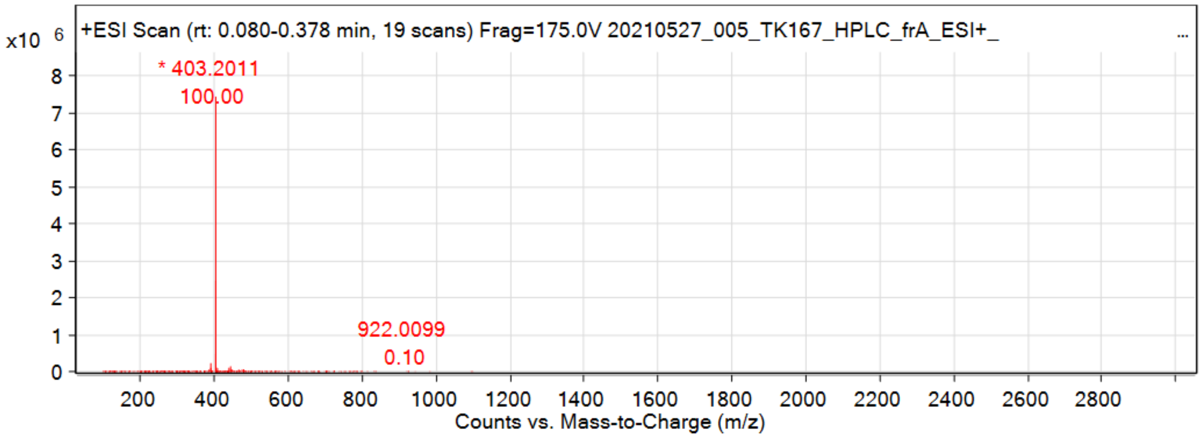

Figure S109: HR-ESI-MS in positive modus of **10a** in acetonitrile.

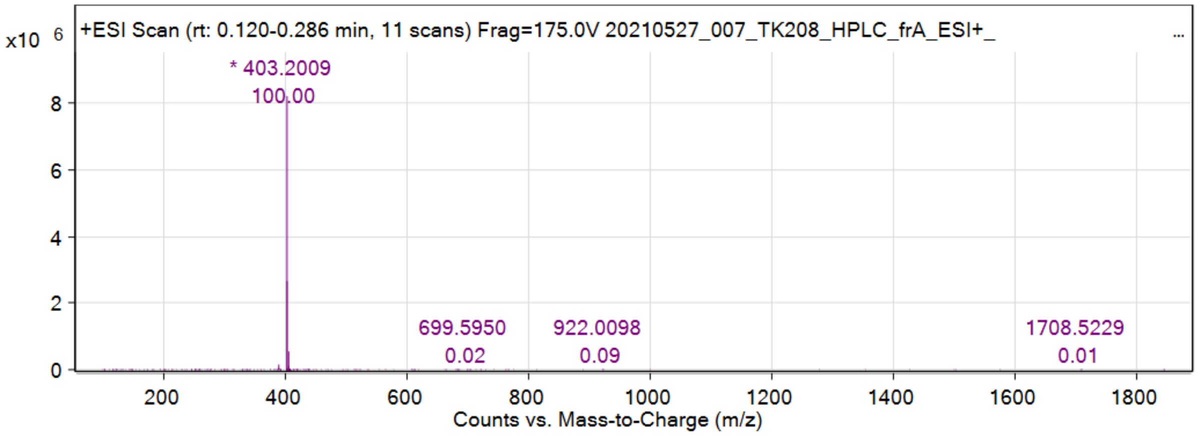

Figure S110: HR-ESI-MS in positive modus of **10b** in acetonitrile.

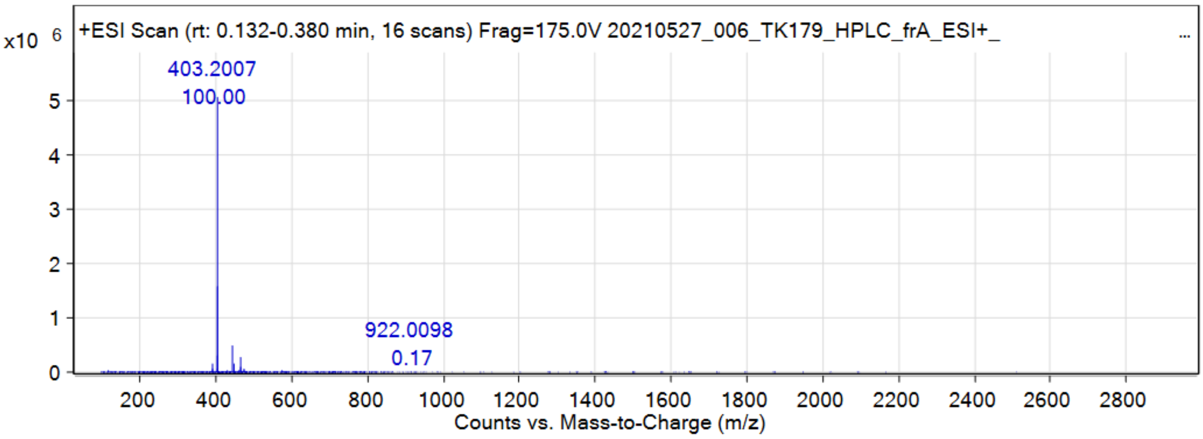

Figure S111: HR-ESI-MS in positive modus of **10c** in acetonitrile.

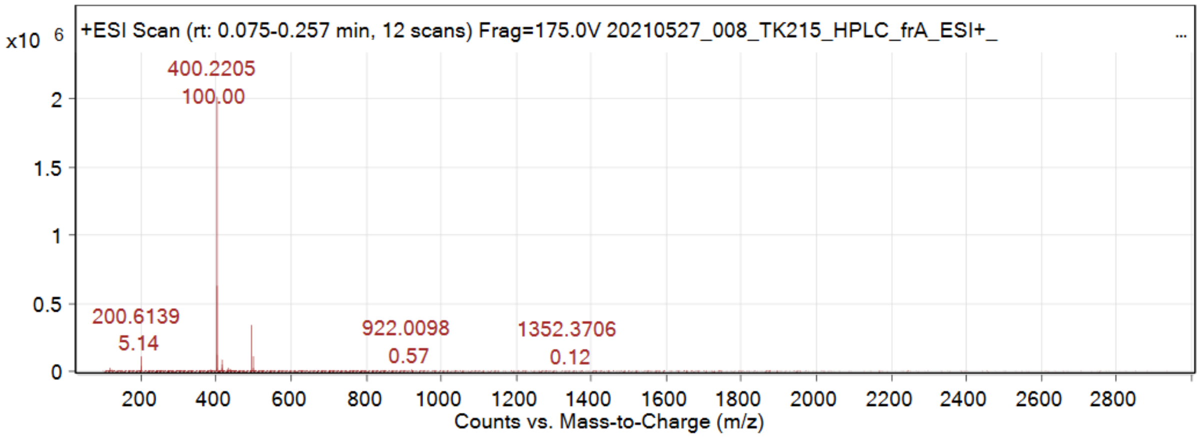

Figure S112: HR-ESI-MS in positive modus of **12** in acetonitrile.

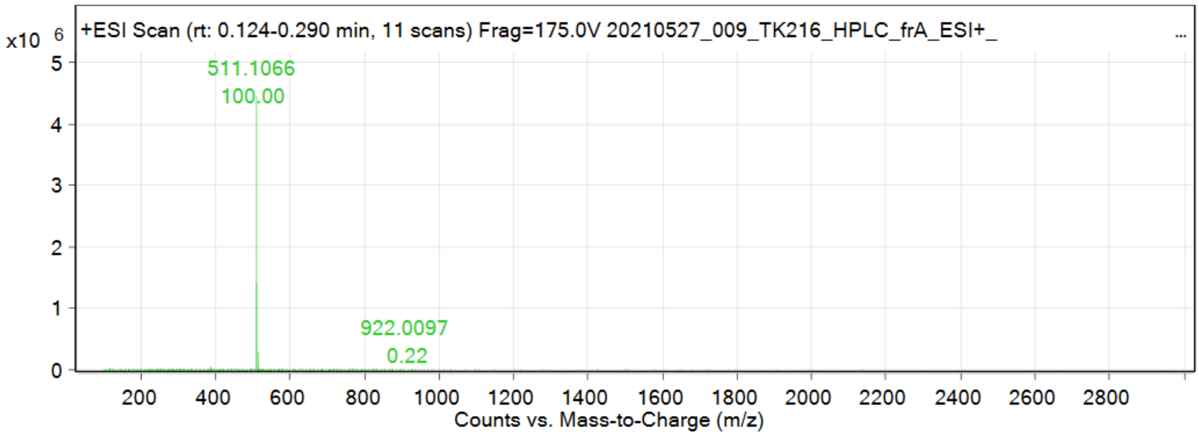

Figure S113: HR-ESI-MS in positive modus of **13** in acetonitrile.

4.5 HPLC chromatograms

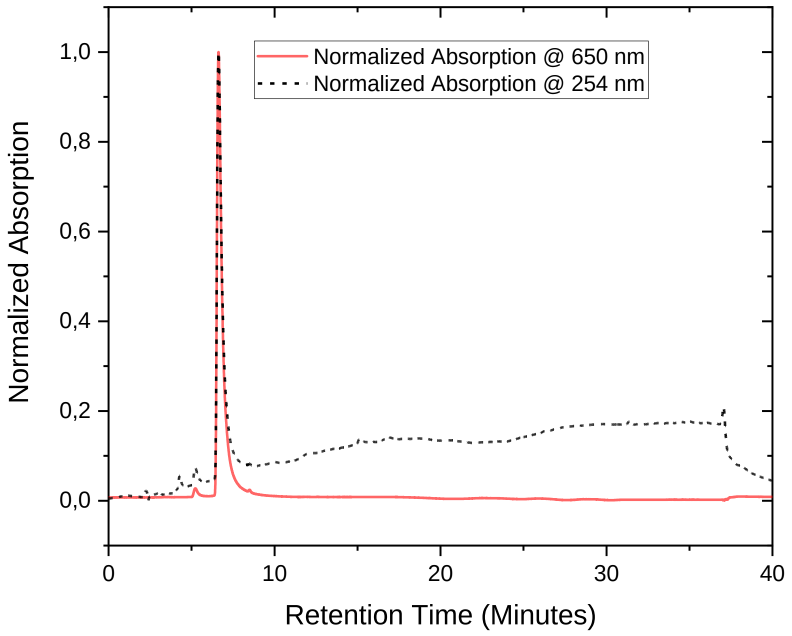

Figure S114: Normalized analytical HPLC chromatogram of **6a** (R_t= 6.6 min). HPLC-Method (*system* 5, HPLC 45–95). The absorption was detected at a wavelength of 254 nm and 650 nm.

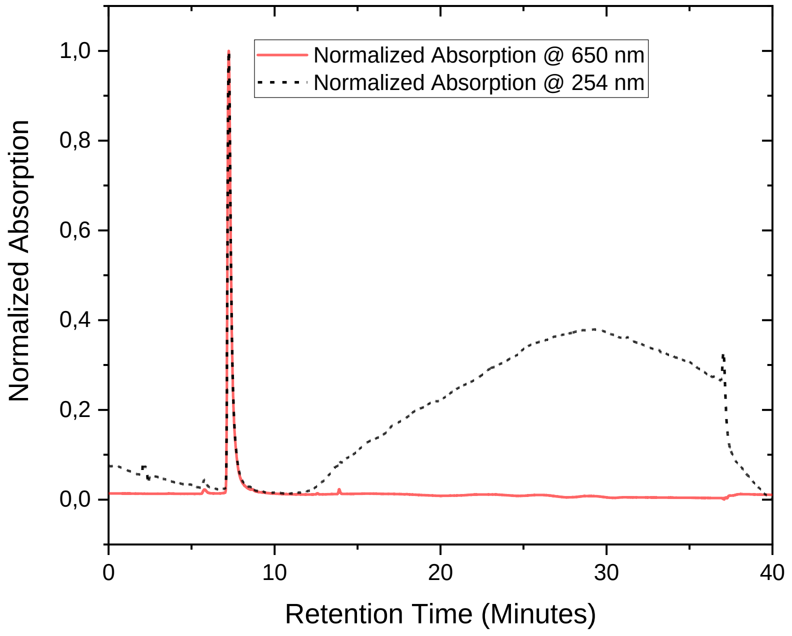

Figure S115: Normalized analytical HPLC chromatogram of **6b** (R_t= 7.2 min). HPLC-Method (*system* 5, HPLC 45–95). The absorption was detected at a wavelength of 254 nm and 650 nm.

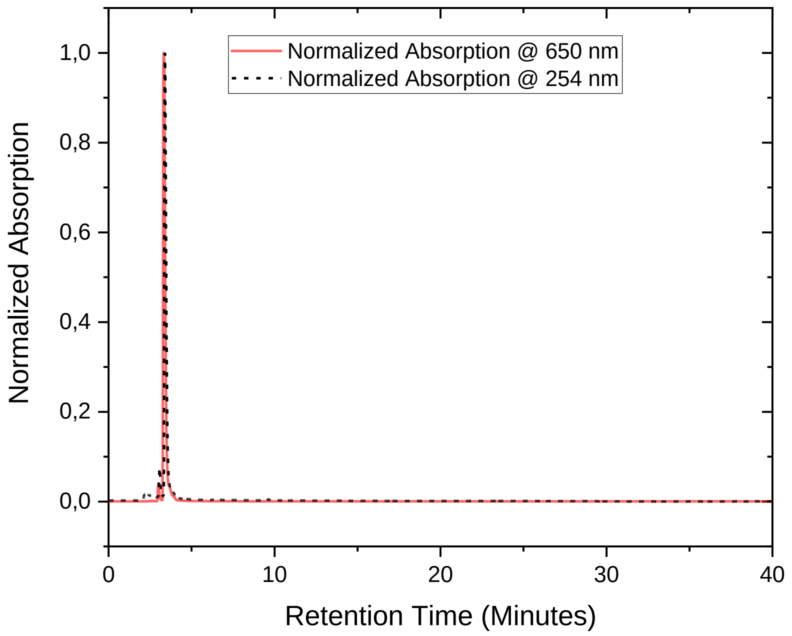

Figure S116: Normalized analytical HPLC chromatogram of **6c** (R_t= 3.4 min). HPLC-Method (*system* 5, HPLC 45–95). The absorption was detected at a wavelength of 254 nm and 650 nm.

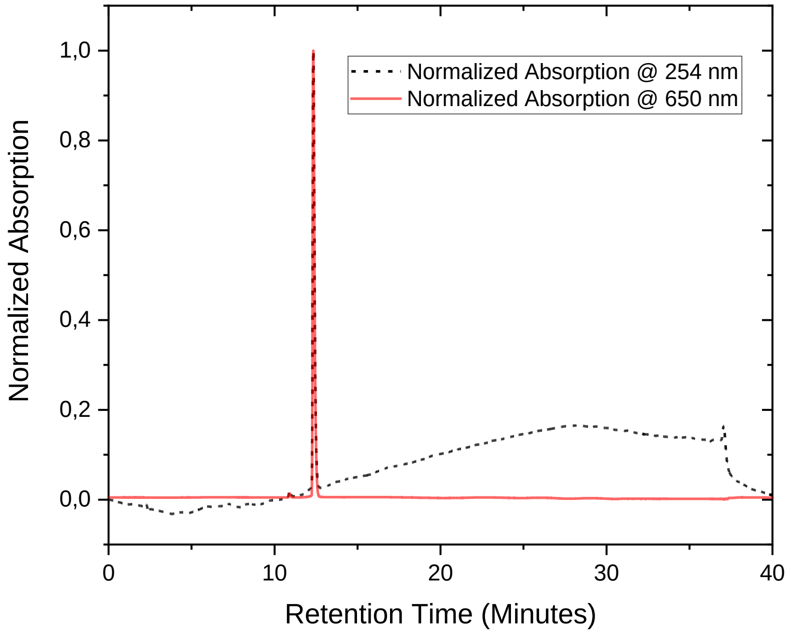

Figure S117: Normalized analytical HPLC chromatogram of **10a** (R_t= 12.3 min). HPLC-Method (*system* 5, HPLC 45–95). The absorption was detected at a wavelength of 254 nm and 650 nm.

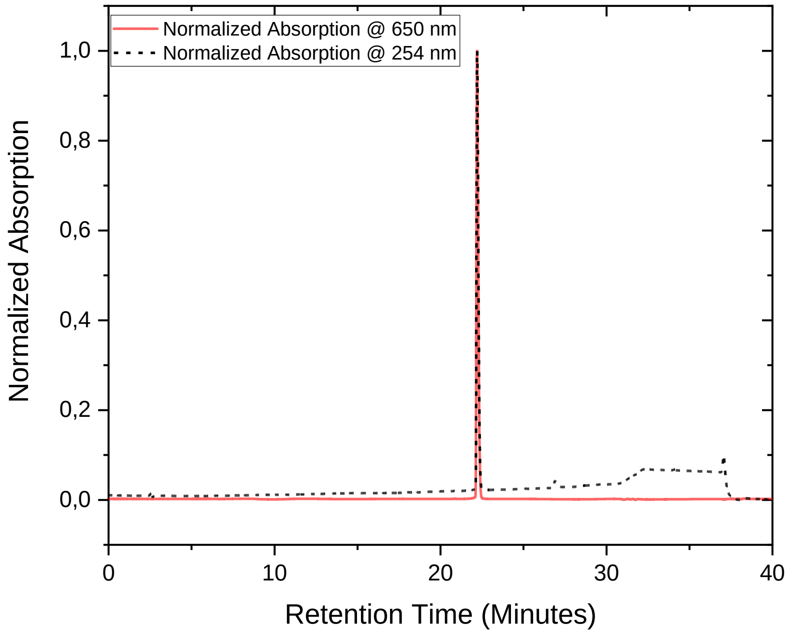

Figure S118: Normalized analytical HPLC chromatogram of **10b** (R_t= 22.2 min). HPLC-Method (*system* 5, HPLC 25–75). The absorption was detected at a wavelength of 254 nm and 650 nm.

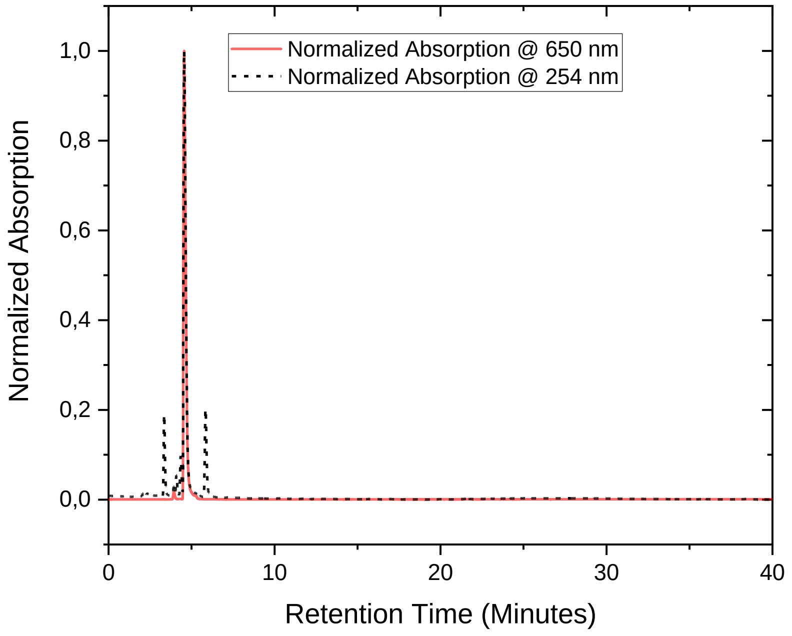

Figure S119: Normalized analytical HPLC chromatogram of **10c** (R_t= 4.6 min). HPLC-Method (*system* 5, HPLC 45–95). The absorption was detected at a wavelength of 254 nm and 650 nm.

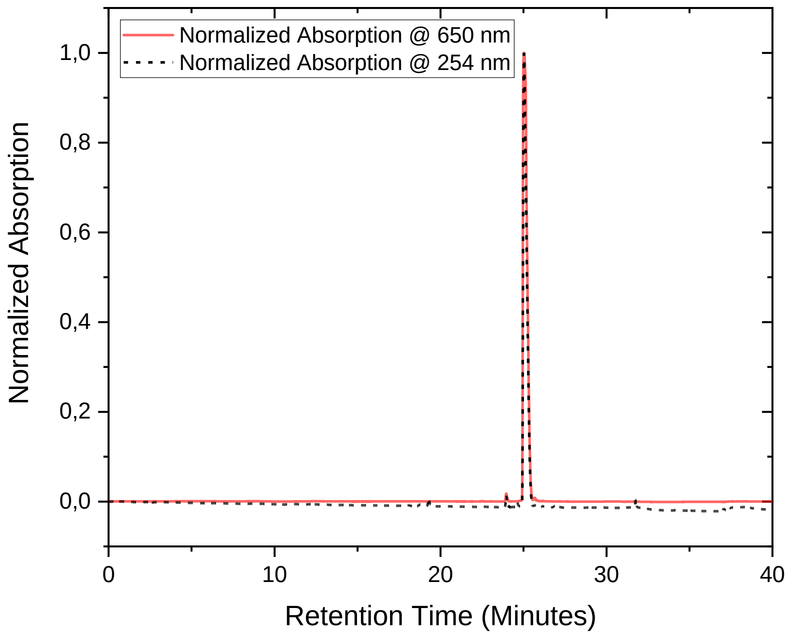

Figure S120: Normalized analytical HPLC chromatogram of **13** (R_t= 25.0 min). HPLC-Method (*system* 5, HPLC 25–75). The absorption was detected at a wavelength of 254 nm and 650 nm.
